# Design, synthesis, and evaluation of novel hydrazide and hydroxamate derivatives as potent antileukemic agents with preferential HDAC-2 inhibitory activity over class I isoform

**DOI:** 10.1039/d6ra08049a

**Published:** 2026-11-03

**Authors:** Fatma Elzahraa Ali, Abdullah Yahya Abdullah Alzahrani, Bandar A. Alyami, Stefan Bräse, Anber F. Mohammed, Bahaa G. M. Youssif

**Affiliations:** a Department of Pharmaceutical Organic Chemistry, Faculty of Pharmacy, Assiut University Assiut 71526 Egypt bgyoussif2@gmail.com (0020)-01044353895; b Faculty of Pharmacy and Drug Research, Assiut National University New Assiut City Assiut 11511 Egypt; c Department of Chemistry, Faculty of Science, King Khalid University Abha 61413 Saudi Arabia; d Department of Pharmaceutical Chemistry, College of Pharmacy, Najran University Najran Saudi Arabia; e Institute of Biological and Chemical Systems, IBCS-FMS, Karlsruhe Institute of Technology Karlsruhe 76131 Germany braese@kit.edu

## Abstract

The development of isoform-selective histone deacetylase (HDAC) inhibitors remains a promising frontier for targeted cancer therapy, offering a strategic opportunity to maximize therapeutic efficacy while minimizing the adverse side effects of pan-HDAC inhibition. In this study, we present a novel series of 4-iodo-3-nitrophenyl-capped hydrazide and hydroxamate compounds that were developed, and investigated for potential to function as effective and preferential HDAC-2 inhibitors. *In vitro* enzyme assays revealed that this new scaffold prefers the HDAC-2 isoform over HDAC-1, which differs significantly from the non-selective reference medication, Vorinostat (SAHA). Upon testing these compounds with the propidium iodide (PI) fluorescence assay across many human cancer cell lines, we observed a remarkable, tissue-specific sensitivity. The foremost derivatives (7a, 7c, and 7d) exhibited much greater efficacy in inhibiting the growth of B-cell acute lymphoblastic (SEM) cells, exhibiting IC_50_ values ranging from 3.18 to 4.17 µM, surpassing the reference drug Vorinostat, which presented an IC_50_ of 5.23 µM. Western blot analyses in SEM cells revealed that the lead compound 7a bound to its intracellular target, resulting in an approximately 59% reduction in total HDAC-2 protein levels. Flow cytometry revealed that a significant reduction in HDAC-2 led to cancer cells being arrested at the key G2/M phase of the cell cycle, with 46.22% of cells detained there compared to merely 7.21% in untreated controls. Subsequent analysis using Annexin V/PI labeling demonstrated that this cell cycle arrest results in a significant elevation of programmed cell death, increasing total apoptosis to 31.12%, while maintaining non-specific necrosis at a minimal 4.29%. Taken together, these findings indicate that the 4-iodo-3-nitrophenyl compounds are effective tools for the targeted treatment of leukemia, displaying preferential HDAC-2 inhibitory activity among the evaluated class I isoforms.

## Abstract 2

Developing isoform-selective HDAC inhibitors is a promising cancer therapy, maximizing efficacy while minimizing side effects of pan-HDAC inhibition.

## Graphical abstract

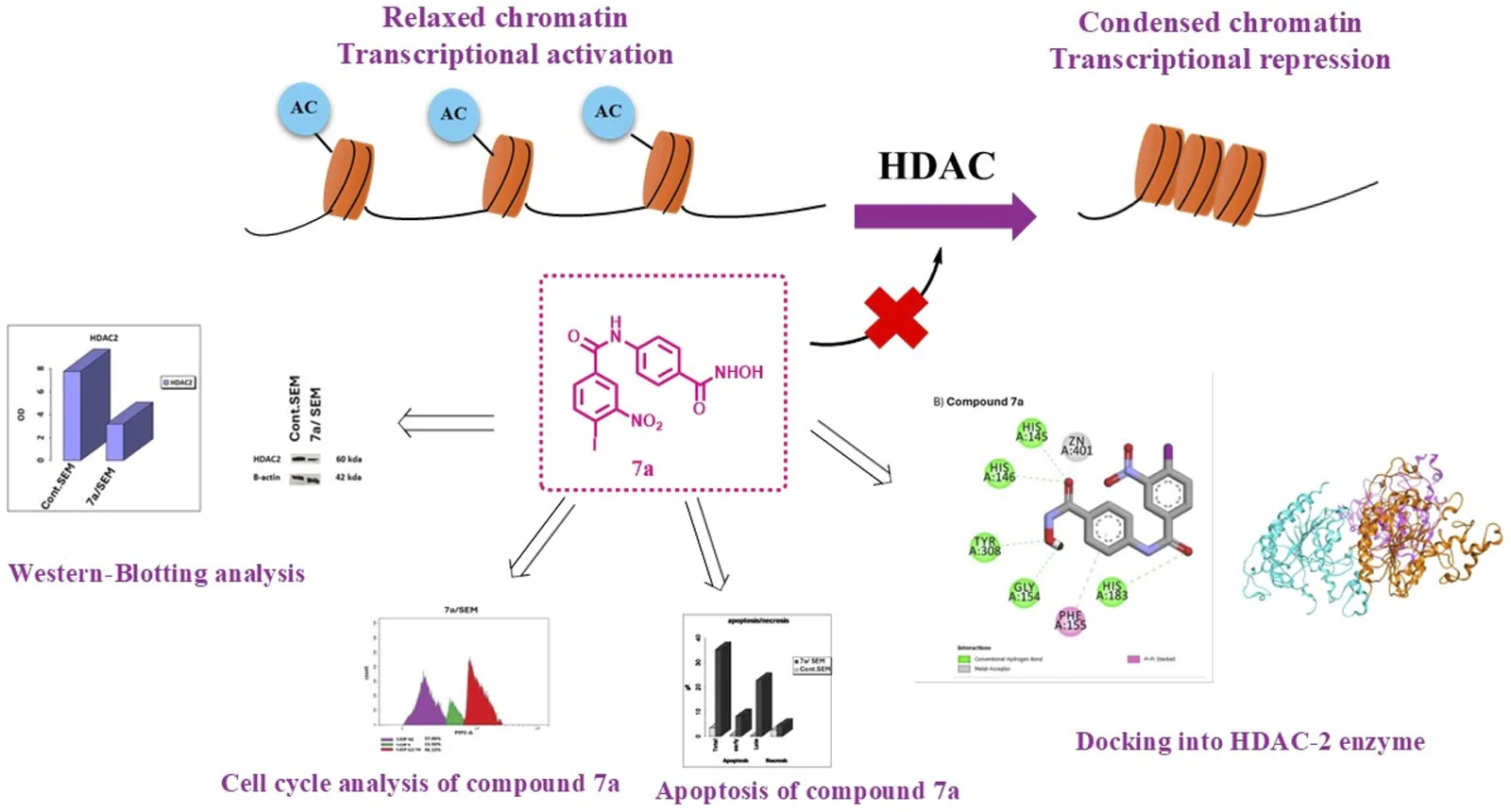

## 1 Introduction

Cancer is one of the major causes of death worldwide, and it is frequently associated with severe consequences such as metastasis, medication resistance, and systemic toxicity.^1,2^ Conventional treatments, such as chemotherapy and radiation, frequently fail to remove resistant cancer stem cells and have a limited therapeutic window, resulting in significant damage to normal tissues.^3,4^ Furthermore, the rapid establishment of adaptive resistance mechanisms in advanced cancers continues to weaken the efficacy of traditional regimens. Thus, there is an urgent clinical need to develop new, highly specific therapeutic approaches that selectively target oncogenic pathways while avoiding unwanted side effects and circumventing known resistance mechanisms.^5,6^

Histone deacetylase (HDAC) inhibitors constitute an effective class of epigenetic treatments that rectify abnormal gene expression patterns, leading to cell cycle arrest, differentiation, and apoptosis in malignant cells.^7,8^ Nonetheless, the non-selective inhibition of many HDAC isoforms across different classes by first-generation pan-HDAC inhibitors sometimes leads to significant clinical toxicities, including bone marrow suppression, tiredness, and cardiac problems.^9,10^ Recent drug discovery initiatives have addressed these constraints by developing isoform- or class-selective HDAC inhibitors.^11^ These selective HDAC inhibitors are designed to target only the dysregulated HDAC enzymes associated with specific malignancies, thereby significantly enhancing the safety profile, reducing off-target toxicities, and improving overall treatment efficacy compared with non-selective prototypes.^12,13^

Histone deacetylase 2 (HDAC-2) is a key isoform that promotes tumor development and survival and represses tumor suppressor genes in several malignancies, making it a compelling therapeutic target.^14,15^ Currently, the pharmaceutical market lacks fully selective, regulatory-approved HDAC-2 inhibitors, despite their high therapeutic potential.^16^ This is largely due to the significant structural homology between HDAC-2 and other class I enzymes, including HDAC-1.^17,18^ The design and production of innovative small molecules exhibiting exceptional selectivity for HDAC-2 represents a significant breakthrough opportunity, as these compounds will provide a specifically tailored epigenetic intervention.^19^ The development of selective HDAC-2 agents provides a unique therapeutic benefit, addressing a significant unmet need in modern tumor therapy pathways and providing a more targeted tool for cancer treatment.

In our earlier work,^16^ we demonstrated that hybrid inhibitors targeting HDAC-2 alongside the receptor tyrosine kinases EGFR and HER-2 offer significant therapeutic advantages. By concurrently inhibiting the epigenetic machinery (HDAC-2) and growth signaling pathways (EGFR and HER-2), these dual-action therapies more effectively halt tumor proliferation and prevent malignancies from activating alternative survival mechanisms that bypass single-target inhibition.^20^ This multi-HDAC/kinase inhibition method effectively obstructs compensatory escape mechanisms, promotes apoptosis, and mitigates the development of resistance, substantiating the molecular basis for further refinement of the hybrid scaffolds.^21,22^

To expand on these results and develop more potent and selective epigenetic modulators, we designed and synthesized a new series of targeted compounds based on the classical pharmacophoric model of HDAC inhibitors. The scaffold of these newly synthesized molecules consists of four main functional parts: a 4-iodo-3-nitrophenyl scaffold as a surface-recognition cap moiety, a carboxamide (–NHCO–) connector unit, and a hydrophobic aromatic linker. The terminal end was modified with various zinc-binding groups (ZBGs), such as hydroxylamine, hydrazide, benzamide, or fluorobenzamide, to interact with the catalytic zinc ion in the enzyme pocket. The growth inhibitory potential of this new series was extensively tested against three human cancer cell lines: MDA-MB-231 (triple-negative breast cancer), A-549 (non-small cell lung cancer), and SEM (B-cell acute lymphoblastic leukemia).

### 1.1. Rational design

Following our recent success in designing multi-target inhibitors of HDAC-2/EGFR/HER-2, including an Iniparib-derived cap moiety,^16^ we proceeded to optimize this pharmacophoric framework further to improve potency, binding affinity, and structural stiffness. The conventional HDAC inhibitor pharmacophore is zinc-dependent and comprises four essential components:^23^ a surface recognition unit (cap), a polar connecting unit (CU), a hydrophobic linker, and a zinc-binding group (ZBG). In our earlier published series (compounds Ia–l, Fig. 1),^16^ the 4-iodo-3-nitrophenyl scaffold was successfully utilized as a cap moiety to concurrently target both receptor tyrosine kinases (EGFR/HER-2) and the epigenetic machinery (HDAC-2). It also suppressed the compensatory signaling pathways that tumors frequently use to evade apoptosis, resulting in a dual impact.

**Fig. 1:**
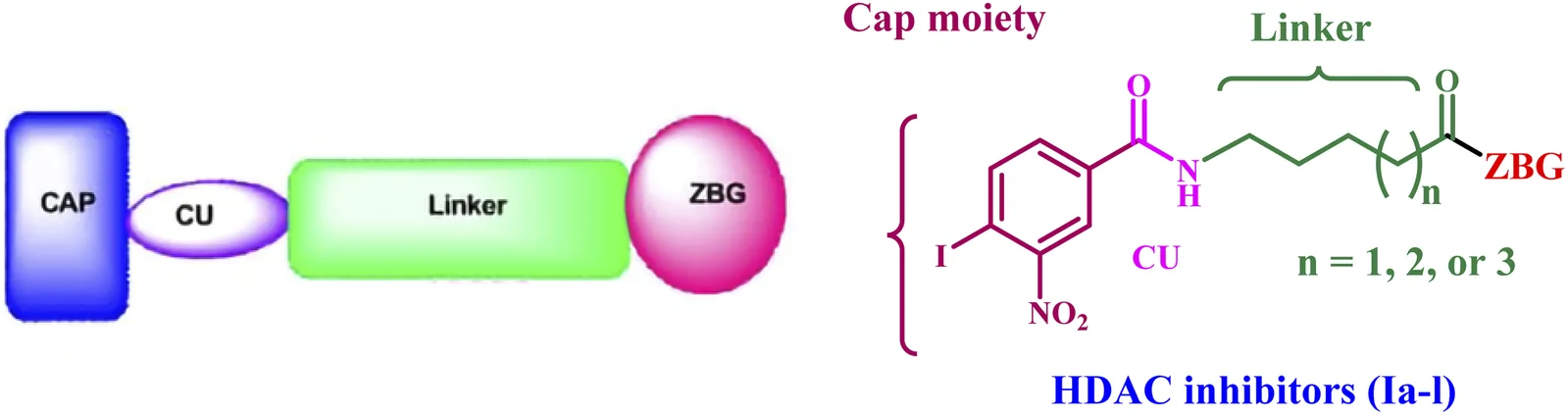
Structure of our previously published HDAC inhibitors (Ia–l).

The flexible aliphatic polymethylene linkers (four to six carbons) in our first-generation series effectively navigated the hydrophobic channel of HDAC-2; however, the saturated aliphatic chains exhibit significant conformational flexibility.^24^ This results in a considerable entropic penalty during the transition from a free solution state to a protein-bound bioactive conformation.^25^ To address this constraint and enhance structural complementarity in the target pockets, we introduce a targeted structural variation in which the flexible aliphatic linkers are replaced with rigid aromatic spacers derived from benzoic acid, phenylacetic acid, and benzylamine. Concurrently, the four thoroughly assessed zinc-binding functionalities, hydroxamic acid, hydrazide, benzamide, and 4-fluorobenzamide, were preserved to systematically examine the structure–activity relationship (SAR) of these rigidified hybrids.

This next-generation series (compounds 7a–l, Fig. 2) uses key medicinal chemistry principles to improve target interaction. Stiff phenyl rings are introduced into the linker domain, thereby generating anticipated structural constraints and reducing the number of rotatable bonds. The entropy loss upon binding to the receptor is reduced by anchoring the hybrid molecule to a pre-organized structure, thereby significantly increasing overall binding efficiency. This structural modification also improves the arrangement of interstitial rings in binding pockets. The use of stiff aromatic rings promotes robust pi–pi stacking and hydrophobic packing networks, which are not possible with standard saturated polymethylene chains.

**Fig. 2:**
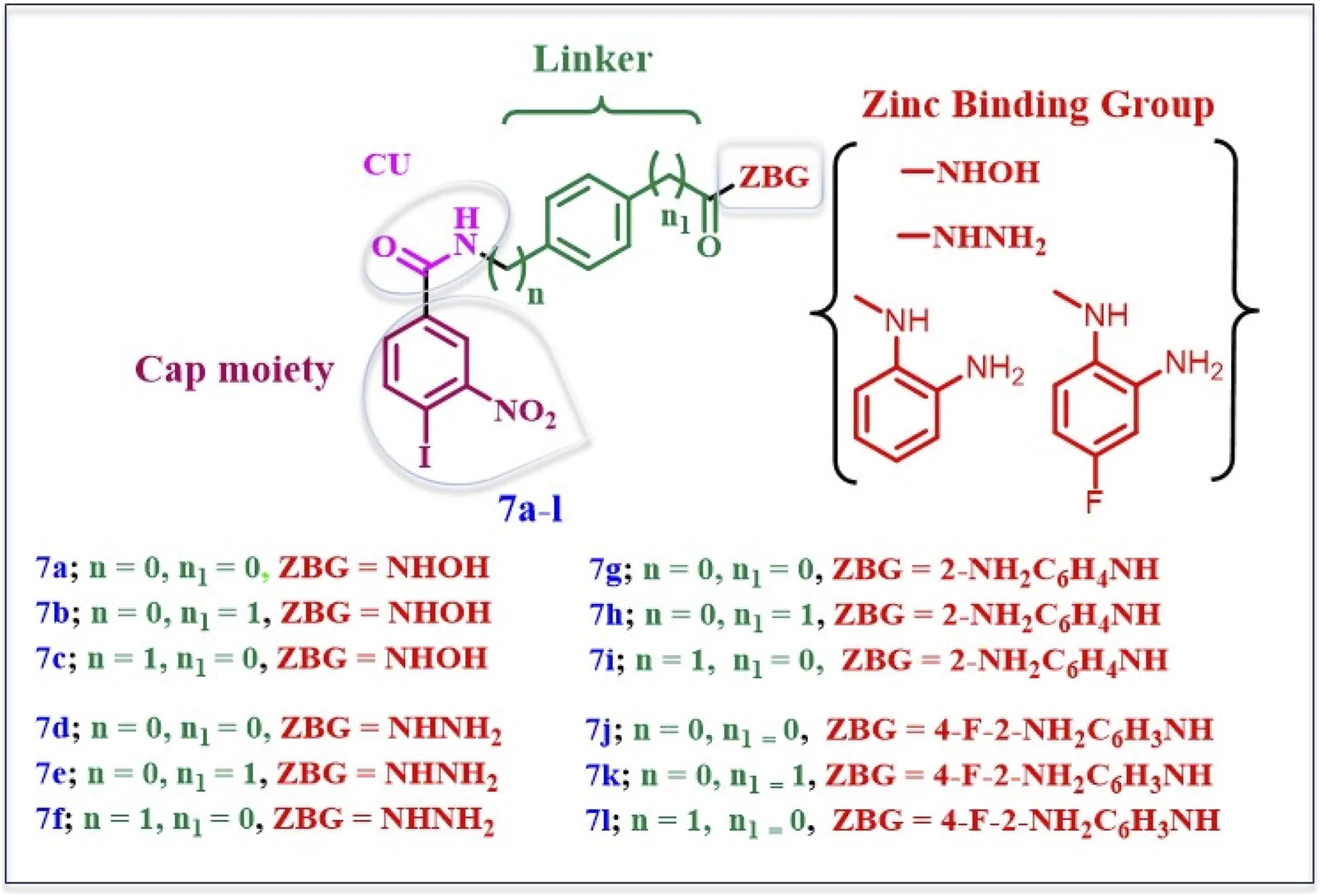
Structure of newly designed HDAC-2 inhibitors (7a–l).

Moreover, the chosen aromatic cores further enable geometric and distance tuning through systematic variation of the distance and the spatial trajectory between the 4-iodo-3-nitrophenyl cap and the terminal ZBGs. In this context, benzoic acid systems directly lock the ZBG into a rigid, fixed aromatic vector. In contrast, phenylacetic acid systems feature a single flexible methylene (–CH_2_–) spacer, providing a balanced compromise between structural rigidity and slight conformational adaptability, allowing the ZBG to slip into the catalytic cleft. In contrast, benzylamine systems structurally invert the connectivity of the central amide, optimizing the hydrogen-bonding donor and acceptor networks with key surface residues such as Asp104.

This design paradigm ultimately retains the cap and ZBG electronics essential for effective therapeutic activity. The electrophilic 4-iodo-3-nitrophenyl cap was intentionally retained due to its proven ability to cross-link with target proteins and generate pro-apoptotic reactive oxygen species (ROS) in hypoxic tumor microenvironments. This study presents a detailed structure–activity relationship (SAR) for rigidified multi-target inhibitors by evaluating this optimized, reactive cap across four chemically diverse ZBGs. The terminal groups comprise high-affinity pan-chelators, including hydroxylamine (hydroxamic acid) and hydrazide moieties, as well as isoform-selective class I binders, such as benzamide and fluorobenzamide groups, delineating a clear framework for future therapeutic enhancement.

## 2. Experimental

### 2.1. Chemistry

General details: See Appendix A (SI file).

The starting materials; 4-iodo-3-nitro benzoic acid (2) ^26,27^and amino esters (4a–c)^28,29^ were prepared according to literature methods.

#### 2.1.1. General procedure for the synthesis of ester derivatives (5a–c)

Carbonyl diimidazole (CDI) (0.65 g, 4.0 mmol) was added to a stirred solution of 4-iodo-3-nitrobenzoic acid (2) (1.17 g, 4.0 mmol) in anhydrous dichloromethane (15 mL). The mixture was stirred at room temperature for 1 h to enable activation. Subsequently, the appropriate amino ester hydrochloride salt derivative (4a–c) (4.0 mmol) and triethylamine (0.50 mL, 4.0 mmol) were added. The reaction mixture was allowed to stir at room temperature for an additional 24 h. Upon completion, the organic layer was washed sequentially with a 10% aqueous HCl solution and a 10% aqueous Na_2_CO_3_ solution. The organic phase was dried over anhydrous sodium sulfate (Na_2_SO_4_), filtered, and concentrated under reduced pressure to yield an orange oil. Trituration of the residue with cold diethyl ether afforded compound 4a–c as yellowish-orange crystals (62–74% yield).

##### 2.1.1.1. Ethyl 4-(4-iodo-3-nitrobenzamido)benzoate (5a)

Yield: 73%, mp: 210–212 °C, *R*_f_. 0.71 (Hexane : ethyl acetate, 2 : 1, v/v), IR (FTIR): *ν*_max_ (cm^−1^): 3416 (NH), 1703 ester (C=O), 1679 amidic (C=O), 1531, 1322 cm^−1^ (NO_2_ stretching), ^1^H NMR (400 MHz, DMSO-*d*_6_) *δ* (ppm) 10.77 (s, 1H, NHCO), 8.47 (d, *J* = 2.1 Hz, 1H, ArH), 8.29 (d, *J* = 8.2 Hz, 1H, ArH), 8.02–7.86 (m, 5H, ArH), 4.29 (q, *J* = 7.1 Hz, 2H, CH_2_), 1.31 (t, *J* = 7.1 Hz, 3H, CH_3_), ^13^C NMR (100 MHz, DMSO-*d*_6_): *δ* 165.78, 163.76, 153.66, 143.41, 142.11, 135.89, 132.86, 130.58, 125.57, 124.38, 120.26, 93.08, 61.03, 14.66. HRMS (ESI) *m*/*z* calculated for C_16_H_12_IN_2_O_5_ [M − H]^−^: 438.9791, found: 438.9000. Anal. calcd. for C_16_H_13_IN_2_O_5_: C, 43.66; H, 2.98; N, 6.36. Found: C, 43.82; H, 3.17; N, 6.60.

##### 2.1.1.2. Methyl [4-(4-iodo-3-nitrobenzamido)phenyl]acetate (5b)

Yield: 62%, mp: 158–159 °C, *R*_f_. 0.40 (Hexane : ethyl acetate, 2 : 1, v/v), IR (FTIR): *ν*_max_ (cm^−1^): 3301 (NH), 1734 ester (C=O), 1662 amidic (C=O), 1522, 1325 cm^−1^ (NO_2_ stretching), ^1^H NMR (400 MHz, DMSO-*d*_6_) *δ* 10.51 (s, 1H, NHCO), 8.45 (d, *J* = 2.1 Hz, 1H, ArH), 8.28 (d, *J* = 8.2 Hz, 1H, ArH), 7.93 (dd, *J* = 8.2, 2.1 Hz, 1H, ArH), 7.67 (d, *J* = 8.0 Hz, 2H, ArH), 7.25 (d, *J* = 8.0 Hz, 2H, ArH), 3.65 (s, 2H, CH_2_CO), 3.61 (s, 3H, OCH_3_), ^13^C NMR (100 MHz, DMSO-*d*_6_) *δ* 171.88, 162.96, 153.34, 141.74, 137.40, 135.93, 132.43, 130.35, 129.80, 123.93, 120.71, 92.25, 51.86, CH_2_ (overlapped in DMSO). HRMS (ESI) *m*/*z* calculated for C_16_H_12_IN_2_O_5_ [M − H]^−^: 438.9791, found: 438.9000. Anal. calcd. for C_16_H_13_IN_2_O_5_: C, 43.66; H, 2.98; N, 6.36. Found: C, 43.79; H, 3.17; N, 6.54.

##### 2.1.1.3. Methyl 4-[(4-iodo-3-nitrobenzamido)methyl]benzoate (5c)

Yield: 74%, mp: 158–160 °C, *R*_f_. 0.31 (Hexane : ethyl acetate, 2 : 1, v/v), IR (FTIR): *ν*_max_ (cm^−1^): 3271 (NH), 1711 ester (C=O), 1631 amidic (C=O), 1531, 1355 cm^−1^ (NO_2_ stretching), ^1^H NMR (400 MHz, DMSO-*d*_6_) *δ* 9.44 (t, *J* = 6.0 Hz, 1H, **NH**CH_2_), 8.41 (d, *J* = 2.0 Hz, 1H, ArH), 8.27 (d, *J* = 8.0 Hz, 1H, ArH), 7.93 (d, *J* = 8.2 Hz, 2H, ArH), 7.89 (dd, *J* = 8.0, 2.0 Hz, 1H, ArH), 7.47 (d, *J* = 8.0 Hz, 2H, ArH), 4.57 (d, *J* = 4.0 Hz, 2H, NH**CH**_2_), 3.84 (s, 3H, OCH_3_), ^13^C NMR (100 MHz, DMSO-*d*_6_) *δ* 166.58, 164.34, 153.67, 145.08, 142.15, 135.54, 132.42, 129.76, 128.75, 127.98, 123.99, 92.44, 52.55, 43.14. HRMS (ESI) *m*/*z* calculated for C_16_H_12_IN_2_O_5_ [M − H]^−^: 438.9791, found:438.9000. Anal. calcd. for C_16_H_13_IN_2_O_5_: C, 43.66; H, 2.98; N, 6.36. Found: C, 43.91; H, 3.19; N, 6.52.

#### 2.1.2. General procedure for the synthesis of carboxylic acid derivatives (6a–c)

To a 3 M methanolic KOH solution (3.4 g KOH) was added the appropriate derivative 5a–c (2 mmol). The mixture was stirred for 1–2 h until TLC analysis indicated complete consumption of the starting material. The solvent was evaporated under vacuum, and the aqueous layer was washed with ethyl acetate (2 × 20 mL). The aqueous phase was subsequently acidified with dilute HCl. The resulting precipitate was filtered, washed with water, and recrystallized from ethyl acetate: *n*-hexane to yield the desired products 6a–c (72–92%).

##### 2.1.2.1. 4-(4-Iodo-3-nitrobenzamido)benzoic acid (6a)

Yield: 88%, mp: 298 °C, *R*_f_. 0.58 (chloroform : methanol, 10 : 1, v/v). IR (FTIR): *ν*_max_ (cm^−1^): 3285 (NH), 3000–2543 carboxylic (OH), 1673 carboxylic (C=O), 1647 amidic (C=O), 1529, 1318 (NO_2_ stretching), ^1^H NMR (400 MHz, DMSO-*d*_6_) *δ* 12.80 (br s, 1H, COOH), 10.60 (s, 1H, NHCO), 8.55 (d, *J* = 2.3 Hz, 1H, ArH), 8.31 (dd, *J* = 8.8, 2.4 Hz, 1H, ArH), 7.96 (d, *J* = 8.9 Hz, 2H, ArH), 7.91 (d, *J* = 8.9 Hz, 2H, ArH), 7.54 (d, *J* = 8.8 Hz, 1H, ArH). ^13^C NMR (100 MHz, DMSO-*d*_6_) *δ* 166.95, 163.37, 154.54, 143.01, 138.71, 134.12, 130.27, 126.33, 125.74, 124.76, 119.74, 114.51. HRMS (ESI) *m*/*z* calculated for C_14_H_10_IN_2_O_5_ [M + H]^+^: 412.9634, found: 412.9000. Anal. calcd. for C_14_H_9_IN_2_O_5_: C, 40.80; H, 2.20; N, 6.80. Found: C, 41.07; H, 2.43; N, 7.04.

##### 2.1.2.2. [4-(4-Iodo-3-nitrobenzamido)phenyl]acetic acid (6b)

Yield: 92%, mp: 202–203 °C, *R*_f_. 0.50 (chloroform : methanol, 10 : 1, v/v). IR (FTIR): *ν*_max_ (cm^−1^): 3403 (NH), 3000–2543 carboxylic (OH), 1686 carboxylic (C=O), 1674 amidic (C=O), 1529, 1318 (NO_2_ stretching), ^1^H NMR (400 MHz, DMSO-*d*_6_) *δ* 10.53 (s, 1H, NHCO), 8.42 (d, *J* = 2.1 Hz, 1H, ArH), 8.26 (d, *J* = 8.3 Hz, 1H, ArH), 7.91 (dd, *J* = 8.3, 2.1 Hz, 1H, ArH), 7.64 (d, *J* = 8.6 Hz, 2H, ArH), 7.24 (d, *J* = 8.6 Hz, 2H, ArH), 3.53 (s, 2H, CH_2_CO). ^13^C NMR (100 MHz, DMSO-*d*_6_) *δ* 173.24, 163.39, 154.79, 139.17, 137.81, 134.36, 131.05, 130.02, 127.12, 124.97, 120.97, 114.73, 40.62. ^.^HRMS (ESI) *m*/*z* calculated for C_15_H_12_IN_2_O_5_ [M + NH_4_]^+^: 444.0056 found: 444.0000. Anal. calcd. for C_15_H_11_IN_2_O_5_: C, 42.28; H, 2.60; N, 6.57. Found: C, 42.47; H, 2.83; N, 6.81.

##### 2.1.2.3. 4-[(4-Iodo-3-nitrobenzamido)methyl]benzoic acid (6c)

Yield: 72% mp: 259–260 °C, *R*_f_. 0.56 (chloroform : methanol, 10 : 1, v/v). IR (FTIR): *ν*_max_ (cm^−1^): 3292 (NH), 3000–2543 carboxylic (OH), 1688 carboxylic (C=O), 1632 amidic (C=O), 1529, 1330 (NO_2_ stretching), ^1^H NMR (400 MHz, DMSO-*d*_6_) *δ* 12.85 (s, 1H, COOH), 9.43 (t, *J* = 6.0 Hz, 1H, **NH**CH_2_), 8.42 (d, *J* = 2.0 Hz, 1H, ArH), 8.27 (d, *J* = 8.2 Hz, 1H, ArH), 7.93–7.85 (m, 3H, ArH), 7.45 (d, *J* = 8.0 Hz, 2H, ArH), 4.57 (d, *J* = 5.9 Hz, 2H, NH**CH**_2_). ^13^C NMR (100 MHz, DMSO-*d*_6_) *δ* 167.64, 164.29, 153.65, 144.58, 142.13, 135.57, 132.42, 129.91, 127.83, 127.61, 124.02, 92.47, 43.17. HRMS (ESI) *m*/*z* calculated for C_15_H_12_IN_2_O_5_ [M + H]^+^: 426.9791, found: 427.0000 anal. calcd. for C_15_H_11_IN_2_O_5_: C, 42.28; H, 2.60; N, 6.57. Found: C, 42.14; H, 2.71; N, 6.83.

#### 2.1.3. General procedure for the synthesis of hydroxamic acid derivatives (7a–c)

A freshly prepared solution of hydroxylamine was obtained by adding KOH (8.4 g, 150 mmol) to a stirred solution of hydroxylamine hydrochloride (6.9 g, 100 mmol) in absolute methanol at 0 °C. After stirring for 30 min, the precipitated KCl was removed by filtration. A portion of the filtrate containing the freshly prepared NH_2_OH (10 mL) was added to the appropriate derivative 5a–c (2.3 mmol), and the mixture was stirred for 1 h. Upon completion, the reaction mixture was neutralized to pH 6–7 with dilute HCl. The resulting precipitate was collected by filtration and recrystallized from aqueous methanol to afford the pure product in 58–79% yield.

##### 2.1.3.1. *N*-[4-(Hydroxycarbamoyl)phenyl]-4-iodo-3-nitrobenzamide (7a)

Yield: 79%, mp: 244–246 °C, *R*_f_. 0.29 (chloroform : methanol, 10 : 1, v/v). IR (FTIR): *ν*_max_ (cm^−1^): 3316, 3254 (broad band, hydroxamate OH, NH), 1652, 1614 amidic (C=O), 1530, 1325 (NO_2_ stretching), ^1^H NMR (400 MHz, DMSO-*d*_6_) *δ* 11.15 (s, 1H, NHOH), 10.47 (s, 1H, NHCO), 8.98 (s, 1H, NHOH), 8.56 (d, *J* = 2.3 Hz, 1H, ArH), 8.14 (dd, *J* = 8.7, 2.3 Hz, 1H, ArH), 7.88–7.68 (m, 4H, ArH), 7.24 (d, *J* = 8.7 Hz, 1H, ArH). ^13^C NMR (100 MHz, DMSO*-d*_6_) *δ* 164.48, 163.96, 155.29, 142.00, 137.00, 134.84,128.19, 128.05, 125.65, 125.55, 120.18, 119.55. HRMS (ESI) *m*/*z* calculated for C_14_H_11_IN_3_O_5_ [M + H]^+^: 427.9743, found: 427.9000. Anal. calcd. for C_14_H_10_IN_3_O_5_: C, 39.37; H, 2.36; N, 9.84. Found: C, 39.58; H, 2.49; N, 10.12.

##### 2.1.3.2. *N*-{4-[2-(Hydroxyamino)-2-oxoethyl]phenyl}-4-iodo-3-nitrobenzamide (7b)

Yield: 58%, mp; 195–197 °C, *R*_f_. 0.29 (chloroform : methanol, 10 : 1, v/v). IR (FTIR): *ν*_max_ (cm^−1^): 3277 (broad band, hydroxamate, OH, NH), 1645 amidic (C=O), 1530, 1323 (NO_2_ stretching). The compound is present as two conformers in ratio (4 : 1), ^1^H NMR (400 MHz, DMSO-*d*_6_) *δ* 10.74 (br s, 1H, NHOH), 10.62 and 10.55 (2 s, 1H, NHCO), 10.28 and 10.04 (2 s, 1H, NHOH), 8.48 and 8.46 (2 d, *J* = 2.3 Hz, 1H, ArH), 8.28 and 6.86 (2 d, *J* = 8.0 and 8.8 Hz, 1H, ArH), 7.94 and 7.89 (2 dd, *J* = 8.8, 2.2 Hz, 1H, ArH), 7.65–7.63 (m, 2H, ArH), 7.26 and 7.22 (2 d, *J* = 8.8 Hz, 2H, ArH), 3.29–3.24 (m, 2H, CH_2_). ^13^C NMR (100 MHz, DMSO-*d*_6_) *δ* 167.31, 167.05, 164.52, 138.25, 136.32, 132.43, 130.49, 128.93, 127.37, 125.37, 120.13, 114.53, CH_2_ overlapped in DMSO. HRMS (ESI) *m*/*z* calculated for C_15_H_11_IN_3_O_5_ [M − H]^−^: 439.9743 Found: 439.8700. Anal. calcd. for C_15_H_12_IN_3_O_5_: C, 40.84; H, 2.74; N, 9.52. Found: C, 41.09; H, 2.86; N, 9.79.

##### 2.1.3.3. *N*-{[4-(Hydroxycarbamoyl)phenyl]methyl}-4-iodo-3-nitrobenzamide (7c)

Yield: 70%, mp: 180–182 °C, *R*_f_. 0.26 (chloroform : methanol, 10 : 1, v/v), IR (FTIR): *ν*_max_ (cm^−1^): 3294, 3173 (broad band, hydroxamate OH, NH), 1639, 1621 amidic (C=O), 1539, 1313 (NO_2_ stretching). The compound is present as two conformers in ratio (9 : 1), ^1^H NMR (400 MHz, DMSO-*d*_6_) *δ* 11.17 and 10.48 (2 s, 1H, NHOH), 9.40 and 9.16 (2 t, *J* = 6.0 Hz, 1H, NHCO), 9.00 (br s, 1H, NHOH), 8.47 and 8.40 (2 d, *J* = 2.3 Hz, 1H, ArH), 8.26 and 7.19 (2 d, *J* = 8.8 Hz, 1H, ArH), 7.87 and 8.06 (2 dd, *J* = 8.8, 2.3 Hz, 1H, ArH), 7.70 (d, *J* = 8.3 Hz, 2H, ArH), 7.40–7.35 (m, 2H, ArH), 4.52–4.47 (m, 2H, NHC**H**_2_). ^13^C NMR (100 MHz, DMSO-*d*_6_) *δ* 164.62, 164.24, 154.99, 153.67, 148.35, 144.72, 143.19, 142.12, 136.93, 135.61, 135.08, 134.42, 132.34, 131.80, 127.70, 127.63, 127.41, 125.30, 125.09, 124.00, 119.52, 92.46, 43.12 42.97. HRMS (ESI) *m*/*z* calculated for C_15_H_11_IN_3_O_5_ [M − H]^−^: 439.9743, found: 439.8500. Anal. calcd. for C_15_H_12_IN_3_O_5_: C, 40.84; H, 2.74; N, 9.52. Found: C, 41.09; H, 2.95; N, 9.71.

#### 2.1.4. General procedure for the synthesis of hydrazide derivatives (7d–f)

To a stirred solution of 1,1′-carbonyldiimidazole (0.45 g, 2.8 mmol) in anhydrous dichloromethane and acetonitrile as cosolvents (20 mL) was added the appropriate carboxylic acid derivative 6a–c (2.5 mmol). The mixture was stirred at room temperature for 3 h to form the acyl imidazole intermediate. Hydrazine hydrate (99%, 0.24 mL, 5 mmol) was then added, and the reaction mixture was stirred for an additional 48 h. Upon completion, the organic phase was washed with a dilute aqueous Na_2_CO_3_ solution (2 × 20 mL), dried over anhydrous [Na_2_SO_4_/MgSO_4_], and concentrated under reduced pressure. The resulting crude products were purified by recrystallization from aqueous methanol to afford compounds 7d–f in 58–75% yield.

##### 2.1.4.1. *N*-(4-(Hydrazinecarbonyl)phenyl)-4-iodo-3-nitrobenzamide (7d)

Yield: 65%, mp > 250 °C, *R*_*f*_. 0.38 (chloroform : methanol, 10 : 1, v/v). IR (FTIR): *ν*_max_ (cm^−1^): 3305 (NH, broad), 1655, 1599 amidic (C=O), 1528, 1324 (NO_2_ stretching). The compound is present as two conformers in ratio (3 : 2), ^1^H NMR (400 MHz, DMSO-*d*_6_) *δ* 10.71 and 10.54 (2 s, 1H, CONH), 9.71 and 9.69 (2 s, 1H, CON**H**NH_2_), 8.54 and 8.50 (2 d, *J* = 2.3 Hz, 1H, ArH), 8.37–8.27 (m, 1H, ArH), 8.01–7.80 (m, 4H, ArH), 7.54 and 7.50 (2 d, *J* = 8.9 Hz, 1H, ArH), 4.51 (br s, 2H, NHN**H**_2_). ^13^C NMR (101 MHz, DMSO-*d*_6_) *δ* 165.97, 165.92, 163.69, 163.58, 154.93, 153.74, 142.09, 141.84, 141.53, 139.20, 136.11, 134.48, 132.81, 129.21, 128.91, 128.19, 128.14, 126.92, 125.96, 125.11, 124.34, 120.18, 120.11, 114.81. HRMS (ESI) *m*/*z* calculated for C_14_H_12_IN_4_O_4_ [M + H]^+^: 426.9903 found: 427.0000. Anal. calcd. for C_14_H_11_IN_4_O_4_: C, 39.46; H, 2.60; N, 13.15. Found: C, 39.78; H, 2.76; N, 13.41.

##### 2.1.4.2. *N*-(4-(2-Hydrazineyl-2-oxoethyl)phenyl)-4-iodo-3-nitrobenzamide (7e)

Yield: 58%, mp; 177–178 °C, *R*_f_. 0.38 (chloroform : methanol, 10 : 1, v/v). The compound is present as two conformers in (3 : 2) ratio. ^1^H NMR (500 MHz, DMSO-*d*_6_) *δ* 10.47 and 10.30 (2 s, 1H, CONH), 9.17 (s, 1H, CO**NH**NH_2_), 8.49 and 8.45 (2 s, 1H, ArH), 8.26 (br d, *J* = 8.7 Hz, 1H), 7.93 and 7.48 (2 d, *J* = 8.3 Hz, 1H), 7.63 (br d, *J* = 8.3 Hz, 2H, ArH), 7.22 (d, *J* = 8.3 Hz, 2H, ArH), 4.35 (br s, 2H, CONH**NH**_2_), 3.98 (s, 2H, CH_2_CO).^13^C NMR (101 MHz, DMSO-*d*_6_) *δ* 170.13, 170.08, 163.26, 163.12, 154.77, 153.69, 142.03, 139.12, 137.68, 137.40, 136.26, 134.39, 132.76, 132.61, 132.28, 130.08, 129.67, 129.60, 127.09, 125.02, 124.29, 120.89, 114.73, 92.76, 40.59 (obscured in solvent signals). HRMS (ESI) *m*/*z* calculated for C_15_H_14_IN_4_O_4_ [M + H]^+^: 441.0060 found: 441.0000, anal. calcd. for C_15_H_13_IN_4_O_4_: C, 40.93; H, 2.98; N, 12.73. Found: C, 41.22; H, 2.87; N, 13.01.

##### 2.1.4.3. *N*-(4-(Hydrazinecarbonyl)benzyl)-4-iodo-3-nitrobenzamide (7f)

Yield; 75%, mp; 165–166 °C, *R*_f_. 0.47 (chloroform : methanol, 10 : 1, v/v). IR (FTIR): *ν*_max_ (cm^−1^): 3276 (broad, NH), 1635, 1620 amidic (C=O), 1522, 1354 (NO_2_ stretching). The compound is present as two conformers in (4 : 1) ratio, ^1^H NMR (500 MHz, DMSO-*d*_6_) *δ* 9.70 (s, 1H, CO**NH**NH_2_), 9.38 and 9.21 (2 br s, 1H, **NH**CH_2_), 8.43–8.38 (m, 1H, ArH), 7.86 and 7.45 (2 d, *J* = 8.9 Hz, 1H, ArH), 7.75 (br d, *J* = 8.0 Hz, 2H, ArH), 7.35 (br d, *J* = 8.0 Hz, 2H, ArH), 4.49 (br d, *J* = 5.8 Hz, 2H, NH**CH**_2_), 3.96 (s, 2H, CONH**NH**_2_). ^13^C NMR (100 MHz, DMSO-*d*_6_) *δ* 166.23, 164.41, 164.26, 154.66, 153.67, 142.97, 142.61, 142.11, 139.14, 135.62, 133.93, 132.43, 132.36, 129.64, 127.63, 127.58, 127.50, 126.56, 124.69, 124.01, 114.76, 92.45, 43.11, 43.01. HRMS (ESI) *m*/*z* calculated for C_15_H_14_IN_4_O_4_ [M + H]^+^: 441.0060 found: 440.9000, anal. calcd. for C_15_H_13_IN_4_O_4_: C, 40.93; H, 2.98; N, 12.73. Found: C, 41.20; H, 3.17; N, 13.02.

#### 2.1.5. General procedure for the synthesis of benzanilide derivatives (7g–i)

To a stirred solution of 1,1′-carbonyldiimidazole (0.45 g, 2.8 mmol) in anhydrous dichloromethane and acetonitrile as cosolvents (20 mL) was added the appropriate carboxylic acid derivative 6a–c (2.5 mmol). The mixture was stirred at room temperature for 3 h. Subsequently, benzene-1,2-diamine (0.27 g, 2.5 mmol) was added, and the reaction mixture was stirred for 48–96 h. Upon completion, the organic layer was washed with a dilute aqueous NaHCO_3_ solution (2 × 20 mL), dried over anhydrous [Na_2_SO_4_/MgSO_4_], and concentrated under reduced pressure. The crude products were purified to afford compounds 7g–i in 30–84% yield. Derivatives 7h and 7i were isolated *via* recrystallization from aqueous methanol. Derivative 7g was purified by column chromatography using chloroform/methanol (10 : 0.5 v/v) as the eluent.

##### 2.1.5.1. *N*-(4-((2-Aminophenyl)carbamoyl)phenyl)-4-iodo-3-nitrobenzamide (7g)

Yield: 47%, mp: 197–199 °C, *R*_f_. 0.67 (chloroform : methanol, 10 : 1, v/v). IR (FTIR): *ν*_max_ (cm^−1^): 3412, 3296 (NH_2_, NH), 1682, 1599 amidic (C=O), 1530, 1324 (NO_2_ stretching). The compound is present in two conformers with (4 : 1) ratio, ^1^H NMR (500 MHz, DMSO-*d*_6_) *δ* 10.71 and 10.69 (2 s, 1H, NHCO), 10.00 and 9.59 (2 s, 1H, anilide NH), 8.46 and 8.44 (2 d, *J* = 2.0 Hz, 1H, ArH), 8.28–8.24 (m, 1H, ArH), 7.99–7.83 (m, 5H, ArH), 7.62–7.61 and 7.11 (m, d, *J* = 7.9 Hz, 1H, anilide ArH), 7.61–7.59 and 6.92 (m and t, *J* = 7.6 Hz, 1H, anilide ArH), 7.51–7.47 and 6.74 (m and d, *J* = 7.9 Hz, 1H, anilide ArH), 7.26–7.24 and 6.55 (m and t, *J* = 7.3 Hz, 1H, anilide ArH), 4.86 (br s, 2H, NH_2_). ^13^C NMR (101 MHz, DMSO-*d*_6_) *δ* 165.18, 163.72, 163.62, 154.97, 153.73, 143.65, 142.18, 142.10, 141.86, 139.15, 136.03, 134.57, 132.87, 130.40, 130.09, 129.12, 129.07, 128.92, 127.19, 126.93, 125.20, 124.41, 123.90, 123.86, 120.26, 120.06, 119.99, 116.75, 116.61, 114.82, 93.14. HRMS (ESI) *m*/*z* calculated for C_20_H_14_IN_4_O_4_ [M − H]^−^: 501.0060 found: 500.8000. Anal. calcd. for C_20_H_15_IN_4_O_4_: C, 47.83; H, 3.01; N, 11.15. Found: C, 48.11; H, 3.23; N, 11.42.

##### 2.1.5.2. *N*-(4-(2-((2-Aminophenyl)amino)-2-oxoethyl)phenyl)-4-iodo-3-nitrobenzamide (7h)

Yield: 84%, mp: 150–151 °C, *R*_f_. 0.57 (chloroform : methanol, 10 : 1, v/v). IR (FTIR): *ν*_max_ (cm^−1^): 3253 (broad, NH), 1652, 1601 amidic (C=O), 1530, 1325 (NO_2_ stretching). The compound is present as 2 conformers in (7 : 3) ratio, ^1^H NMR (400 MHz, DMSO-*d*_6_) *δ* 10.53 (s, 1H, CONH), 9.52 and 9.40 (2 s, 1H, anilide NH), 8.50 and 8.46 (2 d, *J* = 2.1 Hz, 1H, ArH), 8.32 and 8.28 (2 d, *J* = 8.2 Hz, 1H, ArH), 7.98 and 7.93 (2 dd, *J* = 8.2, 2.1 Hz, 1H, ArH), 7.72 (br d, *J* = 8.3 Hz, 2H, ArH), 7.37 and 7.32 (2 d, *J* = 8.3 Hz, 2H, ArH),7.17 (br d, *J* = 7.5 Hz, 1H, anilide ArH), 7.63–7.53 and 6.92 (m, t, *J* = 7.5, 1H, benzamide Ar H), 7.52–7.46 and 6.73 (m, d, *J* = 7.5, 1H, anilide ArH), 7.29–7.22 and 6.55 (m, t, *J* = 7.5, 1H, anilide ArH), 4.86 (s, 2H, NH_2_), 3.73–3.55 (m, 2H, CH_2_CO). ^13^C NMR (100 MHz, DMSO-*d*_6_) *δ* 170.00, 169.65, 163.17, 163.10, 153.71, 153.61, 142.45, 142.03, 142.00, 137.71, 137.49, 136.31, 136.21, 132.78, 132.73, 132.68, 131.92, 131.13, 129.99, 129.86, 126.40, 125.82, 125.63, 125.47, 124.31, 123.76, 120.96, 116.65, 116.33, 92.79, 92.75, 42.86, 42.63. HRMS (ESI) *m*/*z* calculated for C_21_H_18_IN_4_O_4_ [M + H]^+^: 517.0373 found: 517.0000. Anal. calcd. for C_21_H_17_IN_4_O_4_: C, 48.85; H, 3.32; N, 10.85. Found: C, 49.17; H, 3.46; N, 11.08.

##### 2.1.5.3. *N*-(4-((2-Aminophenyl)carbamoyl)benzyl)-4-iodo-3-nitrobenzamide) (7i)

Yield: 30% mp; 149–150 °C, *R*_f_. 0.57 (chloroform : methanol, 10 : 1, v/v). The compound is present as 2 conformers in (4 : 1) ratio, ^1^H NMR (500 MHz, DMSO-*d*_6_) *δ* 9.60 (br s, 1H, benzanilide NH), 9.25 (br t, *J* = 6.1 Hz, 1H, **NH**CH_2_), 8.44–8.41 (m, 1H, ArH), 8.22–8.16 (m, 1H, ArH), 7.92 and 7.88 (2 d, *J* = 8.2 Hz, 2H, ArH), 7.46 (d, *J* = 8.4 Hz, 1H), 7.42 (d, *J* = 8.2 Hz, 2H, ArH), 7.63–7.60 and 7.14 (m, d, *J* = 7.9 Hz, 1H, anilide ArH), 6.93 (d, *J* = 7.4 Hz, 1H, anilide ArH), 7.27–7.23 and 6.75 (m and d, *J* = 7.9 Hz, 1H, anilide ArH), 6.60–6.52 (m, 1H, anilide ArH), 4.86 (s, 2H, NH_2_), 4.53 (d, *J* = 6.0 Hz, 2H, NHC**H**_2_). ^13^C NMR (126 MHz, DMSO-*d*_6_) *δ* 165.69, 164.50, 154.76, 143.65, 143.46, 139.26, 134.00, 133.79, 128.42, 127.63, 127.21, 127.02, 126.71, 124.77, 123.91, 116.84, 116.70, 114.87, 43.13. HRMS (ESI) *m*/*z* calculated for C_21_H_18_IN_4_O_4_ [M + H]^+^: 517.0373 found: 516.9000. Anal. calcd. for C_21_H_17_IN_4_O_4_: C, 48.85; H, 3.32; N, 10.85. Found: C, 49.18; H, 3.44; N, 11.09.

#### 2.1.6. General procedure for the synthesis of fluoro-benzanilide derivatives (7j–l)

To a stirred solution of carbonyl diimidazole (0.45 g, 2.8 mmol) in anhydrous dichloromethane and acetonitrile as cosolvent (20 mL) was added the appropriate acid derivative 6a–c (2.5 mmol) and the mixture was left to stir for 3 h. Then, 4-fluorobenzene-1,2-diamine (0.32 g, 2.5 mmol) was added, and the mixture was left stirring for 96 h. After completion of the reaction, the organic layer was collected, washed with diluted solution of NaHCO_3_ (2 × 20 mL) to obtain compounds 7j–l in 31–60% yield. The obtained products purified by recrystallization from aq. methanol (7k) or column chromatography using chloroform : methanol (10 : 0.5) as an eluting system (7j and 7l).

##### 2.1.6.1. *N*-(4-((2-amino-4-fluorophenyl)carbamoyl)phenyl)-4-iodo-3-nitrobenzamide (7j)

Yield; 45%, mp: 131–133 °C, *R*_f_. 0.66 (chloroform : methanol, 10 : 1, v/v). ^1^H NMR (500 MHz, DMSO-*d*_6_) *δ* 10.68 (s, 1H, NHCO), 9.51 (s, 1H, anilide NH), 8.45 (d, *J* = 1.8 Hz, 1H, ArH), 8.27 (d, *J* = 8.3 Hz, 1H, ArH), 7.97–7.91 (m, 3H, ArH), 7.83 (d, *J* = 8.3 Hz, 2H, ArH), 7.05 (t, *J* = 7.6 Hz, 1H, *p*-F-anilide ArH), 6.48 (dd, *J* = 11.3, 3.1 Hz, 1H, *p*-F-anilide ArH), 6.31 (dt, *J* = 8.4, 3.9 Hz, 1H, *p*-F-anilide ArH), 5.18 (s, 2H, NH_2_). ^13^C NMR (125 MHz, DMSO-*d*_6_) *δ* 165.72, 163.91, 155.03, 142.18, 139.09, 134.58, 132.85, 129.19, 129.04, 125.15, 124.32, 120.19, 120.12, 114.80, 102.87, 102.65, 102.25, 101.93. HRMS (ESI) *m*/*z* calculated for C_20_H_15_FIN_4_O_4_ [M + H]^+^: 521.0122 found: 521.0000. Anal. calcd. for C_20_H_14_FIN_4_O_4_: C, 46.17; H, 2.71; N, 10.77. Found: C, 46.40; H, 2.85; N, 10.98.

##### 2.1.6.2. *N*-(4-(2-((2-amino-4-fluorophenyl)amino)-2-oxoethyl)phenyl)-4-iodo-3-nitrobenzamide (7k)

Yield: 60%, mp: 208–210 °C, *R*_f_. 0.60 (chloroform : methanol, 10 : 1, v/v). IR (FTIR): *ν*_max_ (cm^−1^): 3262 (NH, broad), 1644 amidic (C=O), 1530, 1325 (NO_2_ stretching). The compound is present in 2 conformers in 9 : 1 ratio, ^1^H NMR (500 MHz, DMSO-*d*_6_) *δ* 10.45 and 10.29 (2 s, 1H, NHCO), 9.33 and 9.25 (2 s, 1H, anilide NH), 8.46–8.37 (m, 1H, ArH), 8.25 (br d, *J* = 8.2 Hz, 1H, ArH), 7.95–7.87 (m, 1H, ArH), 7.65 (br d, *J* = 8.1 Hz, 2H, ArH), 7.50–7.42 and 7.04 (m, t, *J* = 5.9 Hz, 1H, *p*-F-anilide ArH) 7.29 (br d, *J* = 8.1 Hz, 2H, ArH), 7.17 and 6.44 (d, *J* = 10.7 Hz, dd, *J* = 11.3, 2.9 Hz, 1H, *p*-F-anilide ArH), 6.75–6.58 and 6.25 (m, td, *J* = 8.7, 2.8 Hz, 1H, *p*-F-anilide ArH), 5.12 (br s, 2H, NH_2_), 3.61 and 3.57 (2 br s, 2H, CH_2_CO). ^13^C NMR (125 MHz, DMSO-*d*_6_) *δ* 169.98, 163.23, 162.06, 160.17, 153.76, 144.91, 144.82, 142.09, 137.53, 136.36, 132.82, 132.67, 129.93, 127.75, 127.66, 124.34, 121.01, 119.82, 102.57, 102.39, 101.96, 101.76, 92.76, 42.55. HRMS (ESI) *m*/*z* calculated for C_21_H_17_FIN_4_O_4_ [M + H]^+^: 535.0279 found: 535.0000. Anal. calcd. for C_21_H_16_FIN_4_O_4_: C, 47.21; H, 3.02; N, 10.49. Found: C, 47.42; H, 3.19; N, 10.63.

##### 2.1.6.3. *N*-(4-((2-amino-4-fluorophenyl)carbamoyl)benzyl)-4-iodo-3-nitrobenzamide (7l)

Yield: 31%, mp: 190–192 °C, *R*_f_. 0.69 (chloroform : methanol, 10 : 1, v/v). ^1^H NMR (500 MHz, DMSO-*d*_6_) *δ* 9.52 (s, 1H, anilide NH), 9.24 (t, *J* = 6.5 Hz, 1H, **NH**CH_2_), 8.40 (d, *J* = 2.3 Hz, 1H, ArH), 8.16 (dd, *J* = 8.9, 2.3 Hz, 1H, ArH), 7.88 (d, *J* = 8.3 Hz, 2H, ArH), 7.43 (d, *J* = 8.9 Hz, 1H, ArH), 7.38 (d, *J* = 8.3 Hz, 2H, ArH), 7.04 (dd, *J* = 8.7, 6.3 Hz, 1H, *p*-F-anilide ArH), 6.48 (dd, *J* = 11.2, 2.9 Hz, 1H, *p*-F-anilide ArH), 6.30 (td, *J* = 8.5, 2.9 Hz, 1H, *p*-F-anilide ArH), 5.17 (s, 2H, NH_2_), 4.49 (d, *J* = 6.0 Hz, 2H, NH**CH**_2_). ^13^C NMR (126 MHz, DMSO-*d*_6_) *δ* 165.92, 164.44, 154.73, 145.96, 143.46, 139.17, 133.99, 133.60, 128.42, 127.55, 126.59, 124.76, 119.75, 114.84, 102.63, 102.45, 102.04, 101.84, 43.07. HRMS (ESI) *m*/*z* calculated for C_21_H_17_FIN_4_O_4_ [M + H]^+^: 535.0279 found: 535.0000. Anal. calcd. for C_21_H_16_FIN_4_O_4_: C, 47.21; H, 3.02; N, 10.49. Found: C, 47.43; H, 3.21; N, 10.75.

### 2.2. Biology

#### 2.2.1. Cell viability assay

To evaluate the safety profile of the synthesized compounds, the effect of derivatives 7a–l on cell viability was examined using the non-tumorigenic MCF-10 A human breast epithelial cell line. Cell viability was measured *via* the standard MTT assay following a 4 day incubation period.^30,31^ Detailed experimental procedures are provided in Appendix A (SI file).

#### 2.2.2. *In vitro* growth inhibitory activity against human cancer cell lines

The growth inhibitory activity of compounds 7a, 7c, and 7d was evaluated against three human cancer cell lines: MDA-MB-231 (triple-negative breast cancer), A-549 (non-small cell lung cancer), and SEM (B-cell acute lymphoblastic leukemia). All cell lines (either normal cell or cancer cell) were purchased from the American Type Culture Collection (ATCC, USA) *via* Holding company for biological products and vaccines (VACSERA), Cairo, Egypt. Cell viability and growth inhibition were quantified using the propidium iodide (PI) fluorescence assay,^32^ and IC_50_ values were determined from dose-response curves. Data were derived from at least two independent experiments, each performed in triplicate per concentration. Detailed experimental protocols are available in Appendix A (SI).

#### 2.2.3. HDAC inhibition assays

To assess the HDAC inhibitory properties of the final compounds, enzymatic assays were performed against various isoforms according to previously described methodologies.^16,33^ Compounds 7a–l were screened at a concentration of 10 µM against HDAC-1, -2, -3, and -8, using DMSO as the vehicle. Vorinostat (SAHA, 0.5 µM) served as the reference control. All screening assays were performed in triplicate across three independent experiments, and the data are reported as the mean values. Based on these initial screening results, the selectivity profile of the most effective compounds against specific HDAC isoforms (HDAC-1 and HDAC-2) was evaluated. Additionally, IC_50_ values for the most potent derivatives were determined, with vorinostat utilized as the positive drug control. Comprehensive experimental details are available in sppendix A (SI).

#### 2.2.4. Western blotting analysis

To evaluate the molecular effects of compound 7a, western blot analysis was performed.^34,35^ SEM cells were treated with compound 7a using beta actin as a loading control. Following treatment, cells were lysed using RIPA buffer supplemented with protease and phosphatase inhibitor cocktails. Total protein content was quantified using the BCA or Bradford protein assay kit. Refer to Appendix A (SI) for more details.

#### 2.2.5. Cell cycle analysis and apoptosis

To investigate the mechanisms underlying the anti-leukemic effects of compound 7a, its impact on cell cycle progression and apoptosis induction was evaluated in SEM cells. Cells were treated with compound 7a at its IC_50_ concentration for 24 hours. Subsequently, the cells were stained with Annexin V-FITC and propidium iodide (PI), and apoptotic populations alongside cell cycle distribution were quantified *via* flow cytometry^36^ using a BD FACSCalibur system. Detailed experimental protocols are provided in Appendix A (SI).

### 2.3. Molecular docking study

Molecular docking simulation for compound 7a was conducted within the active site of HDAC-2 using AutoDock Vina software,^37^ following previously reported protocols. The crystal structure of HDAC-2 was retrieved from the Protein Data Bank (PDB ID: 4LXZ). Comprehensive details regarding the docking parameters, scoring functions, and ligand preparation are provided in Appendix A (SI).

### 2.4. SwissADME predictions

*In silico* pharmacokinetic properties and drug-likeness profiles for compound 7a were predicted using the SwissADME web server^38^ developed by the Swiss Institute of Bioinformatics. Detailed methodology and the calculated physicochemical, ADME, and medicinal chemistry parameters are provided in Appendix A (SI).

## 3. Results and discussion

### 3.1. Chemistry

Scheme 1 illustrates the synthetic pathway of the new key intermediates 5a–c. The starting materials; 4-iodo-3-nitro benzoic acid (2) ^26,27^and amino esters (4a–c) ^28,29^were prepared according to literature methods. Their physical and spectral data were consistent with the reported data. The key intermediates 5a–c were prepared by coupling the protected hydrochloride salt of amino ester (*p*-amino benzoic acid, *p*-amino phenyl acetic acid, and *p*-amino methyl benzoic acid) (4a–c) and compound 2*via* CDI in the presence of Et_3_N and anhydrous dichloromethane. Upon completion of the reaction monitored by TLC, the organic layer was washed with dilute HCl and 10% Na_2_CO_3_. The organic layer was dried over anhydrous sodium sulphate, evaporated, and the resultant residue was washed with cold ether, yielding pure compounds 5a–c in 62–74% yields.

**Scheme 1:**
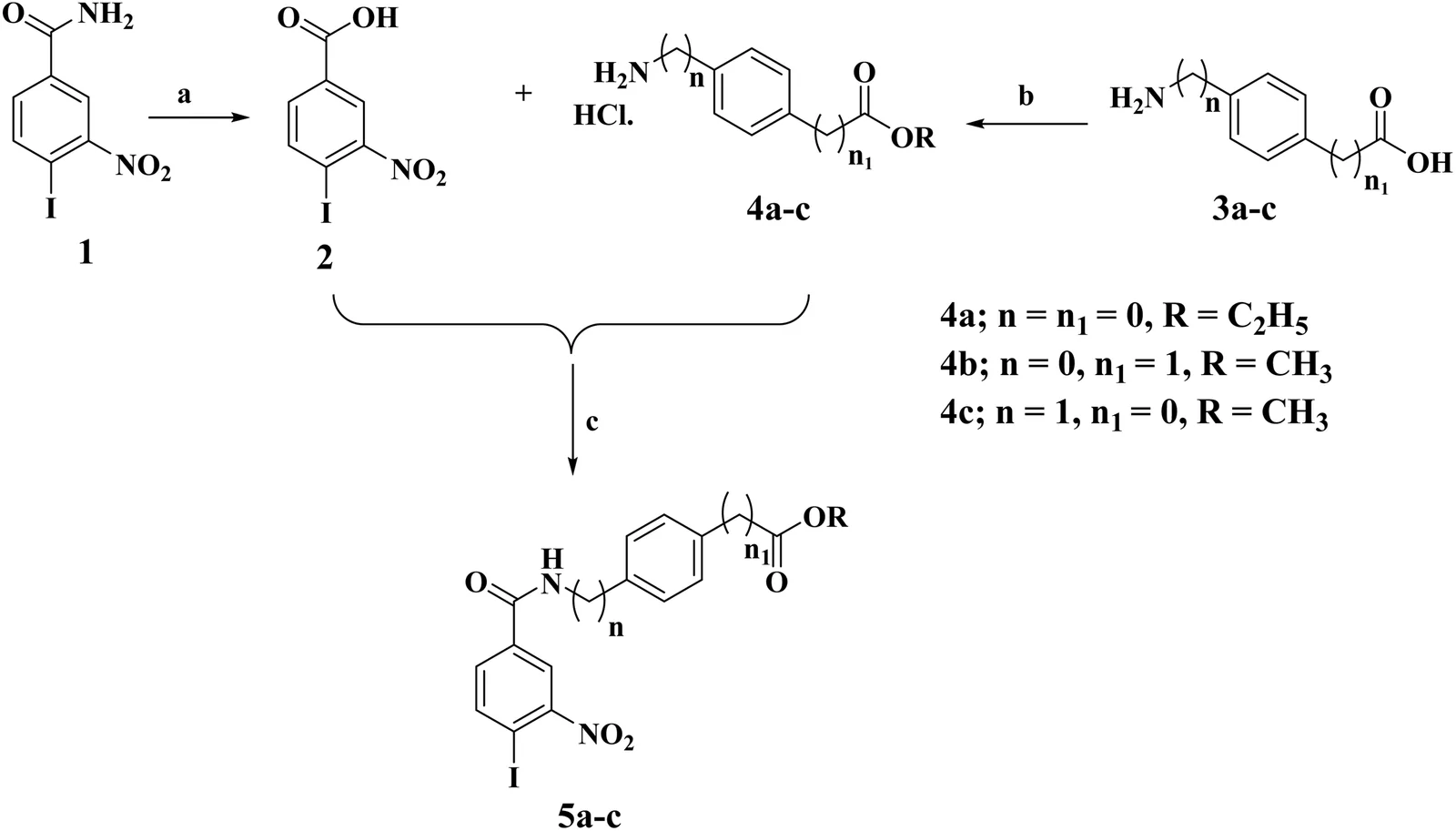
Synthesis of the key intermediates 5a–c. Reagents and reaction conditions: ^*a*^NaOH, H_2_O, heating; ^*b*^acetyl chloride, absolute methanol/ethanol, reflux, 8 h; 85%; ^*c*^CDI, Et_3_N, DCM, r.t, overnight; 62–74% yields.

The structures of compounds 5a–c were confirmed by NMR and representative IR analyses. The IR spectrum of 5a, as an example, revealed a characteristic peak at 3416 cm^−1^ corresponding to the amidic NH stretching vibration. Furthermore, two intense sharp peaks at 1703 and 1679 cm^−1^ correspond to ester and amidic carbonyl stretching vibrations, respectively, in addition to two stretching bands at 1531 and 1322 cm^−1^ corresponding to the NO_2_ group. The ^1^H NMR spectrum (400 MHz, DMSO-*d*_6_) of compound 5a is characterized by the presence of a broad singlet signal corresponding to the amidic NH proton at *δ* 10.77, a quartet and a triplet signal corresponding to the ethyl ester protons OCH_2_CH_3_ at *δ* 4.29 and 1.31 ppm, respectively.

Additionally, the aromatic region was confirmed by the appearance of two doublet signals at *δ* 8.47 and 8.29 ppm corresponding to two aromatic protons, and a multiplet signal at *δ* 8.02–7.86 ppm assigned to 5 aromatic protons. The ^13^C NMR spectra of 5a–c indicated the presence of ester and amidic carbonyl characteristic signals in the range of *δ* 171.88–165.78 ppm and 164.34–162.96 ppm. Additionally, ^13^C NMR spectra of 5a–c showed additional signals corresponding to ethyl and methyl ester carbons. For example, the ^13^C NMR spectrum of compound 5a revealed two signals at *δ* 61.03 and 14.66 ppm. ESI-HRMS (ESI) of compound 5a, as an example, revealed [M − H]^−^ peak at 438.9000.

Scheme 2 demonstrates the synthetic route of compounds 6a–c to 7a–l. The targeted compounds 7a–c were prepared by treating the corresponding ester with hydroxylamine hydrochloride in the presence of KOH. The resulting hydroxamate potassium salt was neutralised with dilute HCl to precipitate the required hydroxamic acid derivatives 7a–c, which were then recrystallized from aqueous methanol, yielding 58–79%.

**Scheme 2:**
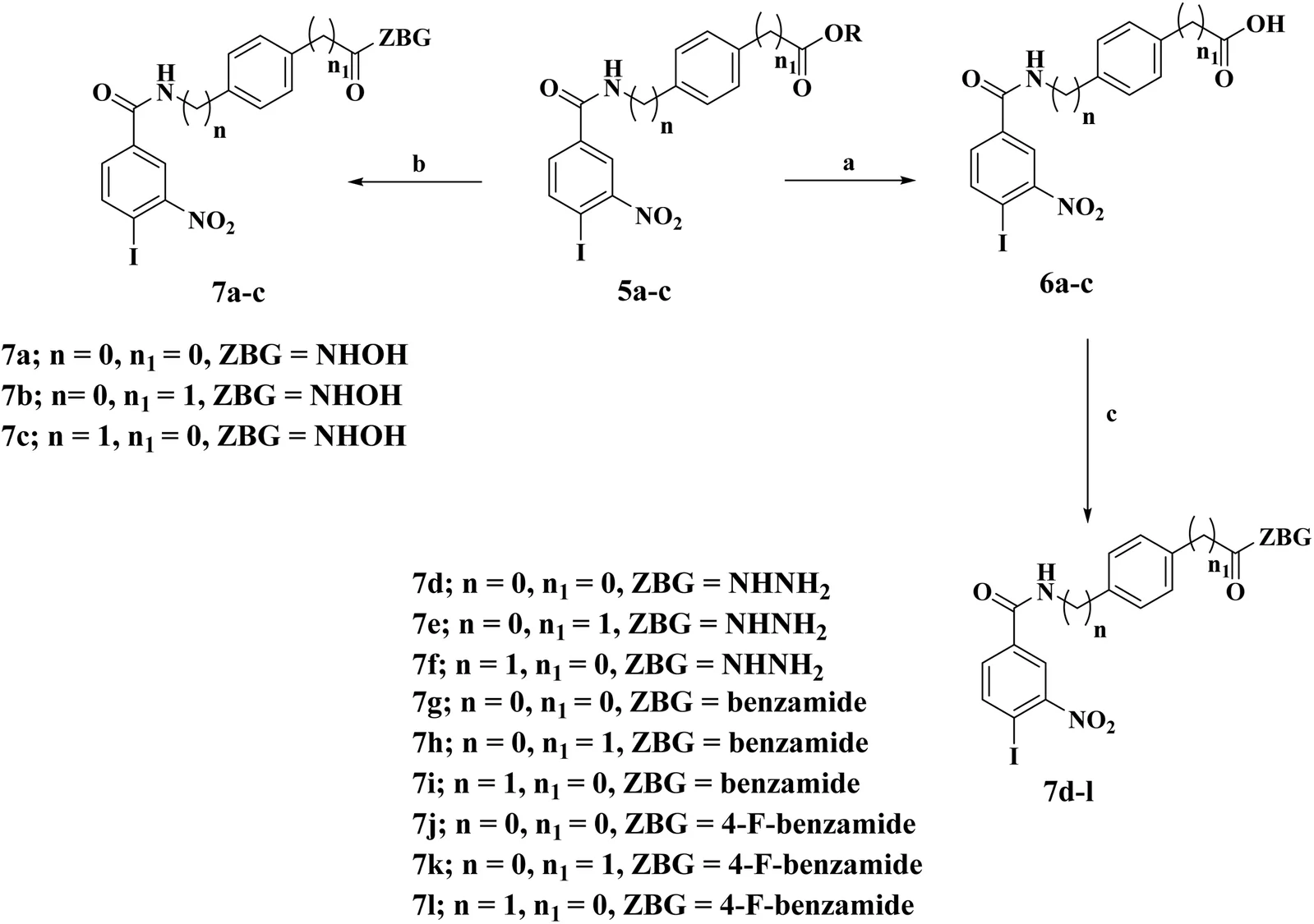
Synthesis of key intermediates 6a–c and targeted compounds 7a–l. Reagents and reaction conditions: ^*a*^3 M alc. KOH, methanol, r.t., 2 h, 72–92%; ^*b*^NH_2_OH·HCl, KOH, 0 °C, absolute methanol, 1 h, 58–79%; ^*c*^CDI, NH_2_NH_2_·H_2_O, DCM/acetonitrile, r.t., 48 h, 58–75%/CDI, benzene-1,2-diamine, DCM, acetonitrile, r.t., 96 h, 30–84%/CDI, 4-floro-benzene-1,2-diamine, DCM, acetonitrile, r.t., 96 h, 31–60%.

The ^1^H NMR spectra of hydroxamic acid derivatives 7a–c exhibited the absence of ethyl or methyl ester groups. They revealed new signals corresponding to hydroxamate (NHOH) group protons within the ranges of *δ* (11.17–10.48 ppm) and (10.28–8.98 ppm). Compounds 7b–i and 7k are present as two conformers, with different ratios, as extracted from ^1^H NMR spectra. Additionally, duplicate signals were observed in the ^13^C NMR spectra. For example, in the ^1^H NMR spectrum of 7b, signal duplication was observed for the aromatic protons of iniparib ring; appearing as two sets of duplicate doublets, and two doublets of doublet signals. Similar duplication was also observed for proton signals of *para* disubstituted benzene ring. This behavior may be attributed to the presence of these compounds as *cis*/*trans* amide rotamers, Fig. 3.

**Fig. 3:**
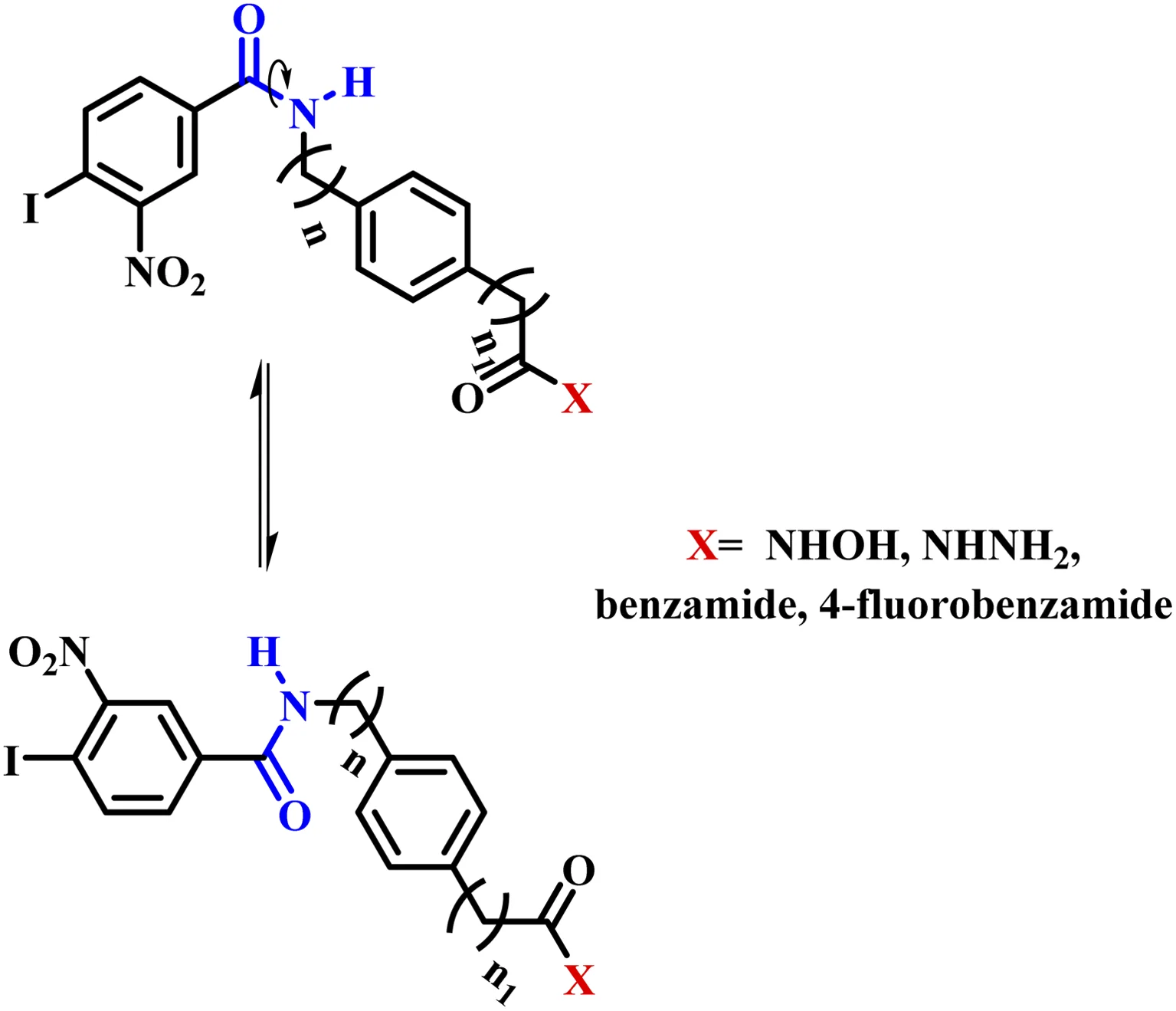
*Cis*/*trans* amide conformers of compounds 7b–i and 7k.

Furthermore, the HRMS (ESI) of compound 7a, as a sample instance, revealed a [M + H]^+^ peak at 427.9000.

Compounds 6a–c were prepared by treating the corresponding esters 5a–c with a 3 M alcoholic solution of KOH, and the crude products were recrystallized from ethyl acetate: hexane in 72–92% yields. The structures of compounds 6a–c are confirmed by the absence of the ester proton and the appearance of a broad singlet signal at *δ* 12.80, as in compound 6a, indicative of a carboxylic OH proton. Furthermore, the IR spectra of 6a, as an example, revealed a broad band between 3000 and 2500 cm^−1^, indicative of the carboxylic OH group, and a strong, intense peak at 1673 cm^−1^, corresponding to the carboxylic carbonyl group.

Subsequent treatment of compounds 6a–c with hydrazine hydrate, *o*-phenylenediamine, or 4-fluoro-*o*-phenylenediamine *via* CDI in dichloromethane and acetonitrile as cosolvent afforded the corresponding hydrazide derivatives 7d–f, benzanilide derivatives 7g–i, and 4-fluoro-benzanilide derivatives 7j–l, respectively. The hydrazide derivatives 7d–f, 7h, 7i, and 7k were purified by recrystallization from aq. methanol. Compounds 7g, 7j, and 7l were purified by column chromatography using chloroform : methanol (10 : 0.5) as the eluent.

The ^1^HNMR spectrum (400 MHz, DMSO-*d*_6_) of compound 7e, as an example, is characterized by the presence of two broad singlet signals at *δ* 9.17 and 4.35 ppm corresponding to the carbohydrazide moiety CONHNH_2_. ESI-HRMS of compound 7e revealed the presence of a [M + H]^+^ peak at 441.0000. The NMR spectra of targeted compounds 7g–i and 7j–l showed additional signals corresponding to the 2-aminoanilide and 4-fluoro-2-aminoanilide moieties, respectively. ^1^H NMR spectrum of compound 7h (400 MHz, DMSO-*d*_6_), as an example, revealed the presence of an additional signal at *δ* (7.61–6.52 ppm) corresponding to 2-aminoanilide aromatic protons and a signal at *δ* 4.86 ppm corresponding to aromatic NH_2_. Moreover, HRMS (ESI) of compound 7h, for example, showed a [M + H]^+^ peak at 517.0000.

Two-dimensional heteronuclear single quantum coherence (^1^H–^13^C HSQC) spectroscopy further confirmed the structure of compound 7h (Fig. 4), revealing direct one-bond (1J_C–H_) connectivity between proton resonances and their corresponding carbons. In the aliphatic region, a particular cross-peak at *δ*_H_ 3.50/*δ*_C_ 42.64 ppm revealed the presence of a single methylene group (CH_2_) connecting the aromatic framework to the terminal amide linker. The complex aromatic pattern (*δ*_H_ 6.54–8.50 ppm, *δ*_C_ 116.11–141.91 ppm) was divided into three distinct electronic environments corresponding to the constituent phenyl rings. The protons of the most electron-deficient Ring I (3-nitrophenyl ring) were the most downfield, owing to the deshielding effects of the nitro and carbonyl groups. The proton H_C_ at *δ*_H_ 8.50 ppm was *ortho*-positioned to the two electron-withdrawing groups and correlated with the carbon at *δ*_C_ 124.34 ppm. The substitution pattern of the nitro-substituted ring was confirmed by cross-peaks between protons H_d_ (8.29 ppm), H_e_ (7.98 ppm), and H_f_ (7.72 ppm) and their respective carbons at *δ*_C_ 124.34, 132.72, and 129.82 ppm.

**Fig. 4:**
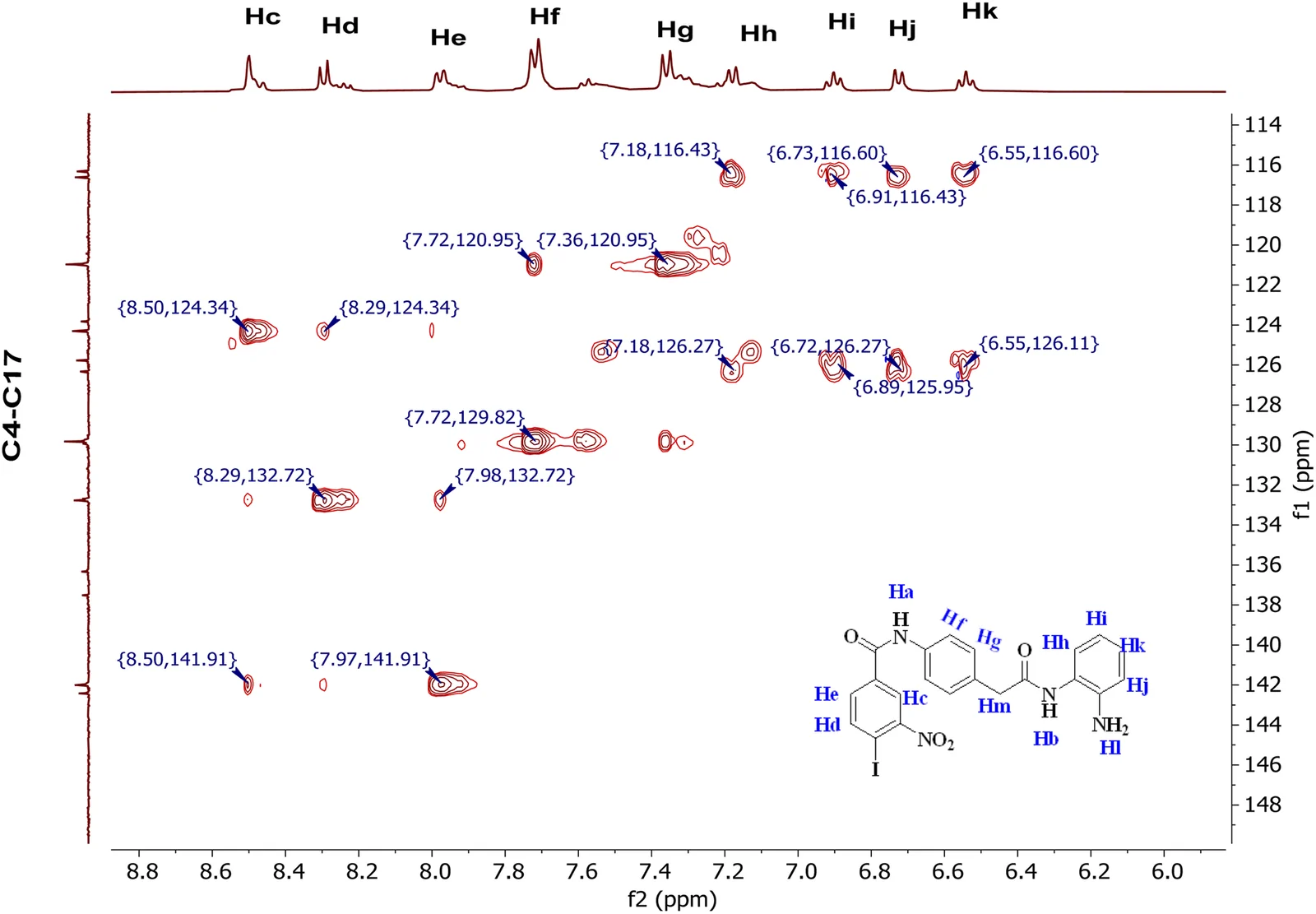
(^1^H–^13^C HSQC) spectrum of compound 7h.

The middle aromatic ring showed intermediate chemical changes, such as a cross-peak with Hg at *δ*_H_ 7.36 ppm, corresponding to *δ*_C_ 120.95 ppm. The terminal 2-aminoaniline ring's strong shielding was attributed to the free aromatic amino group's strong electron-donating resonance effect. This electronic amplification caused a significant upfield shift of the relevant heteronuclear correlations. The combined H_h_ at *δ*_H_ 7.18 ppm and H_j_ at *δ*_H_ 6.72 ppm protons on the carbon matrix at *δ*_C_ 126.27 ppm. The most shielded resonances were assigned to H_i_ at *δ*_H_ 6.73 ppm and H_k_ at *δ*_H_ 6.55 ppm, which were correlated to carbons at *δ*_C_ 116.60 and 116.11 ppm, respectively. The upfield shift of the *ortho*- and *para*-carbons relative to the amino group completely confirms the regiochemistry of the terminal aniline unit.

Finally, the lack of any correlation for the quaternary carbonyl carbons at *δ*_C_ 169.66 and 163.16 ppm, together with the precise identification of all tertiary aromatic carbons, aligns well with the suggested structure of compound 7h.

### 3.2. Biology

#### 3.2.1. Cell viability assay

To evaluate the safety of the newly synthesized compounds (7a–l), their impact on the viability of normal human mammary gland epithelial cells (MCF-10 A) was assessed. Following a four-day incubation, an MTT assay was performed.^30,31^ As shown in Table 1, even at a high concentration of 50 µM of the tested compounds, none of these compounds exhibited cytotoxic effects, with all derivatives maintaining cell viability above 89%.

**Table 1:** *In vitro* inhibitory activities (%) of 7a–l against HDAC-1, -2, -3, and -8 isoforms^a^

Compound	Cell viability%	Inhibitory %			
		HDAC-1	HDAC-2	HDAC-3	HDAC-8
7a	92	68	93	19	≤5
7b	90	36	71	<10	≤5
7c	89	57	88	<10	≤5
7d	91	64	91	12	≤5
7e	90	51	82	<10	≤5
7f	88	60	87	11	≤5
7g	91	12	62	<10	≤5
7h	90	30	70	<10	≤5
7i	93	27	65	<10	≤5
7j	92	15	68	<10	≤5
7k	89	42	77	<10	≤5
7l	91	46	80	<10	≤5
Vorinostat	—	90	91	90	≤5

a —: Not determined.

##### 3.2.1.1. HDAC enzymatic inhibitory assay

Enzymatic evaluation of HDAC inhibition followed an established three-stage approach: (a) initial screening for primary hit identification, (b) selectivity profiling against a panel of HDAC isoforms, and (c) IC_50_ values determination.^16,33^ In the first phase, compounds 7a–l were screened at a concentration of 10 µM against class I enzymes (HDAC-1, -2, -3, and -8), with results summarized in Table 1. Vorinostat (0.5 µM) was included as an internal reference control to confirm assay sensitivity across the different isoforms.

The investigated compounds (7a–l) exhibited significant isoform-selective inhibitory profile for class I HDACs, demonstrating a pronounced preference for HDAC-2 and HDAC-1 compared to HDAC-3 and HDAC-8. Throughout the series, HDAC-2 emerges as the main biological target, where all tested compounds demonstrated moderate to strong inhibitory efficacy. Compound 7a (*n* = *n*_1_ = 0; ZBG = NHOH) is the most effective lead derivative in the series, with an exceptional 93% inhibition of HDAC-2. Almost identical to compound 7d (*n* = *n*_1_ = 0; ZBG = NHNH_2_) at 91%, compound 7c (*n* = 1; *n*_1_ = 0; ZBG = NHOH) at 88%, and compound 7f (*n* = 1; *n*_1_ = 0; ZBG = NHNH_2_) at 87%. The least potent molecule in the series, 7g (*n* = *n*_1_ = 0; ZBG = 2-NH_2_C_6_H_4_NH-), exhibits considerable inhibition at 62%, suggesting that the chemical structure of this series possesses a significant baseline preferential for the HDAC-2 catalytic site.

The inhibitory activity against HDAC-1 has a comparable structural pattern, but at a generally reduced magnitude. Compounds 7a (68%) and 7d (64%) are the most promising candidates against HDAC-1, demonstrating that the structural modifications in these derivatives enhance interactions inside the binding sites of both HDAC-1 and HDAC-2. Conversely, structural variations in compounds 7g (*n* = *n*_1_ = 0; ZBG = benzamide), 7h (*n* = 0; *n*_1_ = 1; ZBG = 2-NH_2_C_6_H_4_NH-), 7i (*n* = 1; *n*_1_ = 0; ZBG = 2-NH_2_C_6_H_4_NH-), and 7j (*n* = 0; *n*_1_ = 0; ZBG = 4-F-2-NH_2_C_6_H_3_NH-) negatively impact binding to HDAC-1, resulting in inhibition levels below 30%, with compound 7g demonstrating a minimum of 12% inhibition. This significant difference suggests that the steric or electronic requirements for the HDAC-1 pocket are substantially more restrictive than those for HDAC-2, and that certain chemical modifications can significantly impede target interaction.

While the reference control Vorinostat expectedly exhibited broad-spectrum, non-selective baseline inhibition across HDAC-1 (90%), HDAC-2 (91%), and HDAC-3 (90%), the 7a–l series displayed a distinct isoform-selective pattern. Unlike broad-spectrum inhibitors, these new scaffolds do not affect HDAC-3 or HDAC-8. In fact, inhibition against HDAC-3 drops below 10% for the entire series, except for minor baseline activities in 7a (19%), 7d (12%), and 7f (11%). Furthermore, all tested compounds are entirely inactive (≤5%) against HDAC-8, which is consistent with Vorinostat's lack of activity against this enzyme.

#### 3.2.2. Structure–activity relationship (SAR) analysis

##### 3.2.2.1. Effect of the zinc-binding group (ZBG)

The structural comparison of compounds 7a (*n* = *n*_1_ = 0; ZBG = NHOH), 7d (*n* = *n*_1_ = 0; ZBG = NHNH_2_), 7g (*n* = *n*_1_ = 0; ZBG = 2-NH_2_C_6_H_4_NH-), and 7j (*n* = *n*_1_ = 0; ZBG = 4-F-2-NH_2_C_6_H_3_NH-) provides clear insight into how the chemical nature of the ZBG affects biological activity, as the core structural framework (*n* = *n*_1_ = 0) is identical. Because linker length and cap moiety have been fixed, the different inhibitory percentages are exclusively due to the capacity of each functional group to chelate the catalytic (Zn^2+^) ion and fit into the enzyme's active site pocket. The biological findings demonstrate a clear and constant order of activity of the ZBGs against the principal targets, beginning with the highly potent hydroxamic acid (7a) and progressing to the structurally similar hydrazide bioisostere (7d) and ending with the bulky benzamides (7g and 7j).

The superiority of compound 7a (93% for HDAC-2, 68% for HDAC-1) supports the high standard in epigenetic drug design, which employs hydroxamic acid (NHOH) as the ZBG.^23^ Its geometrical flexibility allows it to generate strong bidentate coordinate interactions with the (Zn^2+^) ion, as well as an ideal network of hydrogen bonds with the conserved amino acid residues around the pocket's base.^39^ Replacement of this group by hydrazide (NHNH_2_) in compound 7d, still retains the inhibitory activity almost completely, with 91% inhibition against HDAC-2 and 64% against HDAC-1. This minor drop demonstrates that the hydrazide motif is a superior, less toxic bioisostere for hydroxamic acid in this compact structure.

In contrast, replacing the ZBG with a benzamide motif in compound 7g leads to a significant reduction in activity, particularly against HDAC-1, where inhibition decreases to a record low of 12%, and HDAC-2, which declines to 62%. This significant loss of activity is due to steric hindrance from the rigid benzene ring blocking the narrow catalytic channel entrance. Incorporating a strongly electron-withdrawing fluorine atom at the *para* position of compound 7j optimizes zinc binding. This 4-fluoro-benzamide ZBG heavily polarizes the adjacent amide carbonyl oxygen, thereby enhancing electrostatic coordination with the Zn^2+^ ion. This electronic modification achieves a robust 68% inhibition against the more adaptable HDAC-2 pocket yet fails to improve the poor binding affinity against HDAC-1 (15%).

##### 3.2.2.2. Effect of linker size and variation (*n* and *n*_1_)

The comparison of the length and position of the flexible methylene linkers (CH_2_)n and (CH_2_)*n*_1_ reveals that the size of the spacer influences the binding affinity within the target binding sites, where the ZBGs remain unchanged. The ideal spatial configuration for simultaneous HDAC-1/HDAC-2 inhibition is attained through the completely planar zero-linker framework (*n* = 0, *n*_1_ = 0) for the small and flexible ZBGs, specifically the hydroxamic acids (7a–c) and hydrazides (7d–f). The efficacy reduces when a single methylene (CH_2_) is introduced on either side of the core phenyl ring, but the extent of efficacy reduction is significantly influenced by the position of the chain extension.

Elongation of the linker on the zinc-binding side of the molecule (compounds 7b and 7e, *n* = 0, *n*_1_ = 1) significantly reduces efficacy, thereby indicating that the modified alignment adjacent to the ZBG impairs optimal zinc chelation. Conversely, linker elongation on the cap side of the molecule (7c and 7f, (*n* = 1, *n*_1_ = 0) is far better tolerated. This is because the spacious, solvent-exposed surface rim of class I HDACs easily accommodates minor structural extensions of the capping core.

The linker preference is completely inverted in the bulky unsubstituted benzamide subseries (7h and 7i). The zero-linker parent, 7g (*n* = *n*_1_ = 0; ZBG = 2-NH_2_C_6_H_4_NH-) blocks the active site entrance by placing the bulky aromatic benzamide ring directly against it, limiting entry to the pocket. The addition of a methylene spacer to the ZBG side of compound 7h (*n* = 0; *n*_1_ = 1; ZBG = 2-NH_2_C_6_H_4_NH-) significantly enhances efficacy to 30% against HDAC-1 and 70% against HDAC-2. The extra methylene unit provides a flexible hinge, enabling the large aromatic ZBG to adjust its orientation and bypass the narrow geometric constraints of the internal channel.

In the 4-fluoro-benzamide subseries (7k and 7l), adding a fluorine atom improves the molecule's electronic properties. This electronic boost helps the compounds overcome crowding and fit into tight spaces. Compound 7l (*n* = 1, *n*_1_ = 0; ZBG = 4-F-2-NH_2_C_6_H_3_NH-) exhibits a significant 80% inhibition of HDAC-2, closely succeeded by its analogue 7k (*n* = 0, *n*_1_ = 1; ZBG = 4-F-2-NH_2_C_6_H_3_NH-) with 77% inhibition. These findings indicate that favorable electronic properties compensate for size differences in the HDAC-2 binding pocket. However, they cannot overcome the strict structural crowding within the narrow HDAC-1 channel.

#### 3.2.3. *In vitro* HDAC-1/HDAC-2 inhibitory activities

Based on preliminary cell viability and screening profiles from Table 1, the most effective candidates (7a, 7c, 7d, 7e, 7f, and 7l) have been selected for further evaluation to determine their half-maximal inhibitory concentration (IC_50_) values against the human HDAC-1 and HDAC-2 isoforms. The enzymatic profiling results are summarized in Table 2. The evaluated derivatives demonstrated remarkable potency as target-specific HDAC-2 inhibitors, exhibiting enhanced nanomolar activity within a narrow IC_50_ range of 37.16 to 69.55 nM, compared to the reference vorinostat of IC_50_ value of 30.70 nM.

**Table 2:** *In vitro* inhibitory potencies (IC_50_) and selectivity profiles of 7a, 7c, 7d, 7e, 7f, and 7l against human HDAC-1 and HDAC-2 enzymes^a^

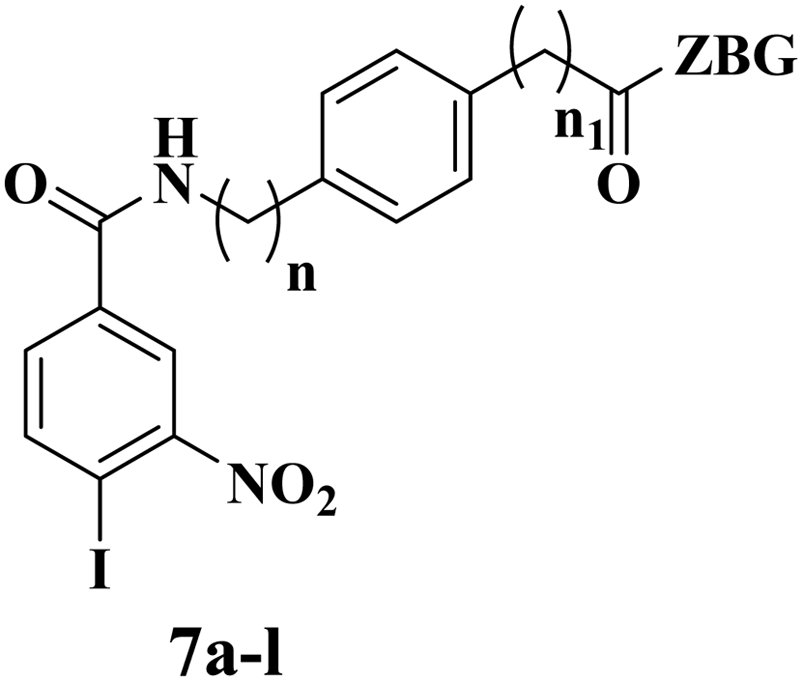						
Compound	*n*	*n*_1_	ZBG	IC_50_ (nM)		Selectivity index (SI)
				HDAC-1	HDAC-2	
7a	0	0	NHOH	113.30 ± 4.25	37.16 ± 2.12	3.05
7c	1	0	NHOH	253.50 ± 15.30	45.50 ± 1.80	5.57
7d	0	0	NHNH_2_	198.10 ± 11.90	39.30 ± 2.70	5.05
7e	0	1	NHNH_2_	>300	64.01 ± 3.54	>4.68
7f	1	0	NHNH_2_	>300	53.30 ± 1.90	>5.62
7l	1	0	4-F-2-NH_2_C_6_H_3_NH	>300	69.55 ± 3.47	4.31
Vorinostat	—	—	—	22.10 ± 1.50	30.70 ± 2.70	0.72

a —: Not applicable.

Conversely, these derivatives exhibited a notable reduction in binding affinity for the closely related HDAC-1 isoform, with a markedly elevated IC_50_ range of 113.30 to over 300 nM, as several compounds above the maximum testing threshold of 300 nM. This differential potency results in a moderate but clear selectivity preference for HDAC-2 over HDAC-1 (with selectivity indices between roughly 3.05 to 5.57). Rather than acting as mixed, cross-reactive agents, these scaffolds provide a promising molecular scaffold for the development of targeted epigenetic treatments.

The Selectivity Index (SI), defined as the IC_50_ (HDAC-1)/IC_50_ (HDAC-2) ratio, was established to determine the quantitative preference of these derivatives toward the HDAC-2 isoform. Vorinostat, a therapeutic pan-HDAC inhibitor, showed a minor selectivity for the HDAC-1 isoform, with a SI of 0.72 (IC_50_ = 22.10 ± 1.50 nM against HDAC-1 and 30.70 ± 2.70 nM against HDAC-2). All evaluated compounds successfully inverted this pattern, demonstrating clear preference for HDAC-2 over HDAC-1 with SI values ranging from 3.05 to > 5.63.

A thorough examination of the IC_50_ data from Table 2 reveals the significance of linker length and the nature of the ZBG in isoform distinction. In the hydroxamate-containing derivatives (ZBG = NHOH), increasing the carbon spacer length from (*n* = 0) (7a) to (*n* = 1) (7c) decreased binding affinity against HDAC-1 by more than two-fold, with IC_50_ increasing from 113.30 ± 4.25 nM to 253.50 ± 15.30 nM. By contrast, structural elongation had a moderate effect on HDAC-2 inhibition, with the IC_50_ changing from 37.16 ± 2.12 nM to 45.50 ± 1.80 nM, boosting the SI from 3.05 to an astounding 5.57. This demonstrates that for HDAC-1, the catalytic channel cannot withstand the steric or conformational limitations imposed by a longer linker, but the HDAC-2 pocket can easily accommodate the enlarged structure.

Additionally, substituting the hydroxamate group in 7a with a hydrazide ZBG (–NHNH_2_) in compound 7d (*n* = 0, *n*_1_ = 0) significantly reduced its efficacy against HDAC-1 (IC_50_ = 198.10 ± 11.90 nM) while preserving strong nanomolar efficacy against HDAC-2 (IC_50_ = 39.30 ± 2.70 nM), resulting in a 5.04-fold selectivity for the anticipated isoform.

Compounds 7e, 7f, and 7l exhibited the highest isoform discrimination, with HDAC-1 inhibition beyond the detectable limit of 300 nM, whereas inhibition of HDAC-2 remained consistently potent. The inclusion of a single methylene unit in the hydrazide derivatives, either at ZBG side (*n*_1_ = 1, 7e) or within the cap-moiety side (*n* = 1, 7f), entirely disrupted critical binding interactions in the HDAC-1 pocket, rendering IC_50_ values indeterminable (ND, >300 nM). Nonetheless, they exhibited a strong affinity for HDAC-2, with IC_50_ values of 64.01 ± 3.54 nM and 53.30 ± 1.90 nM, resulting in conservative minimal SI estimates of >4.69 and >5.63, respectively. Compound 7l demonstrated a comparable selective profile, as the substantial 4 F-benzamide ZBG consistently inhibited the HDAC-1 interaction (IC_50_ > 300 nM) while effectively accommodating the HDAC-2 catalytic cavity (IC_50_ = 69.55 ± 3.47 nM; SI > 4.31).

To investigate the therapeutic efficacy and safety profile of the synthesized series, the patterns in isoform selectivity against HDAC-1 and HDAC-2 were associated with cell viability percentages derived from the initial *in vitro* screening (Table 1). Non-selective zinc chelation across numerous homeostatic class I HDAC isoforms causes severe clinical toxicities and cellular stress, which are frequently associated with typical pan-HDAC inhibitors. Importantly, our target derivatives showed very high cell survival rates (strictly between 88% and 92%), which directly correlates with their improved preferential inhibition for HDAC-2 over HDAC-1.

This high cell survival profile demonstrates that a shift in compound affinity away from HDAC-1 reduces the cytotoxicity associated with pan-class I disruption. For example, compound 7c (SI = 5.57), the most selective sub-300 nM derivative, maintained 89% cell viability. The highly selective threshold-excluded hydrazides 7e (SI > 4.69) and 7f (SI > 5.63) had little cytotoxicity, with cell survival of 90% and 88%, respectively. This novel link demonstrates a structure-toxicity relationship in which structural alterations that boost HDAC-2 preferential inhibition reduce off-target cellular toxicity, providing a safer chemical scaffold for targeted epigenetic therapeutics.

Finally, the structural factors influencing the enzymatic inhibitory percentages in Table 1 were entirely consistent when assessing the absolute potencies (IC_50_) against HDAC-1 and HDAC-2 in Table 2; specifically, the hydroxamate moiety emerged as the most effective ZBG, followed in order by the hydrazide and 4 F-benzamide modifications. Additionally, minimizing spacer lengths to (*n* = *n*_1_ = 0) resulted in optimal potency, as further chain elongation consistently weakened inhibitory activity.

#### 3.2.4. *In vitro* growth inhibitory activity against human cancer cell lines

Compounds 7a, 7c, and 7d were chosen for examination in cell-based assays utilizing propidium iodide (PI) fluorescence assay^32^ because of their remarkable nanomolar efficacy against HDAC-2 and preferential inhibition over HDAC-1. To definitively validate this isoform preference, we selected a panel of cancer models encompassing a diverse array of cancer types, including MDA-MB-231 (triple-negative breast cancer), A-549 (non-small cell lung cancer), and SEM (B-cell acute lymphoblastic leukemia) cell lines. These particular lines provide as an impressive biological testing platform, demonstrating the overexpression of class I HDACs and a substantial dependence on HDAC-2-mediated survival pathways.^40^ The resulting IC_50_ values against the tested cell lines are presented in Table 3. The clinically approved, Vorinostat (SAHA), served as the reference control.

**Table 3:** IC_50_ values of 7a, 7c, and 7d against a panel of cancer cell lines

Comp	*In vitro* cytotoxicity IC_50_ (µM)		
	MDA-MB-231	A-549	SEM
7a	5.47 ± 0.30	5.95 ± 0.40	3.18 ± 0.10
7c	7.64 ± 0.40	7.26 ± 0.30	4.17 ± 0.20
7d	6.34 ± 0.40	6.72 ± 0.50	3.98 ± 0.20
Vorinostat	4.50 ± 0.20	4.17 ± 0.20	5.23 ± 0.30

The cell-based screening revealed a remarkable, tissue-specific sensitivity profile. The new compounds exhibited modest, single-digit micromolar growth inhibition activity against the solid tumor models (MDA-MB-231 and A-549), slightly inferior to the reference medication, yet indicated excellent growth inhibitory efficacy against the hematological SEM cell line. Compounds 7a (IC_50_ = 3.18 ± 0.10 µM), 7c (IC_50_ = 4.17 ± 0.20 µM), and 7d (IC_50_ = 3.98 ± 0.20 µM) considerably surpassed Vorinostat (IC_50_ = 5.23 ± 0.30 µM) in inhibiting the growth of leukemia cells. This significant shift underscores a specific therapeutic susceptibility that differentiates our molecular scaffold from conventional broad-spectrum medicines.

The increased sensitivity observed in the SEM cell line relative to the tested solid tumor lines (MDA-MB-231 and A549) correlates with the preferential class I isoform profile of this series, particularly their marked preference for HDAC-2 relative to HDAC-1. Vorinostat, the positive control, exhibits a non-selective pan-HDAC inhibition profile with an unsatisfactory selectivity index (SI) of 0.72 in cell-free enzymatic tests. Conversely, our synthesized 4-iodo-3-nitrophenyl-capped compounds function as preferential inhibitors of HDAC-2, resulting in significant selectivity indices favoring HDAC-2 (7a, SI = 3.05; 7c, SI = 5.57; and 7d, SI = 5.05).

Hematological malignancies, including acute leukemia models like SEM, have been reported to exhibit a pronounced epigenetic reliance on class I HDAC isoforms, where HDAC-2 often serves as a key player in dysregulated transcriptional programs and survival mechanisms. While a broader cell line panel is required to definitively establish comprehensive tissue-specific selectivity,^41,42^ our preliminary results indicate that these newly developed compounds exert a potent cellular influence that uniquely impacts the tested leukemia model. Consequently, the superior potency of 7a, 7c, and 7d over Vorinostat in the SEM cell line supports our design rationale, suggesting that refining class I selectivity toward HDAC-2-preferential inhibition represents a promising direction for further therapeutic optimization.

#### 3.2.5. Western-blot analysis of compound 7a

A preliminary western blot screening (*n* = 1) ^34,35^was performed on SEM cells using compound 7a to validate whether the cell-free enzymatic affinity and phenotypic cytotoxicity observed across our series translate into target regulation in live cancer cells. The intracellular expression levels of the primary target, HDAC-2 (60 kDa), post-treatment were evaluated, with β-actin (42 kDa) serving as an internal loading control to assess relative protein levels.^43^ Densitometric evaluation of this single experiment indicated an observational tendency toward reduction in HDAC-2 protein levels following 7a treatment (Fig. 5).

**Fig. 5:**
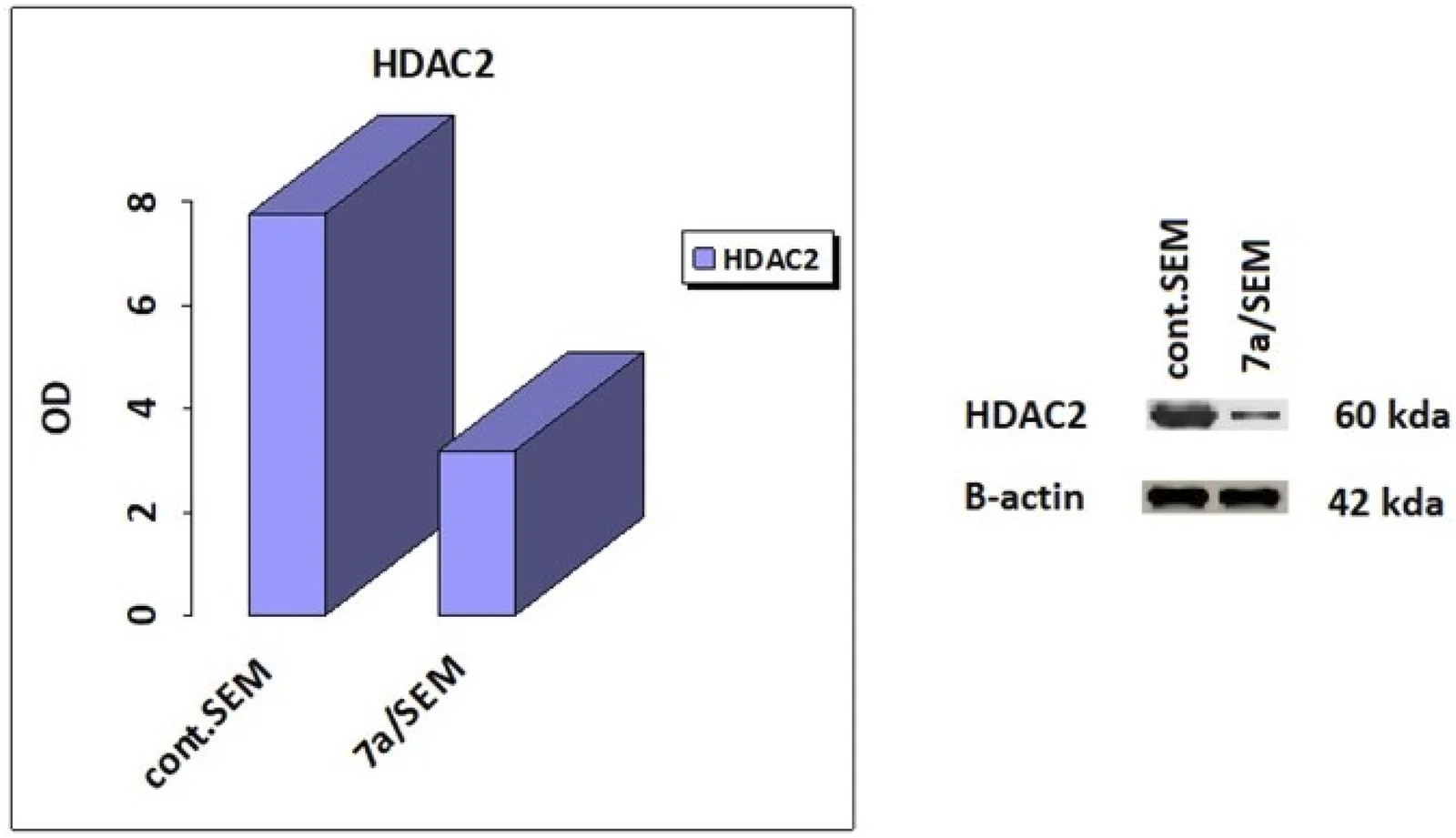
Western blot analysis of HDAC-2 expression in SEM cancer cells. Cells were treated with compound 7a (1 mg mL^−1^) for 24 h. β-Actin was used as the loading control. Data are presented as mean ± SD (*n* = 3 independent experiments).

The control SEM cells exhibited a baseline optical density (OD) value of 7.77 for HDAC-2, which alignment with the common overexpression of class I HDACs in leukemic tumors, while the OD of the HDAC-2 protein band was measured at 3.18 following incubation with compound 7a (representing an approximate 59% reduction in relative band intensity for this batch). This descriptive drop in relative HDAC-2 abundance provides an initial, qualitative indication of possible cellular protein downregulation following treatment with compound 7a.

While this single screening does not carry statistical power and cannot conclude whether this pattern stems from direct target-mediated degradation or downstream transcriptional suppression, it provides a preliminary visual correlation with the growth inhibitory activity observed in our biological assays. This descriptive observation serves as a useful preliminary linkage between our cell-free enzymatic data and potential intracellular effects, supporting the core design of this 4-iodo-3-nitrophenyl series, though extensive independent biological replication will be required in future work to statistically validate and characterize the exact magnitude of this downregulation.

#### 3.2.6. Cell cycle analysis of compound 7a

To clarify the cellular mechanism responsible for the notable growth inhibitory effects of this set of compounds, flow cytometry^36^ was utilized to evaluate the distribution of human SEM cells across the several phases of cell division after treatment with compound 7a. Under baseline untreated conditions, control SEMs displayed normal proliferation patterns, with a majority of cells in the resting G0/G1 phase (59.66%), a significant actively replicating group in the synthesis (S) phase (33.13%), and a small percentage advancing through the mitotic preparation (G2/M) phase (7.21%). Treatment with compound 7a completely destroys this usual distribution pattern, reducing the (G0/G1) population to (37.86%) and the active synthesis phase population to (15.92%). This reduction was accompanied by a considerable multi-fold increase in tumor cells in the G2/M phase, attaining significance at 46.22%. This notable change signifies that compound 7a induces a precise and considerable disruption in the cell cycle at the key G2/M threshold, thus preventing the subpopulations from proceeding to mitosis, Fig. 6.

**Fig. 6:**
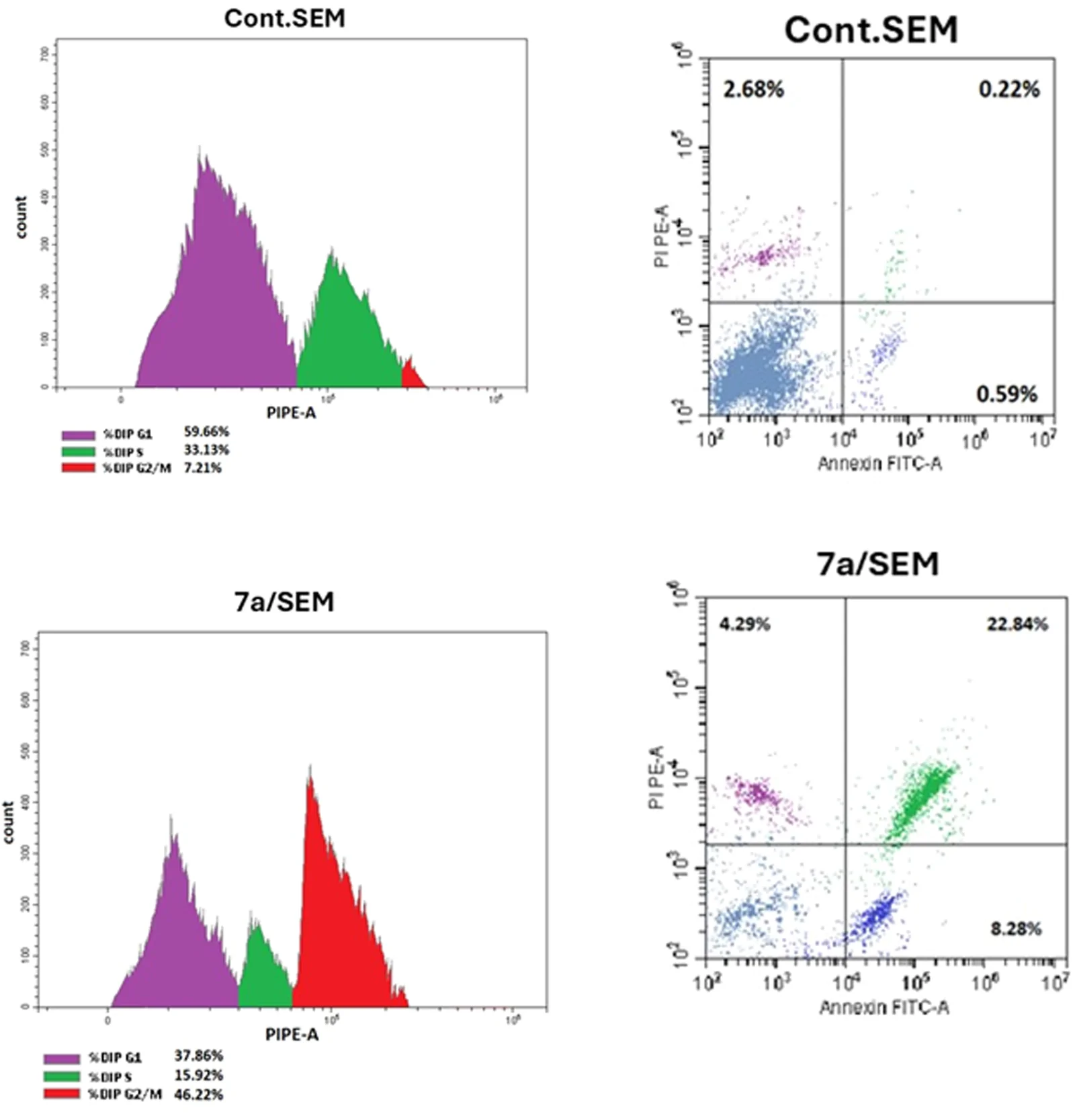
Flow cytometric cell cycle analysis of SEM cancer cells treated with compound 7a. Cells were treated with compound 7a (1 mg mL^−1^) for 24 h. DNA content was quantified using the ab139418 propidium iodide flow cytometry kit. Data are presented as mean ± SD (*n* = 3 independent experiments).

The observed cell cycle arrest in the G2/M phase is consistent with the target's preferential inhibition and intracellular HDAC-2 depletion, as established in prior tests.^44,45^ Class I histone deacetylases, particularly the HDAC-2 isoform, are critical for maintaining the G2/M DNA damage and spindle checkpoints. Compound 7a selectively suppresses and downregulates HDAC-2, altering the acetylation status of core histones and centromere-associated proteins. This epigenetic modification prevents the normal expression and assembly of essential mitotic drivers. Compound 7a permanently arrests SEM cells in the post-replicative G2/M phase, halting mitosis and forcing the cells into apoptosis. This selective inhibition confirms the molecular mechanism driving the potent growth inhibitory action observed in our cell-based assays.

#### 3.2.7. Apoptosis and necrosis analysis of compound 7a

To assess whether the G2/M cell cycle arrest is an irreversible phenomenon that impedes cancer cell proliferation *via* programmed cell death, flow cytometric analysis utilizing Annexin V/PI double-staining^46^ was conducted on human SEM cells treated with compound 7a, Table 4.

**Table 4:** Results of apoptosis and necrosis of compound 7a

Compound/Cell line	Early apoptosis (%)	Late apoptosis (%)	Total apoptosis (early + late)	Necrosis (%)
7a/SEM	8.28	22.84	31.12	4/29
Control/SEM	0.59	0.22	0.81	2.68

As expected, under untreated control conditions, the SEM population demonstrated high viability, with a total apoptotic rate of only 0.81% (including 0.59% early apoptotic and 0.22% late apoptotic subpopulations) and a background necrotic percentage of 2.68%. Conversely, incubation with compound 7a prompted a substantial, multi-fold shift towards programmed cell death pathways. The total apoptotic cell population increased dramatically to 31.12%, comprising a considerable 8.28% of cells in early apoptosis and a predominant 22.84% in late-stage apoptosis. Concurrently, the necrotic subpopulation remained minimal (4.29%), supporting the hypothesis that the cellular damage induced by 7a predominantly directs cells toward regulated apoptotic death rather from uncontrolled necrotic lysis.

This significant induction of apoptosis strongly correlates with the biological profile observed during our enzymatic screening. Specifically, treatment with compound 7a reduces cellular HDAC-2 levels, resulting in a persistent G2/M phase checkpoint arrest, halting cancer cell development. The cells seem to experience programmed cell death as they cannot surpass this checkpoint barrier to advance to mitosis. While the exact downstream molecular mediators remain to be elucidated, this phenotypic shift is hypothesized to involve classical pro-apoptotic signaling pathways triggered by HDAC-2 suppression. Experimentally, the elevated proportion of late apoptotic cells (22.84%) compared to early apoptotic cells (8.28%) highlights a pronounced commitment to apoptotic death. This robust apoptotic response provides a plausible explanation for the low micromolar IC_50_ values observed in our PI assays.

### 3.3. Docking study

A molecular docking analysis was performed at the active site of human HDAC-2 (PDB ID: 4LXZ)^40,47^ to obtain structural insights into the observed inhibitory activity and binding modes of the target. The top-ranked docking posture of the primary lead, compound 7a (predicted binding energy: −7.8 kcal mol^−1^), was modelled and compared to the clinical reference medicine, Vorinostat (predicted binding energy: −8.21 kcal mol^−1^). While these scores indicate a highly favorable binding orientation within the active site, which matches our biochemical results, they are computational models rather than absolute quantitative measurements of experimental binding affinity.

Comparative analysis revealed that both inhibitors were successfully docked at the catalytic base, forming key metal-acceptor coordinate bonds between their hydroxamate carbonyl oxygens and the catalytic zinc ion (Zn 401). This zinc coordination is additionally stabilized in both complexes by a conserved hydrogen-bonding network with His145 and His146. Both molecules also use Gly154 to tightly fix the zinc-binding head at the bottom of the pocket, giving a clear molecular explanation for their highly comparable *in vitro* HDAC-2 inhibition profile.

Although exhibiting catalytic similarities, the two inhibitors demonstrate markedly different binding mechanisms *via* the hydrophobic channel and the outer surface rim. Vorinostat navigates the tunnel with its flexible and linear six-carbon aliphatic chain, forming flexible pi–alkyl interactions with Phe155 and Phe210, Fig. 7(A). Vorinostat aliphatic flexibility optimizes van der Waals surface interactions while minimizing steric strain. This elucidates the marginally enhanced binding energy of Vorinostat.

**Fig. 7:**
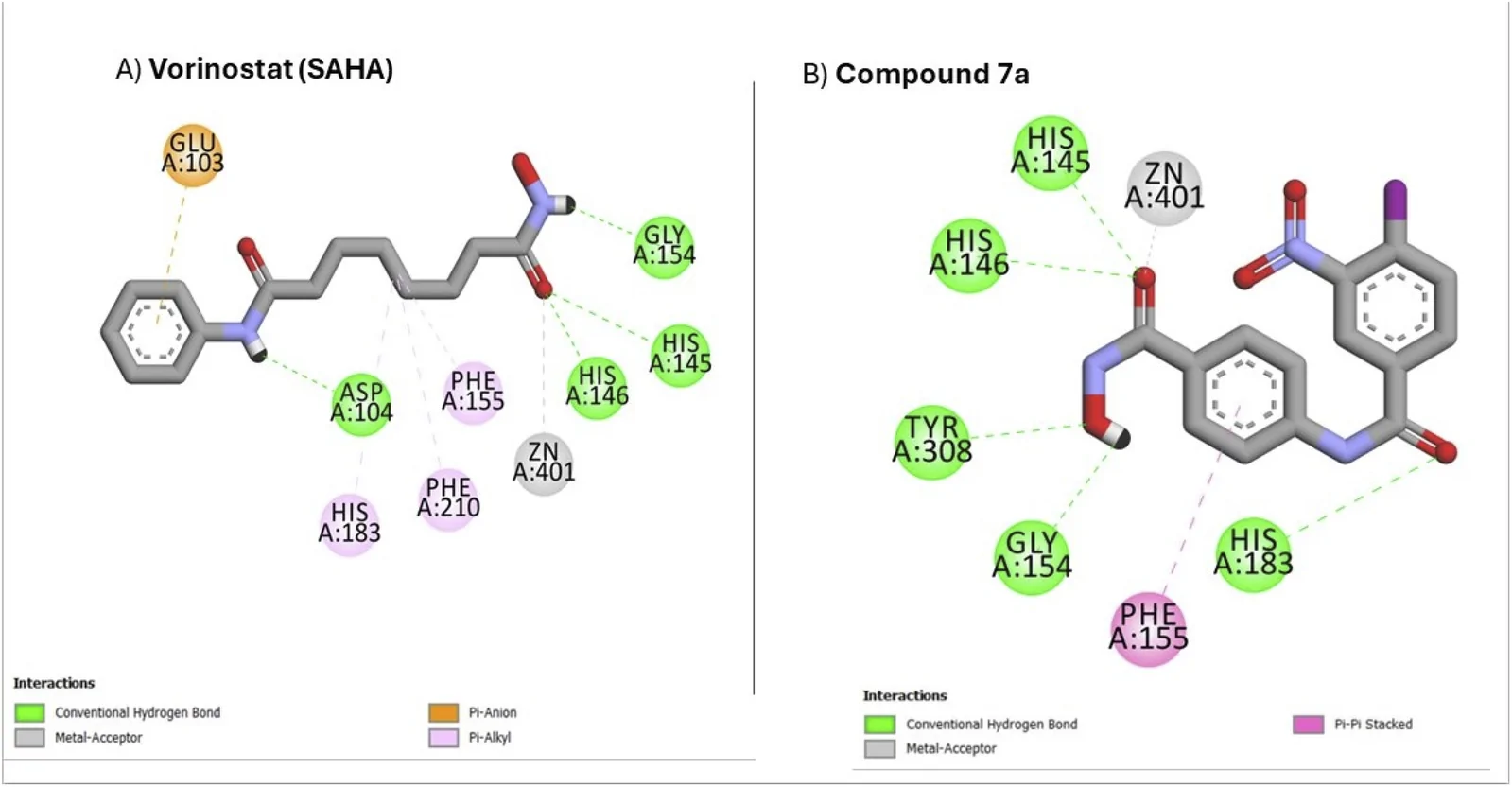
2D representation of docking study of compound 7a and Vorinostat within HDAC-2 active site (A) Vorinostat; (B) compound 7a.

In contrast, compound 7a, which has a zero-linker structure, includes a rigid core phenylene unit within this tight channel. This inflexible ring forms a strongly directed pi–pi stacking interaction with Phe155, giving it a limited degree of conformational mobility while forming a stable structural anchor, Fig. 7(B).

The cap group of Vorinostat is fixed in the solvent-accessible outer pocket through a conventional hydrogen bond between the capping amide NH group of Vorinostat and Asp104, as well as a pi-anion attachment between the terminal phenyl ring of Vorinostat and Glu103. Compound 7a interacts in an alternative manner at the outer pocket, extending its carbonyl oxygen to establish conventional hydrogen bond with His183. The alternate anchoring point positions the substituted 4-iodo-3-nitrobenzamide cap directly onto the enzyme's surface pocket.

The comparative structural study clearly demonstrates compound 7a′s extreme target selectivity. Vorinostat flexible aliphatic chain allows it to adapt smoothly to the changing forms of other class I cavities (resulting in unwanted broad-spectrum pan-inhibition), whereas 7a′s rigidly compacted zero-linker scaffold maintains a rigid geometry. This spatial arrangement fits nicely into the pockets of HDAC-1 and HDAC-2 but causes significant steric conflicts or geometry mismatches within the channels of HDAC-3 and HDAC-8. Finally, these docking studies show that compound 7a successfully takes off a modest loss in flexible binding energy to adopt a highly rigid structure, resulting in high target potency and perfect isoform selectivity.

### 3.4. Physicochemical properties of compound 7a

This section presents a thorough examination of the physicochemical properties of the lead compound 7a using Swiss ADME Predictions.^48^ The essential electronic, structural, and steric features are elaborated upon here; however, assessments of lipophilicity, water solubility, and pharmacokinetic profile have been excluded from the primary text for brevity. Complete datasets outlining these criteria, together with thorough assessments of drug-likeness and medicinal chemistry attributes, can be found in Appendix A of the SI.

The calculated physicochemical properties for compound 7a suggest an appealing drug-like profile in accordance with the established structural criteria for oral bioavailability. The molecular weight of 427.15 g mol^−1^ is below the 500 g mol^−1^ threshold established by Lipinski's Rule of Five,^49^ suggesting an appropriate molecular size that generally enhances membrane permeability.

The molecular hydrogen-bonding capacity improves this drug-like profile, comprising 3 hydrogen-bond donors and 5 hydrogen-bond acceptors, both conforming to the Lipinski standard (≤5 donors and ≤10 acceptors). Furthermore, the molecule demonstrates an ideal equilibrium of structural flexibility, possessing six rotatable bonds, which conforms to Veber's criteria (≤10) for efficient oral absorption. The attributes indicate that compound 7a has the essential structural integrity required for advancement as an orally active pharmaceutical agent.

The electronic and surface properties of 7a offer a more thorough understanding of its potential pharmacokinetics and tissue distribution. The topological polar surface area (TPSA) was measured at 124.25 Å^2^, well below the ideal threshold of 140 Å^2^ for sufficient intestinal epithelial permeability.^50^ Nevertheless, the TPSA value considerably surpasses the 90 Å^2^ threshold often required for passive diffusion across the blood–brain barrier; thus, 7a is probably impeded from entering the central nervous system. This peripheral limitation may be advantageous when the therapeutic target is external to the brain, as it reduces the risk of unwanted effects mediated by the central nervous system. Furthermore, the molecule demonstrates a notable molar refractivity of 91.11, mostly affected by the presence of the heavy and highly polarizable iodine atom, together with 12 aromatic heavy atoms. The increased refractivity indicates that 7a has a considerable molecular volume and a high level of polarizability, suggesting its propensity to form strong van der Waals and hydrophobic interactions within the binding sites of the target receptors.

A notable structural characteristic of compound 7a is a Fraction Csp^3^ score of 0.00, indicating a highly conjugated, wholly planar molecular structure.^51^ The complete absence of saturated carbons amplifies aromatic stacking potential and may augment binding affinity for the target through pi–pi interactions; however, a zero fraction of Csp^3^ is often associated with poor water solubility and heightened crystal lattice energy. The planar configuration may promote dense intermolecular stacking in the solid state, thus hindering dissolution. Thus, although the overall oral bioavailability and structural attributes of compound 7a are exceptionally promising for initial lead optimization, the potential solubility challenges associated with the planar, aromatic core will be a crucial factor in the forthcoming stages of formulation and development.

## 4. Conclusion

We developed, synthesized, and evaluated the biological activity of a novel series of 4-iodo-3-nitrobenzoyl-capped hydrazide and hydroxamate derivatives as a targeted approach for epigenetic cancer therapy. In contrast to traditional pan-HDAC inhibitors with non-selective activity (*e.g.*, Vorinostat), this newly developed series demonstrates a clear preference for the class I HDAC-2 isoform over HDAC-1. In PI assays, the lead derivatives (7a, 7c, and 7d) showed strong growth inhibitory activity against human B-cell acute lymphoblastic leukemia (SEM) cells, outperforming Vorinostat. Western blot analysis revealed substantial intracellular target engagement, resulting in a notable 59% reduction of the total HDAC-2 protein pool. Flow cytometry analysis verified that the reduction of the intracellular target significantly impaired essential mitotic regulators, resulting in a substantial cell cycle arrest at the pivotal G2/M transition checkpoint (46.22%). Ultimately, this extensive checkpoint block was validated using Annexin V/PI double-staining to elicit an effective, regulated apoptotic cascade, thereby elevating total cellular apoptosis to 31.12% and preventing uncontrolled, inflammatory necrosis.

To summarize, the purposeful modification of spacer lengths and different zinc-binding groups in this series resulted in significant derivatives with a distinct preference for HDAC-2 over the homologous HDAC-1 among class I HDACs. With the outstanding cell viability profiles and high isoform discrimination established in class I, future research will include extended profiling against a larger panel of histone deacetylases. Future screens will focus on the selectivity of the lead candidates against other critical regulatory isoforms, such as HDAC-6, HDAC-11, and the class IIa isoforms HDAC-5, -7, and -9. These thorough analyses will map the pan-HDAC selectivity landscape of this new chemical scaffold, allowing for specific therapeutic uses *in vivo*.

## Conflicts of interest

The author reported no potential conflicts of interest(s).

## Supplementary Material

RA-OLF-D6RA08049A-s001

## Data Availability

The authors declare that the data supporting the findings of this study are available within the supplementary information (SI). Supplementary information is available. See DOI: https://doi.org/10.1039/d6ra08049a.
