# Supplementary material for: Design, synthesis, and evaluation of novel hydrazide and hydroxamate derivatives as potent antileukemic agents with preferential HDAC-2 inhibitory activity over class I isoform
Source: RSC Adv. 2026 Nov 3. Online ahead of print. doi: 10.1039/d6ra08049a (PMC13729014; doi:10.1039/d6ra08049a)
Supplement: Supplementary file 1 [file RA-OLF-D6RA08049A-s001.pdf]

Supplementary Information (SI) for RSC Advances.
This journal is © The Royal Society of Chemistry

Design, synthesis, and evaluation of novel hydrazide and hydroxamate derivatives as potent antileukemic agents with preferential HDAC-2 inhibitory activity over Class I isoform

Fatma Elzahraa Ali[1,2], Abdullah Yahya Abdullah Alzahrani[3], Bandar A. Alyami[4], Stefan Bräse[5*], Anber F. Mohammed[1,2], Bahaa G.M. Youssif[1,2*]

[1]Department of Pharmaceutical Organic Chemistry, Faculty of Pharmacy, Assiut University, Assiut 71526, Egypt, [2]Faculty of pharmacy and drug research, Assiut National University, New Assiut City, Assiut, 11511, Egypt; [3]Department of Chemistry, Faculty of Science, King Khalid University, Abha 61413, Saudi Arabia, [4]Department of Pharmaceutical Chemistry, College of Pharmacy, Najran University, Najran, Saudi Arabia, [5]Institute of Biological and Chemical Systems, IBCS-FMS, Karlsruhe Institute of Technology, 76131 Karlsruhe, Germany.

**To whom correspondence should be addressed:*

Bahaa G. M. Youssif, Ph.D. Pharmaceutical Organic Chemistry Department, Faculty of Pharmacy, Assiut University, Assiut 71526, Egypt.

Tel.: (002)-01044353895

E-mail address: bgyoussif2@gmail.com

Stefan Bräse

Institute of Biological and Chemical Systems, IBCS-FMS, Karlsruhe Institute of Technology, 76131 Karlsruhe, Germany. E-mail address: braese@kit.edu

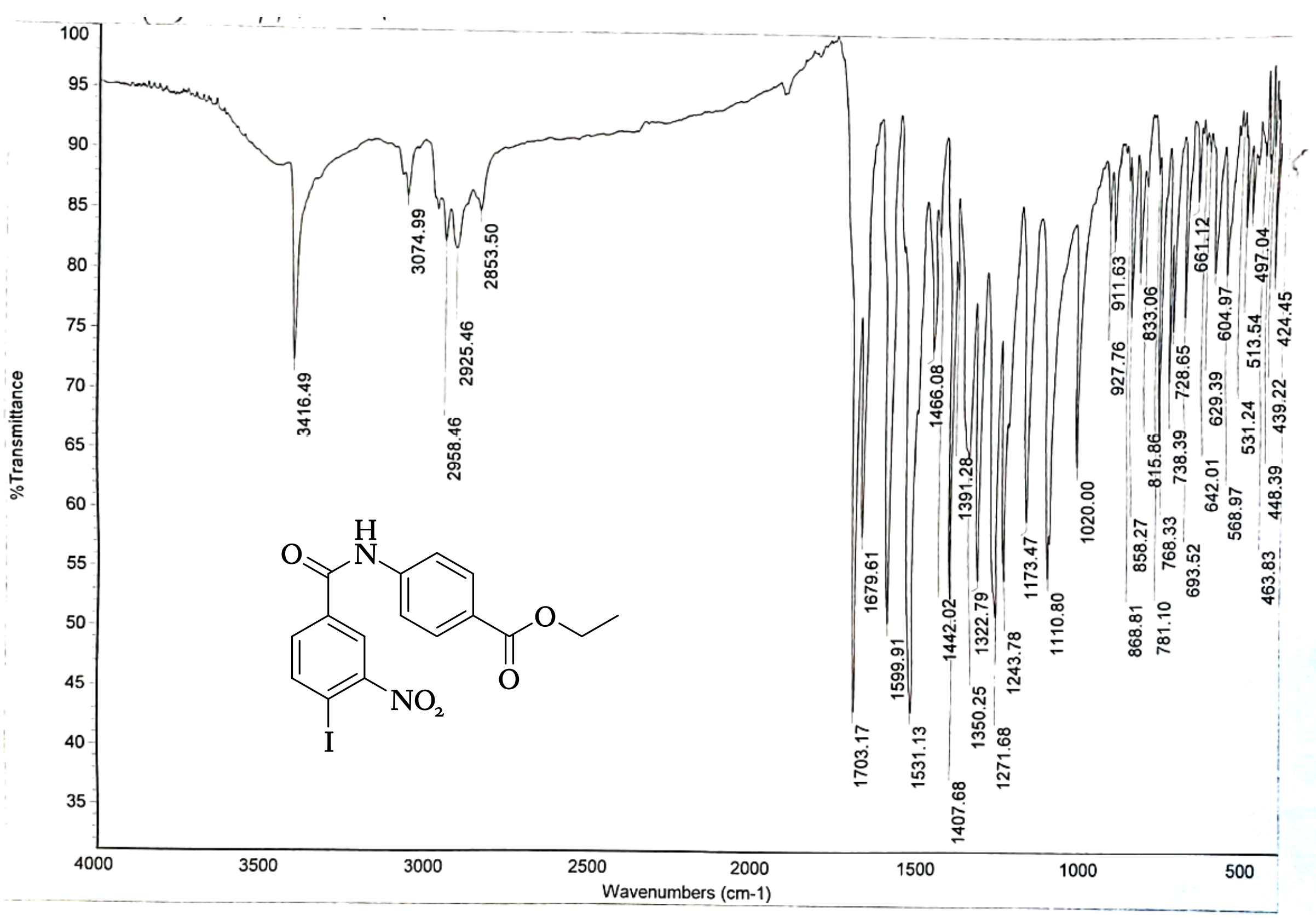

Figure S1: IR spectrum of compound **5a**

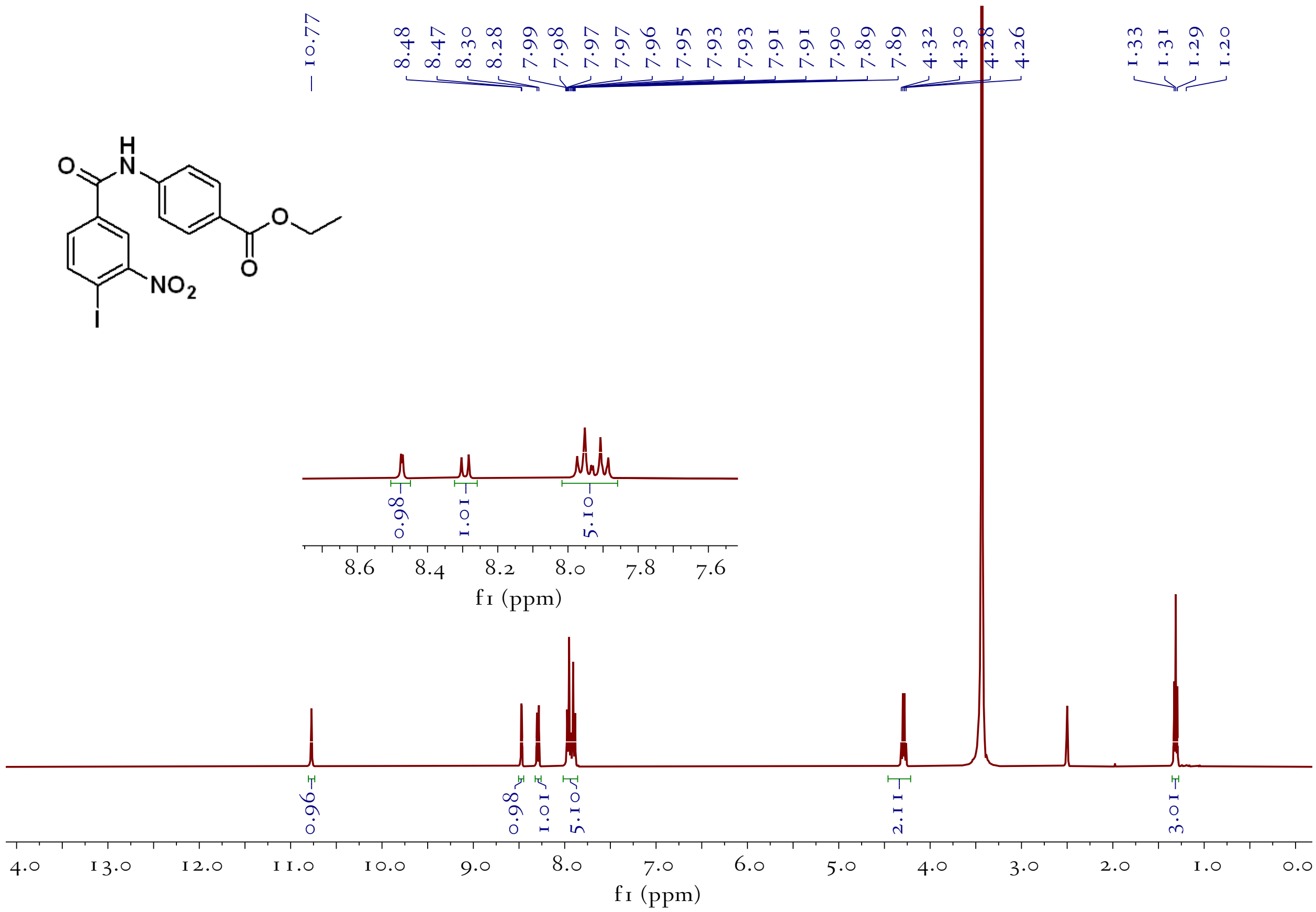

Figure S2: ^{1}H NMR (400 MHz, DMSO-d_6) of compound **5a**

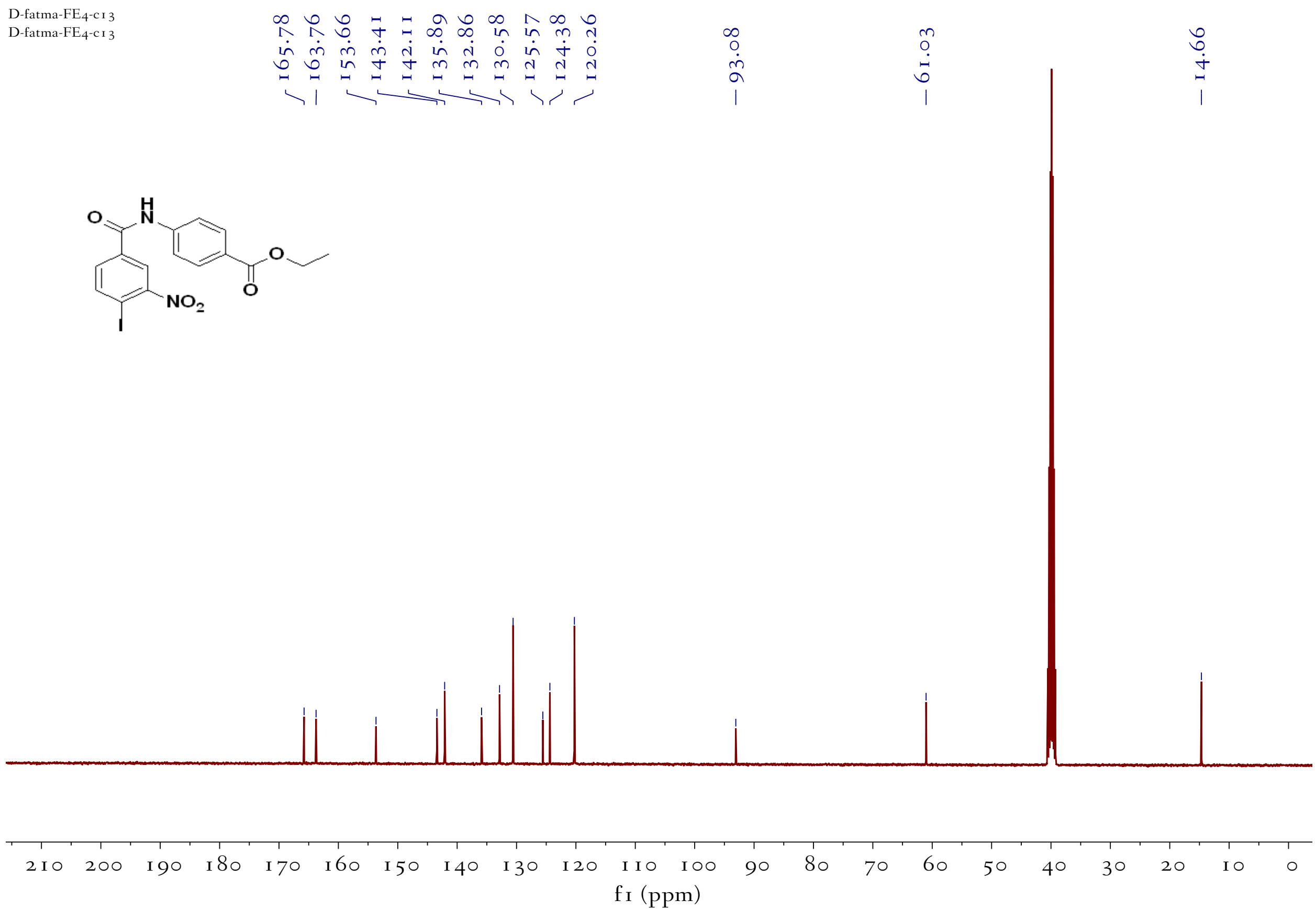

Figure S3: ^{13}C NMR (100 MHz, DMSO-d_6) of compound **5a**

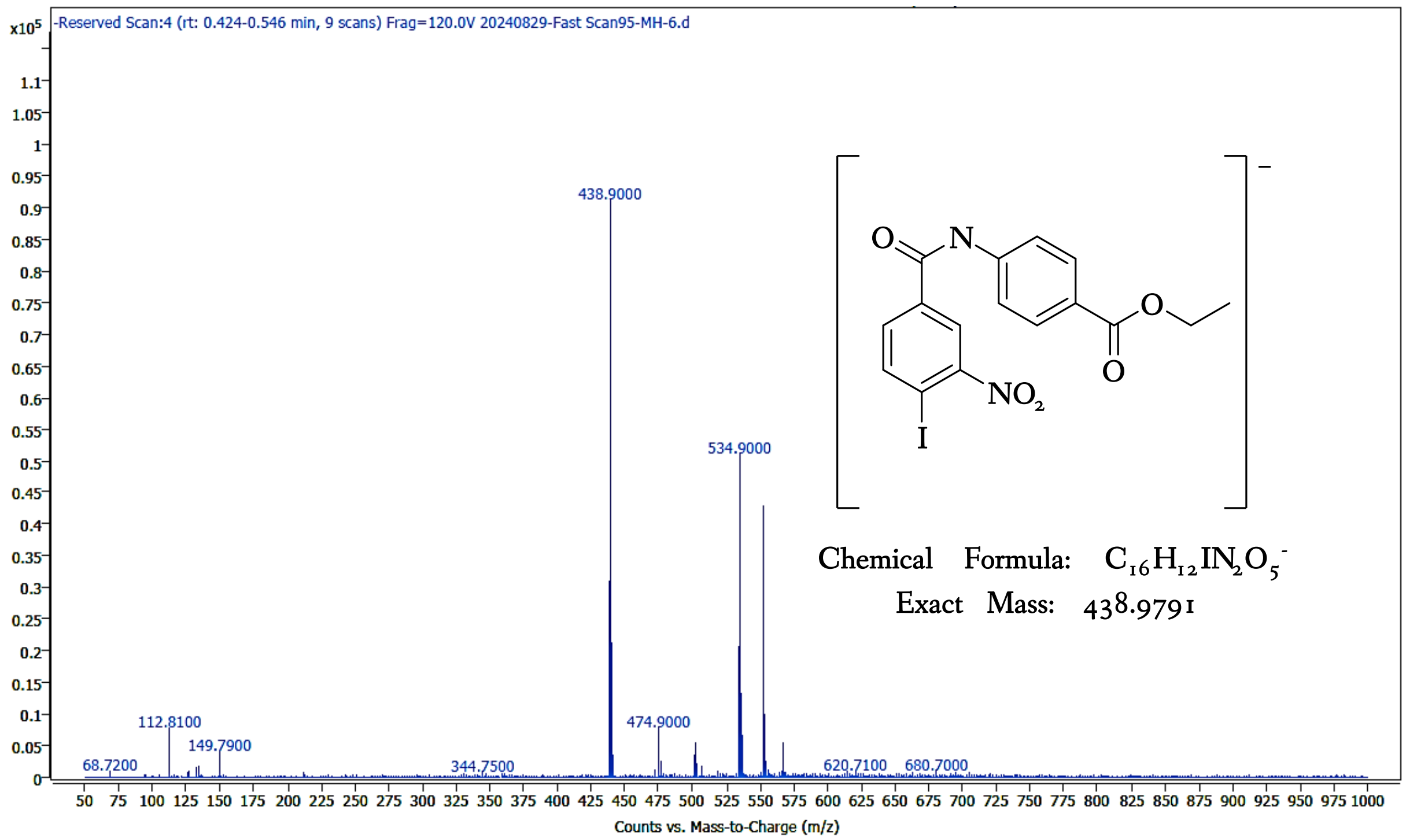

Figure S4: HRMS-ESI Spectrum of compound **5a**

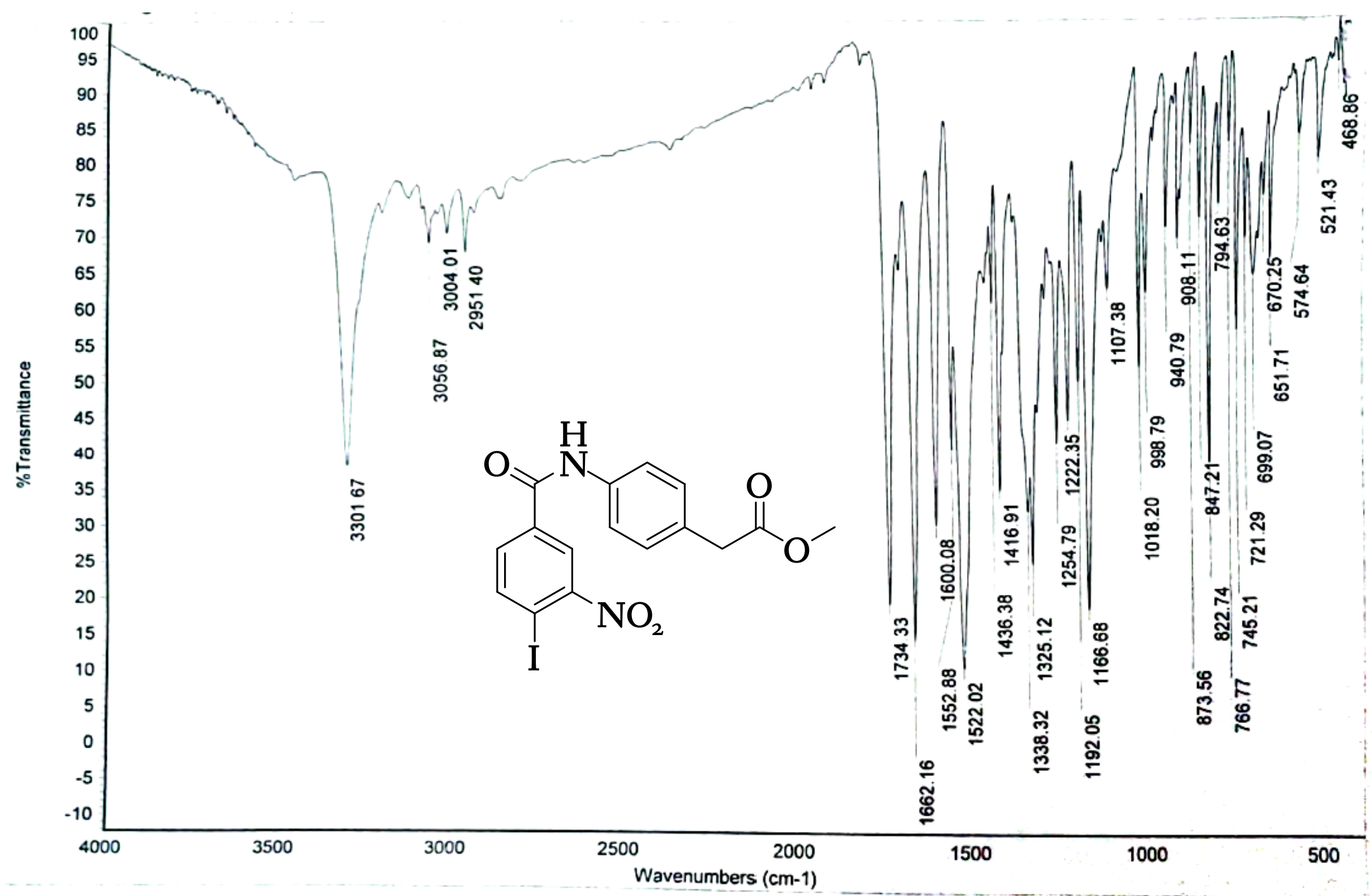

Figure S5: IR spectrum of compound **5b**

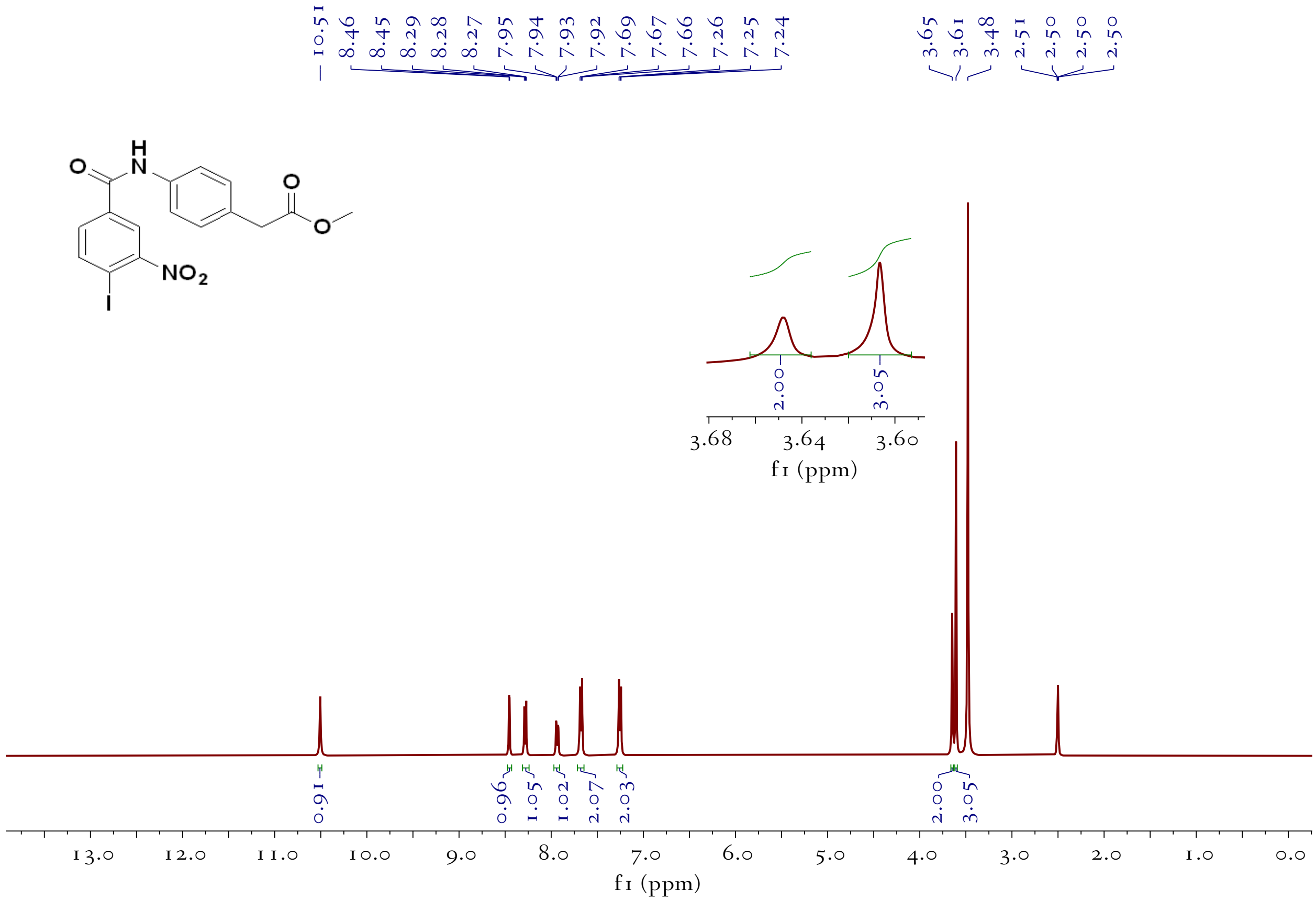

Figure S6: ^{1}H NMR (400 MHz, DMSO-d_6) of compound **5b**

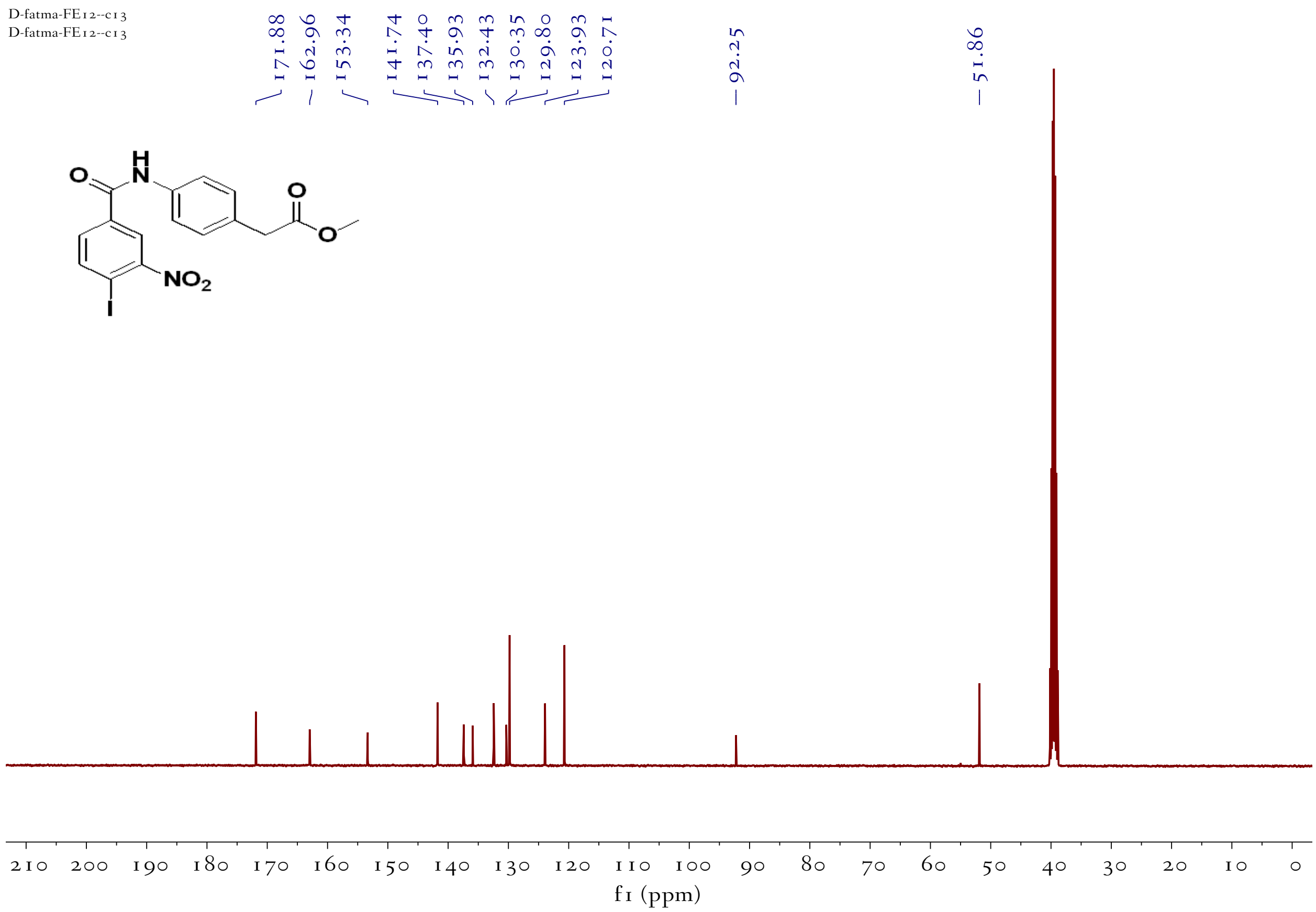

Figure S7: ^{13}C NMR (100 MHz, DMSO-d_6) of compound **5b**

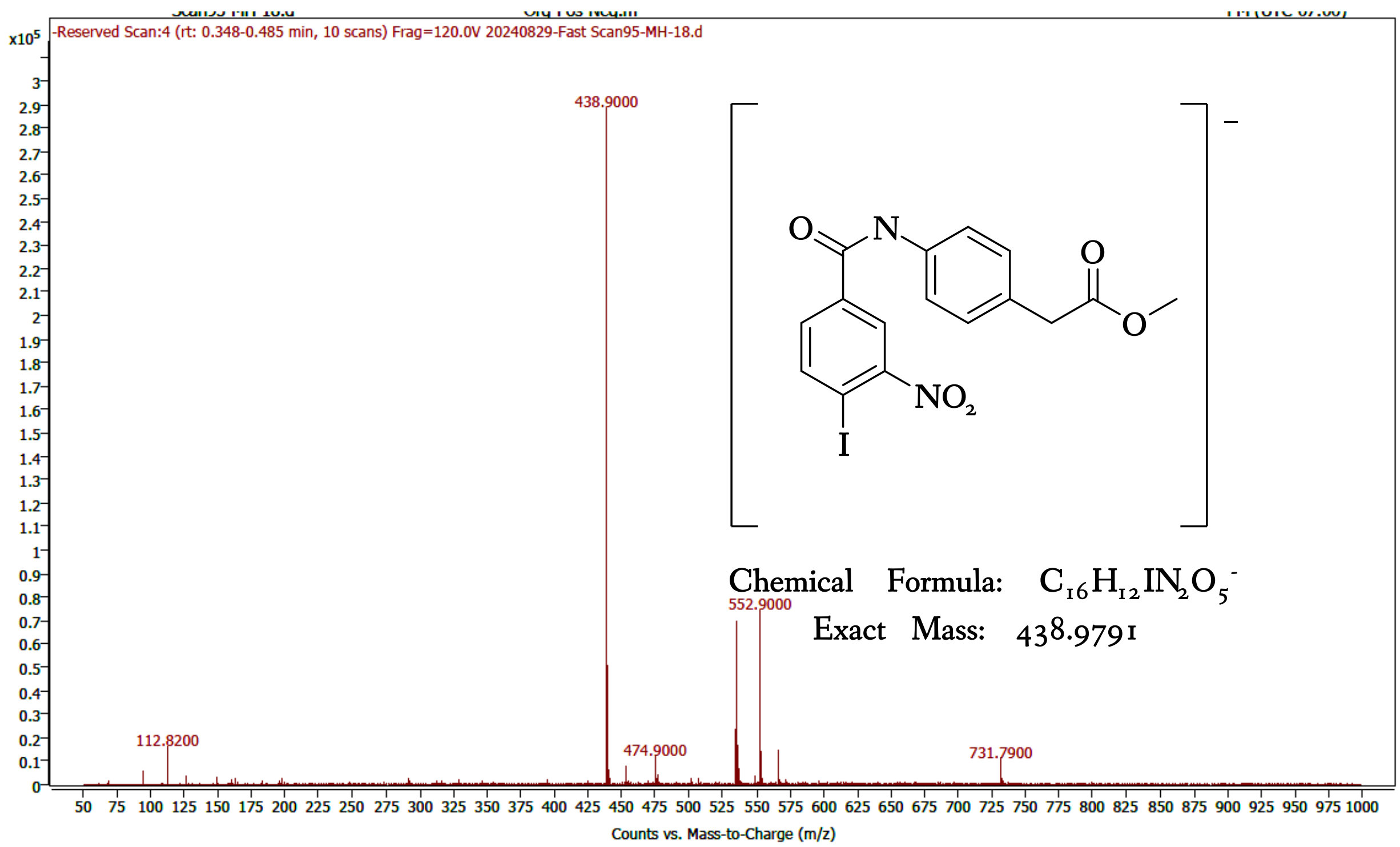

Figure S8: HRMS-ESI Spectrum of compound **5b**

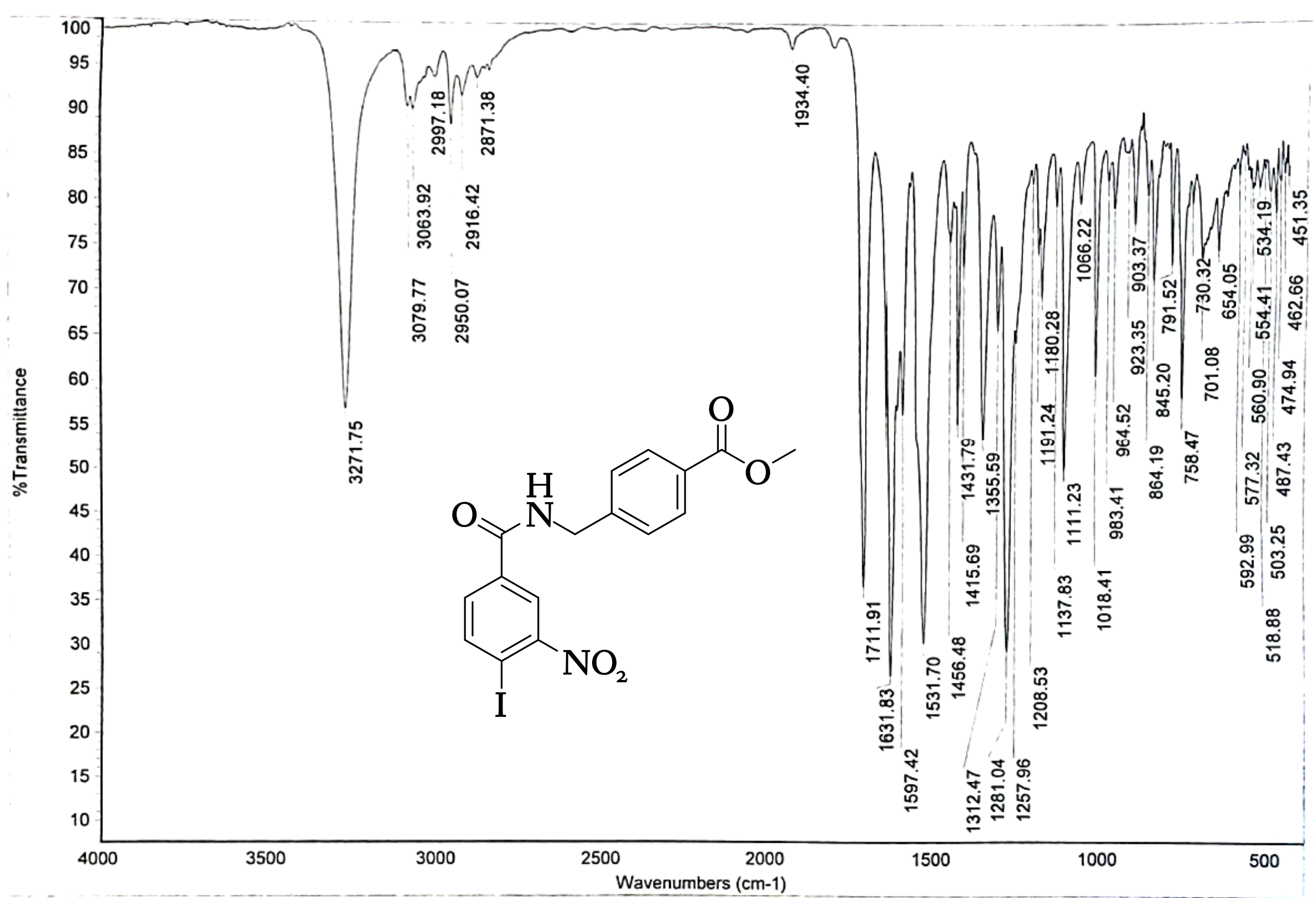

Figure S9: IR spectrum of compound **5c**

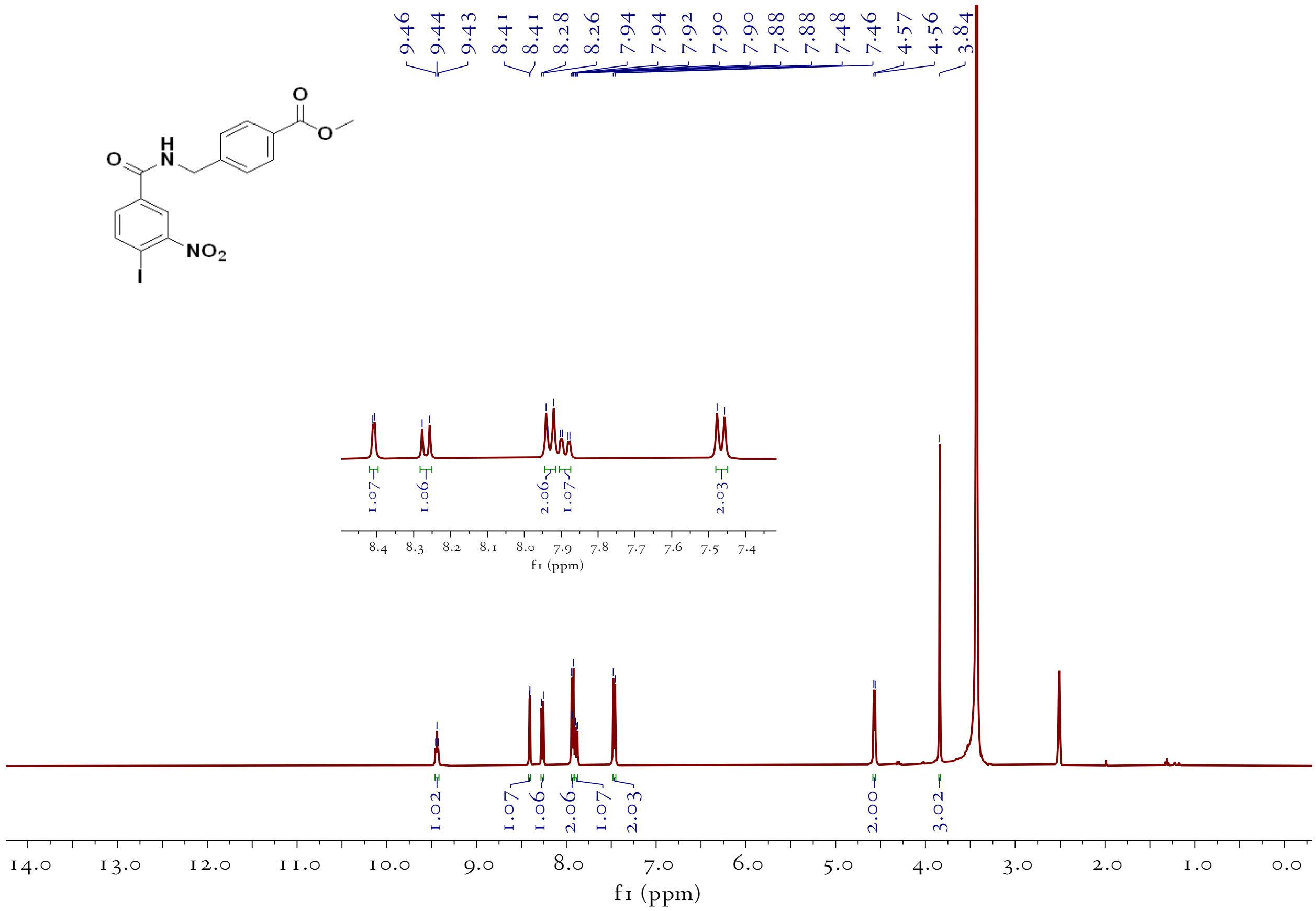

Figure S10: ^{1}H NMR (400 MHz, DMSO-d_6) of compound **5c**

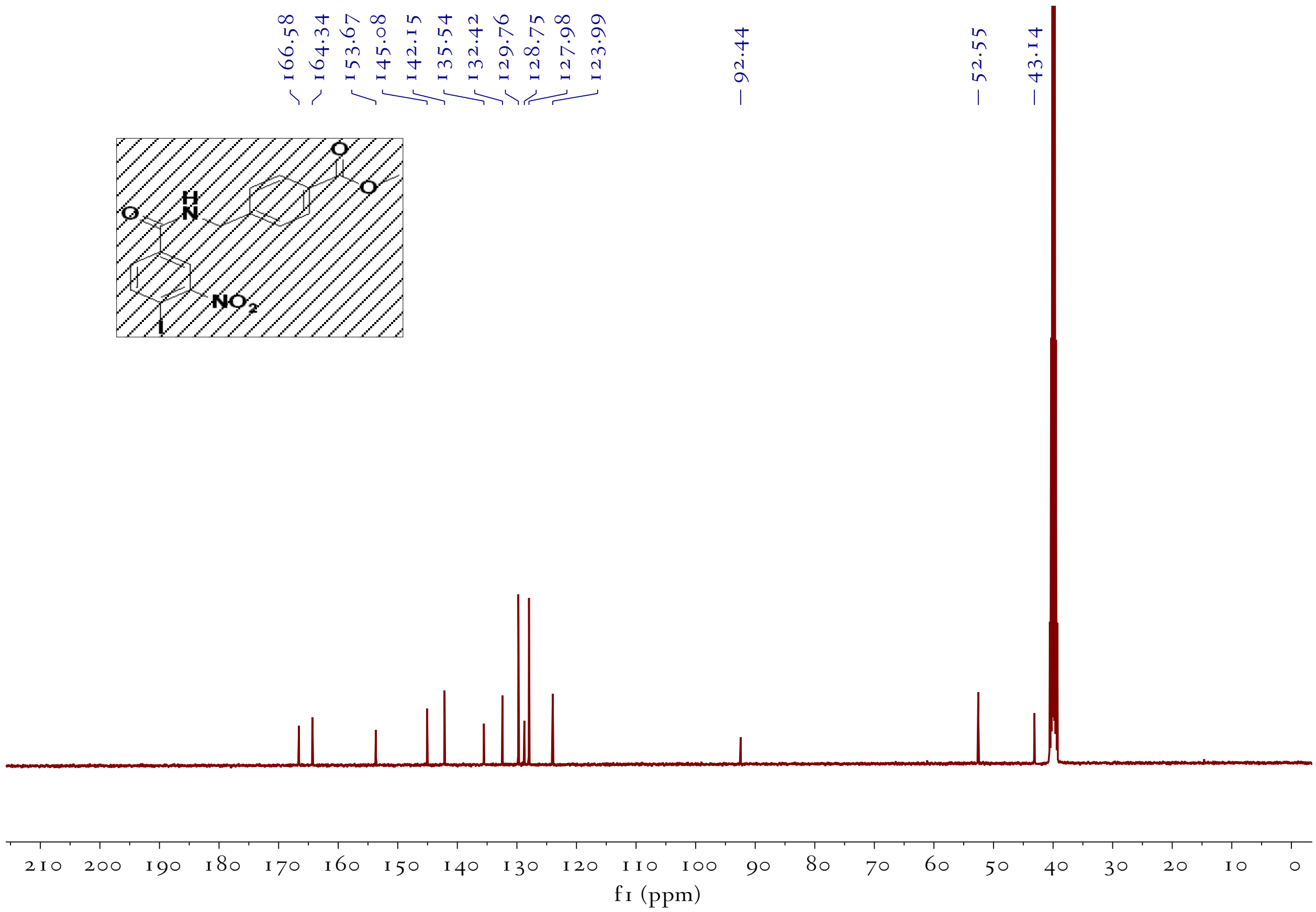

Figure S11: ^{13}C NMR (100 MHz, DMSO-d_6) of compound **5c**

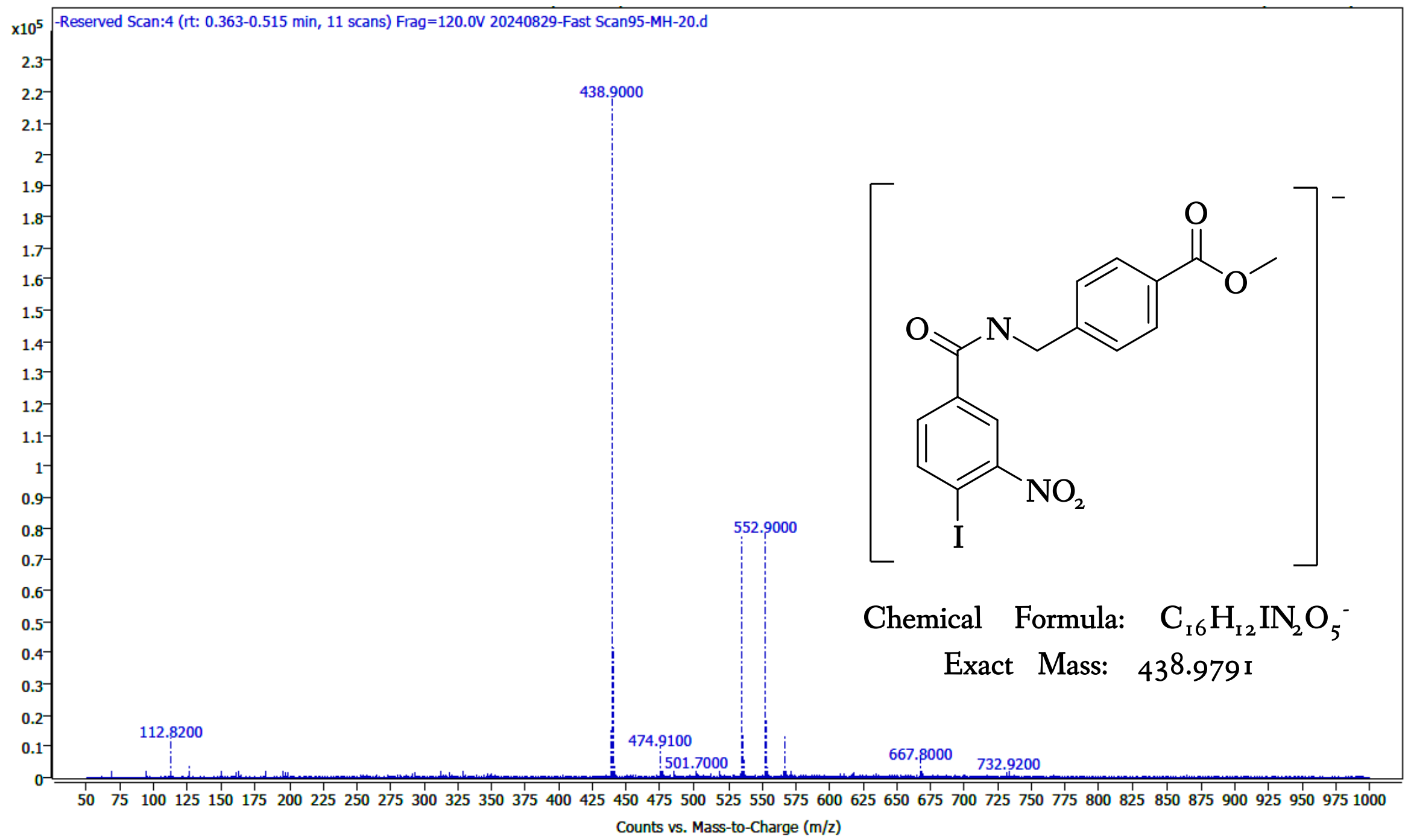

Figure S12: HRMS-ESI Spectrum of compound **5c**

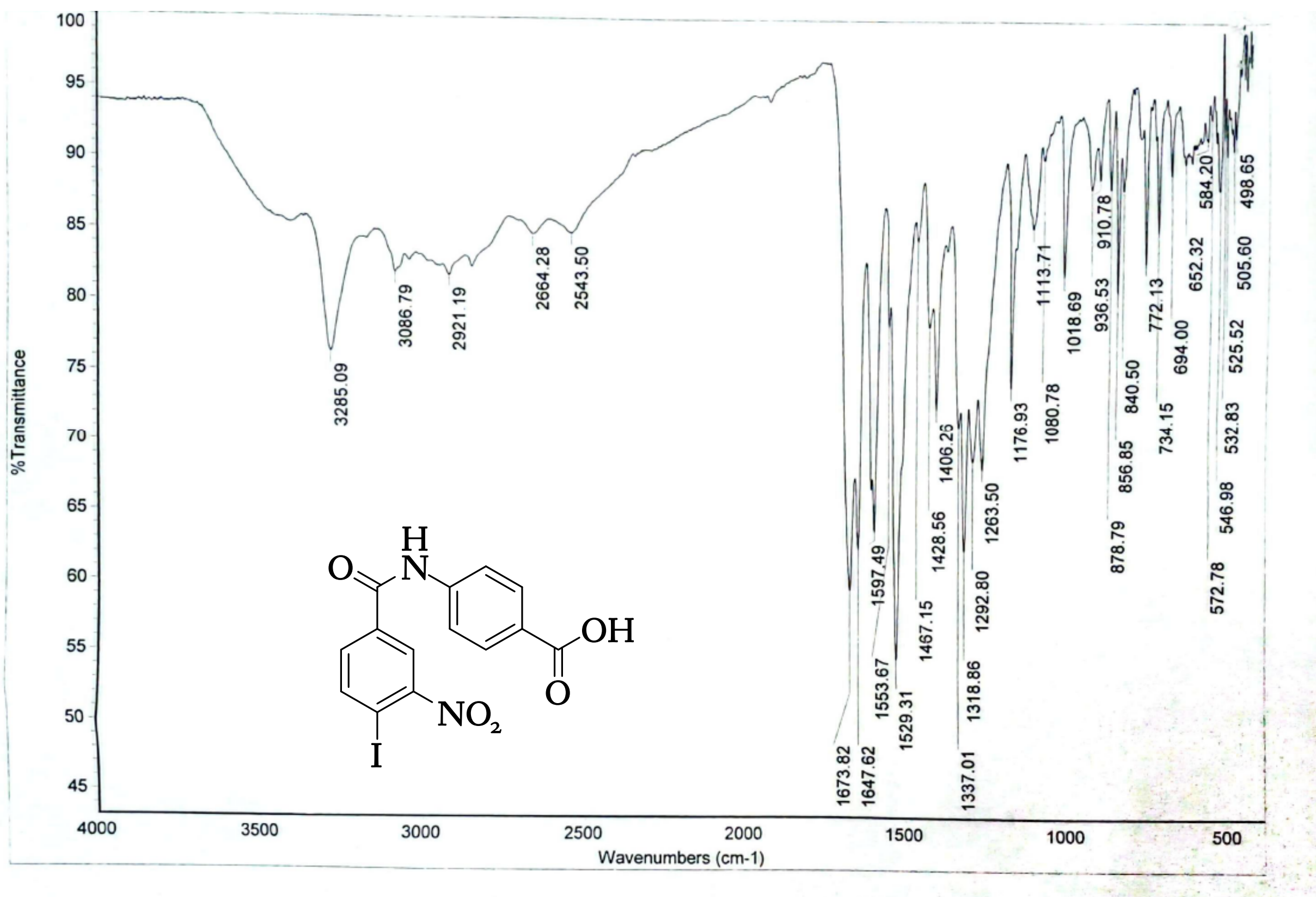

Figure S13: IR spectrum of compound **6a**

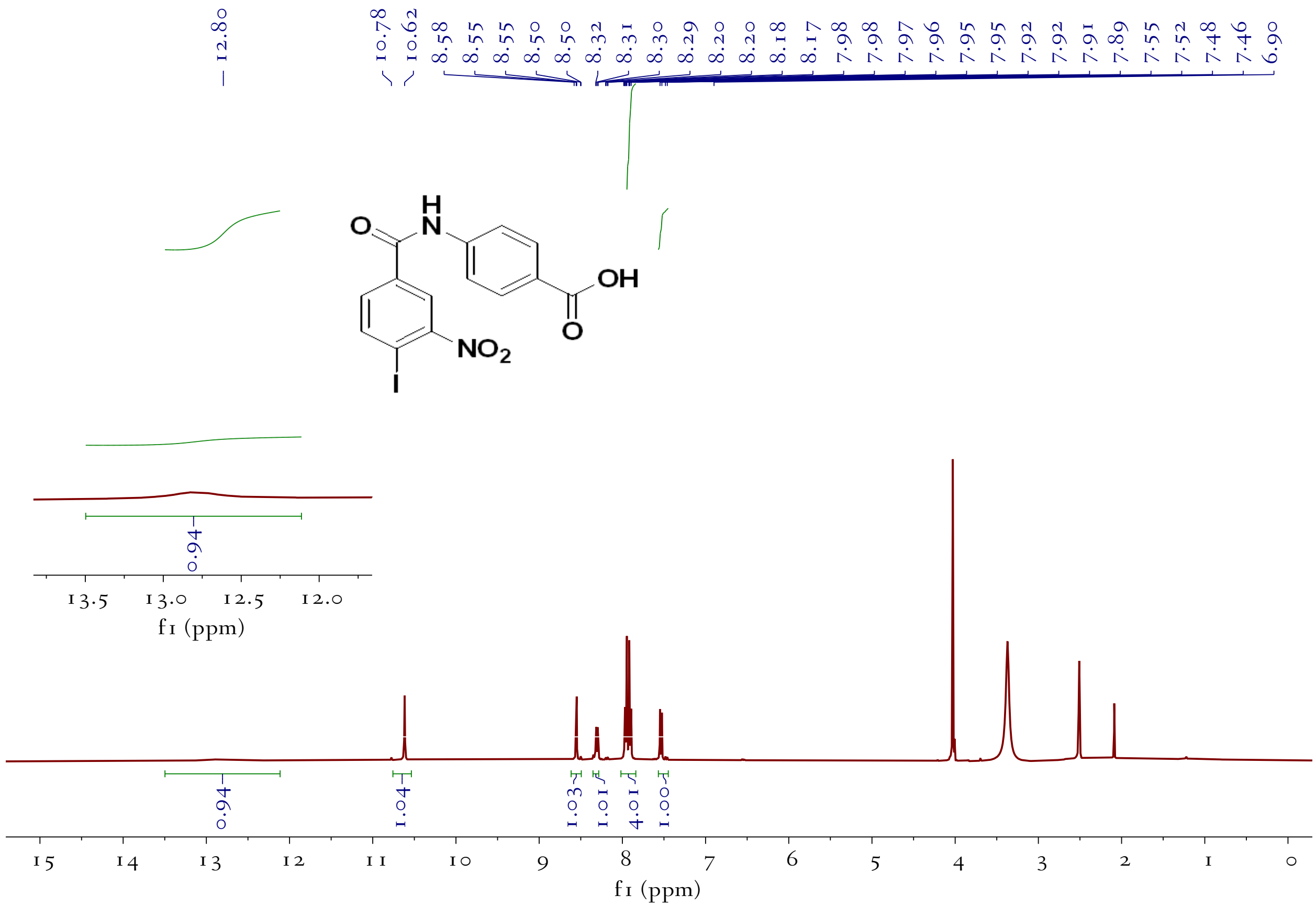

Figure S14: ^{1}H NMR (400 MHz, DMSO-d_6) of compound **6a**

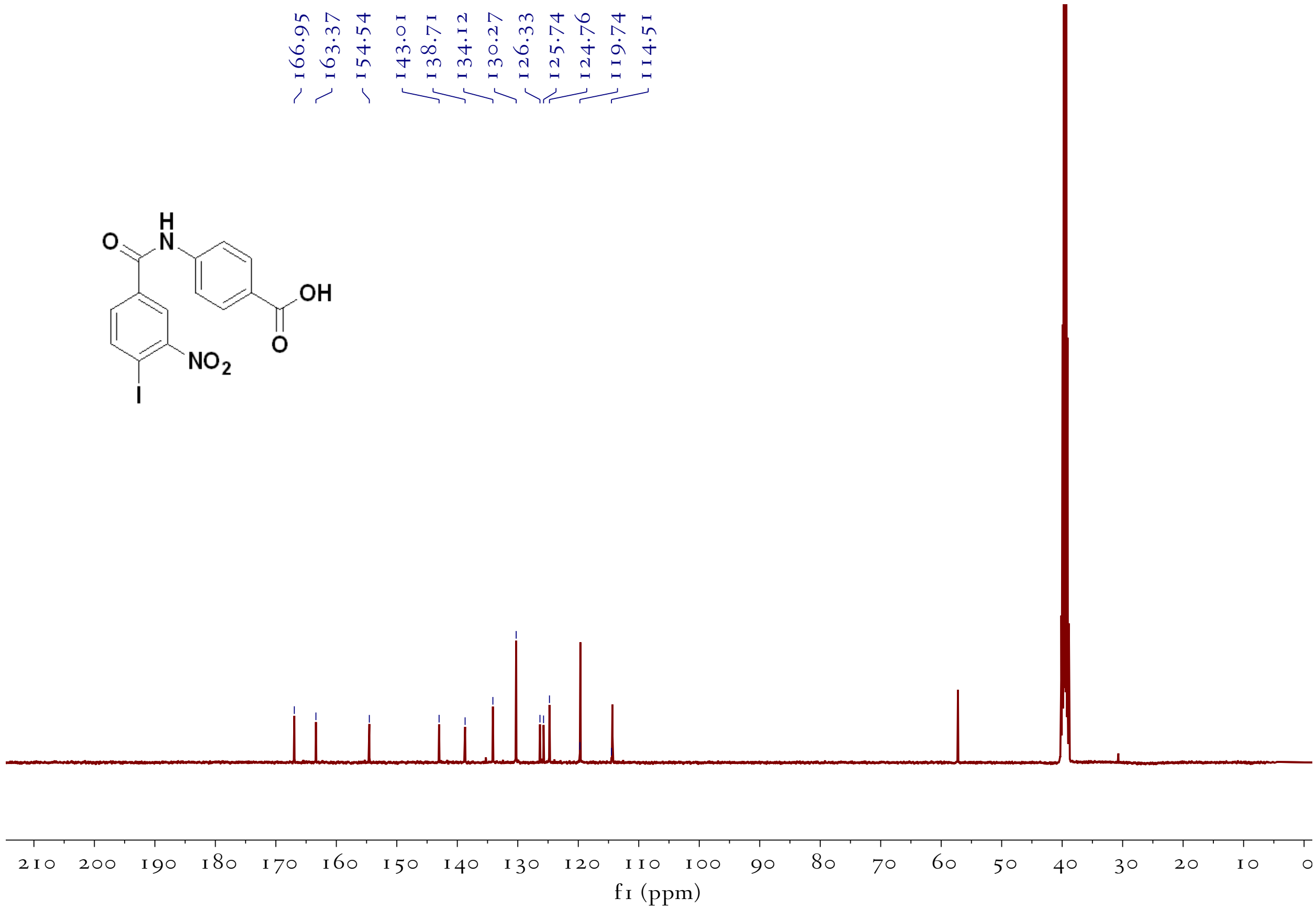

Figure S15: ^{13}C NMR (100 MHz, DMSO-d_6) of compound **6a**

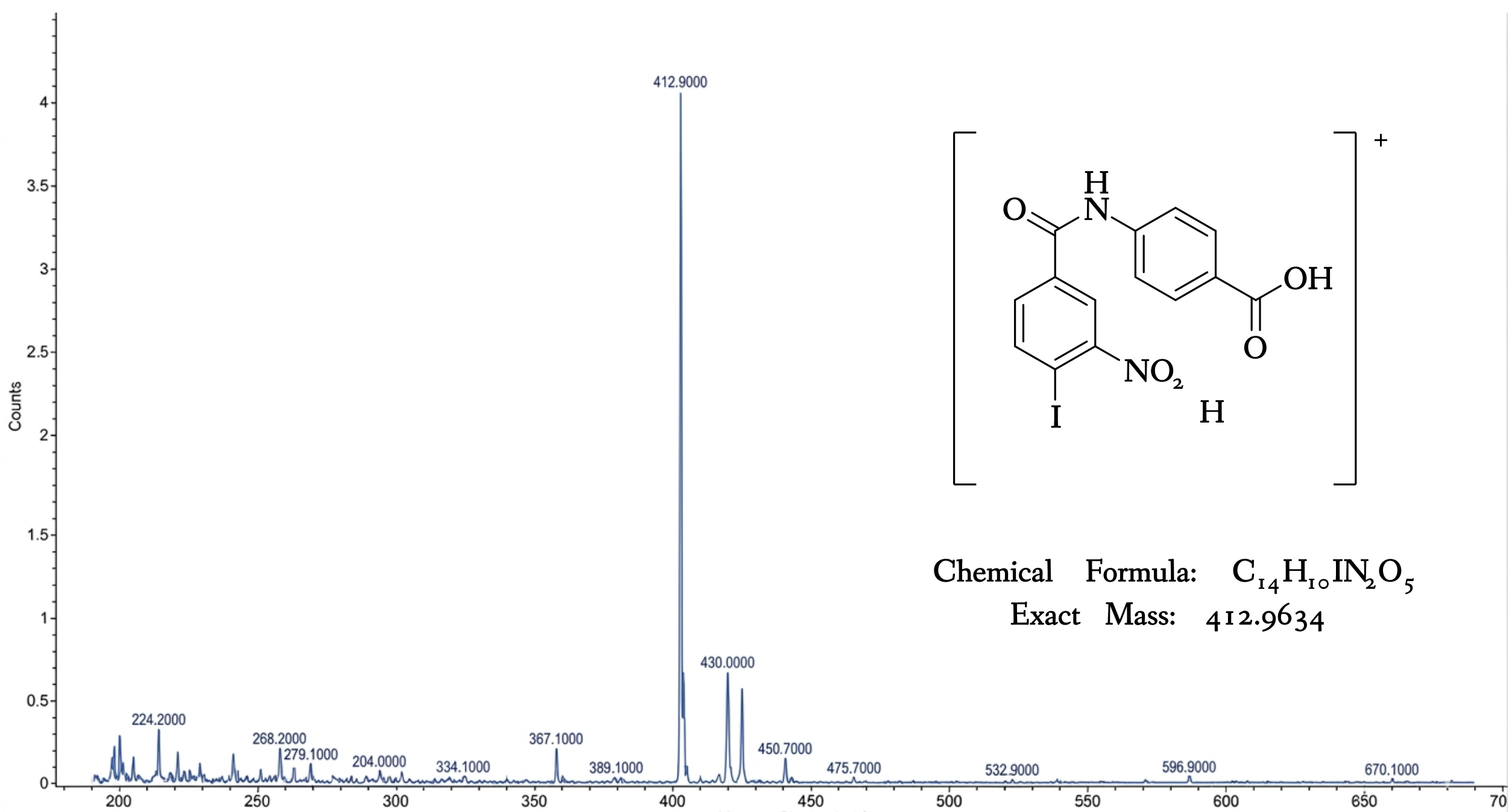

Figure S16: HRMS-ESI Spectrum of compound **6a**

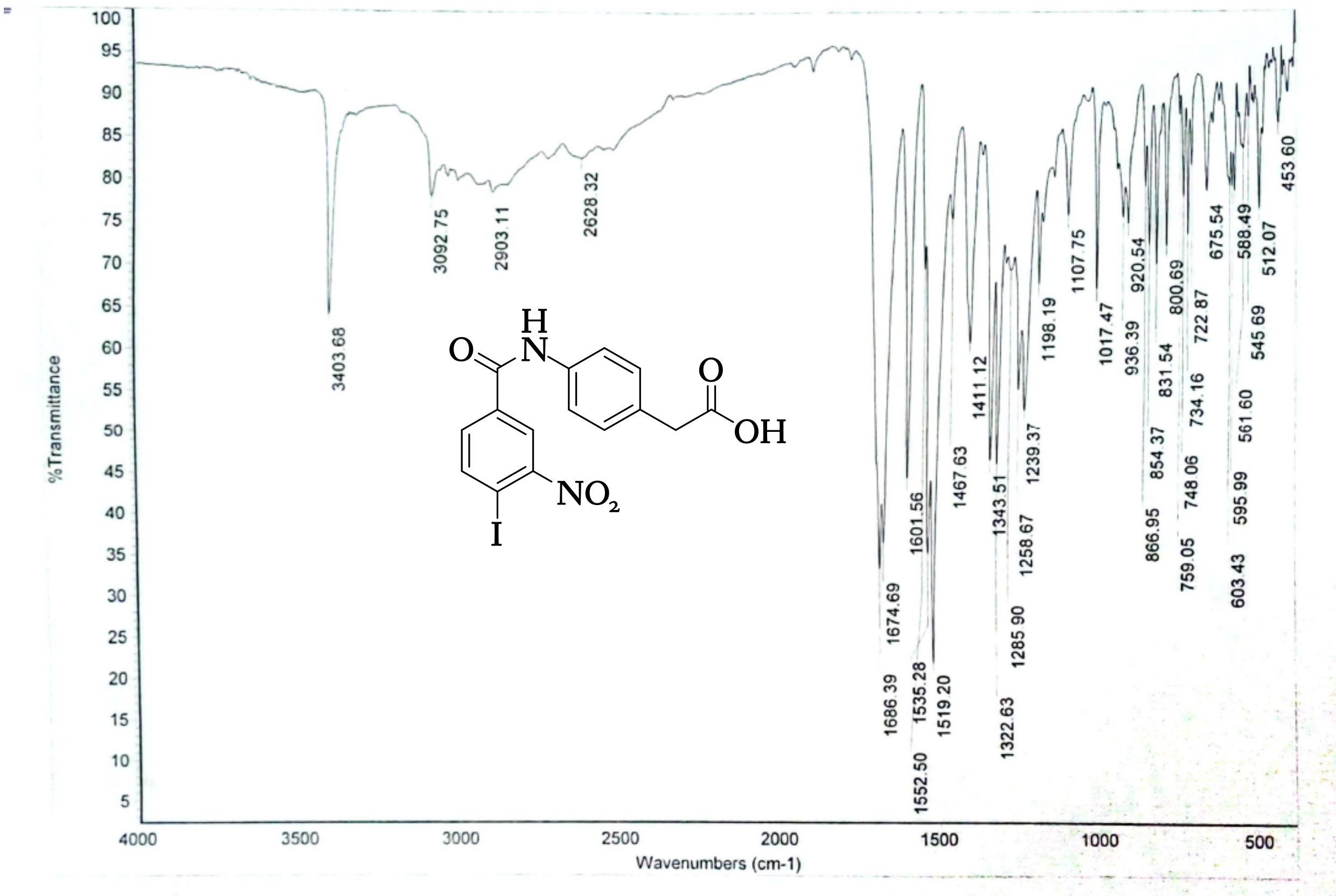

Figure S17: IR spectrum of compound **6b**

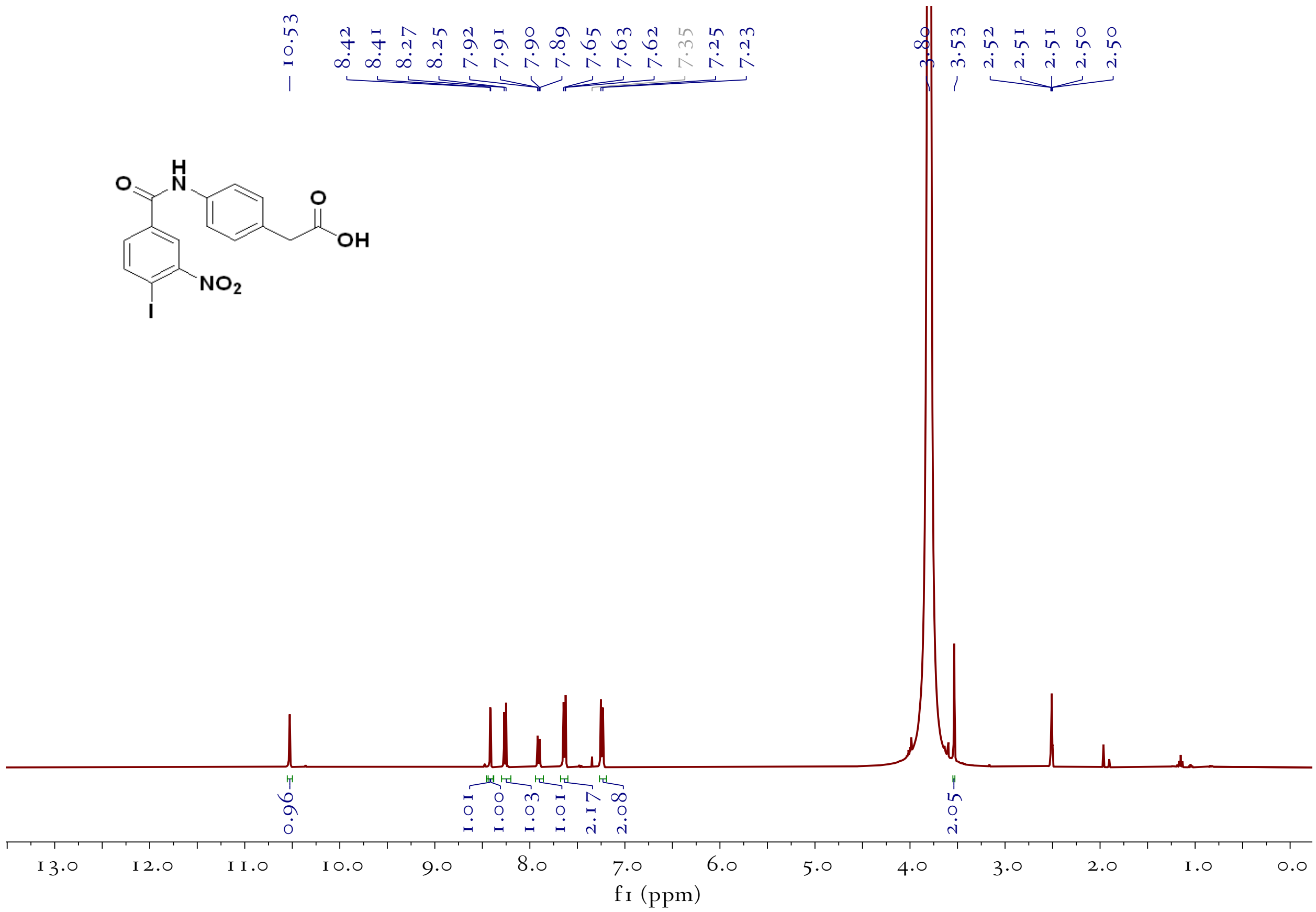

Figure S18: ^{1}H NMR (400 MHz, DMSO-d_6) of compound **6b**

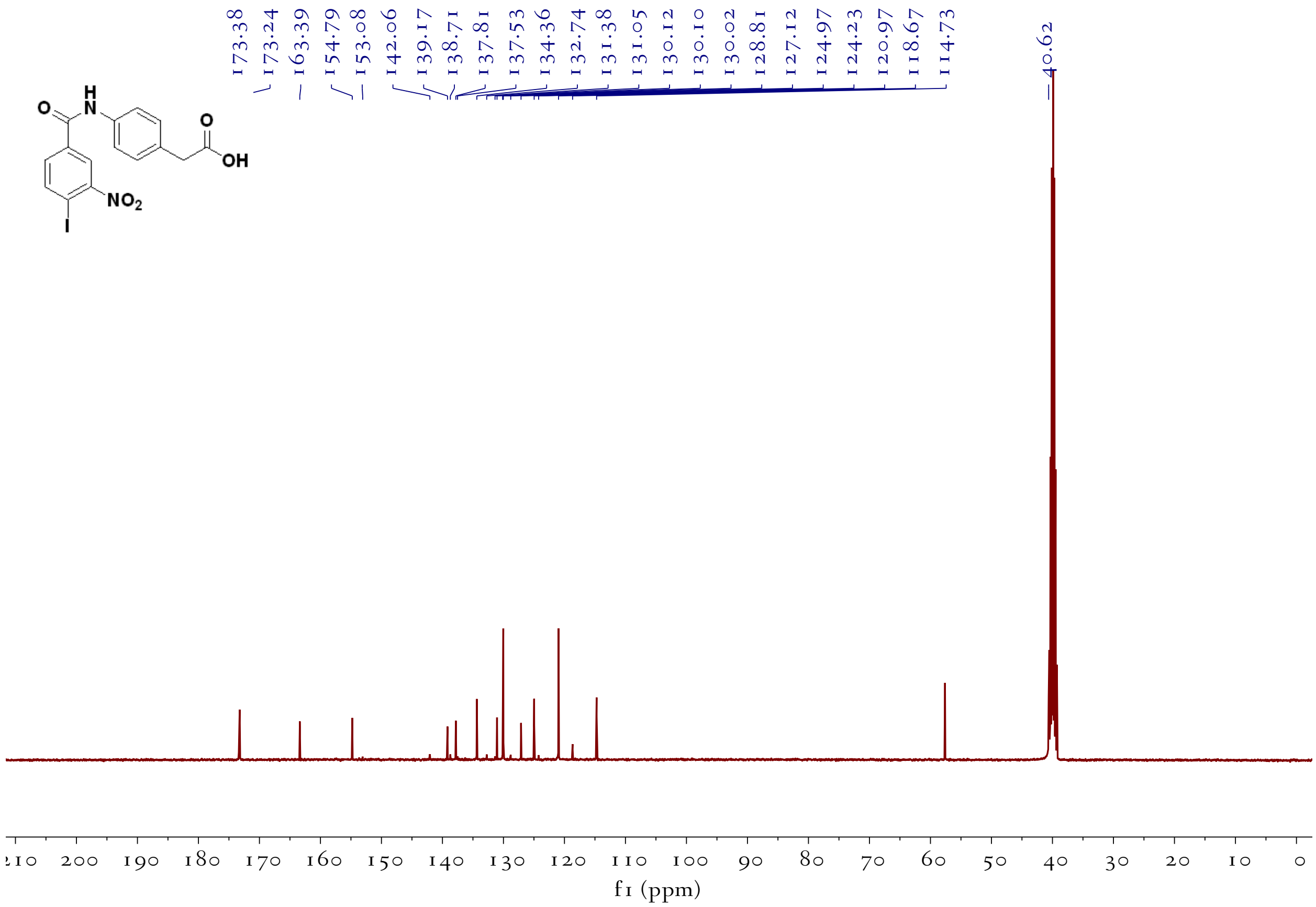

Figure S19: ^{13}C NMR (100 MHz, DMSO-d_6) of compound **6b**

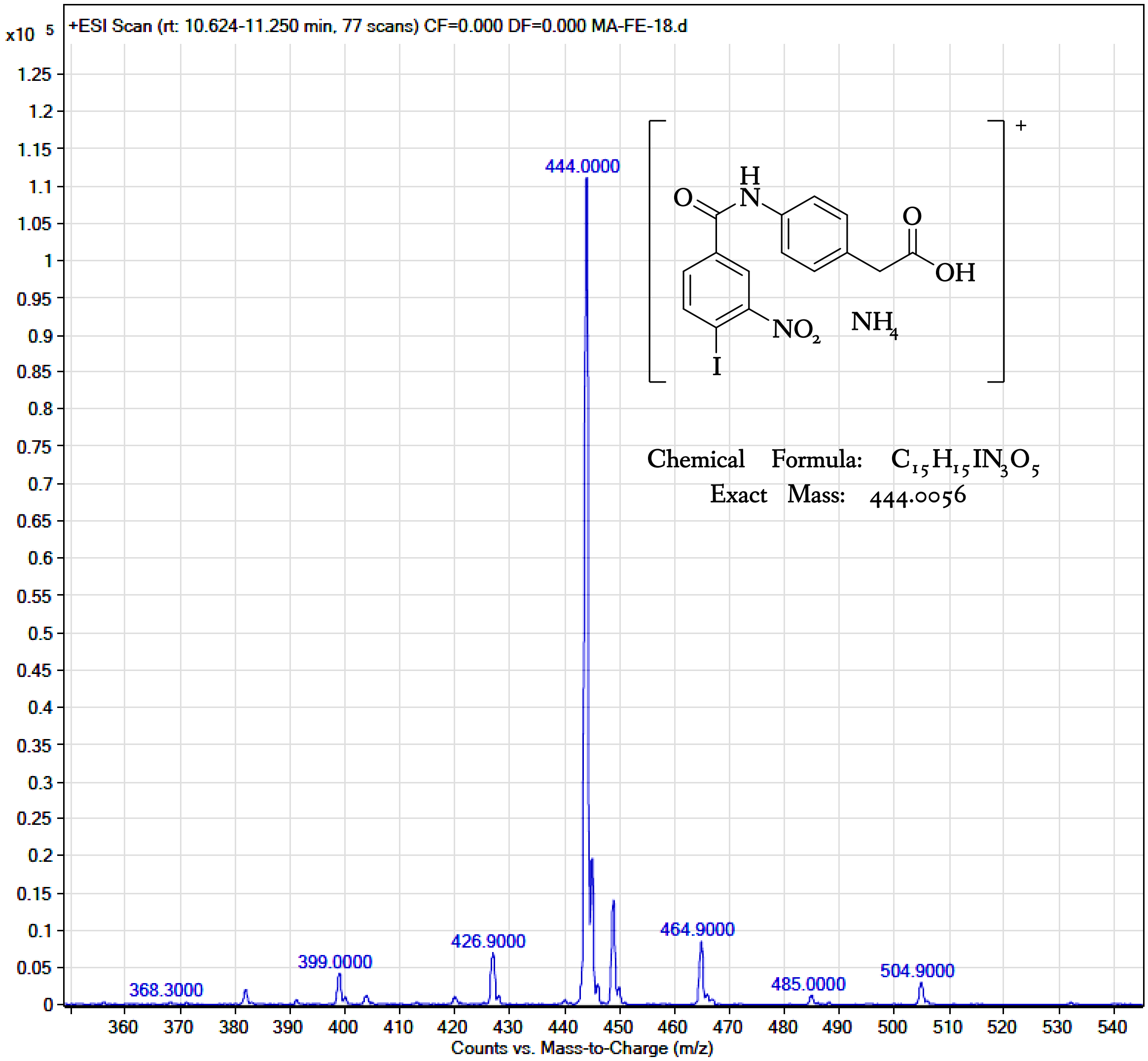

Figure S20: HRMS-ESI Spectrum of compound **6b**

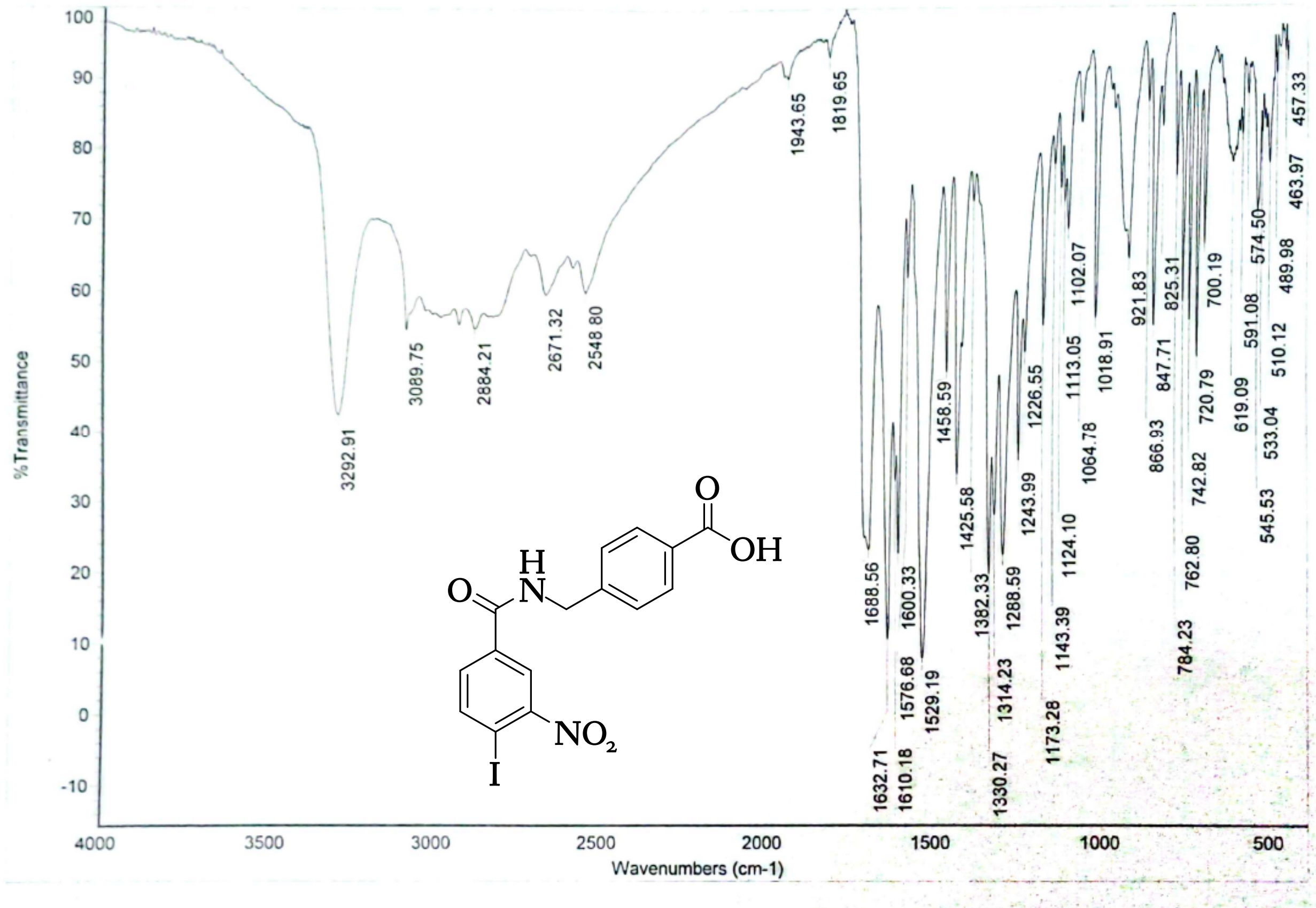

Figure S21: IR spectrum of compound **6c**

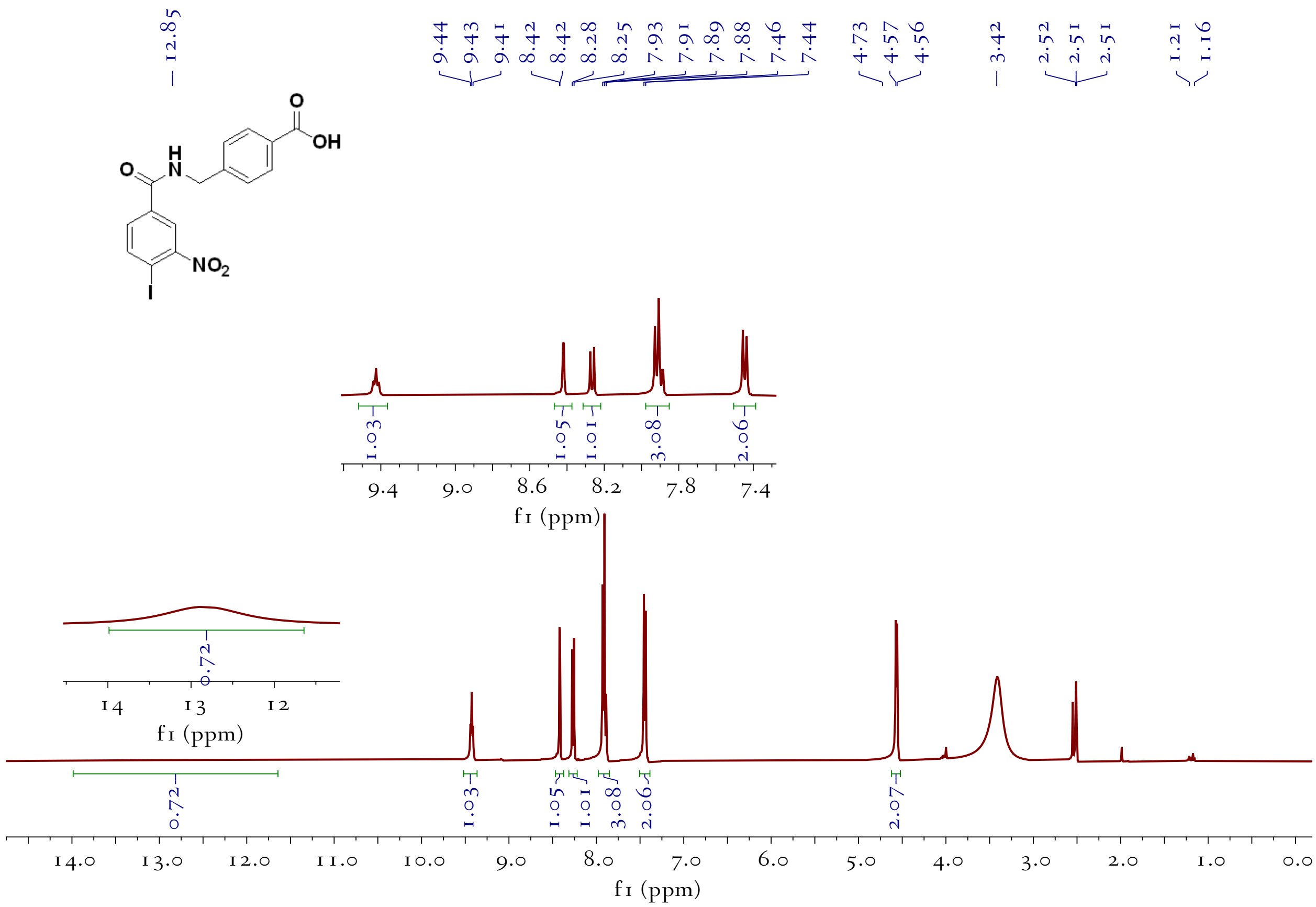

Figure S22: ^{1}H NMR (400 MHz, DMSO-d_6) of compound **6c**

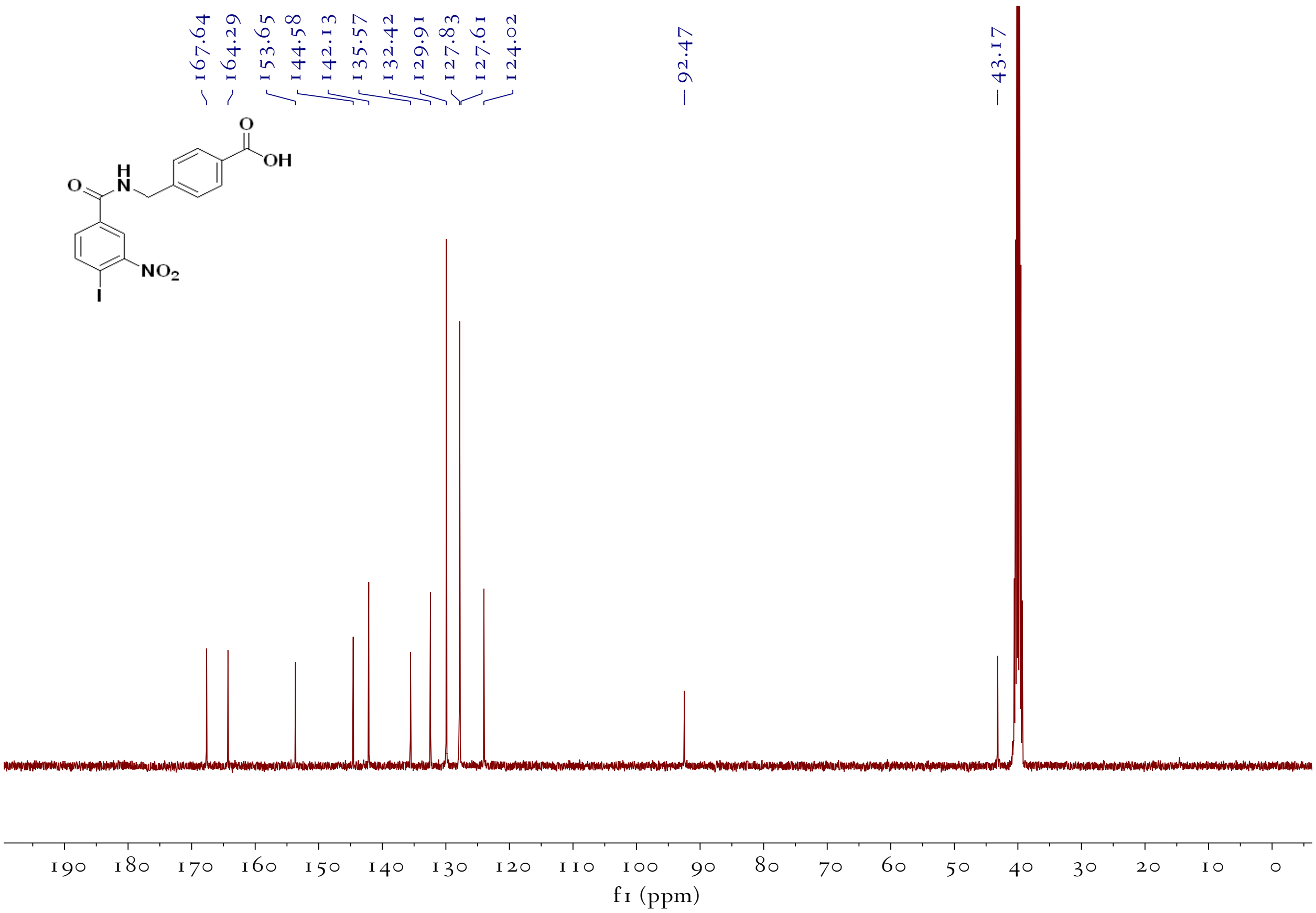

Figure S23: ^{13}C NMR (100 MHz, DMSO-d_6) of compound **6c**

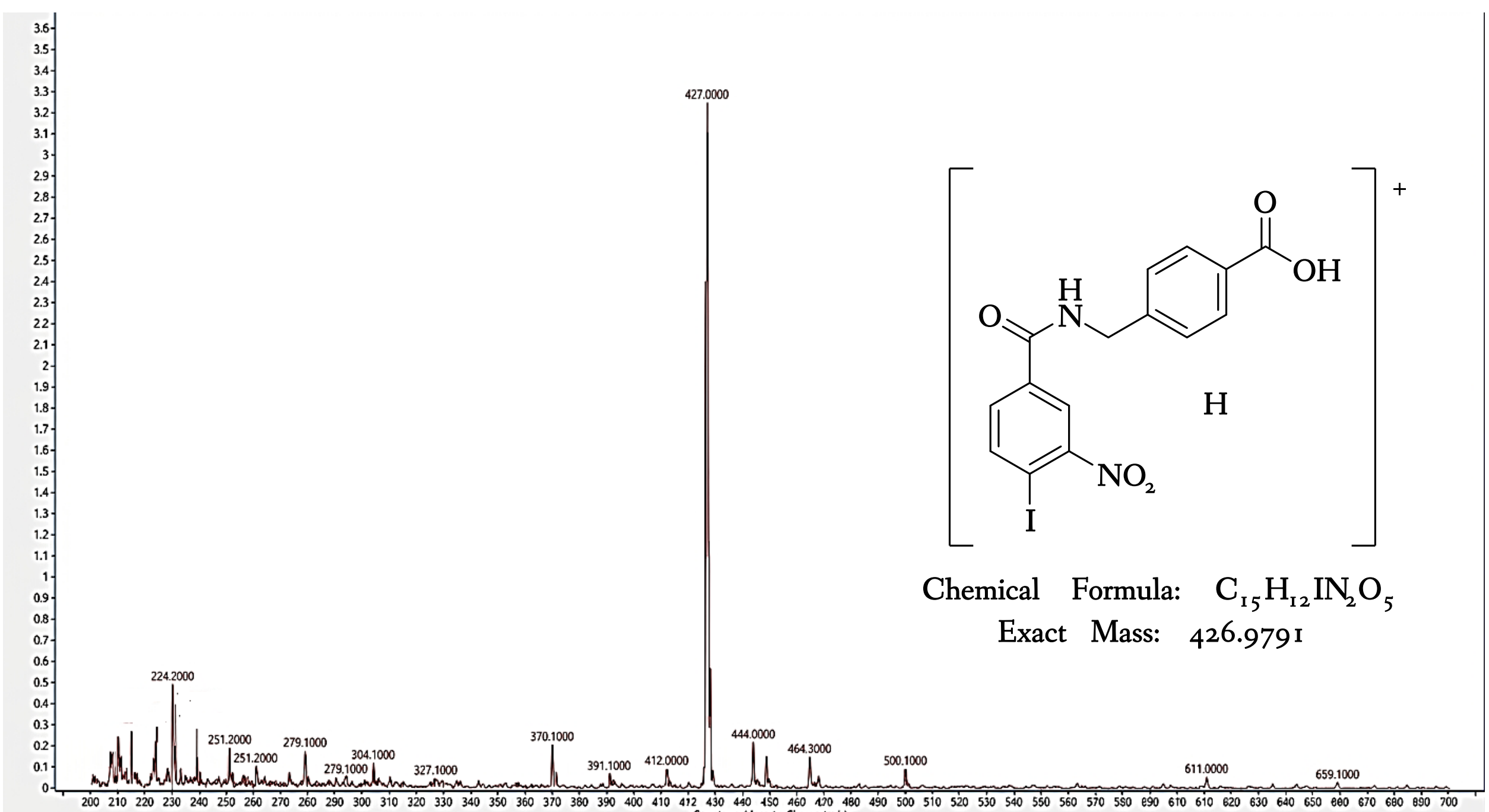

Figure S24: HRMS-ESI Spectrum of compound **6c**

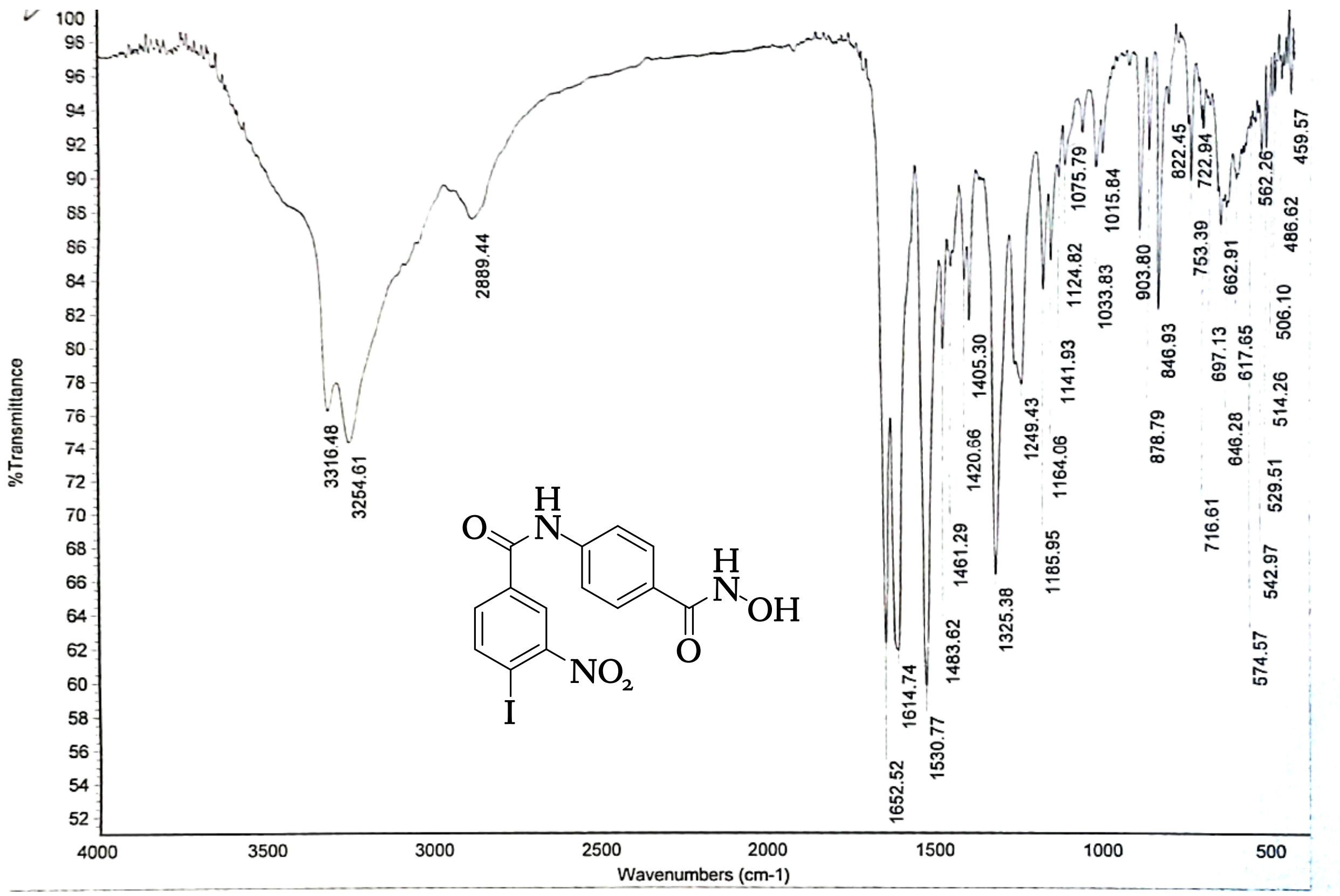

Figure S25: IR spectrum of compound **7a**

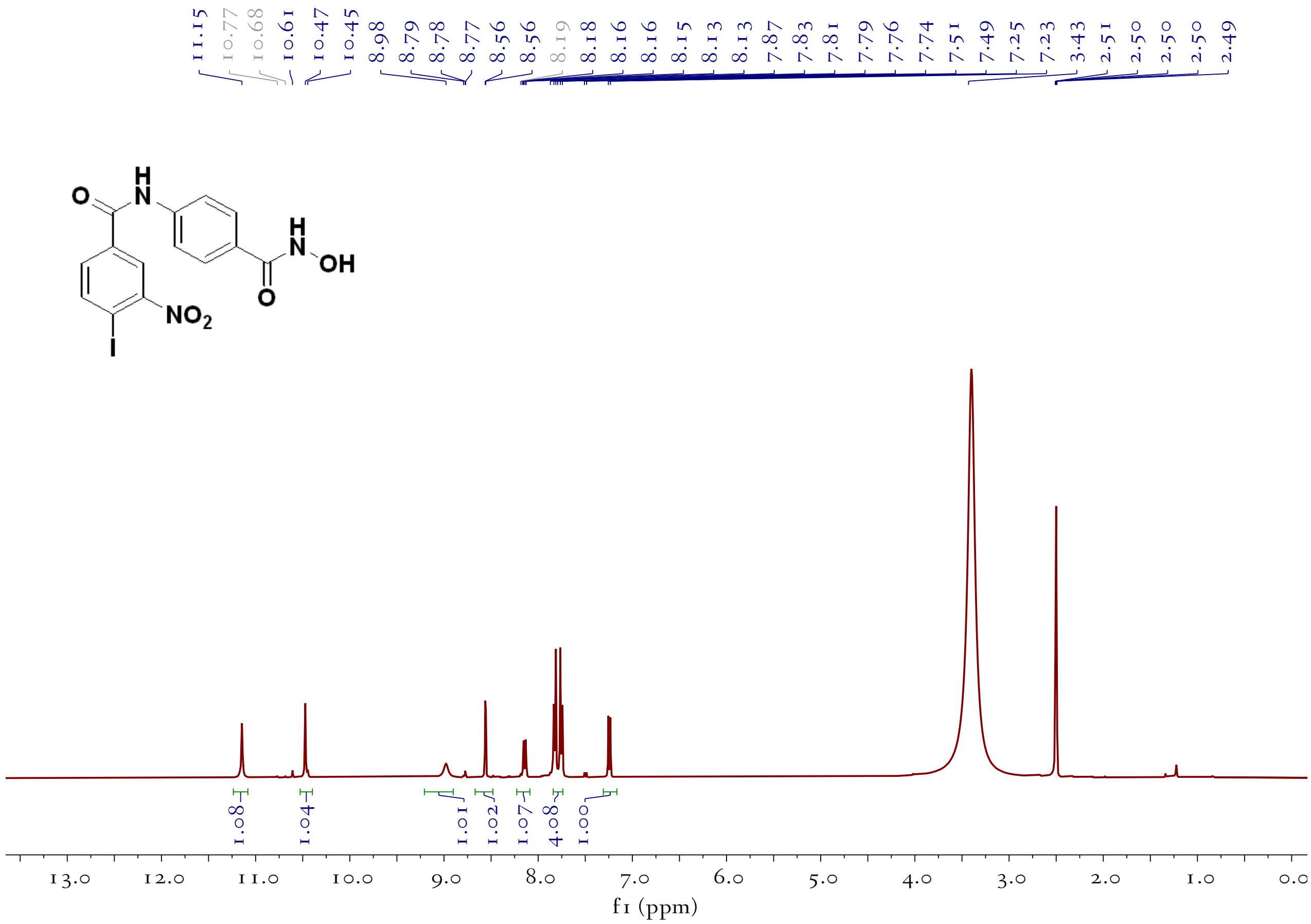

Figure S26: ^{1}H NMR (400 MHz, DMSO-d_6) of compound **7a**

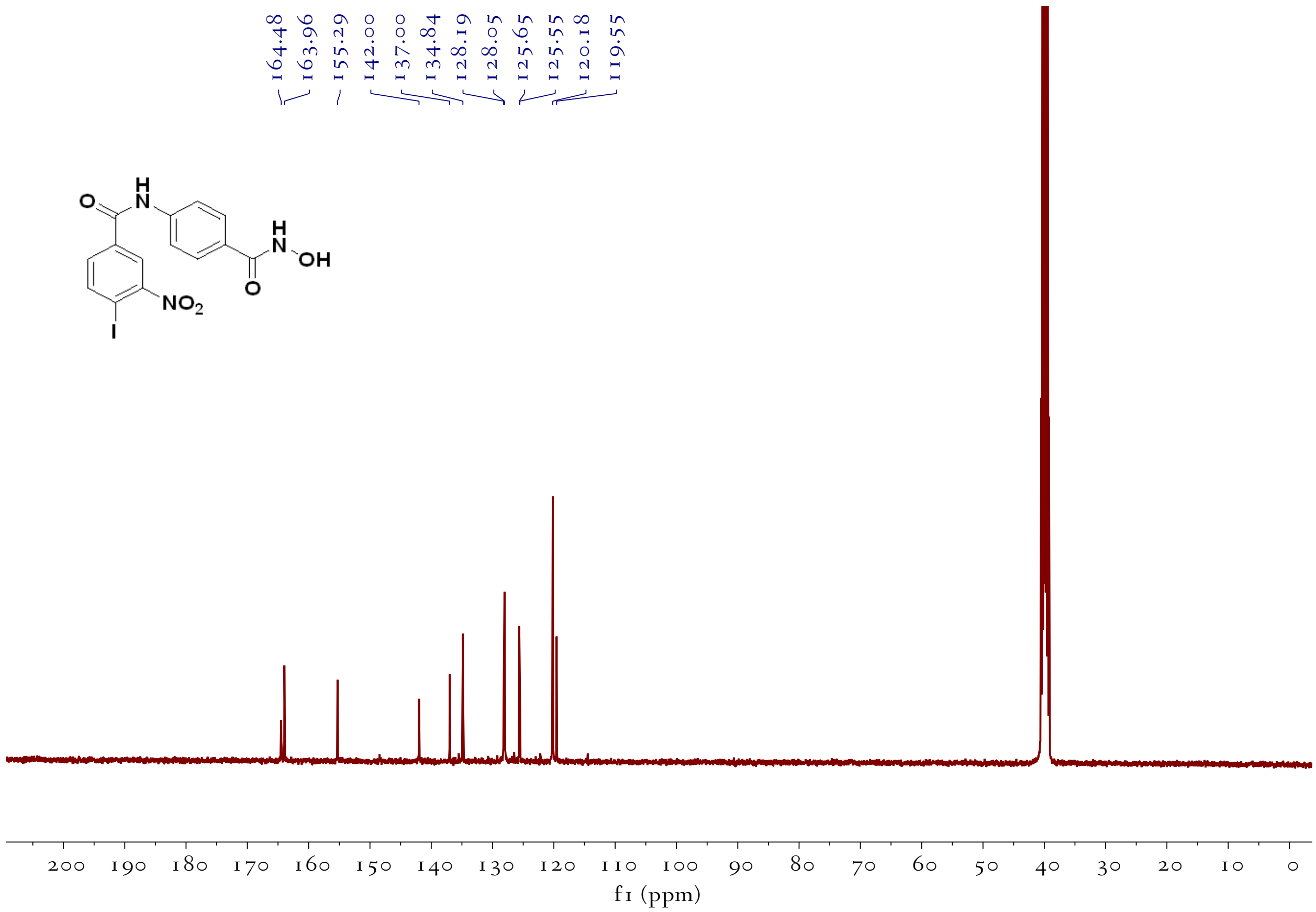

Figure S27: ^{13}C NMR (100 MHz, DMSO-d_6) of compound **7a**

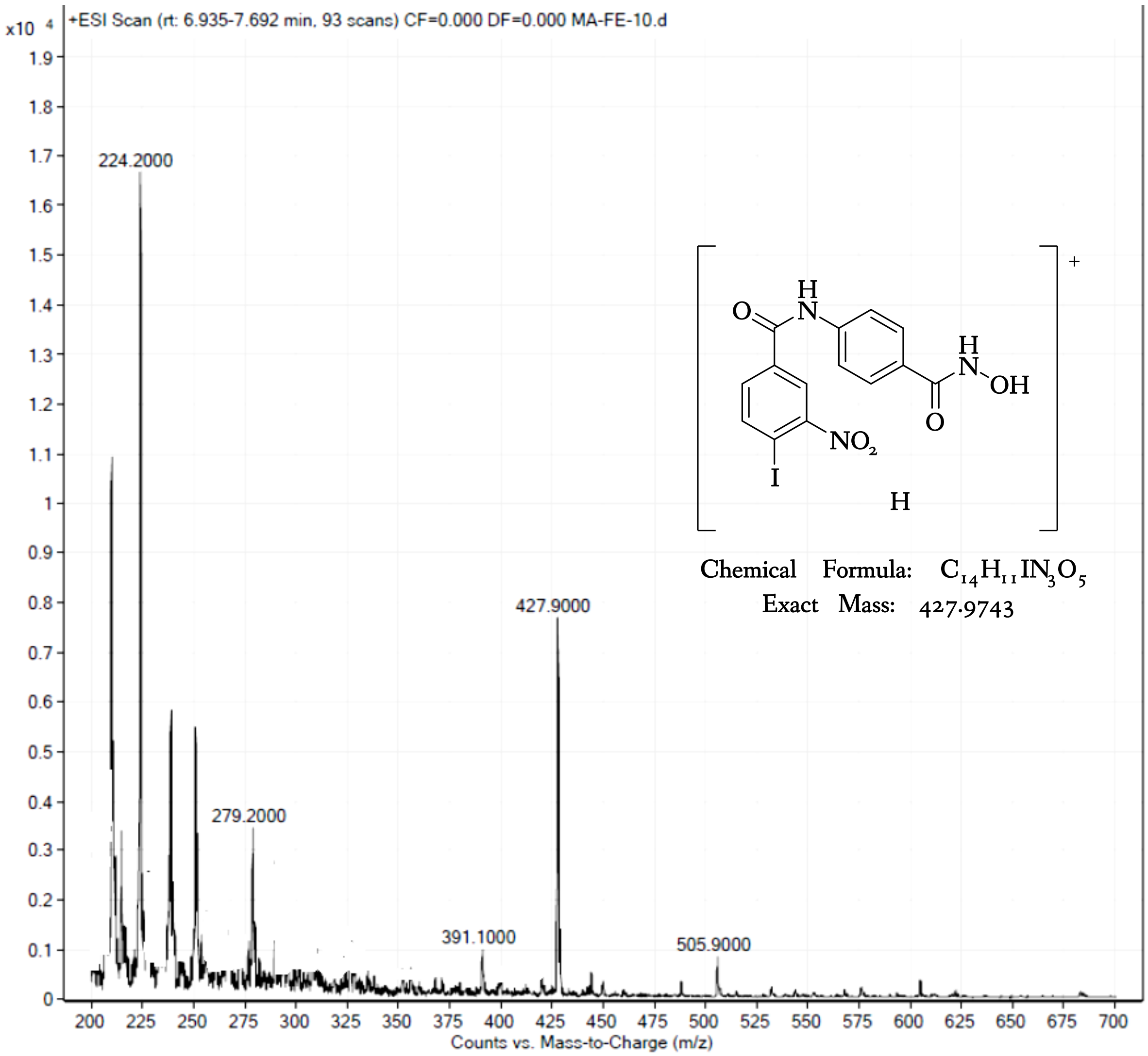

Figure S28: HRMS-ESI Spectrum of compound **7a**

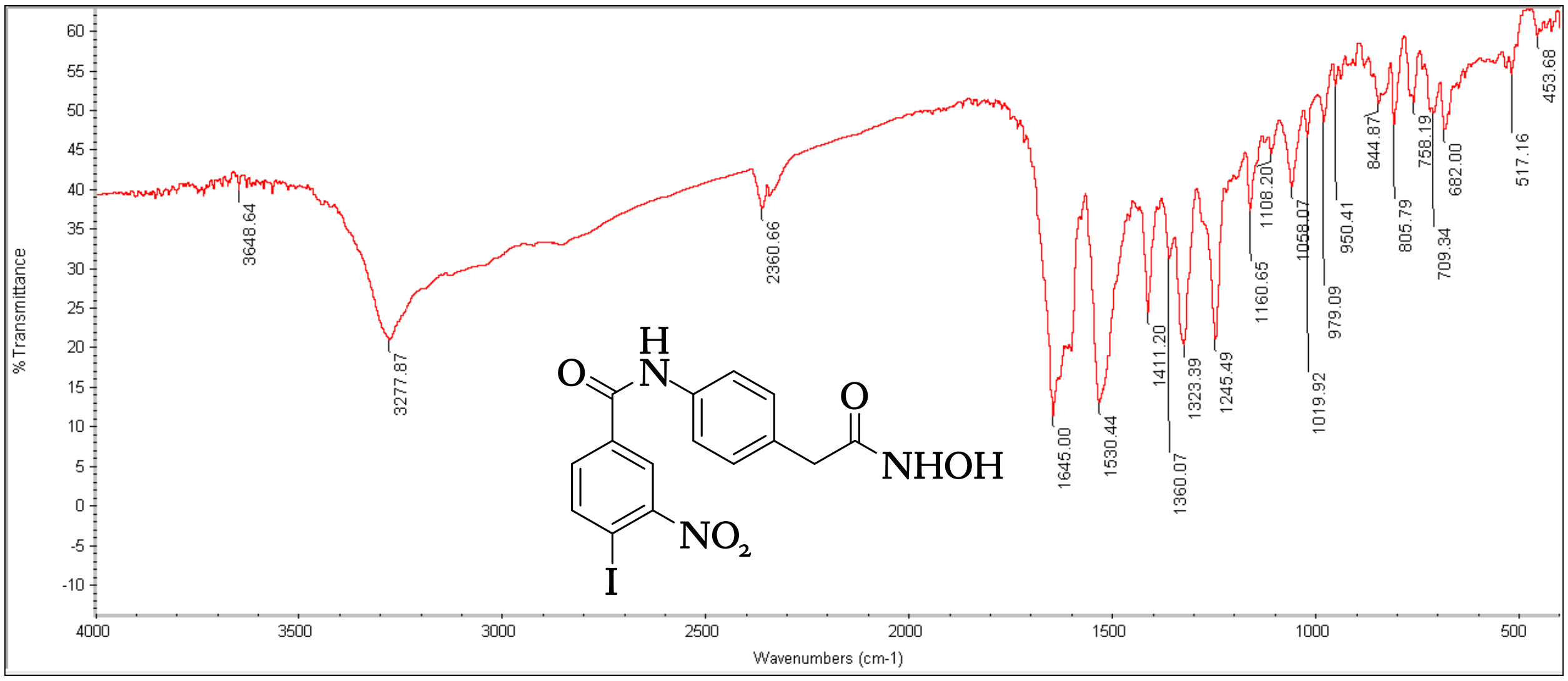

Figure S29: IR spectrum of compound **7b**

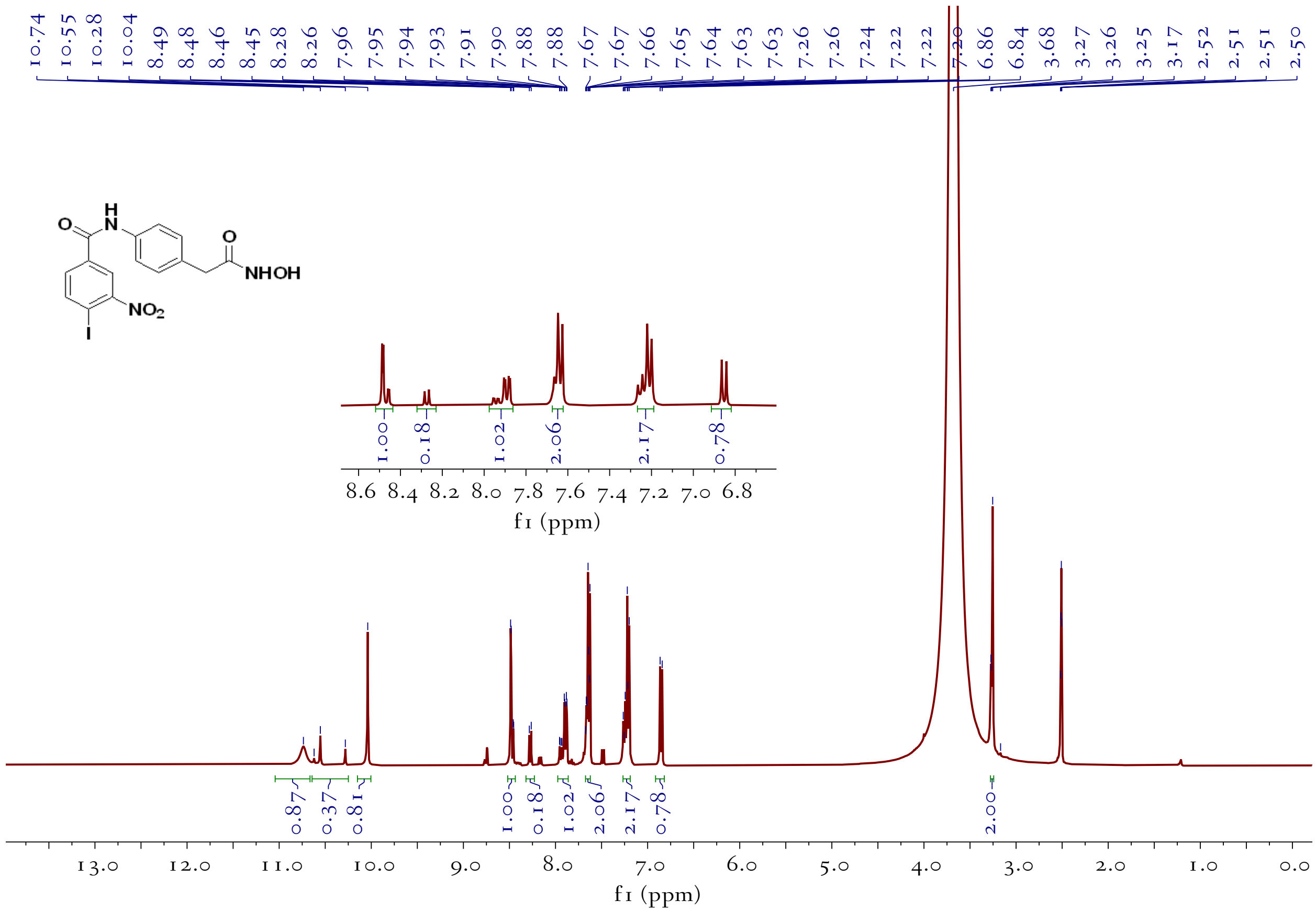

Figure S30: ^{1}H NMR (400 MHz, DMSO-d_6) of compound **7b**

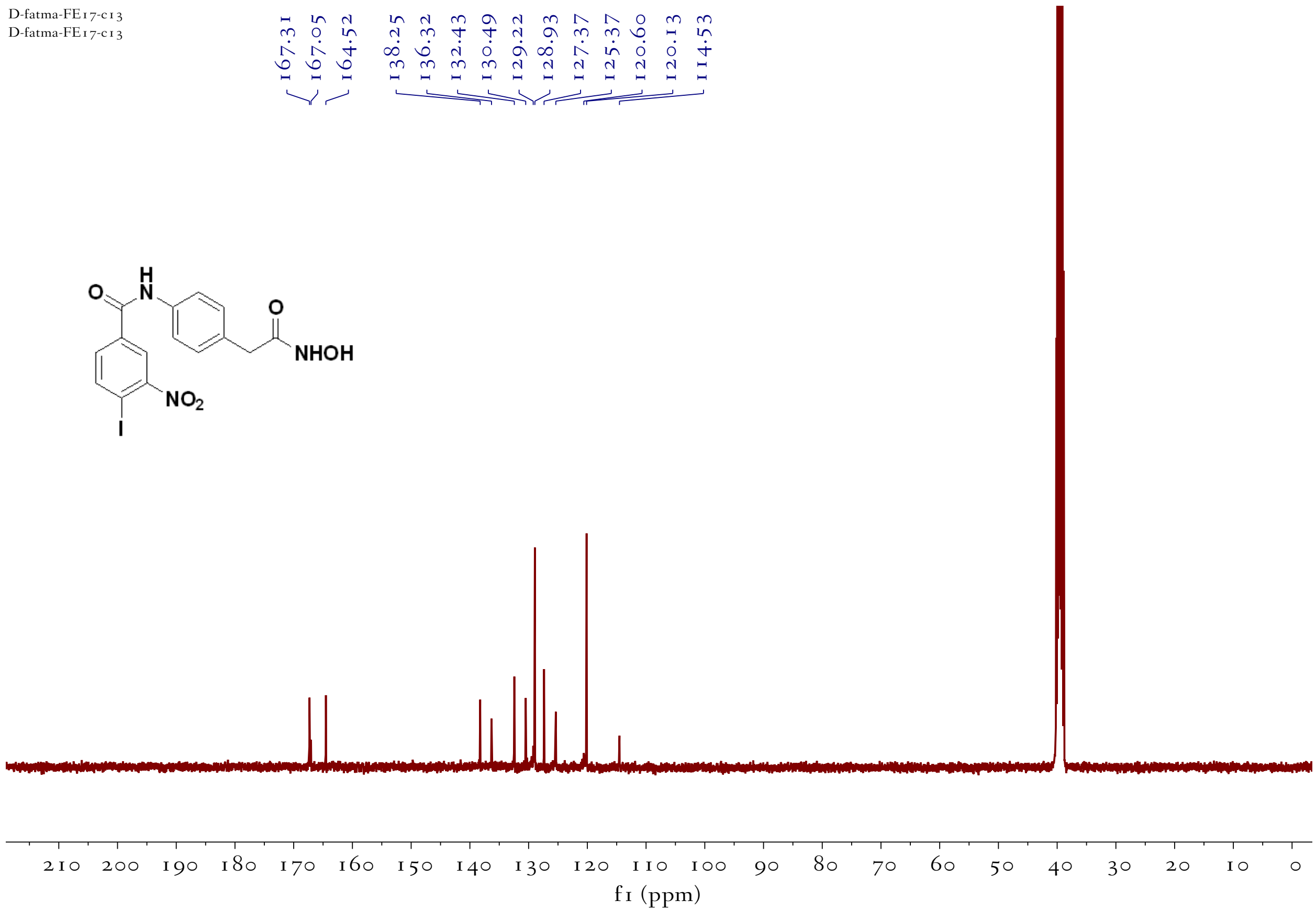

Figure S31: ^{13}C NMR (100 MHz, DMSO-d_6) of compound **7b**

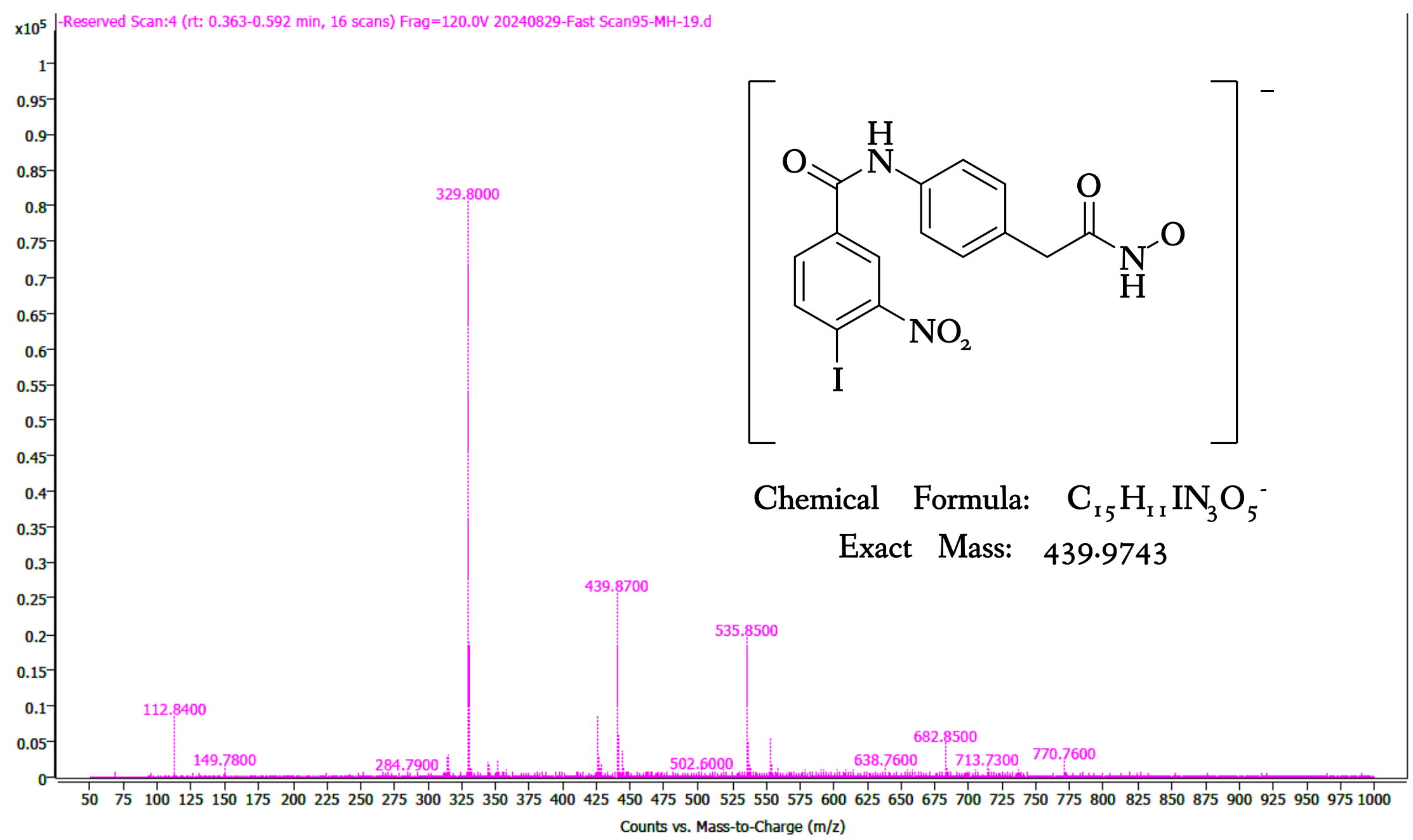

Figure S32: HRMS-ESI Spectrum of compound **7b**

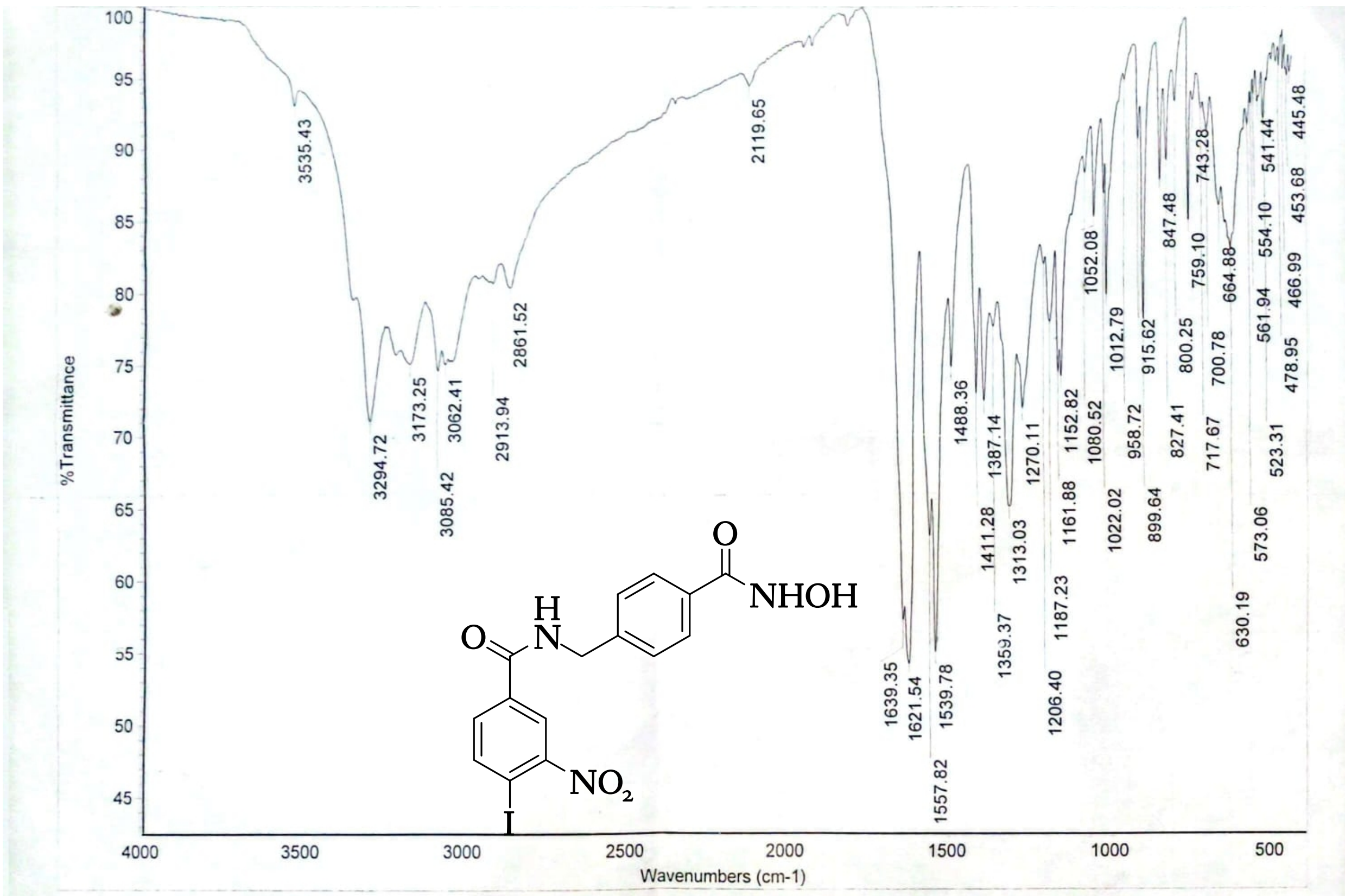

Figure S33: IR spectrum of compound **7c**

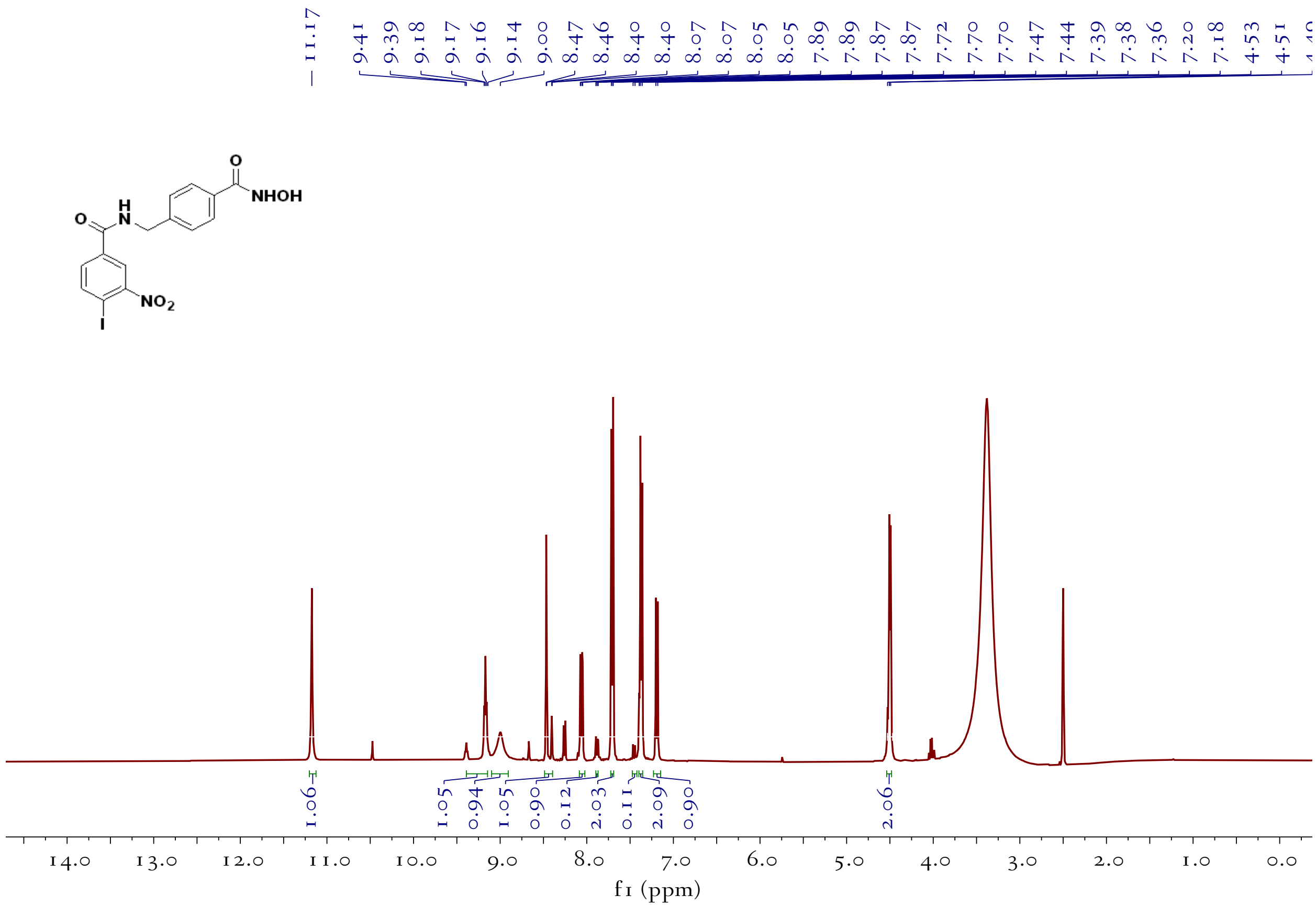

Figure S34: ^{1}H NMR (400 MHz, DMSO-d_6) of compound **7c**

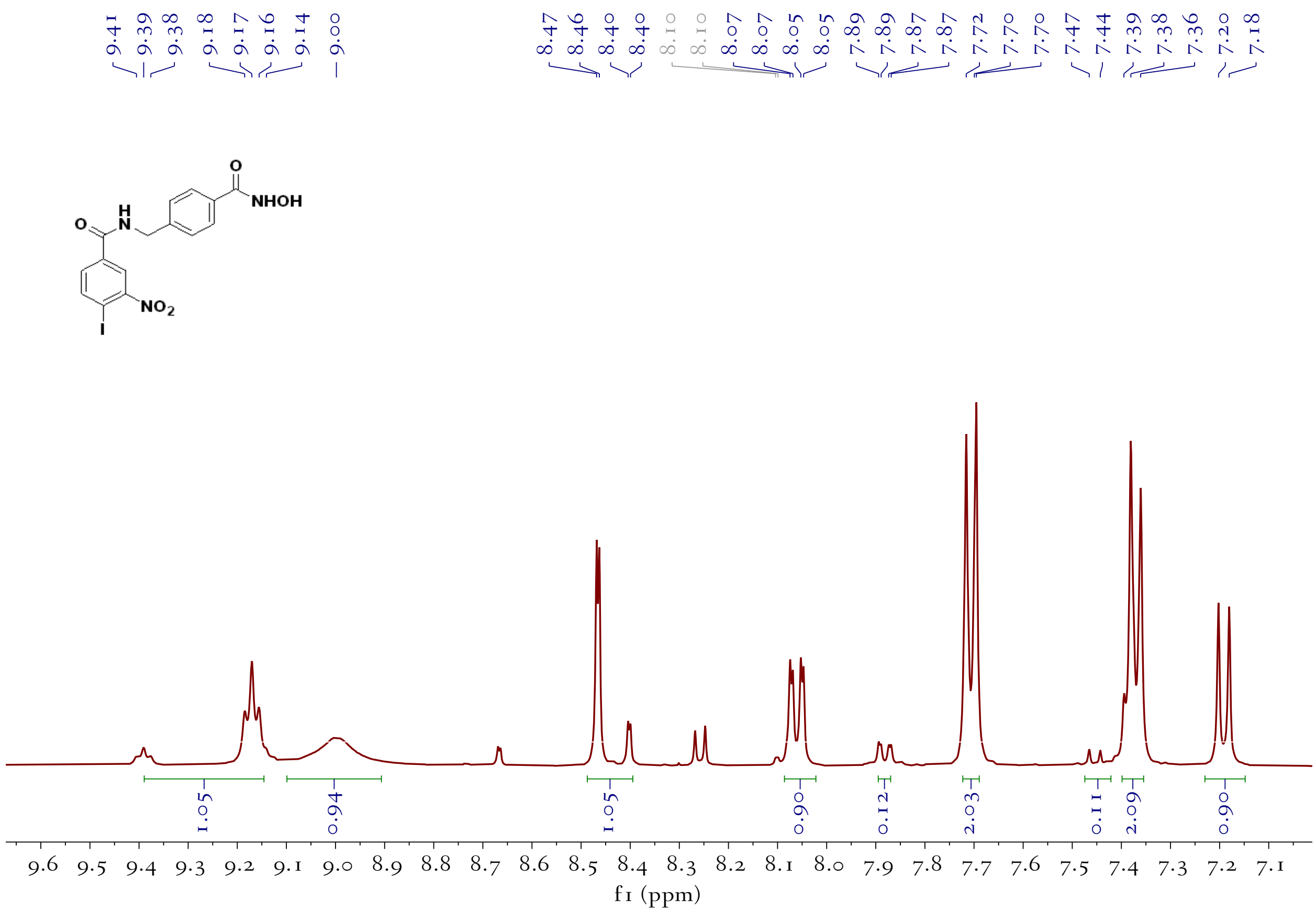

Figure S35: Aromatic expansion (400 MHz, DMSO-d_6) of compound **7c**

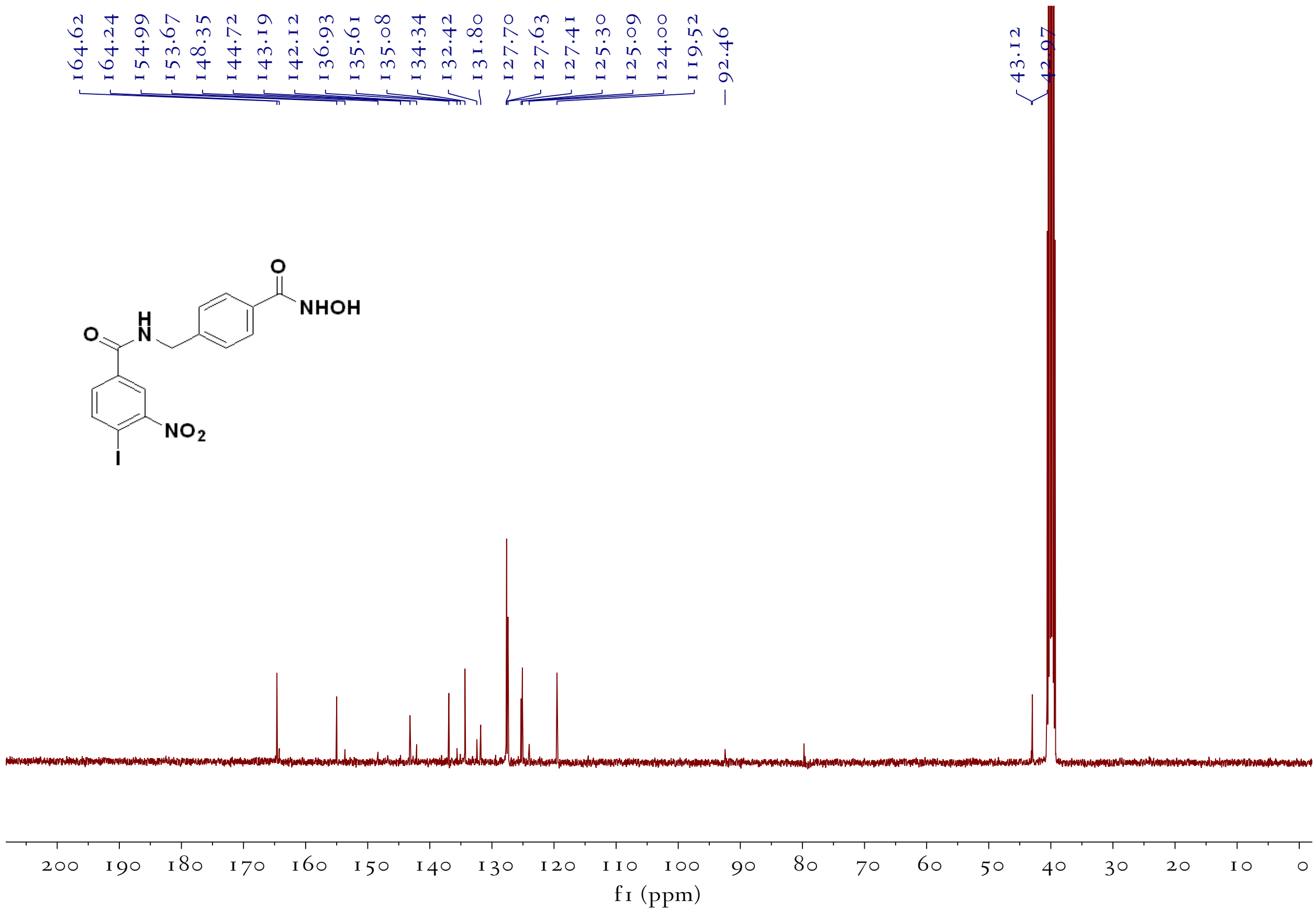

Figure S36: ^{13}C NMR (100 MHz, DMSO-d_6) of compound **7c**

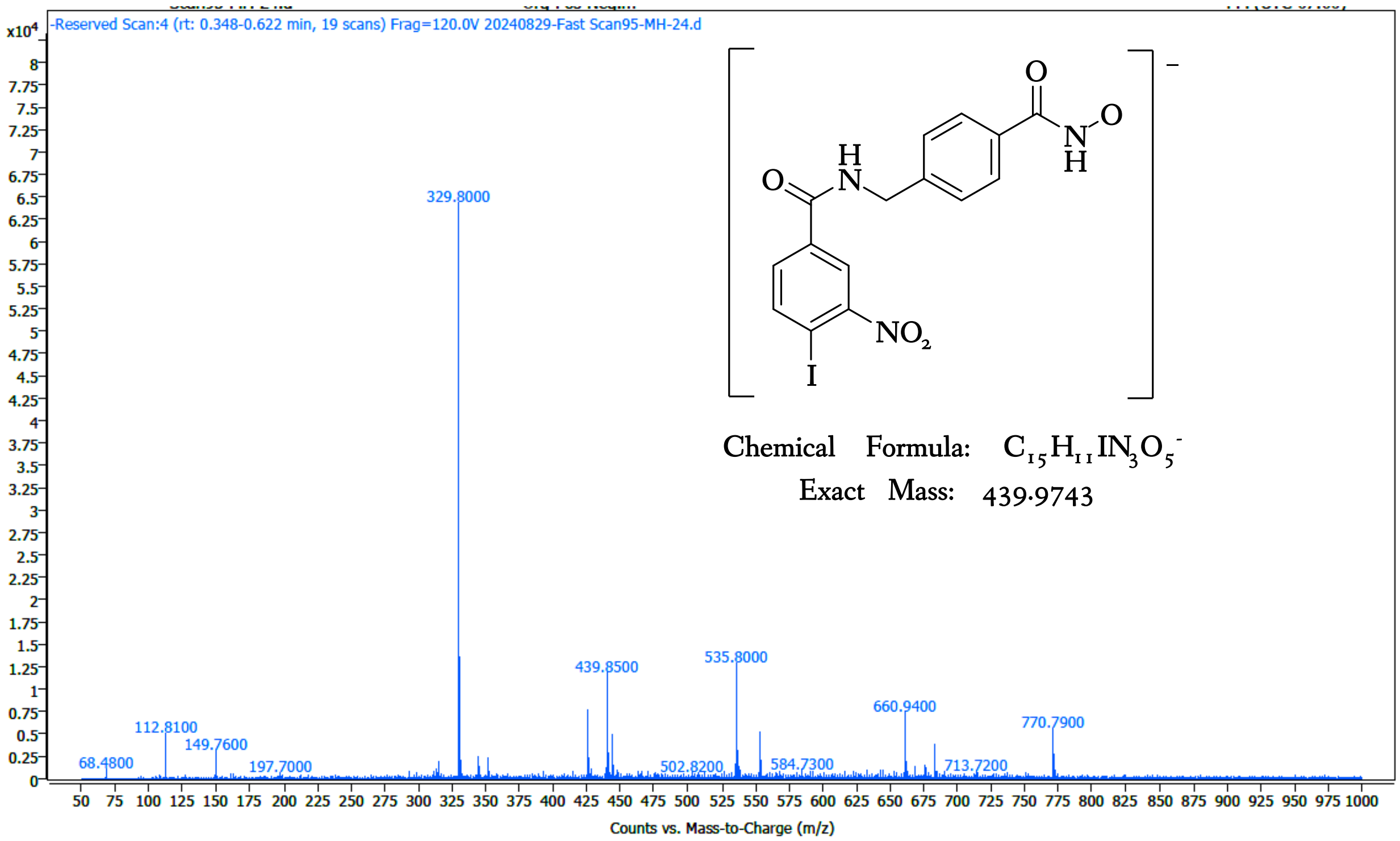

Figure S37: HRMS-ESI Spectrum of compound **7c**

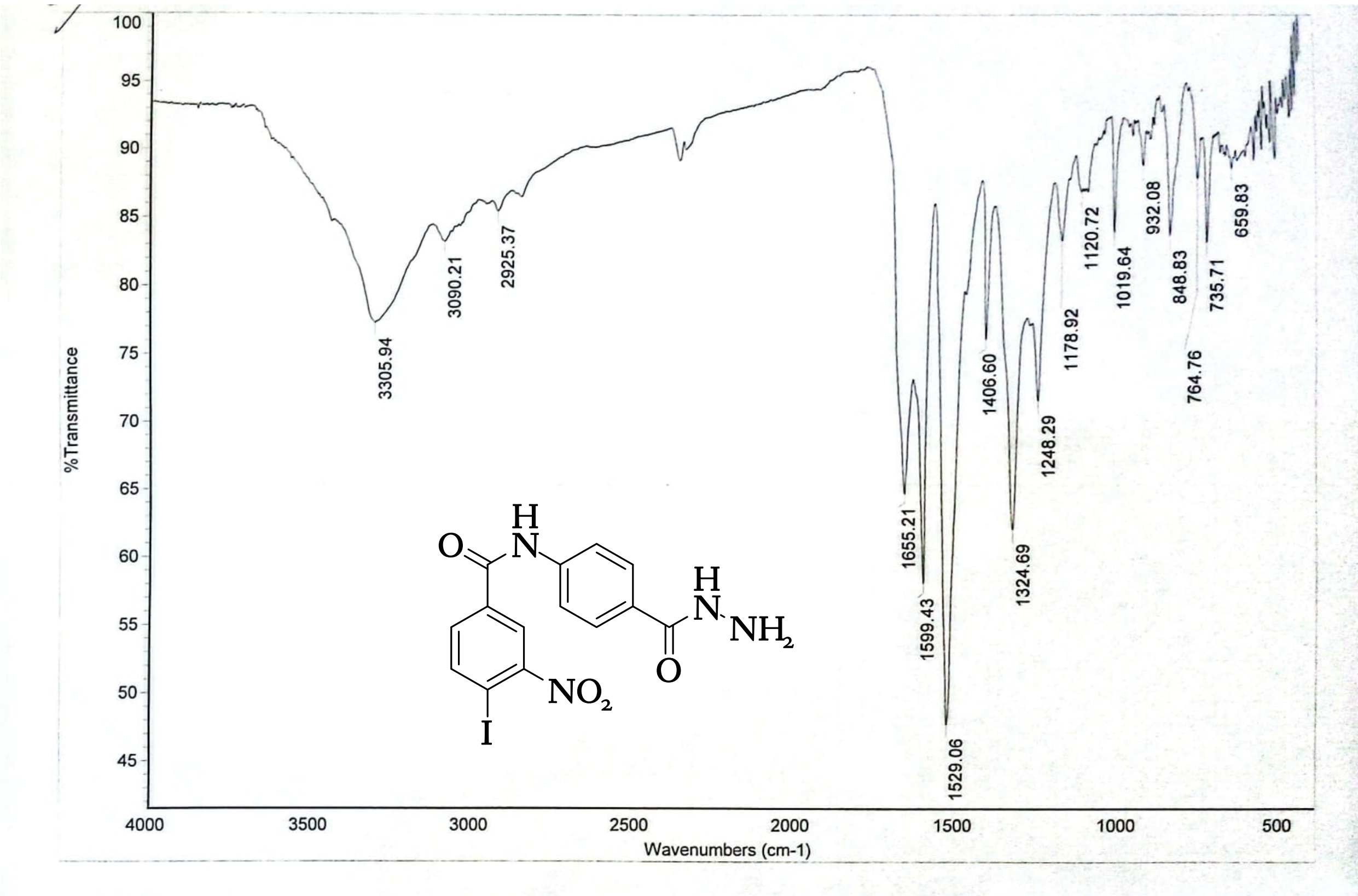

Figure S38: IR spectrum of compound **7d**

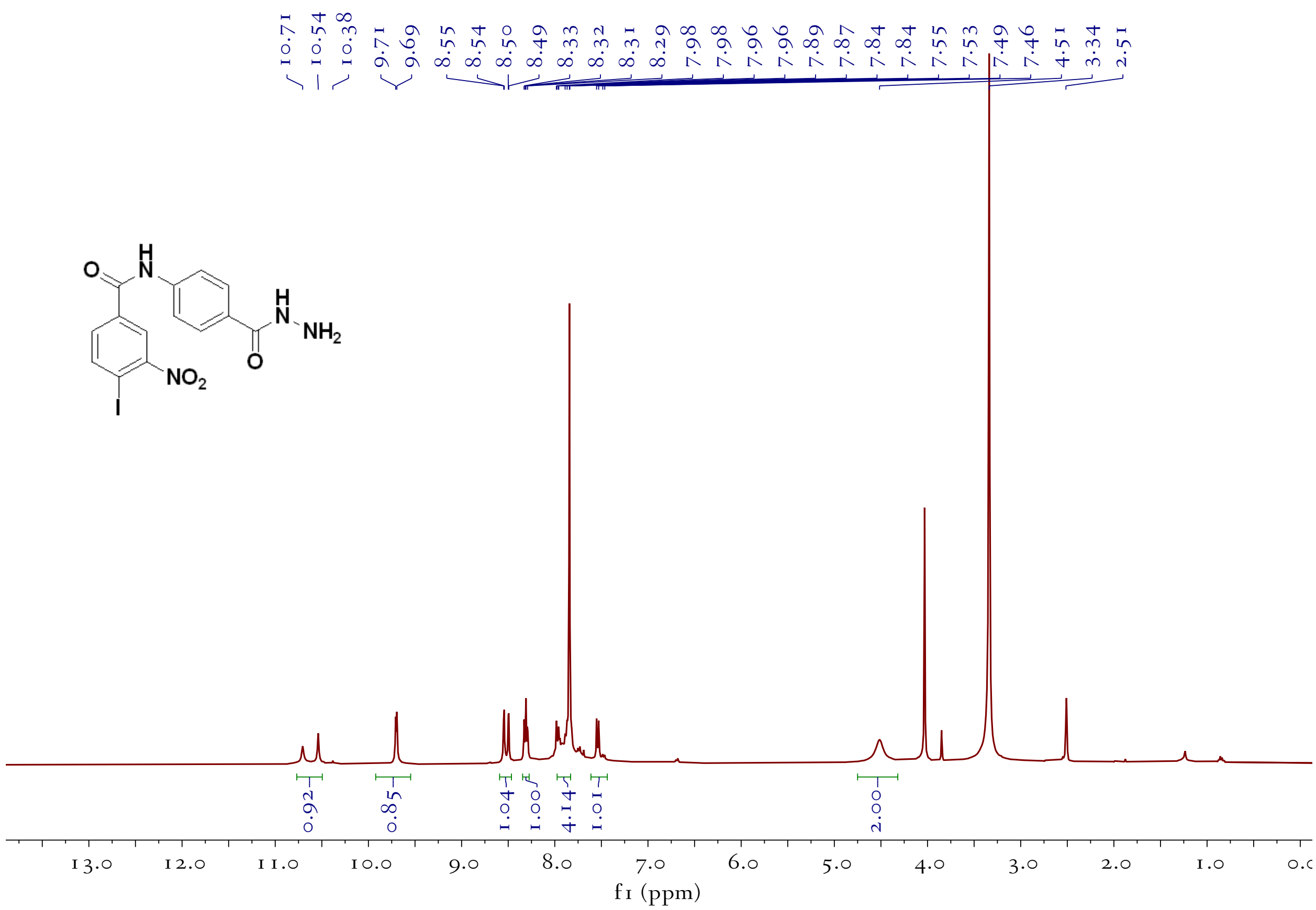

Figure S39: ^{1}H NMR (400 MHz, DMSO-d_6) of compound **7d**

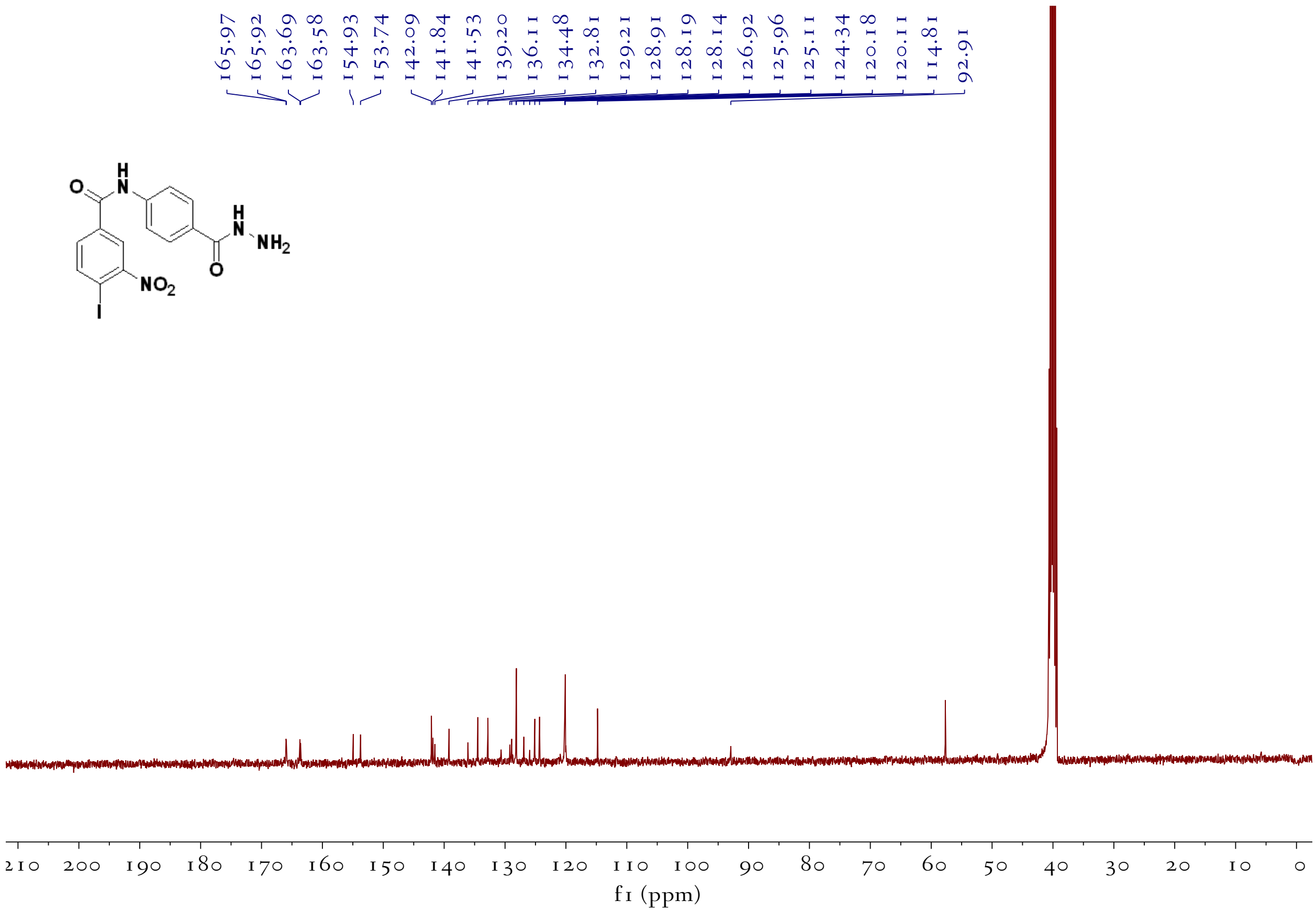

Figure S40: ^{13}C NMR (100 MHz, DMSO-d_6) of compound **7d**

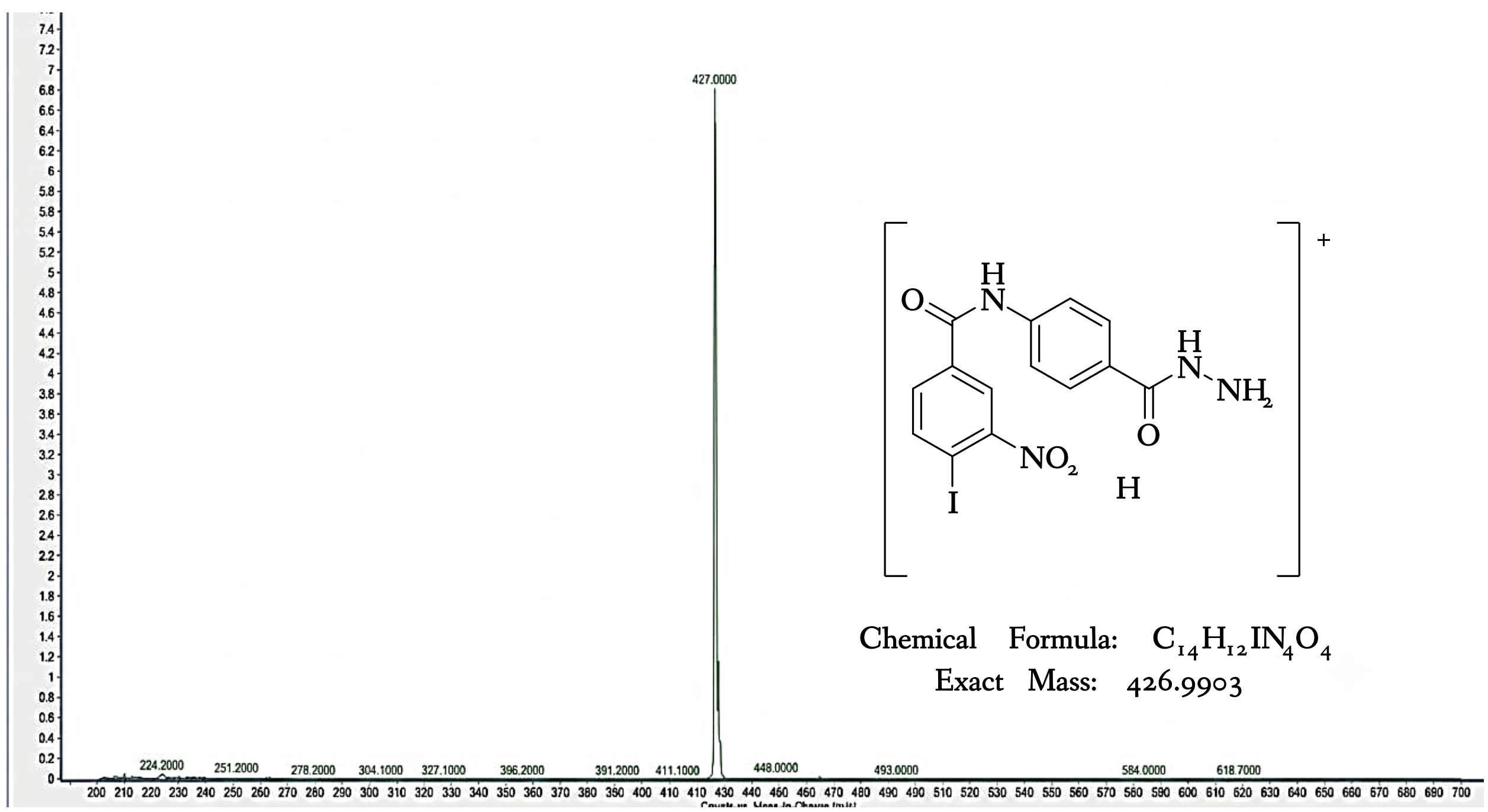

Figure S41: HRMS-ESI Spectrum of compound **7d**

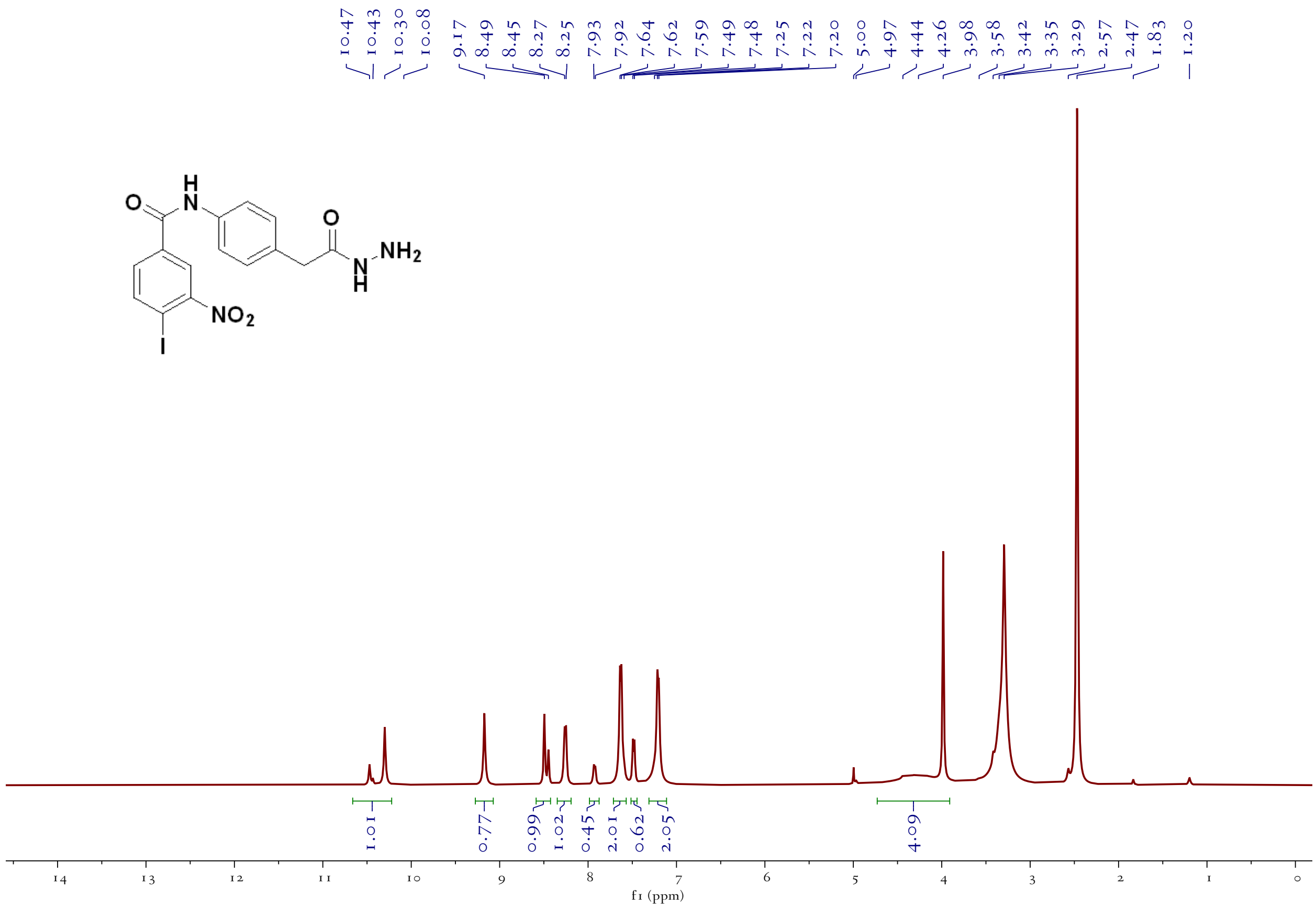

Figure S42: ^{1}H NMR (400 MHz, DMSO-d_6) of compound **7e**

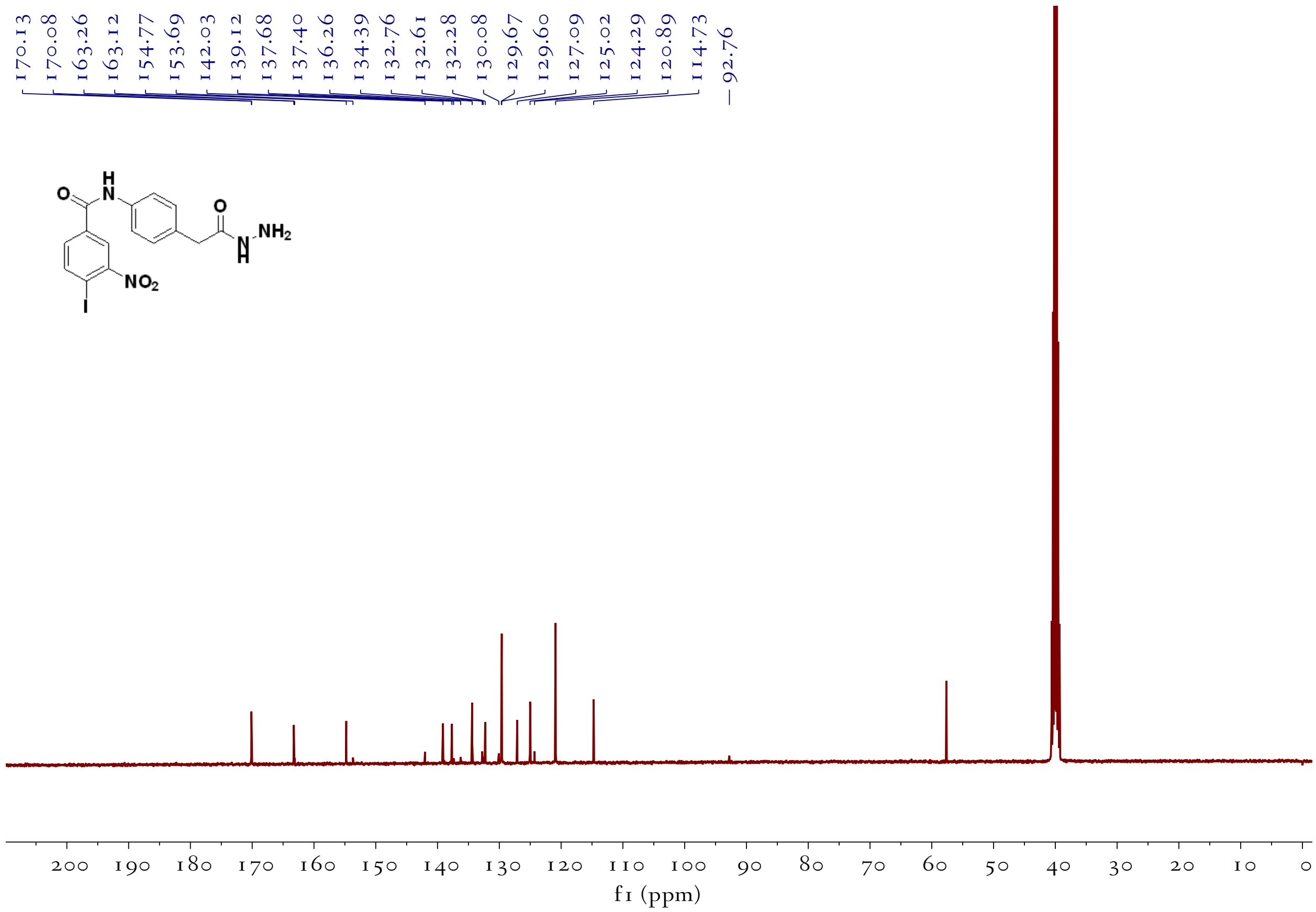

Figure S43: ^{13}C NMR (100 MHz, DMSO-d_6) of compound **7e**

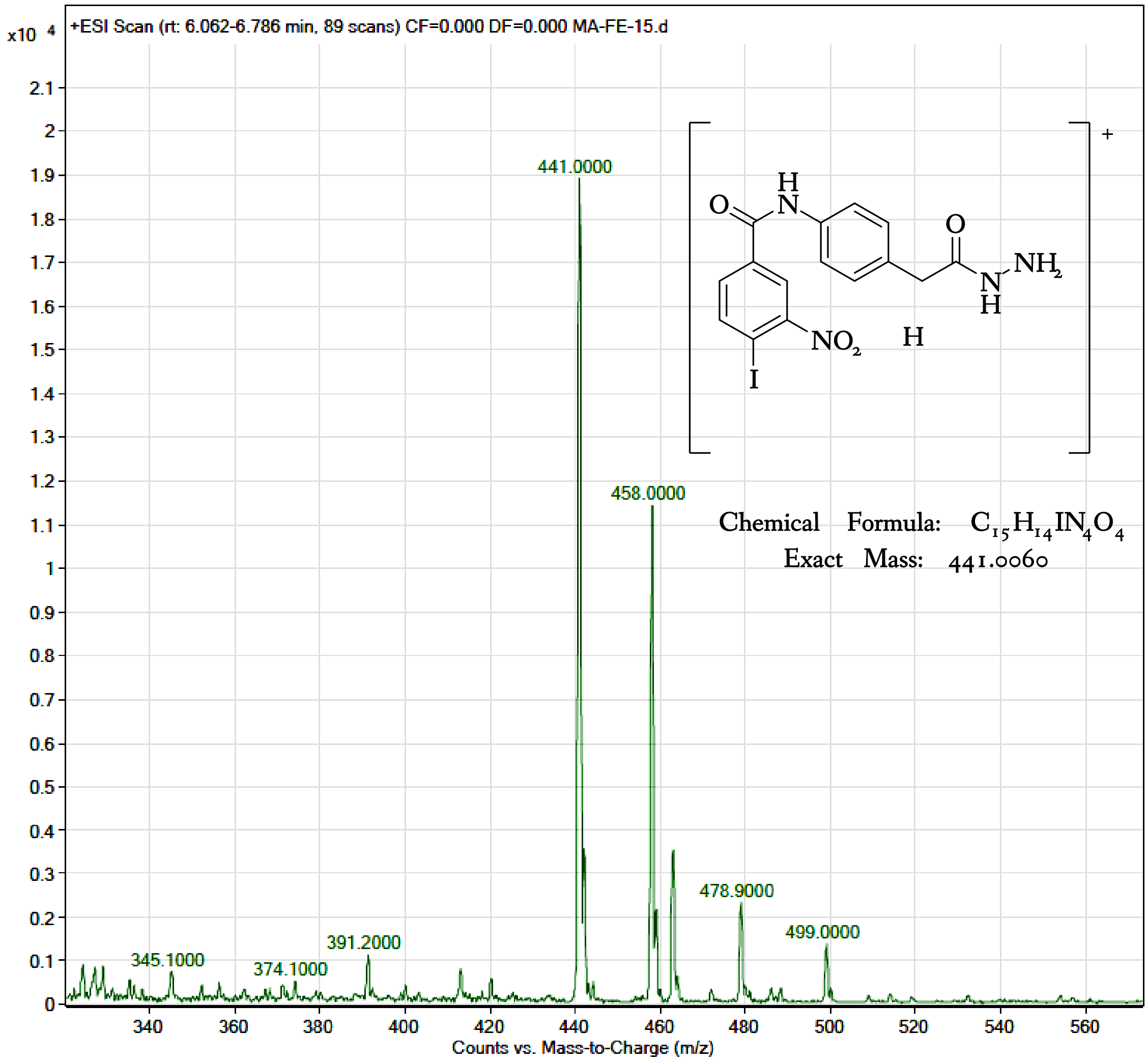

Figure S44: HRMS-ESI Spectrum of compound **7e**

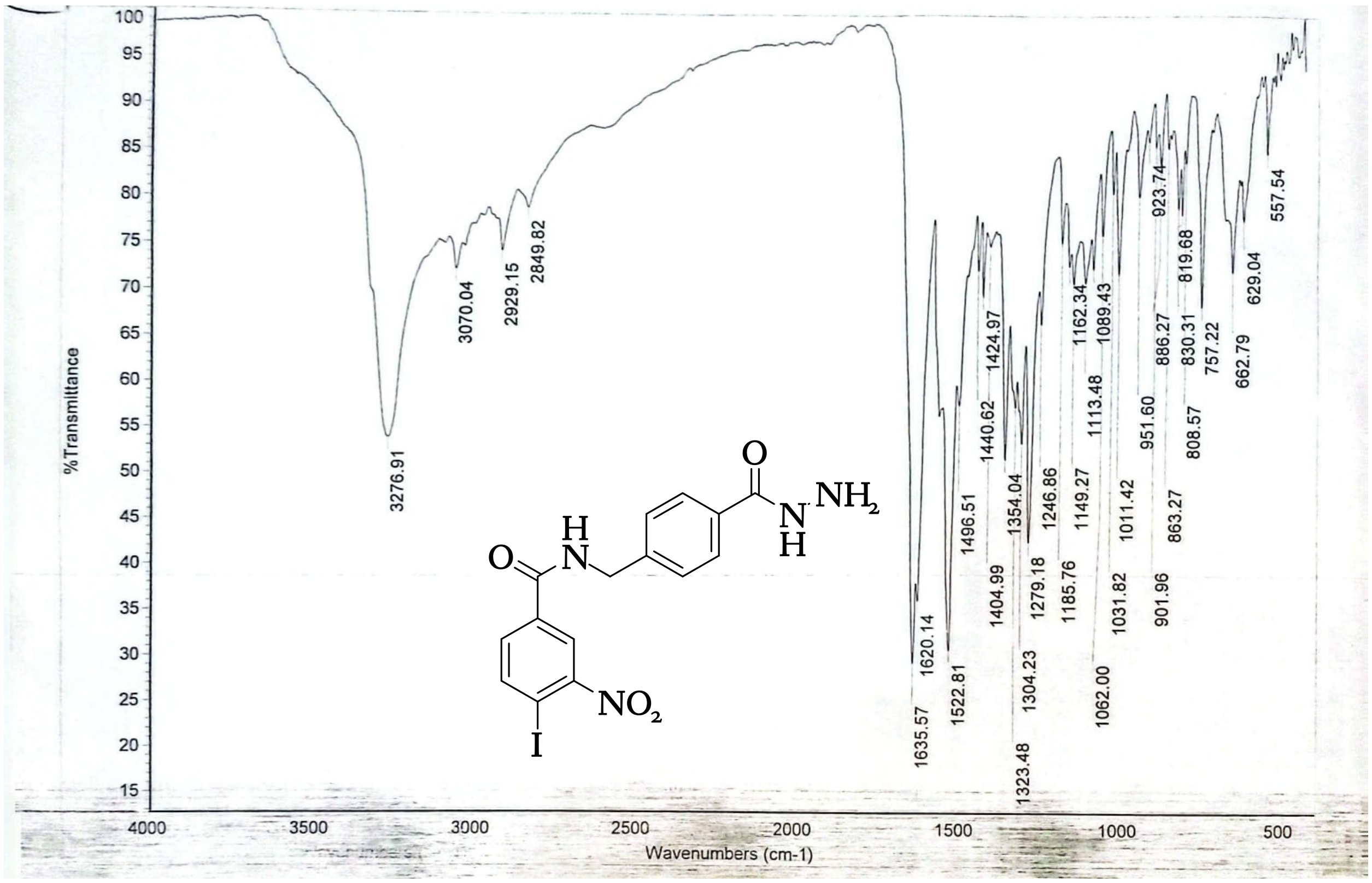

Figure S45: IR spectrum of compound **7f**

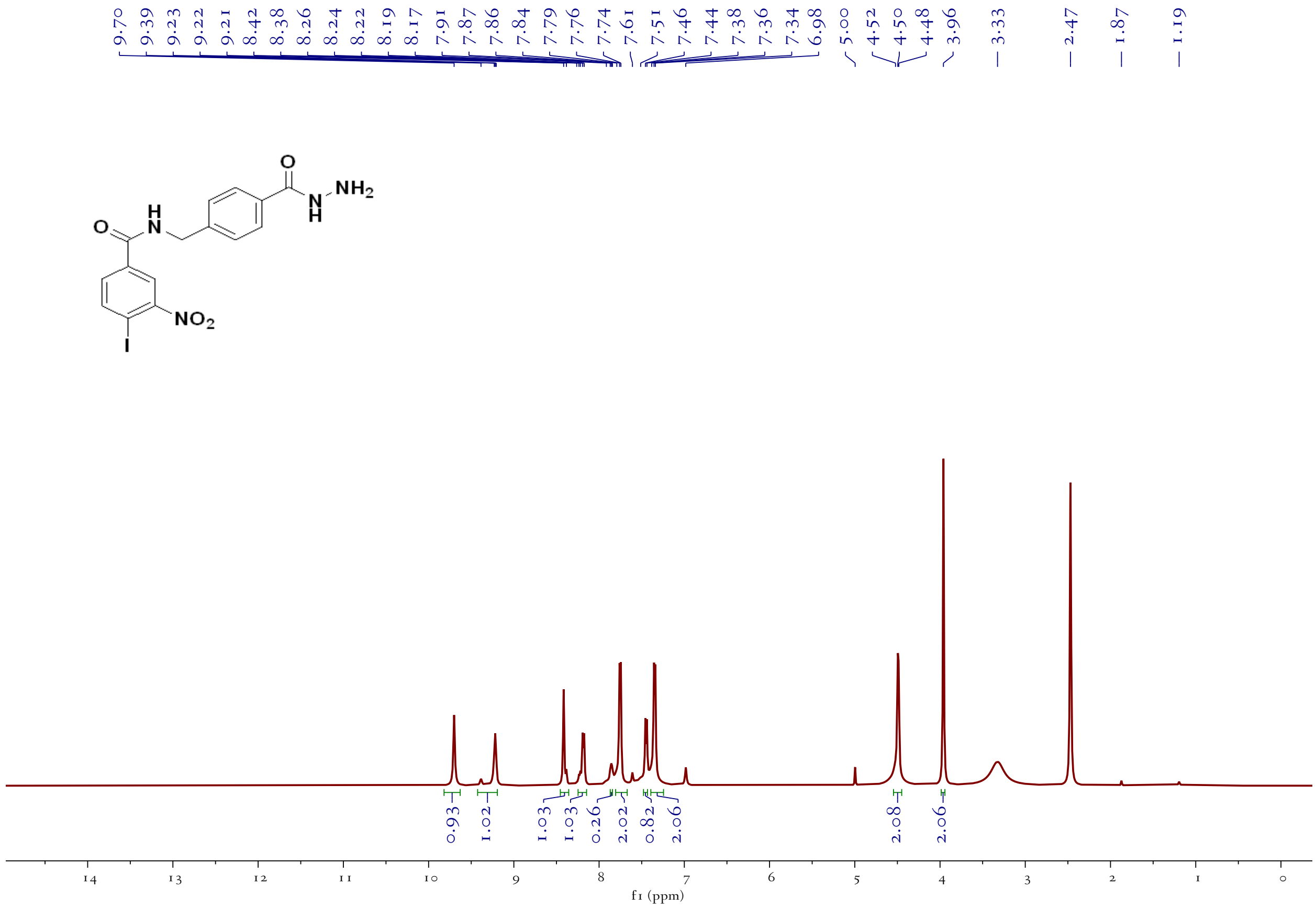

Figure S46: ^{1}H NMR (400 MHz, DMSO-d_6) of compound **7f**

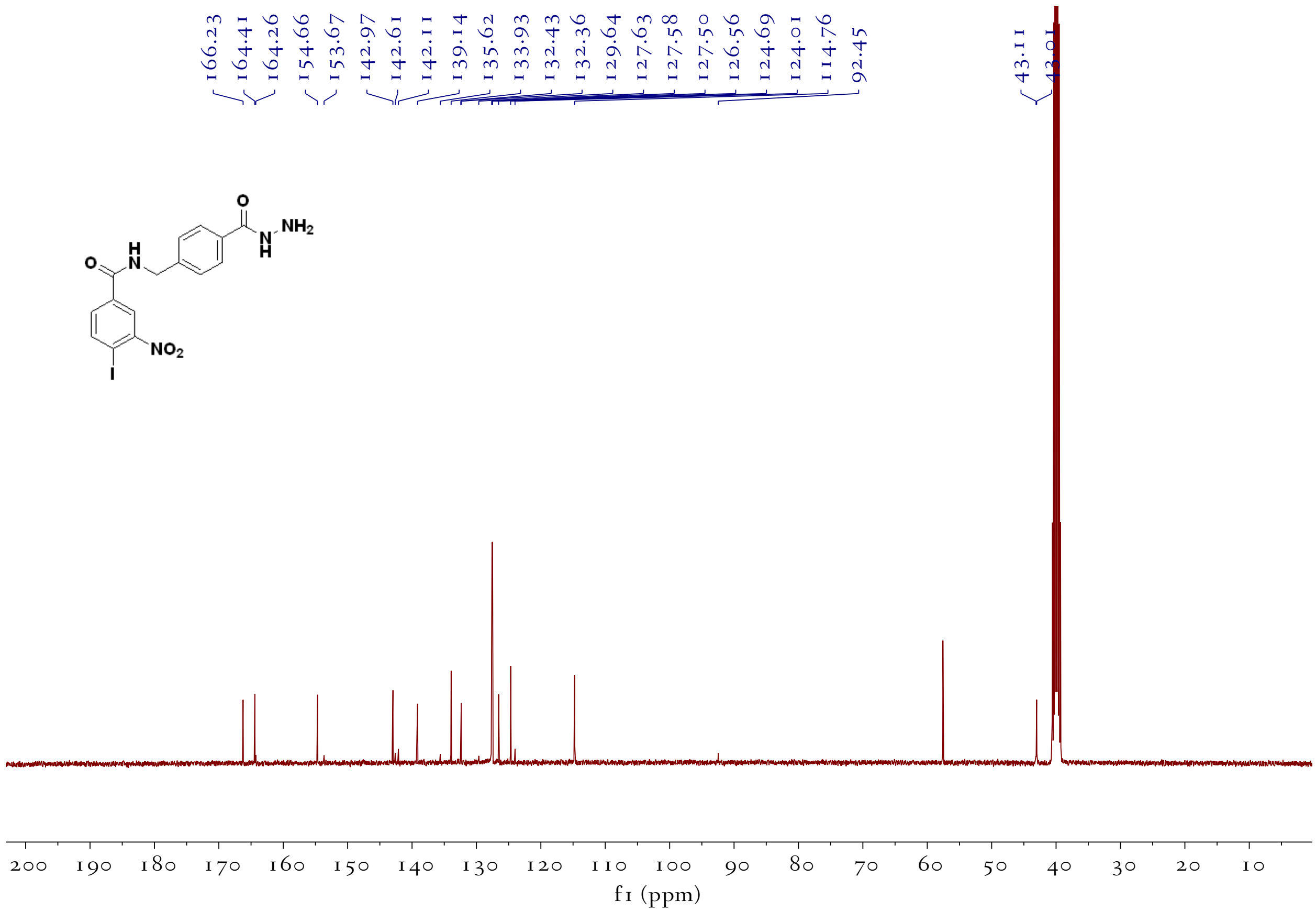

Figure S47: ^{13}C NMR (100 MHz, DMSO-d_6) of compound **7f**

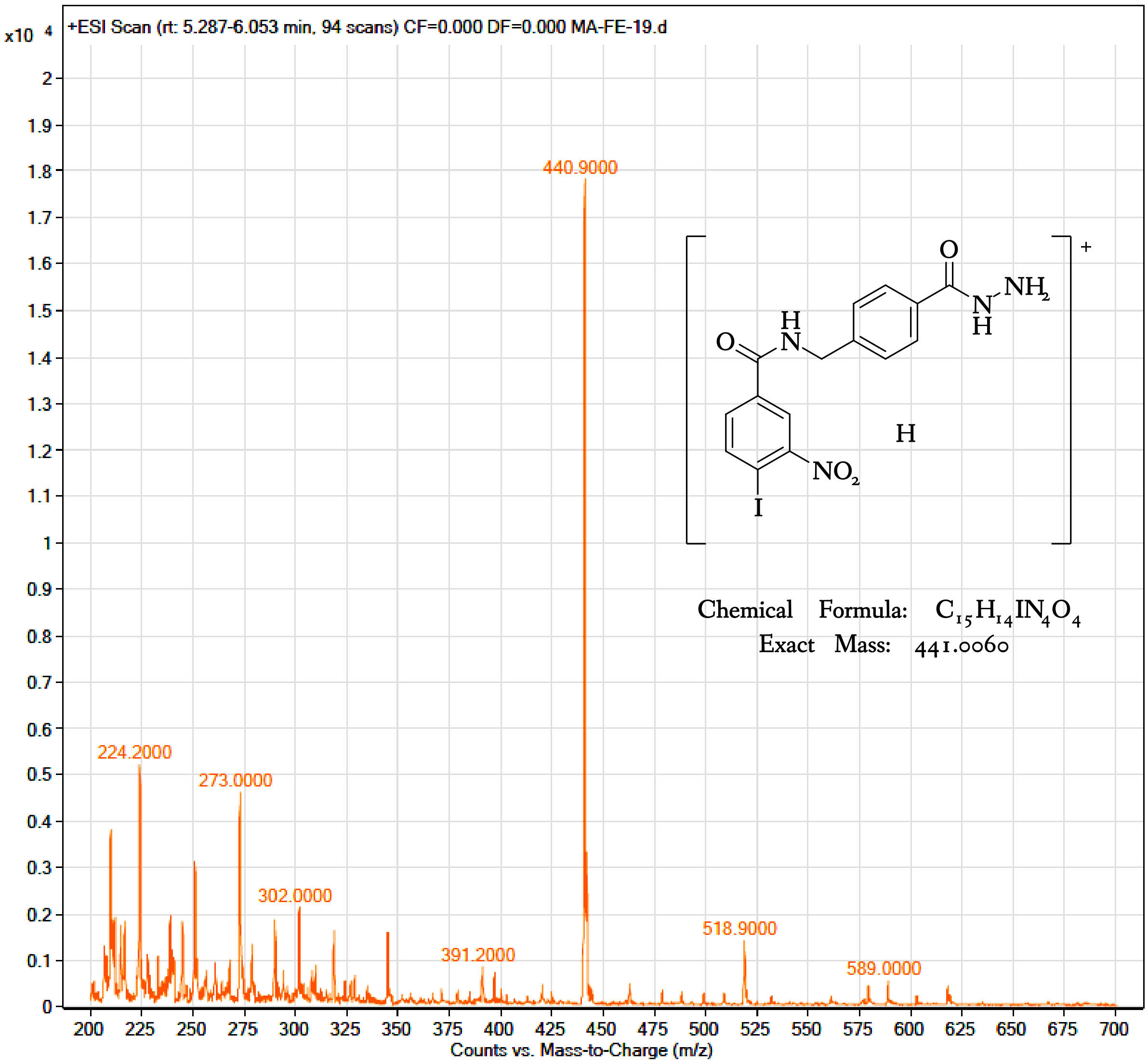

Figure S48: HRMS-ESI Spectrum of compound **7f**

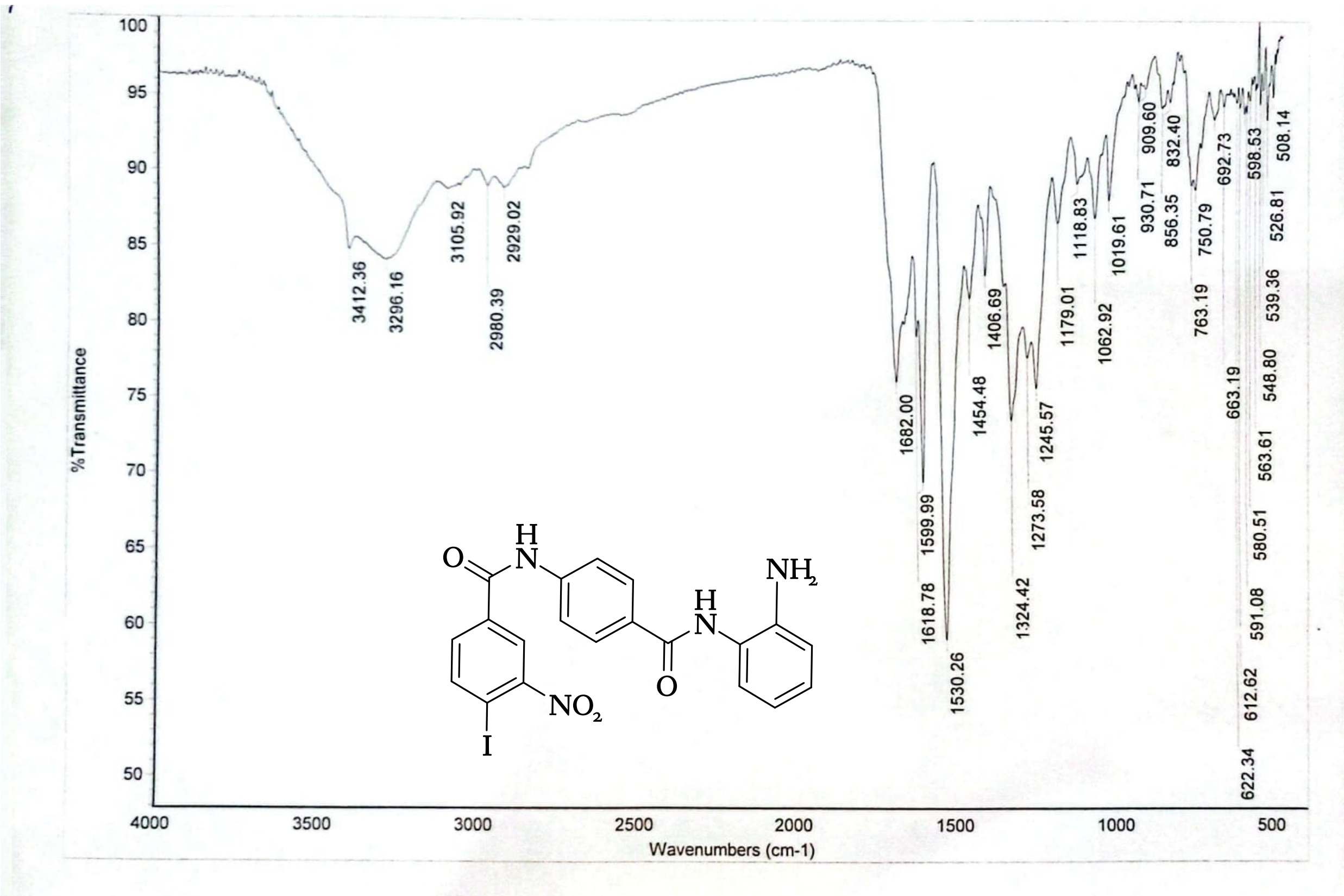

Figure S49: IR spectrum of compound **7g**

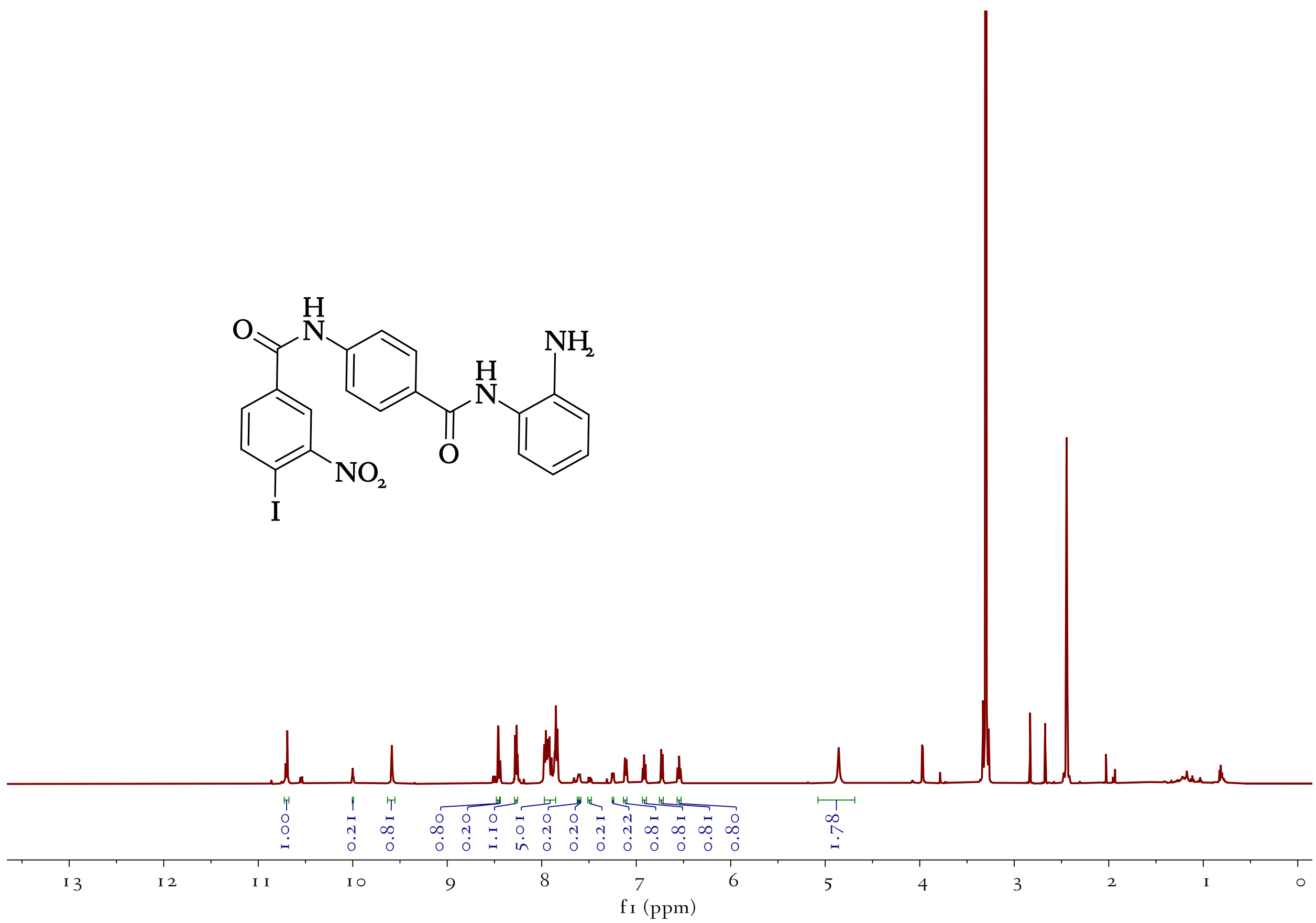

Figure S50: ^{1}H NMR (400 MHz, DMSO-d_6) of compound **7g**

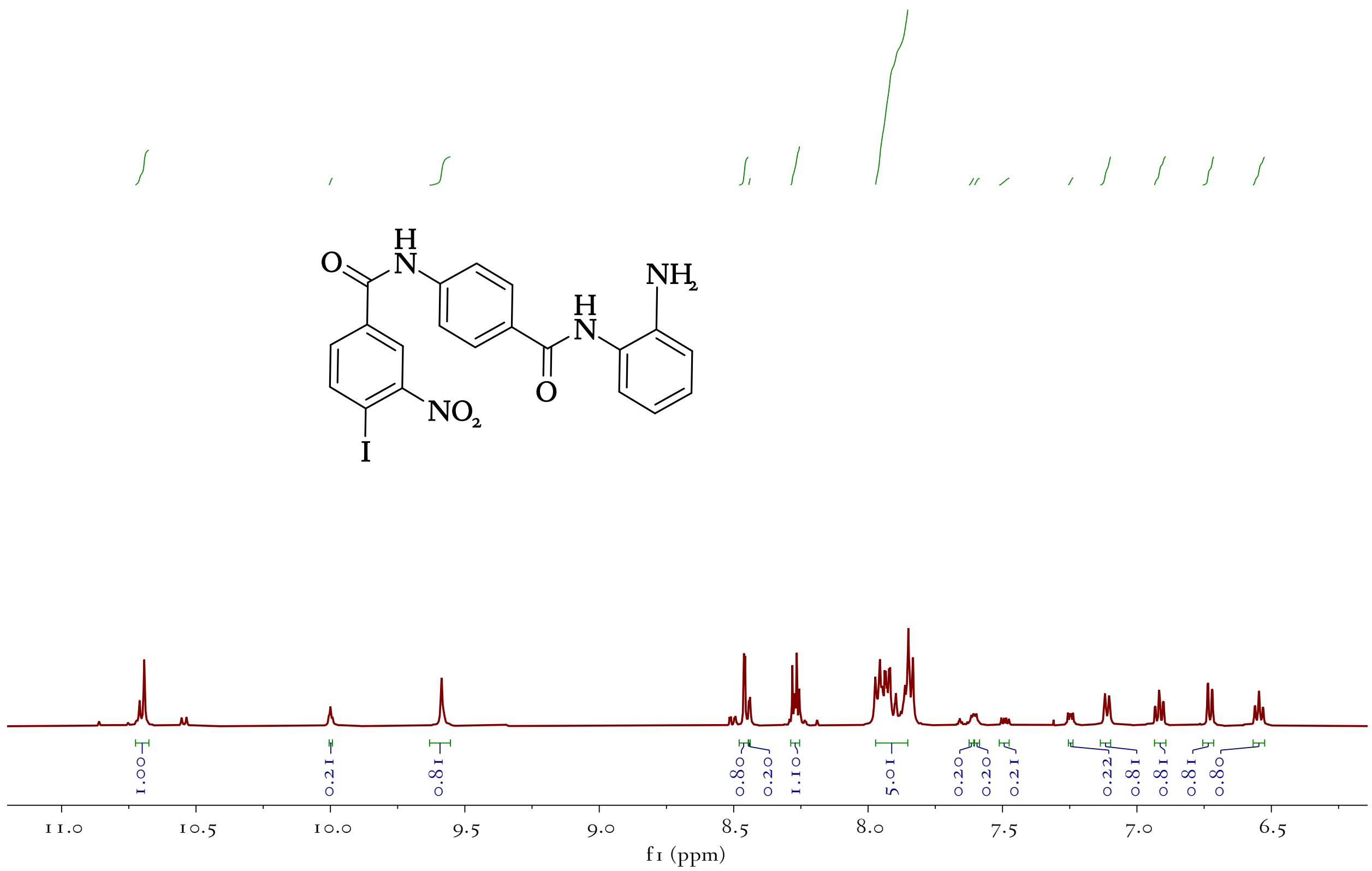

Figure S51: ^{1}H NMR expansion (400 MHz, DMSO-d_6) of compound **7g**

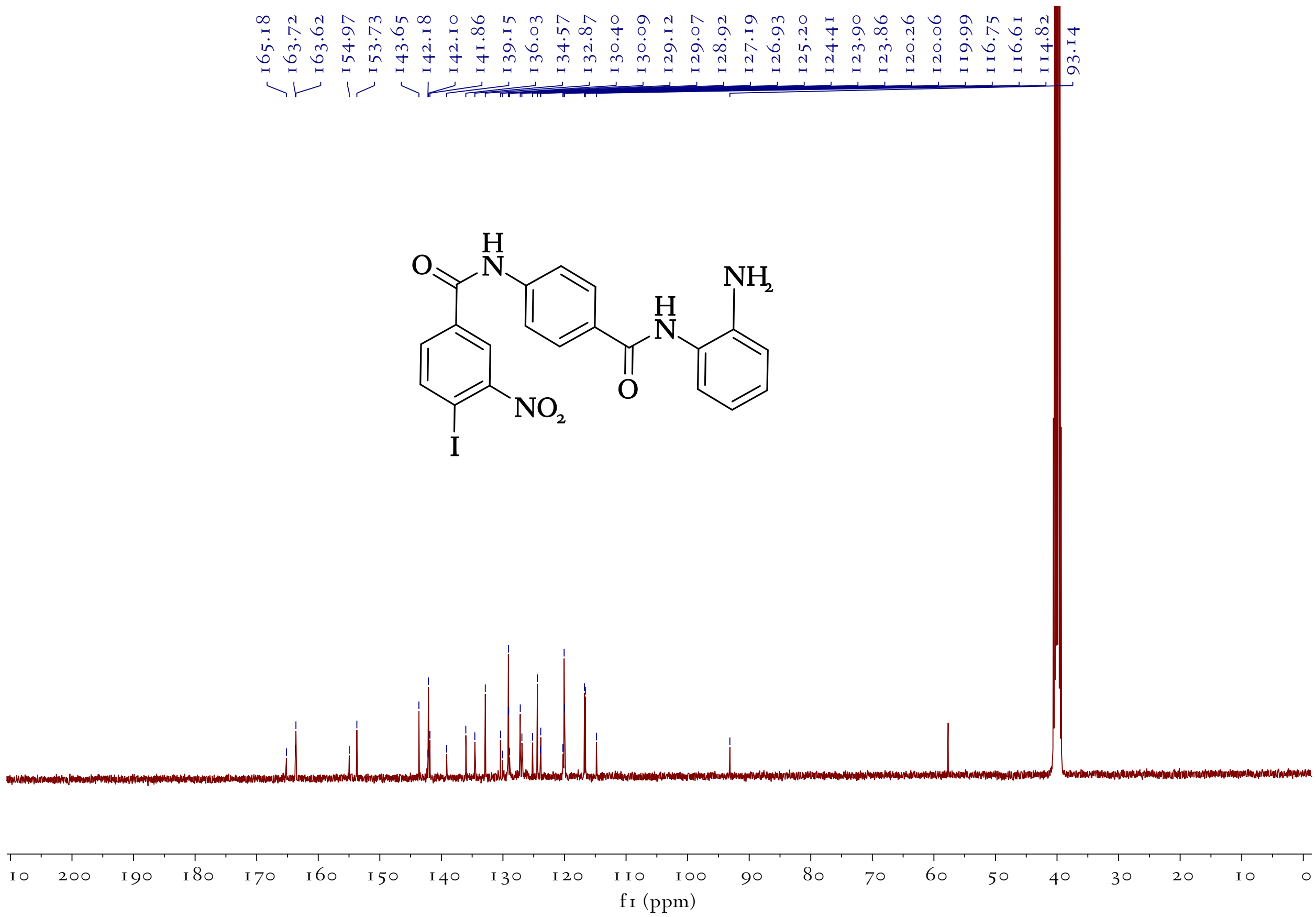

Figure S52: ^{13}C NMR (100 MHz, DMSO-d_6) of compound **7g**

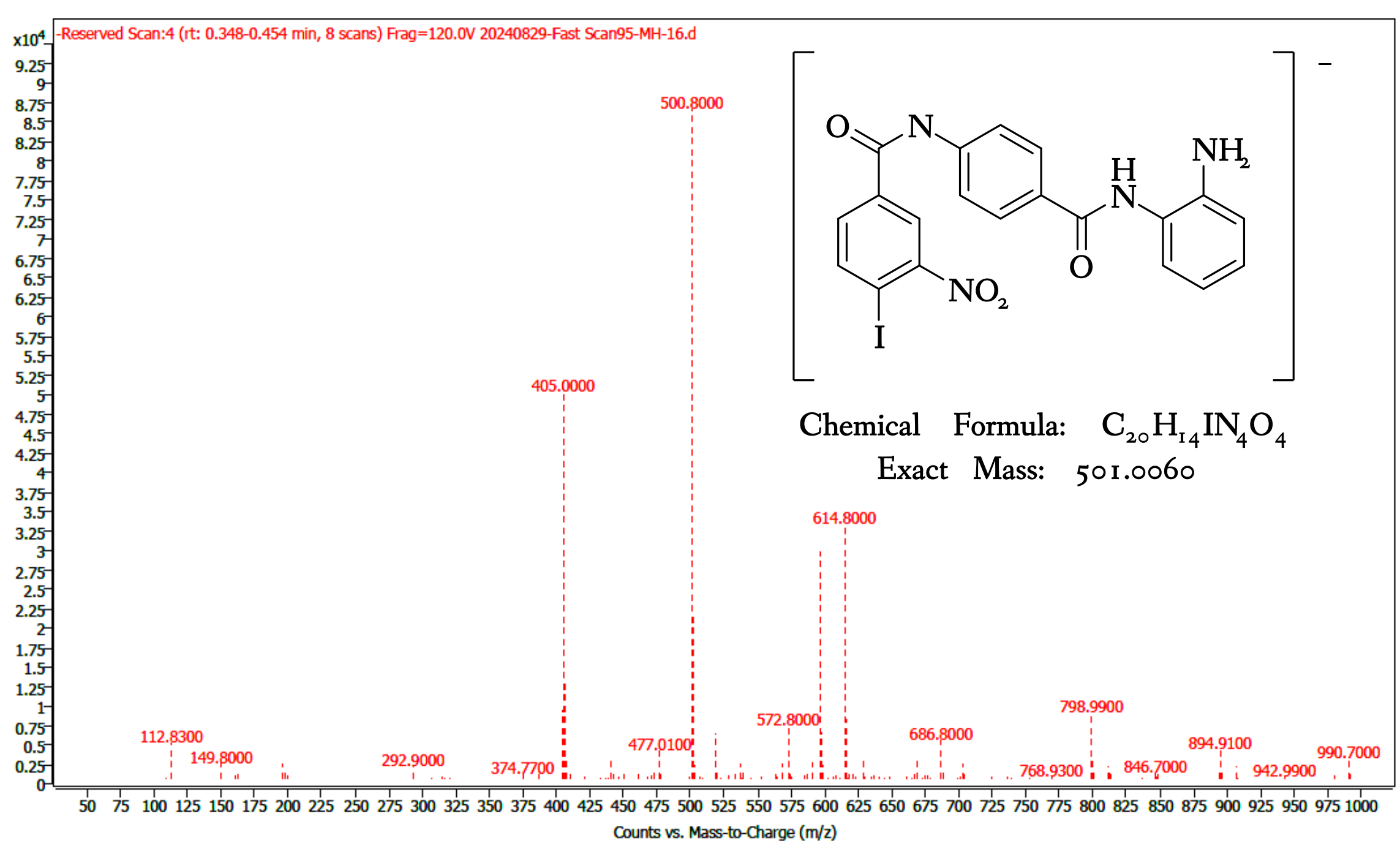

Figure S53: HRMS-ESI Spectrum of compound **7g**

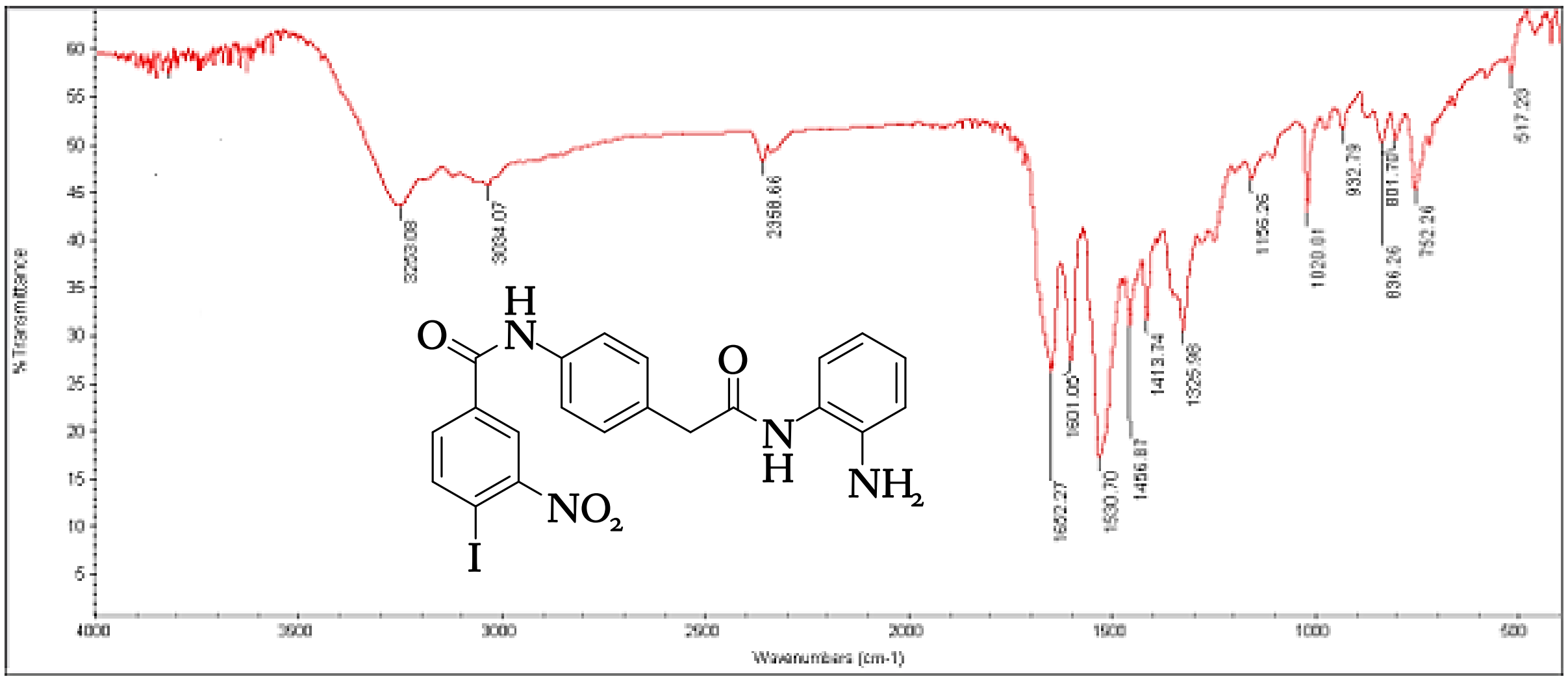

Figure S54: IR spectrum of compound **7h**

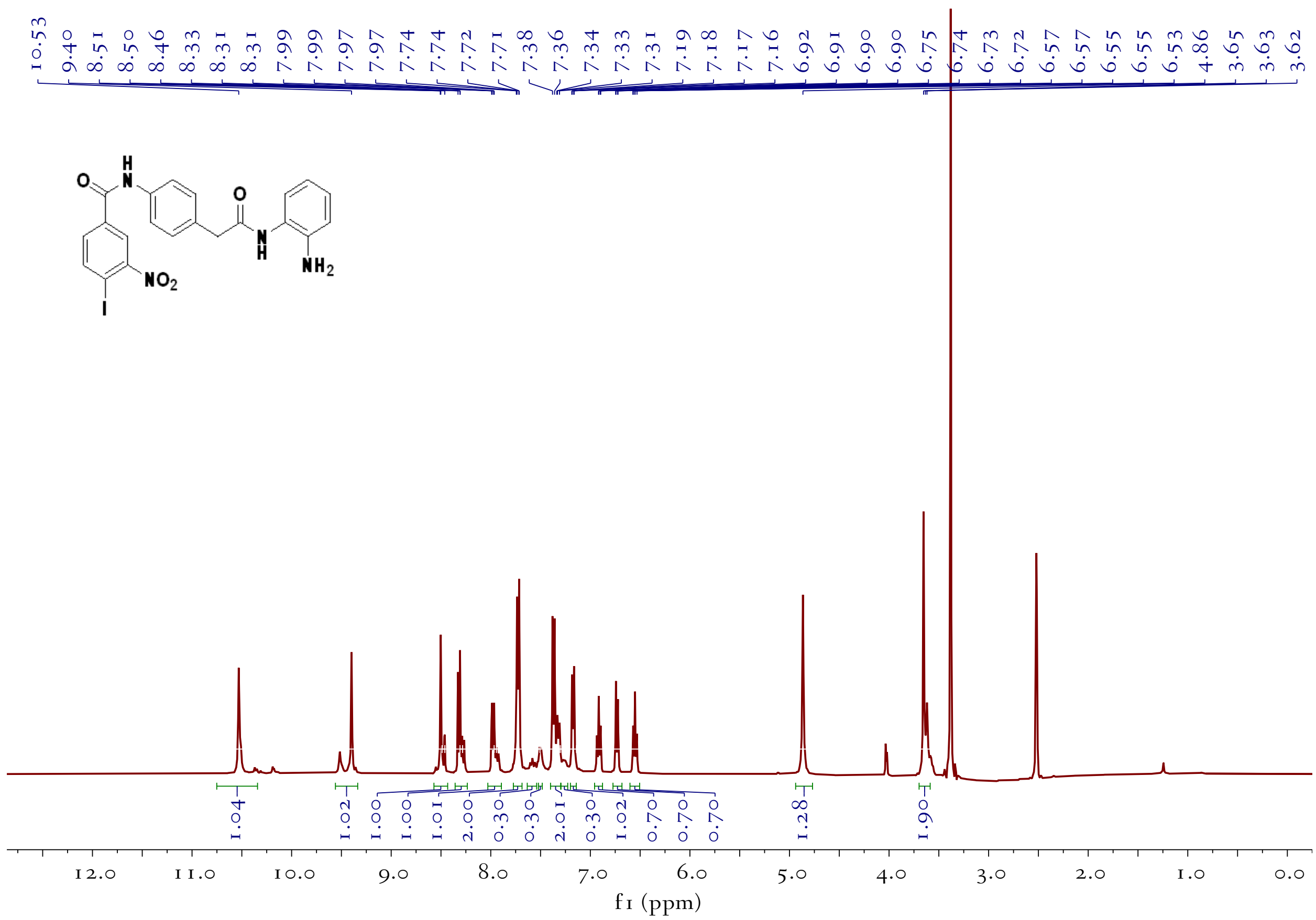

Figure S55: ^{1}H NMR (400 MHz, DMSO-d_6) of compound **7h**

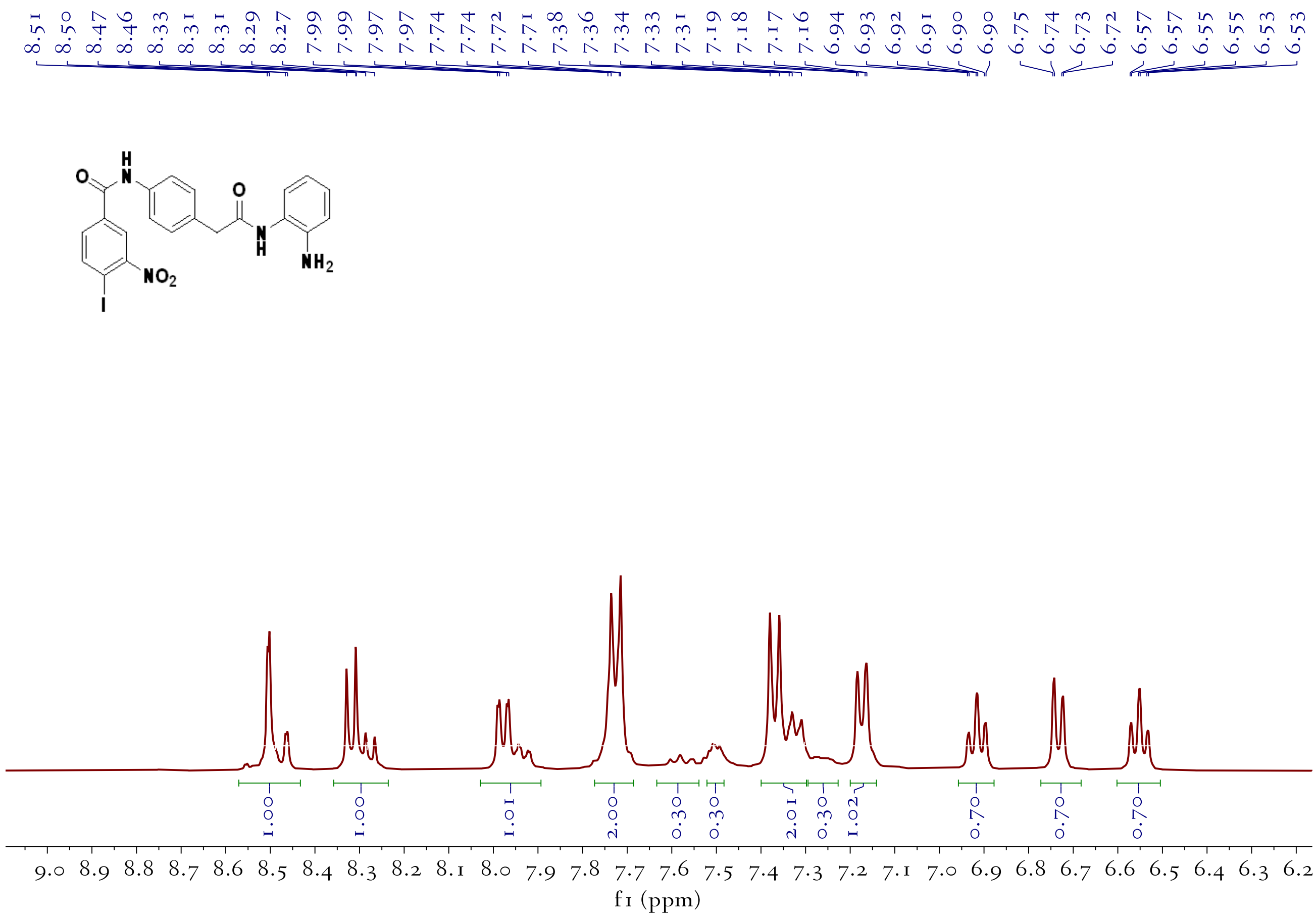

Figure S56: aromatic expansion of compound **7h**

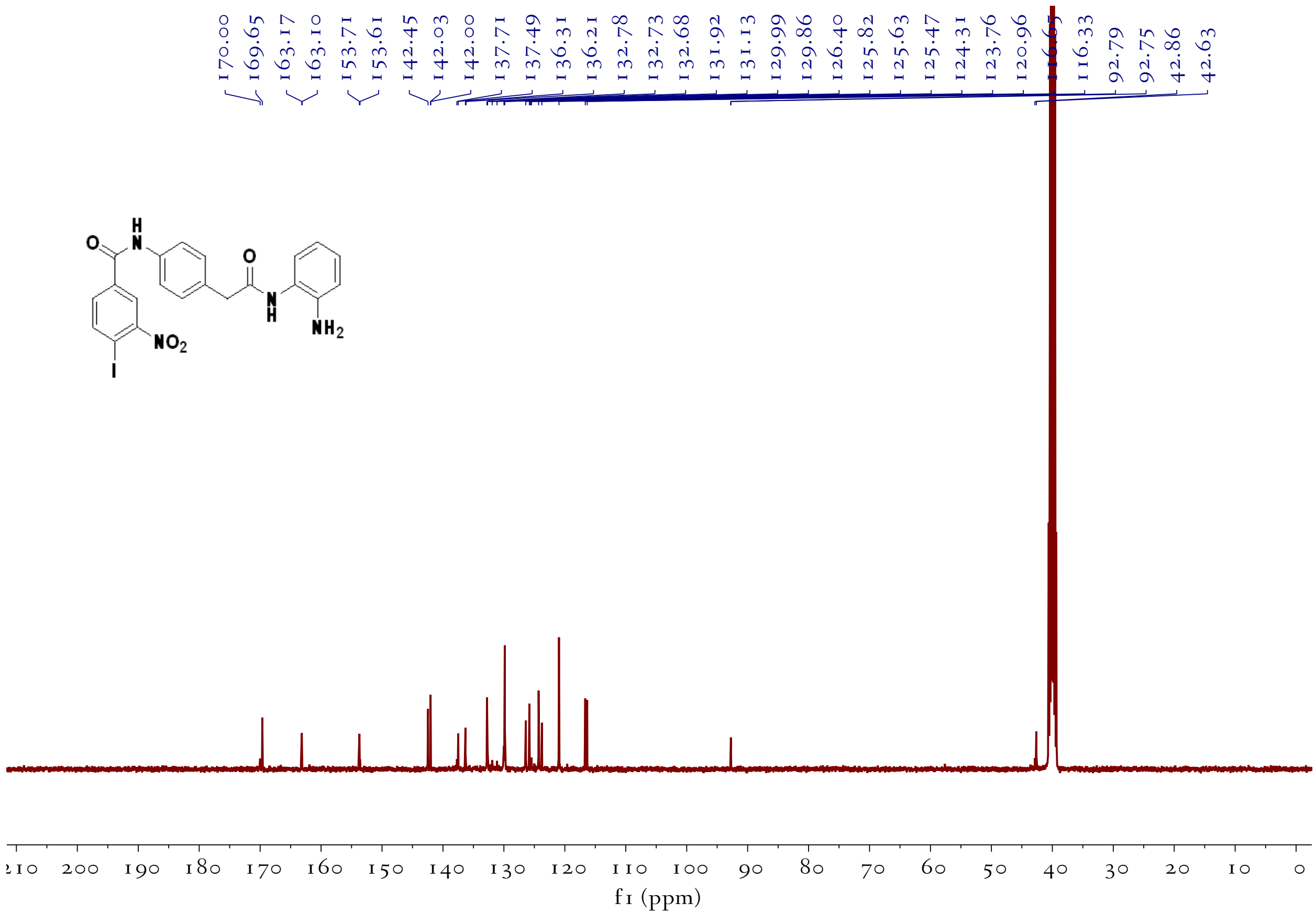

Figure S57: 13C NMR (100 MHz, DMSO-d_6) of compound **7h**

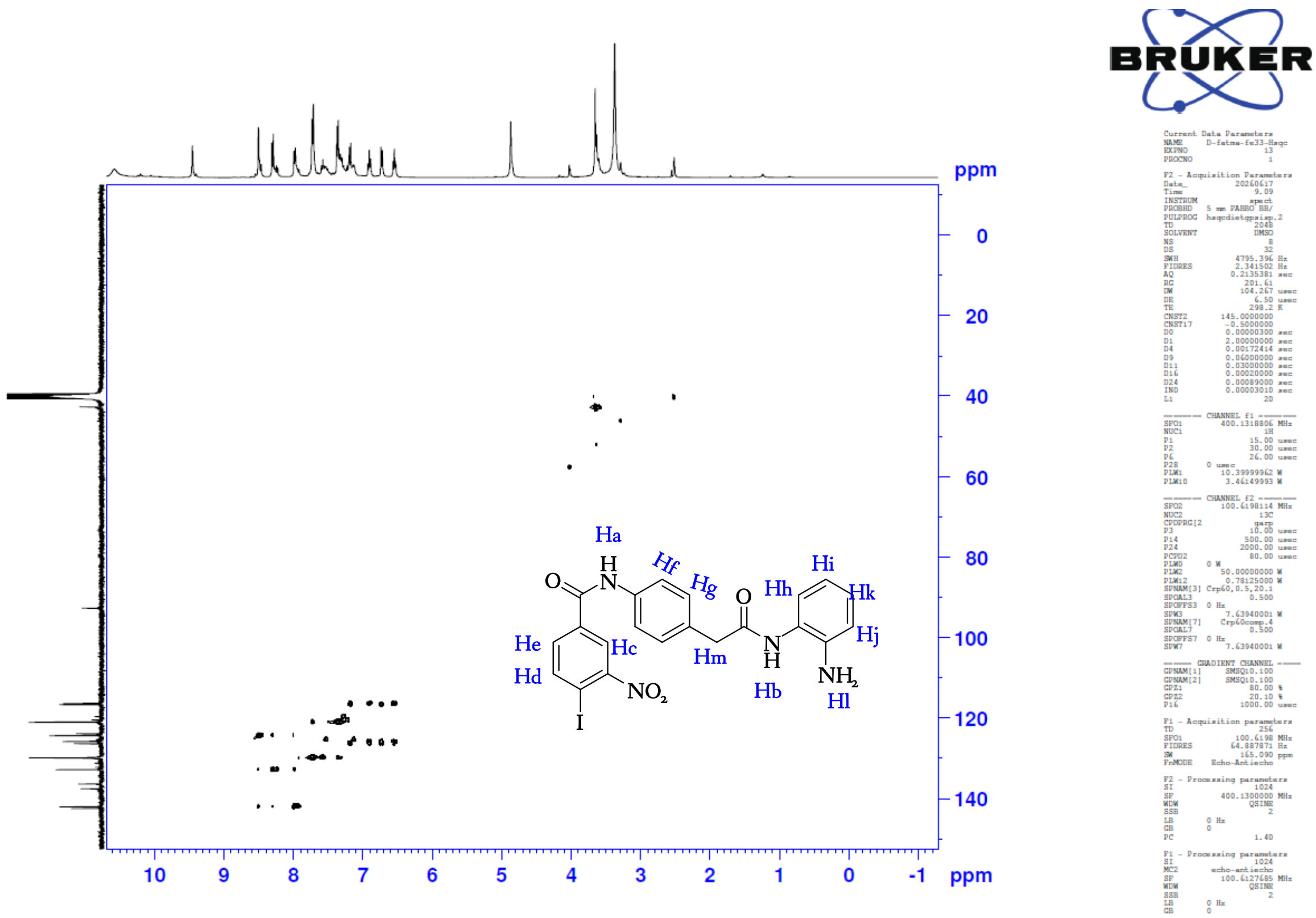

Figure S58: ^{1}H-^{13}C HSQC spectrum of compound **7h**

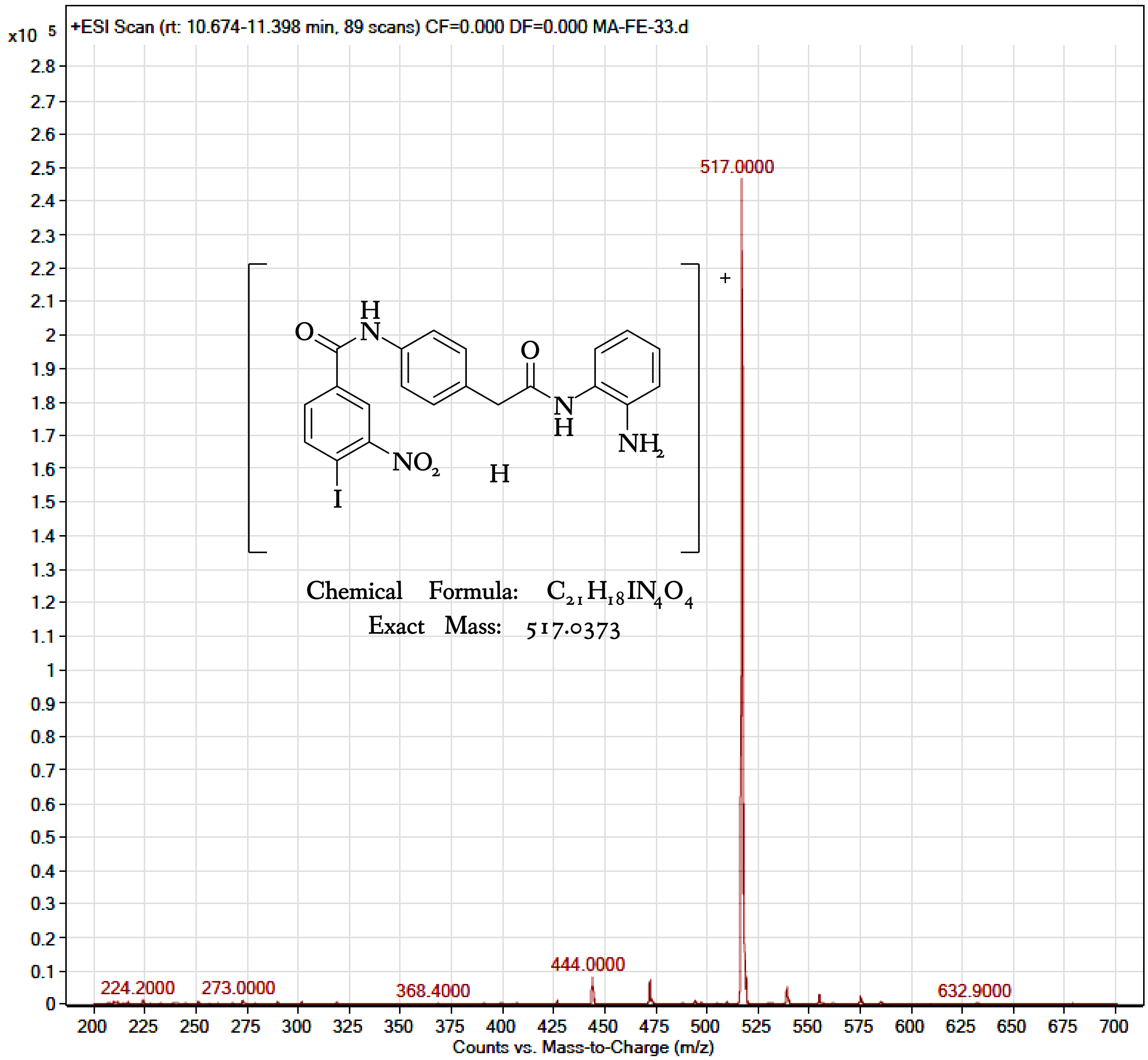

Figure S59: HRMS-ESI Spectrum of compound **7h**

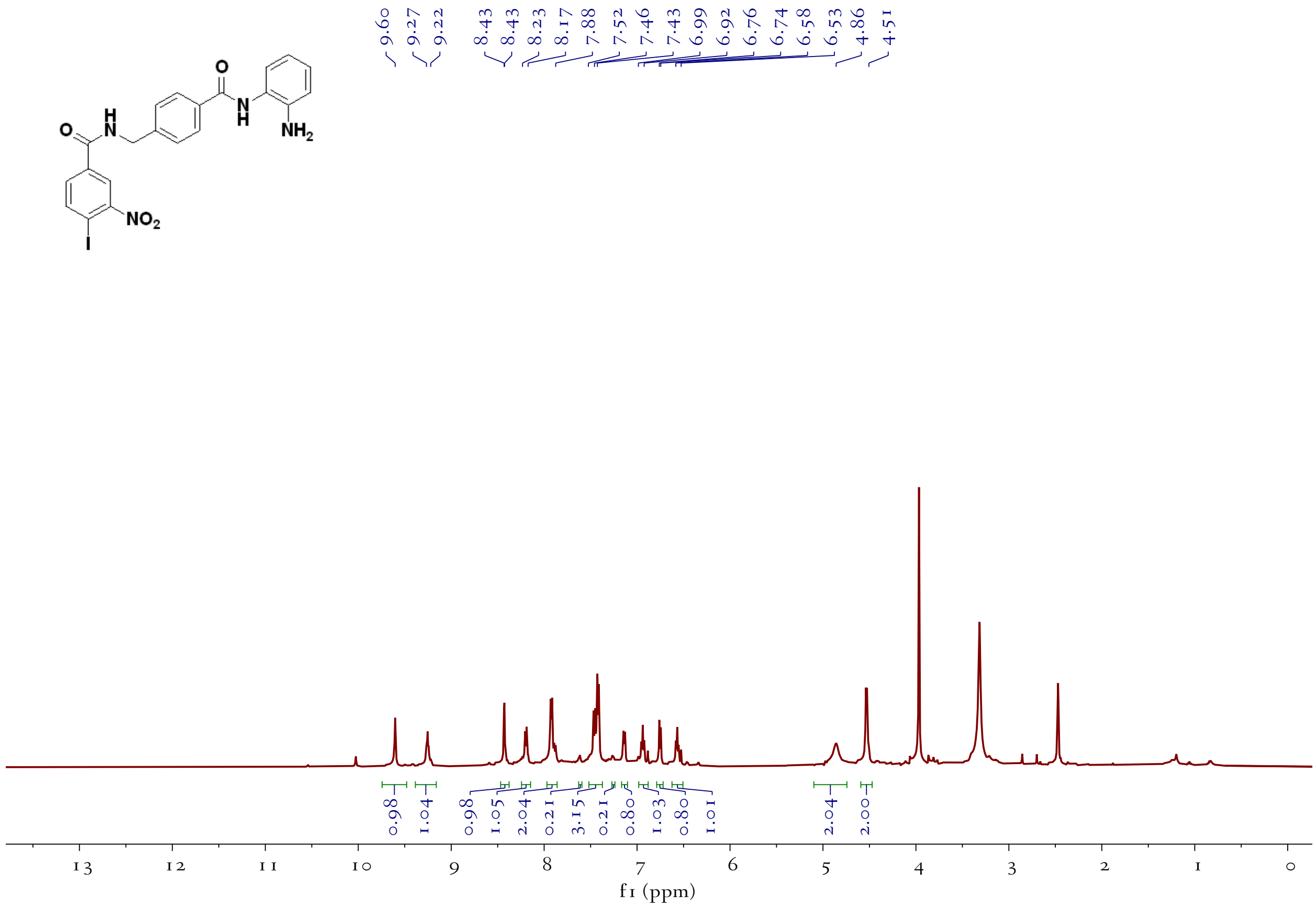

Figure S60: ^{1}H NMR (500 MHz, DMSO-d_6) of compound **7i**

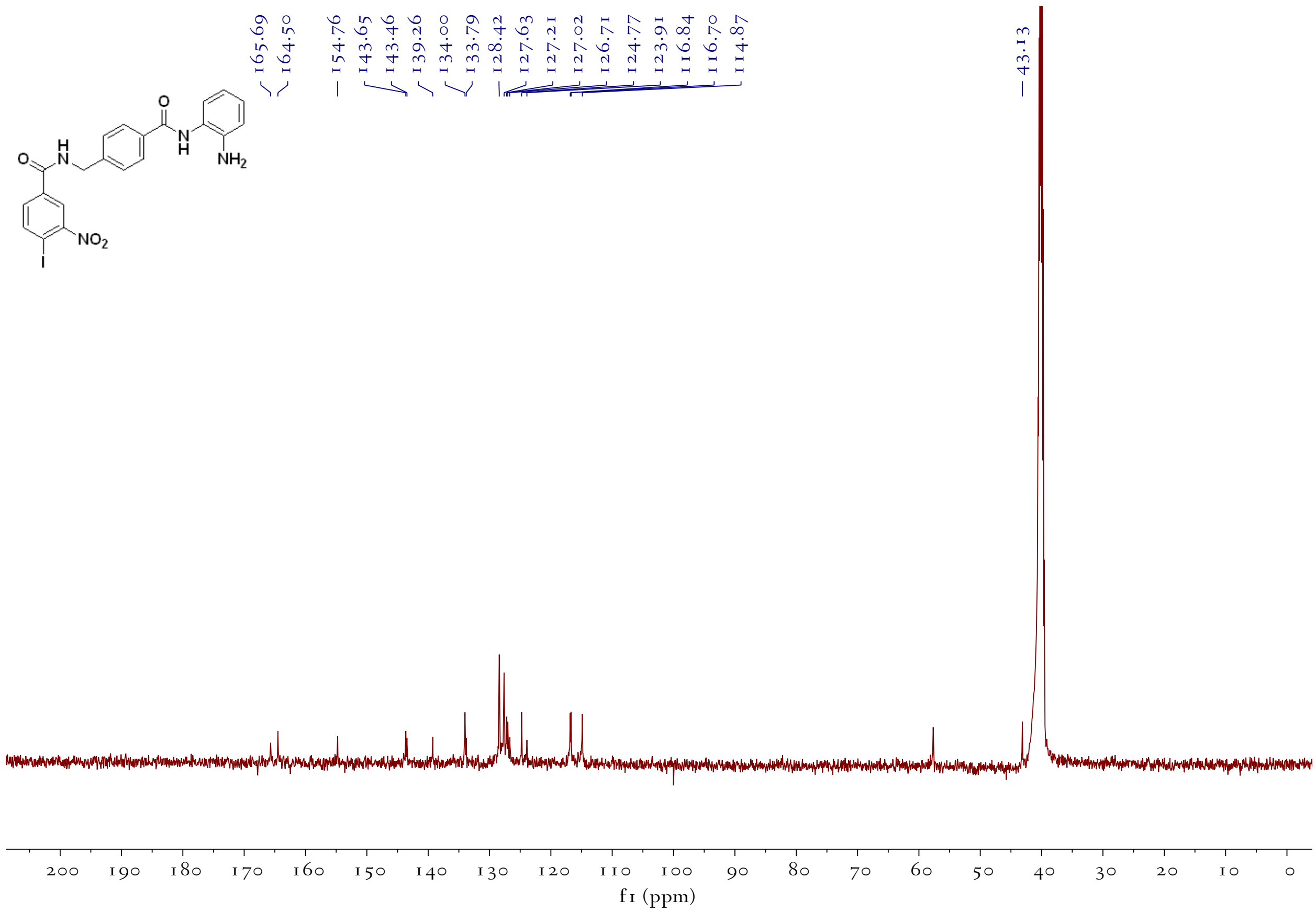

Figure S61: ^{13}C NMR (125 MHz, DMSO-d_6) of compound **7i**

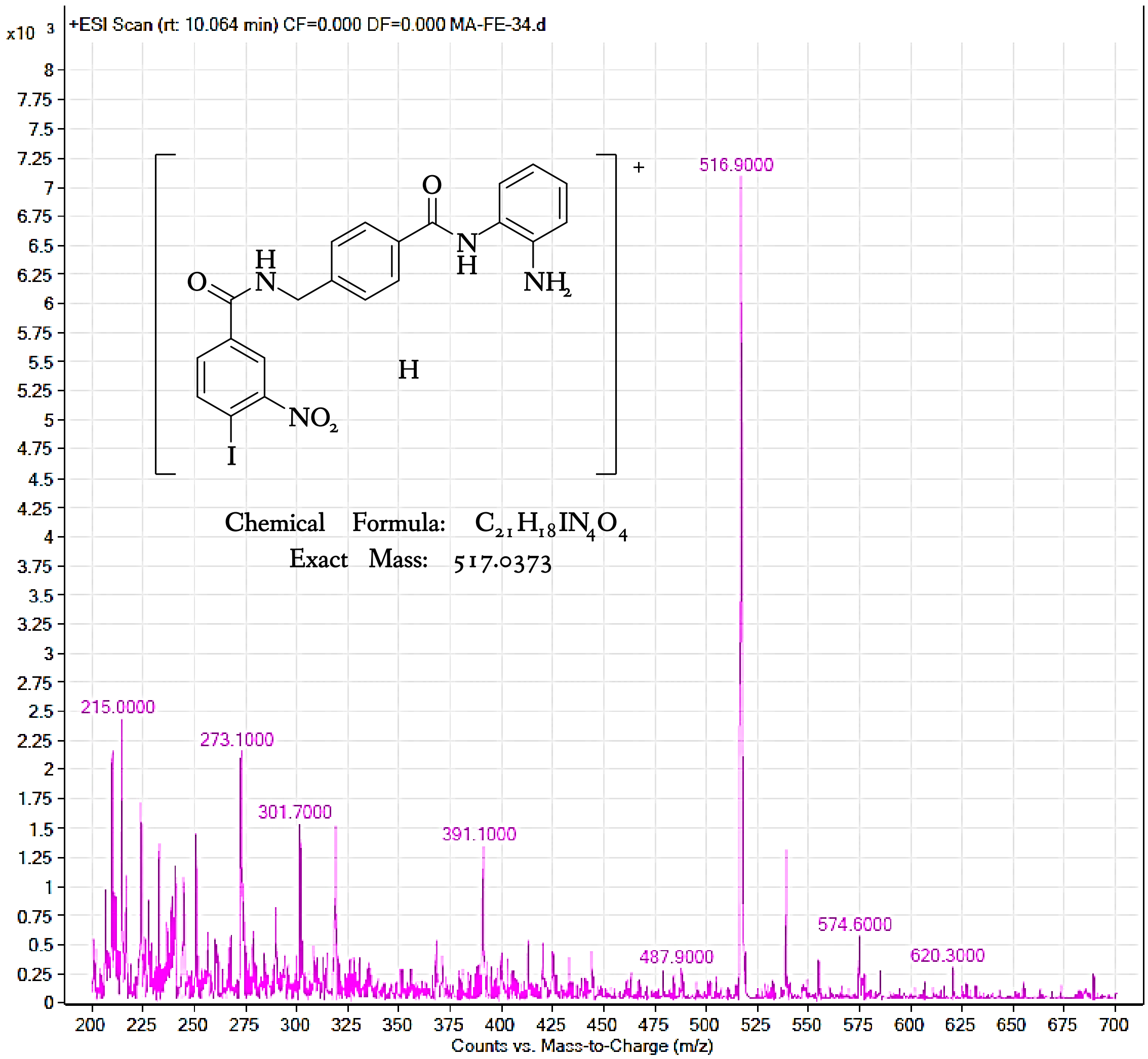

Figure S62: HRMS-ESI Spectrum of compound **7i**

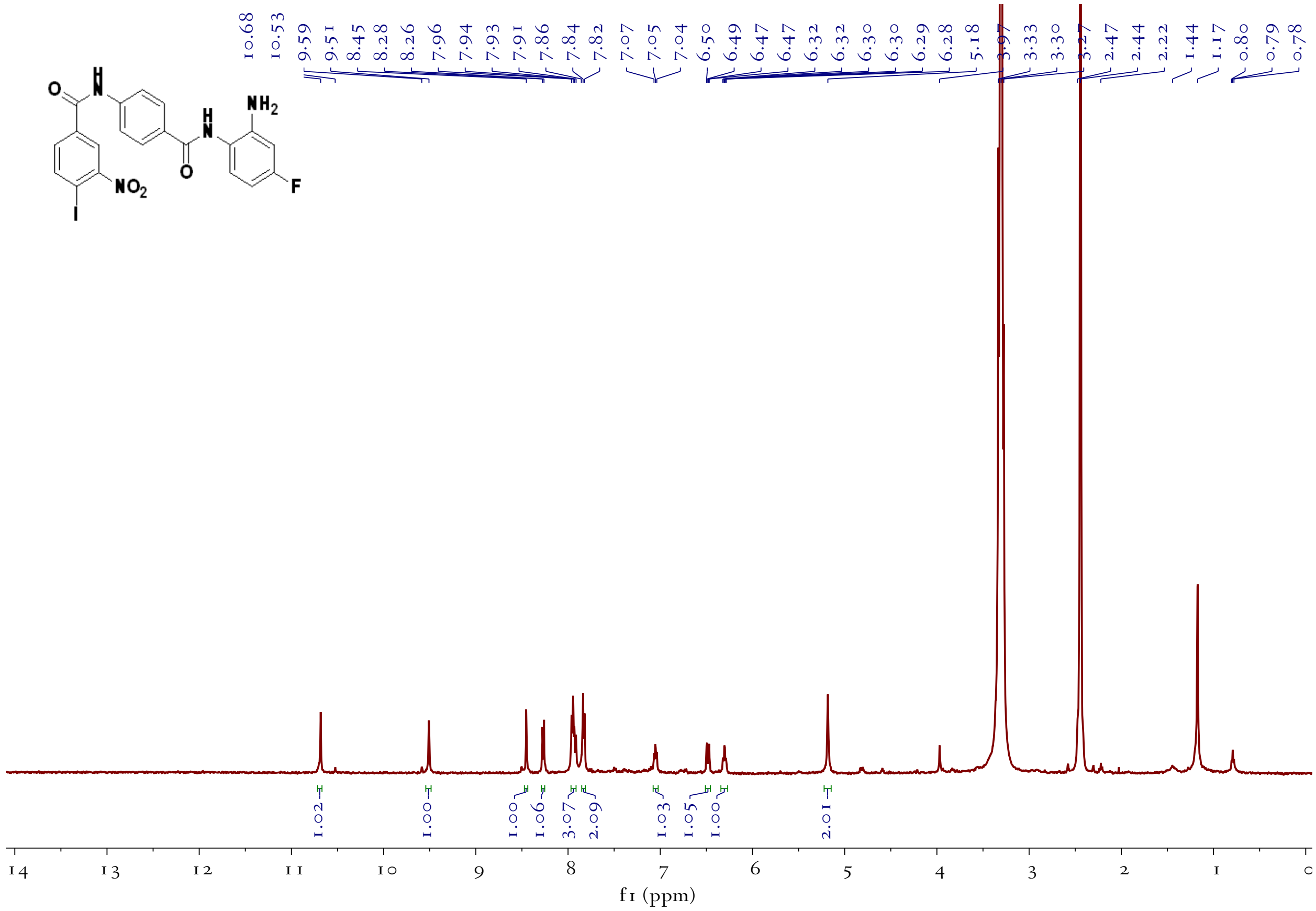

Figure S63: ^{1}H NMR (500 MHz, DMSO-d_6) of compound **7j**

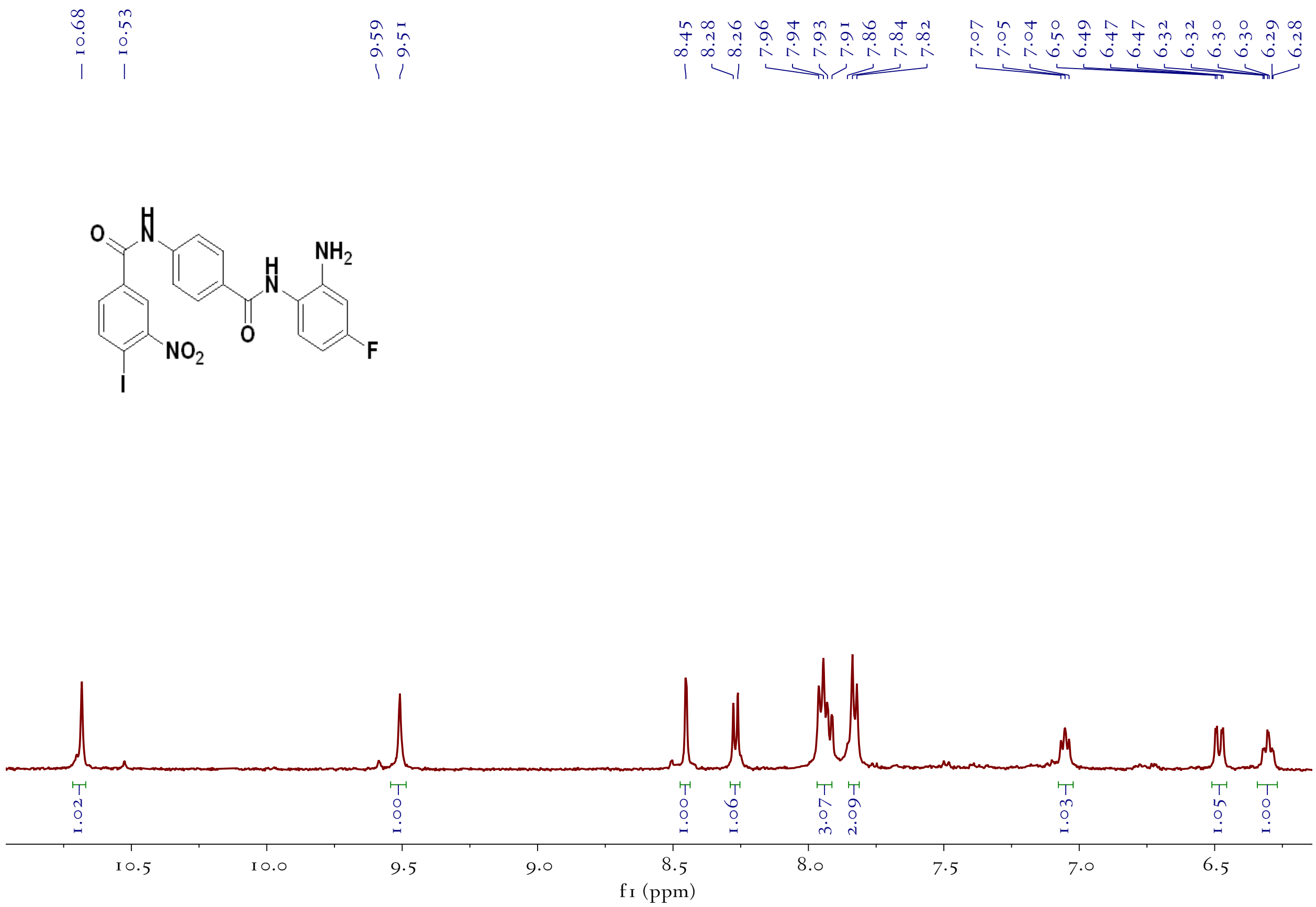

Figure S64: ^{1}H NMR expansion (500 MHz, DMSO-d_6) of compound **7j**

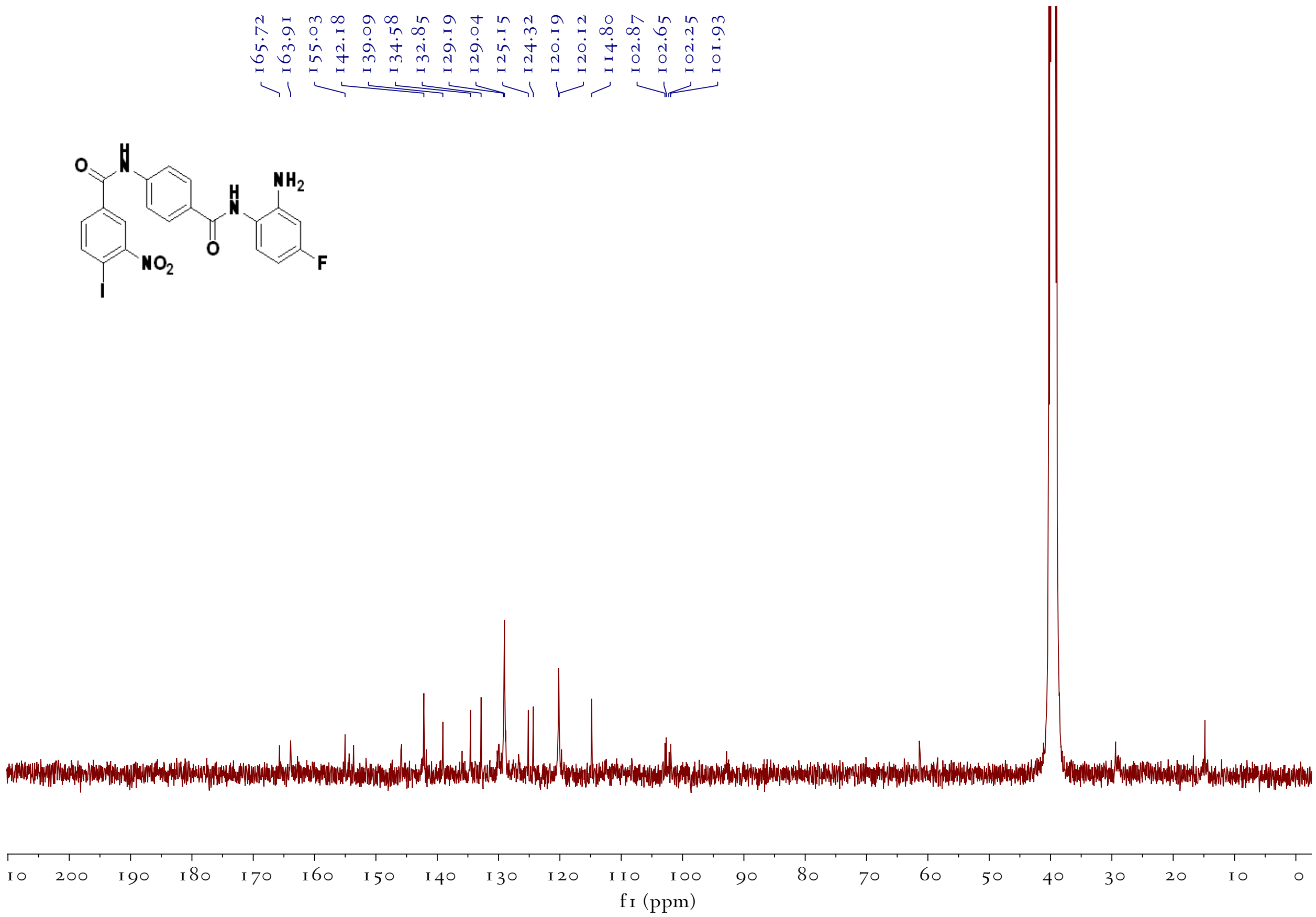

Figure S65: ^{13}C NMR (400 MHz, DMSO-d_6) of compound **7j**

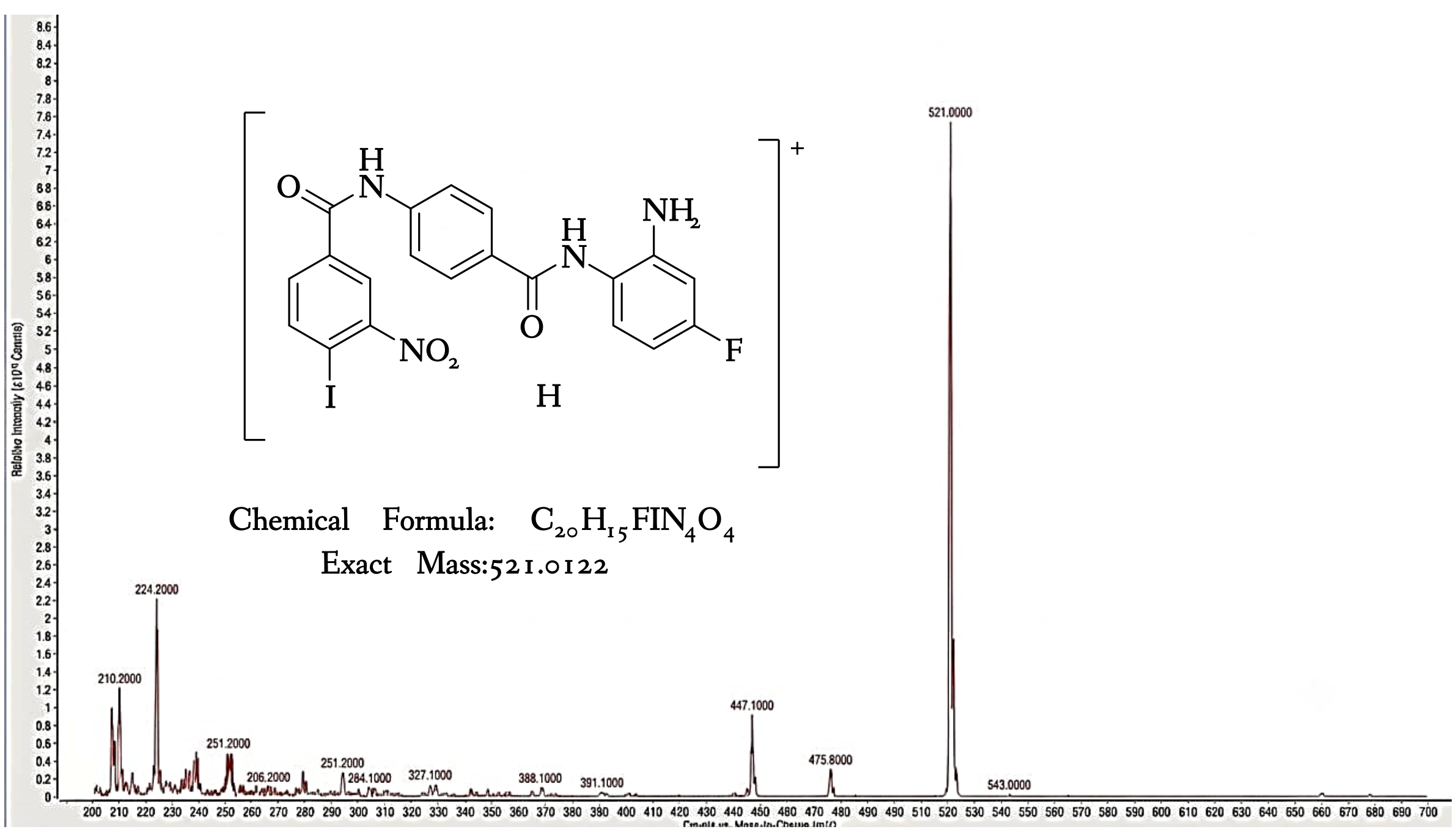

Figure S66: HRMS-ESI Spectrum of compound **7j**

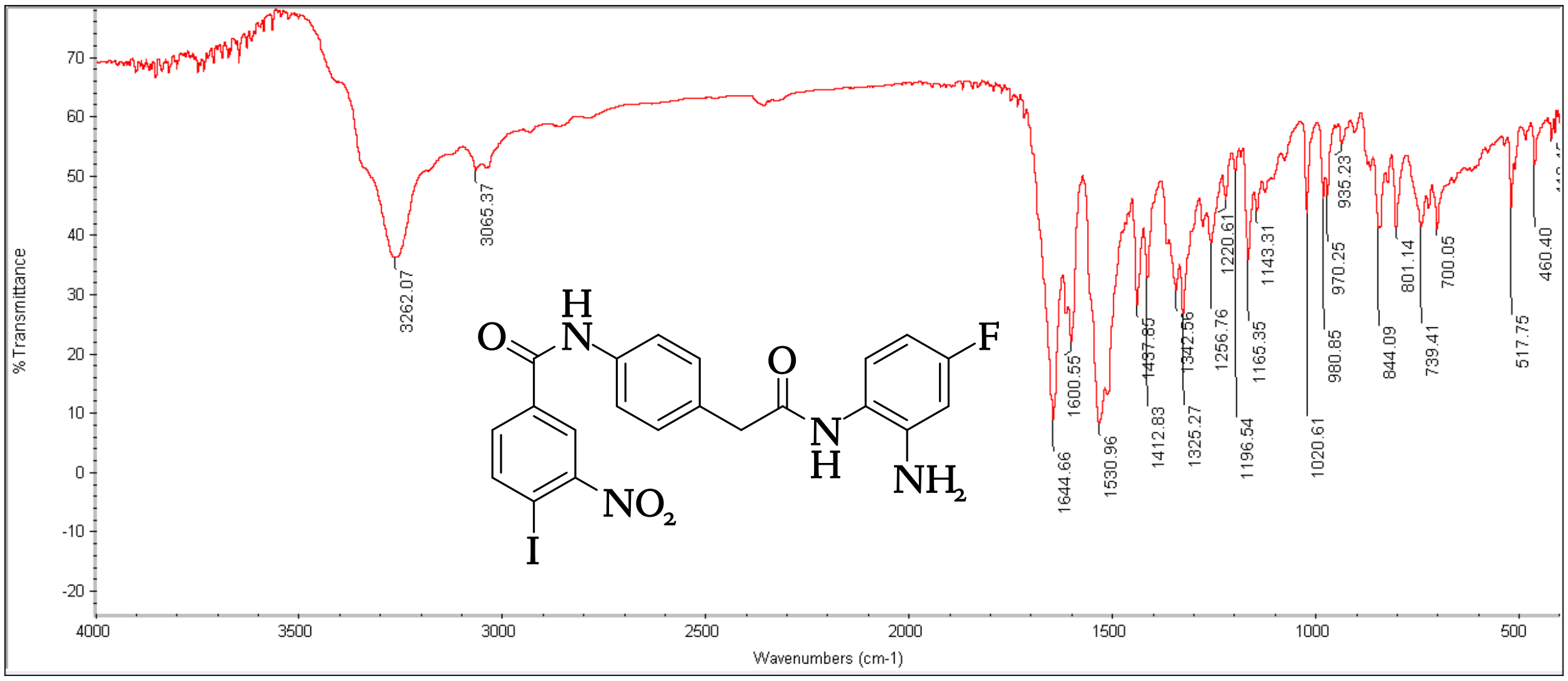

Figure S67: IR spectrum of compound **7k**

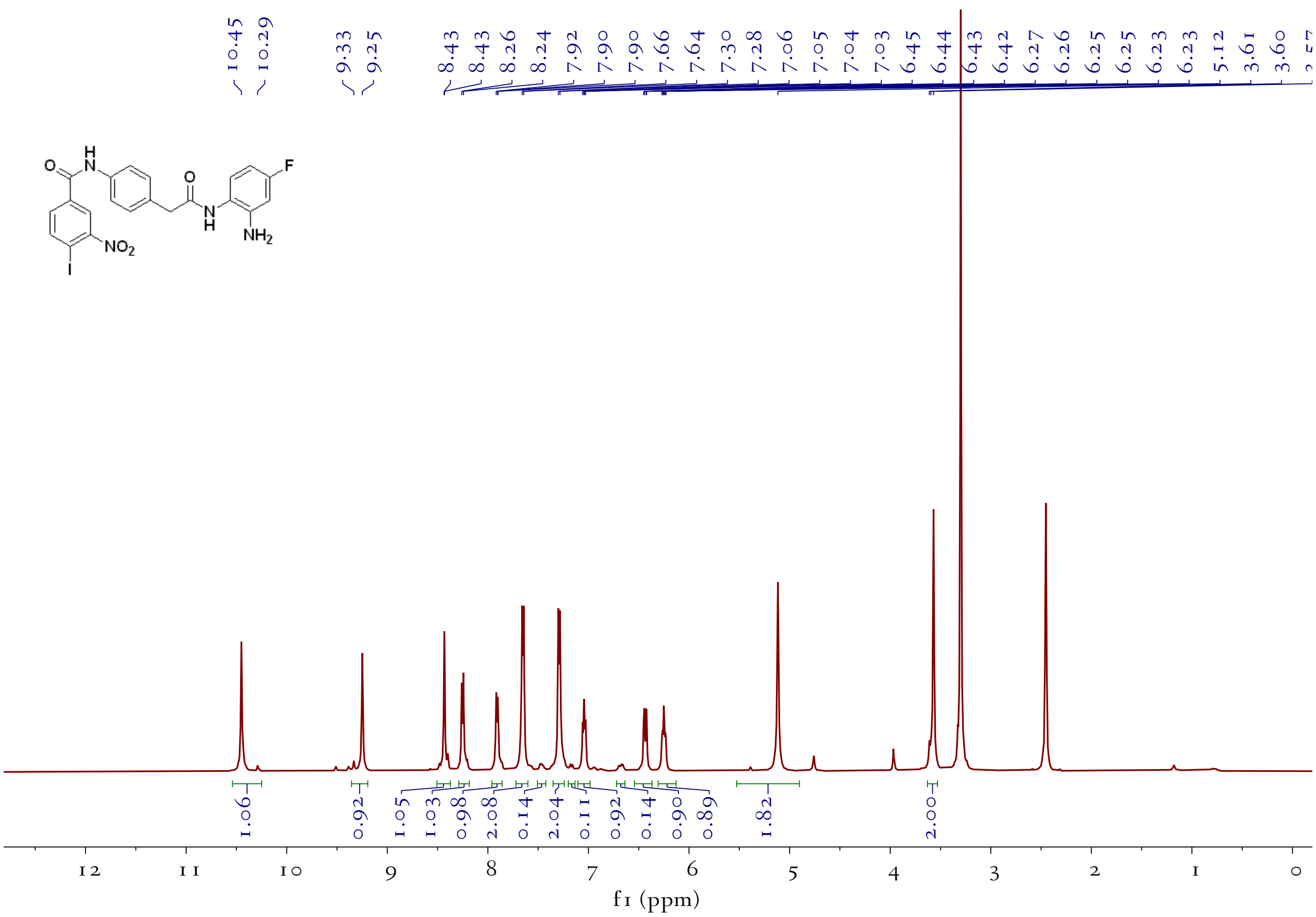

Figure S68: ^{1}H NMR (400 MHz, DMSO-d_6) of compound **7k**

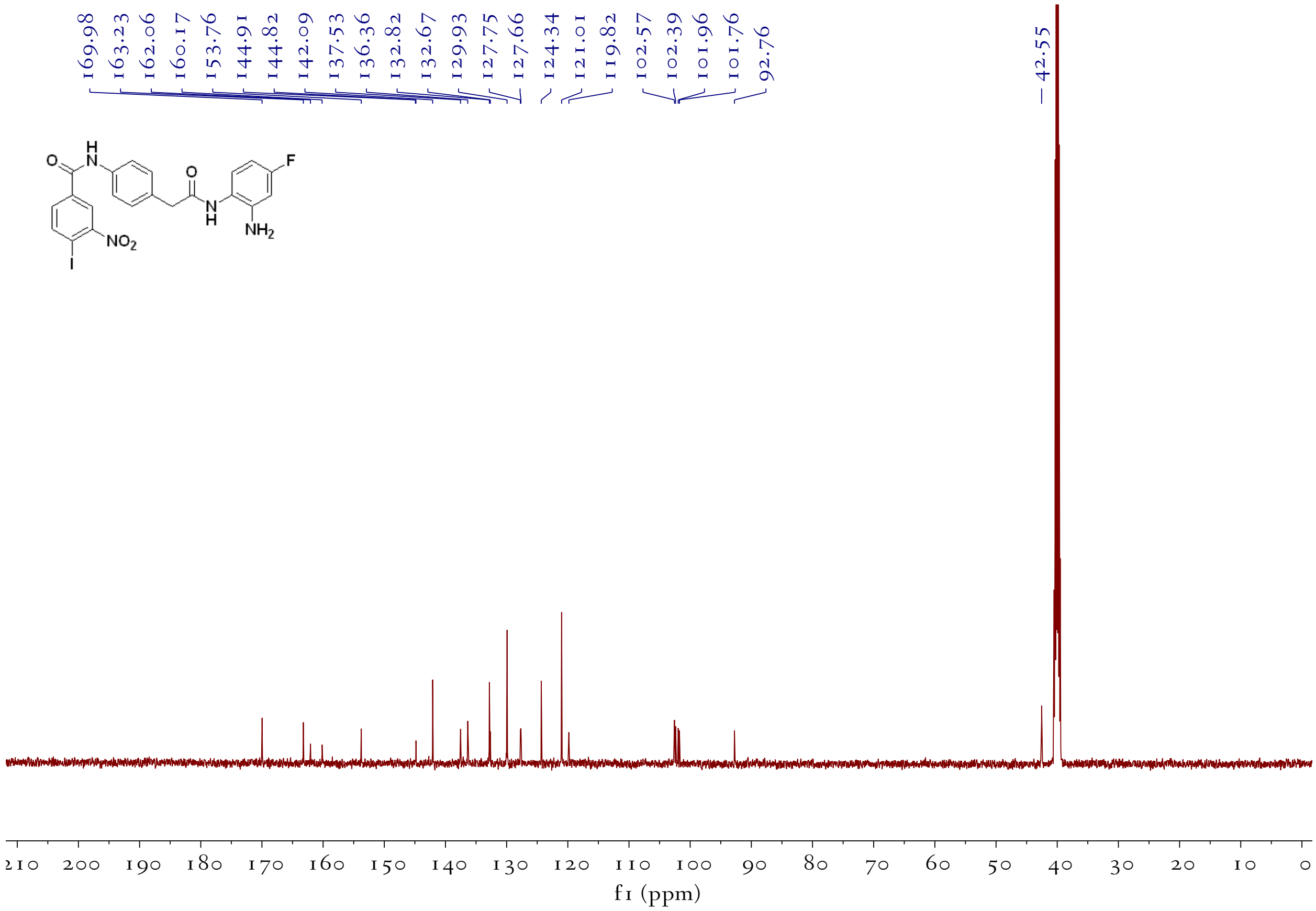

Figure S69: ^{13}C NMR (100 MHz, DMSO-d_6) of compound **7k**

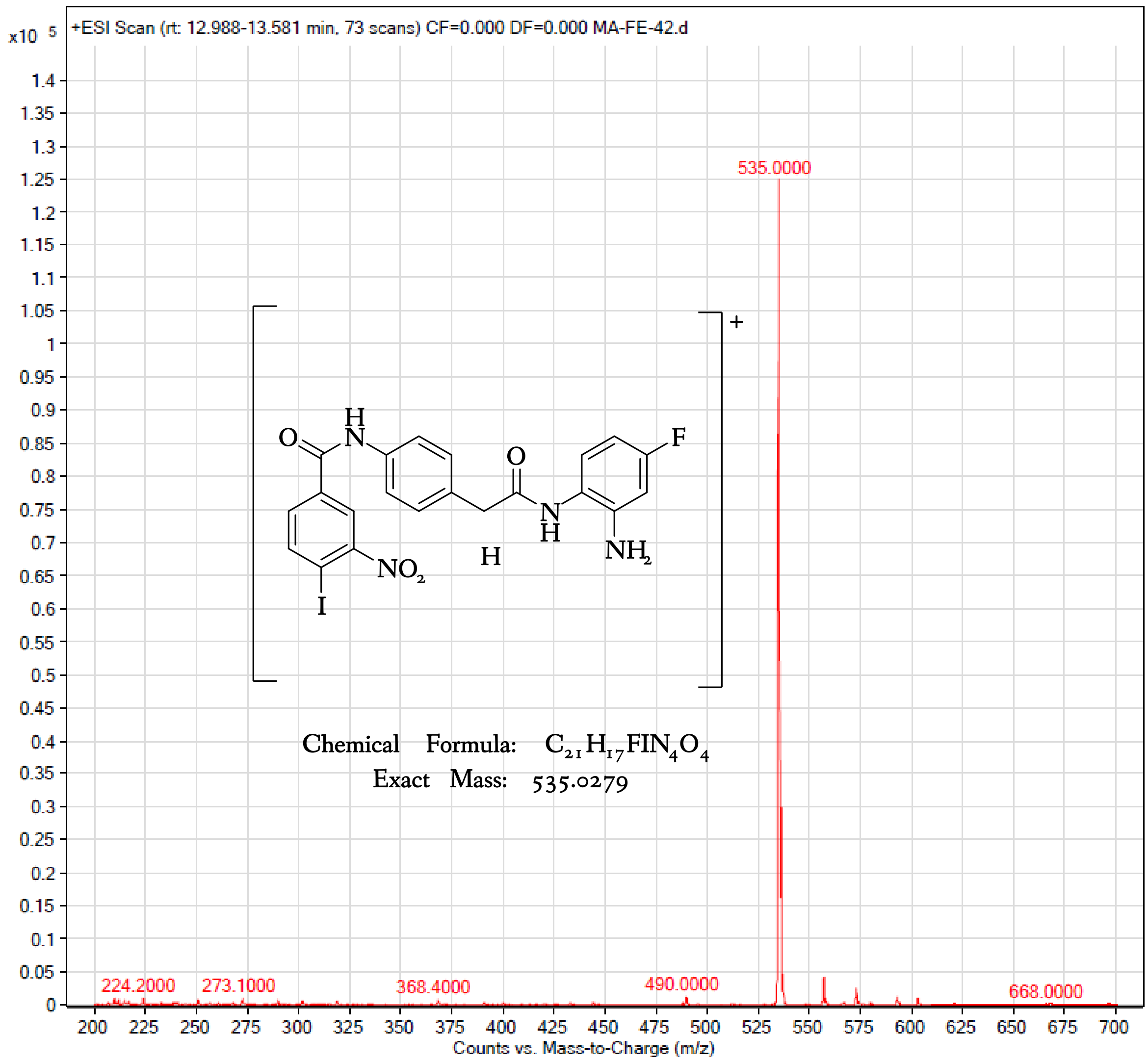

Figure S70: HRMS-ESI Spectrum of compound **7k**

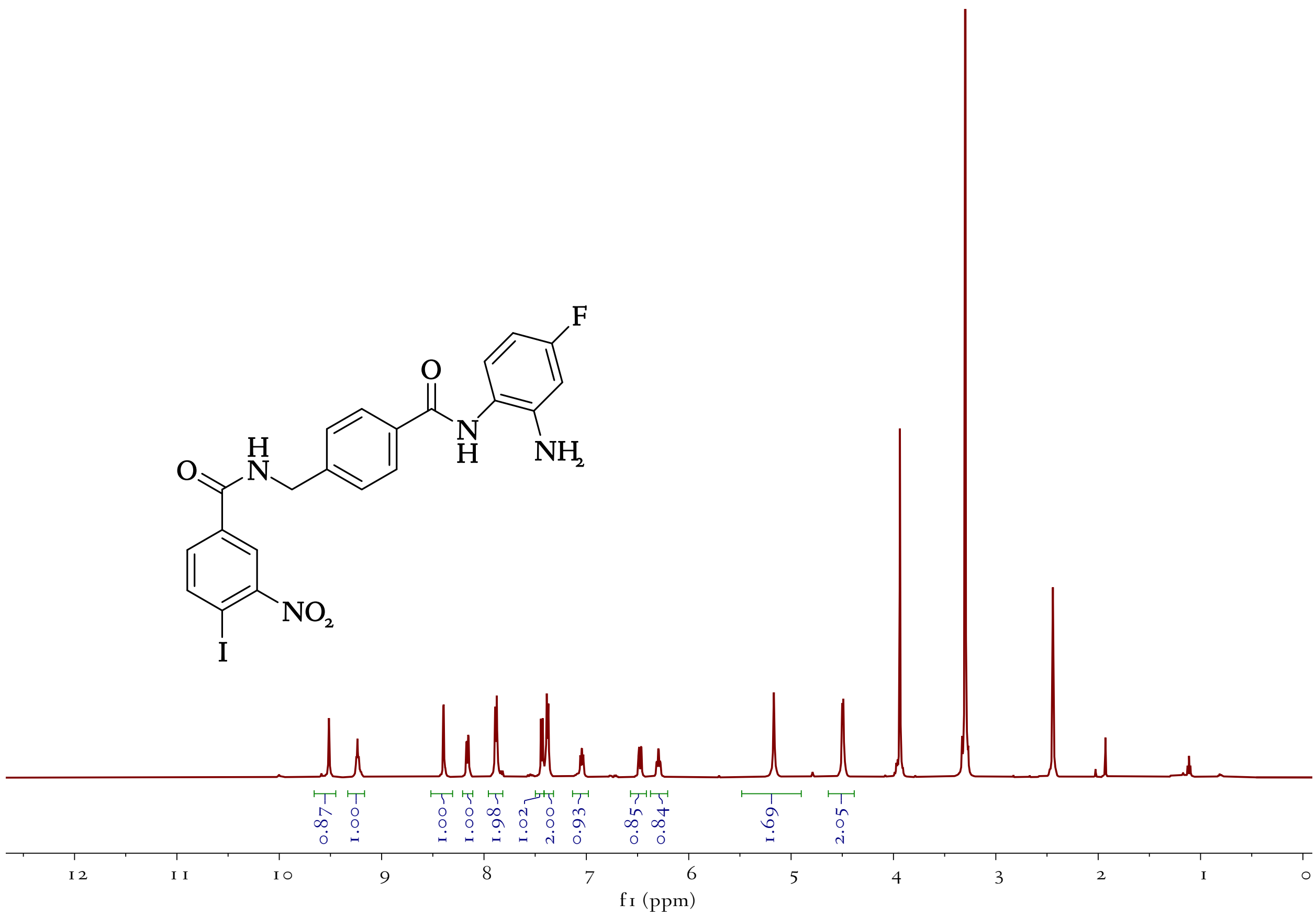

Figure S71: ^{1}H NMR (500 MHz, DMSO-d_6) of compound **7l**

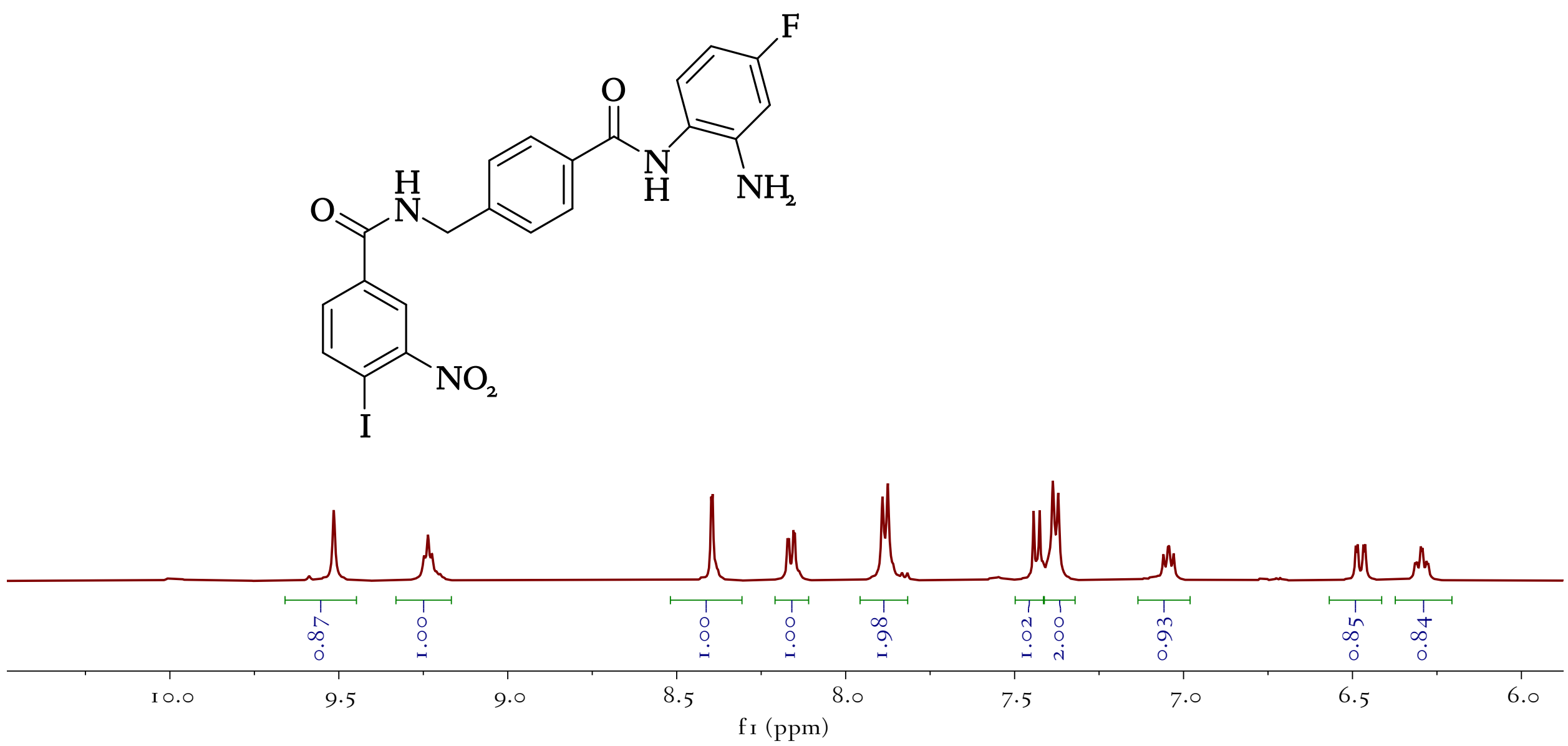

Figure S72: ¹H NMR expansion (500 MHz, DMSO-d_6) of compound **7l**

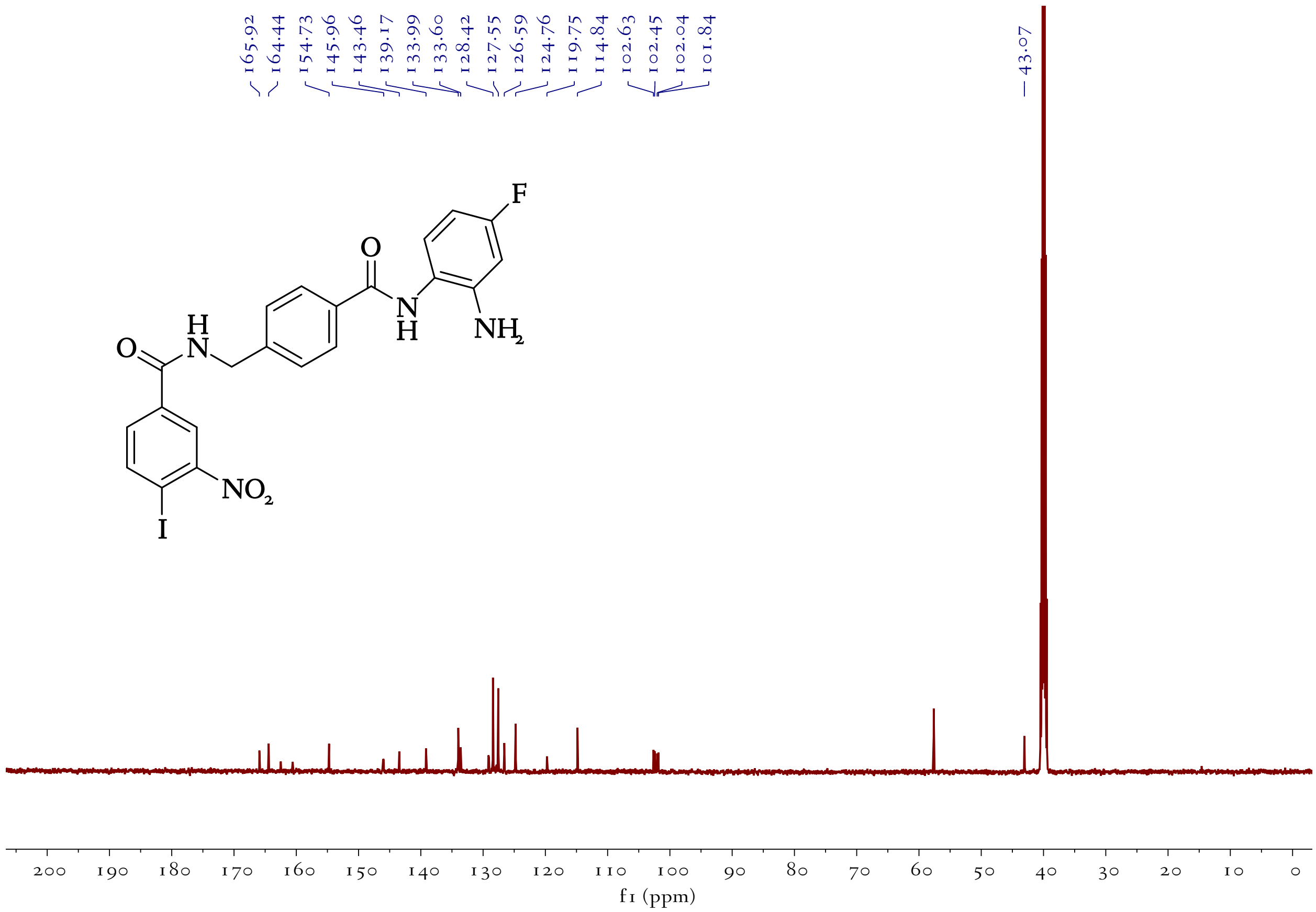

Figure S73: ^{13}C NMR (125 MHz, DMSO-d_6) of compound **7l**

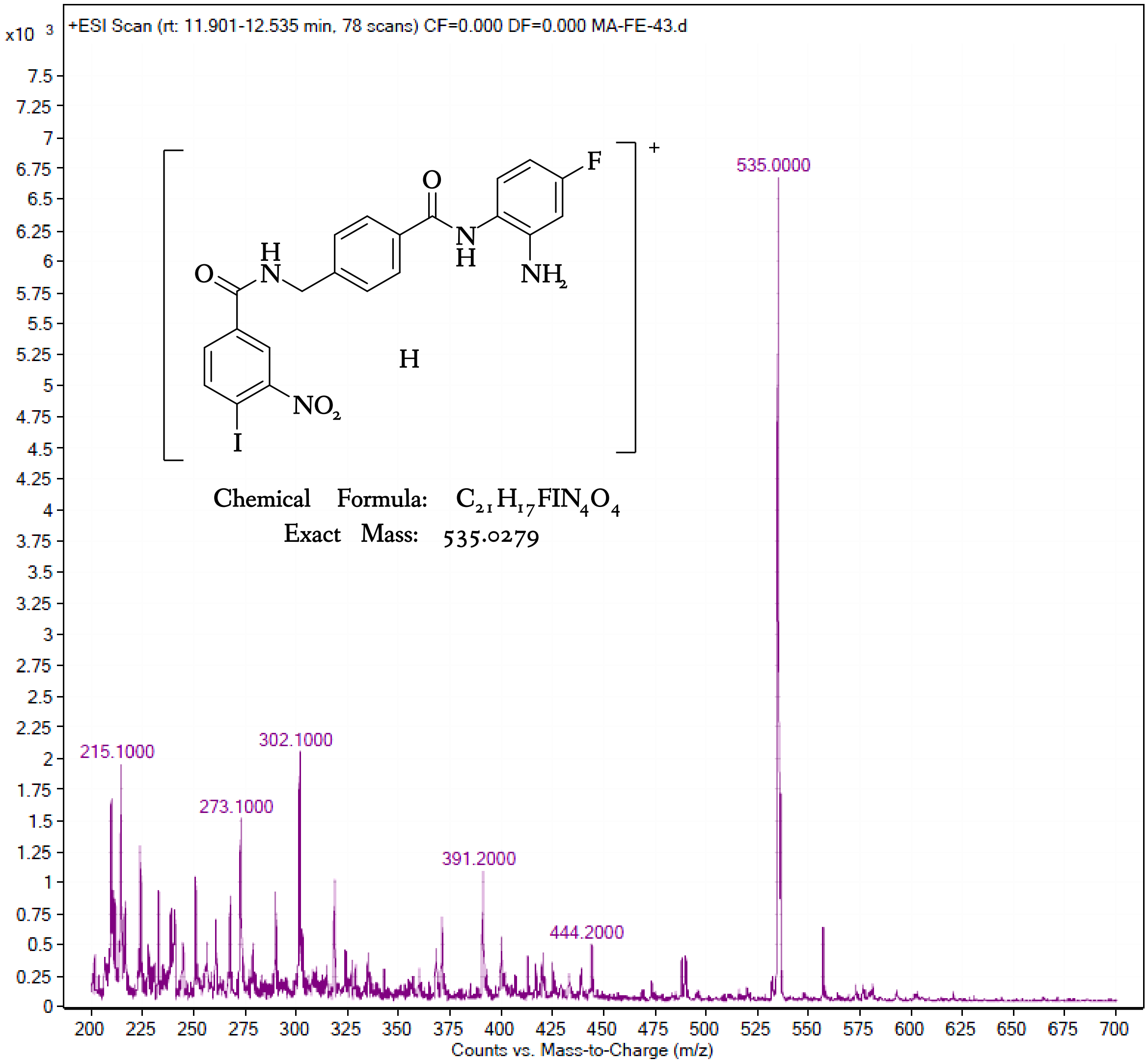

Figure S74: HRMS-ESI Spectrum of compound **7l**

Elemental microanalysis of compounds 5a-c, 6a-c, 7a-l

جامعة الأزهر
Al-Azhar University
المركز الإقليمي للفطريات وتطبيقاتها
The Regional Center for Mycology and Biotechnology

Requester Data:

Name: Dr. Fatma Elzahraa Ali Mohamed

Authority: Faculty of Pharmacy, Assuit University

Sample Data:

Eighteen samples had been submitted for elemental analysis.

Analysis Report:

Sample Code	C%	H%	N%
**FE 04**	**43.82**	**3.17**	**6.60**
**FE 06**	**46.40**	**2.85**	**10.98**
**FE 07**	**39.78**	**2.76**	**13.41**
**FE 08**	**41.07**	**2.43**	**7.04**
**FE 10**	**39.58**	**2.49**	**10.12**
**FE 12**	**43.79**	**3.17**	**6.54**
**FE 14**	**48.11**	**3.23**	**11.42**
**FE 15**	**41.22**	**2.87**	**13.01**
**FE 16**	**43.91**	**3.19**	**6.52**
**FE 17**	**41.09**	**2.86**	**9.79**
**FE 18**	**42.47**	**2.83**	**6.81**
**FE 19**	**41.20**	**3.17**	**13.02**
**FE 20**	**41.09**	**2.95**	**9.71**
**FE 21**	**42.14**	**2.71**	**6.83**
**FE 33**	**49.17**	**3.46**	**11.08**
**FE 34**	**49.18**	**3.44**	**11.09**
**FE 42**	**47.42**	**3.19**	**23.63**
**FE 43**	**47.43**	**3.21**	**10.75**

Head of the Lab

Dr. M. Mourad

Prof. Dr. Mohamed Mourad

11/8/2025

جامعة الأزهر
AL-AZHAR UNIVERSITY
المركز الإقليمي للفطريات

DIRECTOR

M. Mansour

12.8.202-

Prof. Dr. Mohamed Mansour

تليفون: ٢٢٦٢٠٣٧٣ (٠٢) فاكس: ٢٢٦٢٠٣٧٣ (٠٢)
شارع المخيم الدائم - مدينة نصر - القاهرة
http://www.azhar.edu.eg.htm
البريد الإلكتروني: rcmb@azhar.edu.eg
http://www.azhar.edu.eg./pages/fungi_center.htm
صندوق بريد ١١٧٥١ مدينة نصر القاهرة
Facebook: RCMB AZHAR

Experimental code	Manuscript code
Fe04	5a
Fe12	5b
Fe16	5c
Fe08	6a
Fe18	6b
Fe21	6c
Fe10	7a
Fe17	7b
Fe20	7c
Fe07	7d
Fe15	7e
Fe19	7f
Fe14	7g
Fe33	7h
Fe34	7i
Fe06	7j
Fe42	7k
Fe43	7l

Appendix A

4. EXPERIMENTAL

4.1. Chemistry

Materials and methods

All reagents and solvents were of general purpose or analytical grade and purchased from Sigma Aldrich Ltd, Fisher Scientific, Fluka and Acros. ^{1}H and ^{13}C NMR spectra were recorded with a Bruker Avance III spectrometer operating at 400, 100 MHz respectively, with Me_4Si as internal standard and DMSO-d6 as a solvent. Elemental analysis was performed by the regional center for mycology and biotechnology (Cairo, Egypt). TLC was carried out on precoated silica plates (Keisel gel 60 F254, BDH) using Hexane: Ethyl acetate, 1 : 2, v/v. Compounds were visualized by illumination under UV light (254 nm). Melting points were determined on an electrothermal instrument and are uncorrected. All solvents were dried prior to use and stored over 4 Å molecular sieves, under nitrogen. All the compounds were ≥ 95% pure.

4.2. Biological evaluation

4.2.1 Cell Viability assay (MTT assay)

MTT assay was performed to investigate the effect of the synthesized compounds on mammary epithelial cells (MCF-10A). The cells were propagated in medium consisting of Ham's F-12 medium/

Dulbecco's modified Eagle's medium (DMEM) (1:1) supplemented with 10% foetal calf serum (GIBCO, UK), 2 mM glutamine, insulin (10 μg/mL), hydrocortisone (500 ng/mL) and epidermal growth factor (20 ng/mL). Trypsin ethylenediamine tetra acetic acid (EDTA) was used to passage the cells after every 2-3 days. 96-well flat-bottomed cell culture plates were used to seed the cells at a density of 104 cells mL^{-1}. The medium was aspirated from all the wells of culture plates after 24 h followed by the addition of synthesized compounds (in 200 μL medium to yield a final concentration of 0.1% (v/v) dimethyl sulfoxide) into individual wells of the plates. Four wells were designated to a single compound. The plates were allowed to incubate at 37°C for 96 h. Afterwards, the medium was aspirated and 3-[4,5-dimethylthiazol-2-yl]-2,5-diphenyl tetrazolium bromide (MTT) (0.4 mg/mL) in medium was added to each well and subsequently incubated for 3 h. The medium was aspirated and 150 μL dimethyl sulfoxide (DMSO) was added to each well. The plates were vortexed followed by the measurement of absorbance at 540 nm on a microplate reader. The results were presented as inhibition (%) of proliferation in contrast to controls comprising 0.1% DMSO.

4.2.2. Growth inhibitory assay

To explore the antiproliferative potential of compounds propidium iodide fluorescence assay was performed using different cell lines such as, MDA-MB-231 (triple-negative breast cancer), A-549 (non-small cell lung cancer), and SEM (B-cell acute lymphoblastic leukemia) cell lines. All cell lines were obtained from ATCC (American Type Cell Culture). To calculate the total nuclear DNA, a fluorescent dye (propidium iodide, PI) is used which can attach to the DNA, thus offering a quick and precise

technique. PI cannot pass through the cell membrane, and its signal intensity can be considered as directly proportional to quantity of cellular DNA. Cells whose cell membranes are damaged or have changed permeability are counted as dead ones. The assay was performed by seeding the cells of different cell lines at a density of 3000-7500 cells/well (in 200 μl medium) in culture plates followed by incubation for 24 h at 37 °C in humidified 5% CO_2 /95% air atmospheric conditions. The medium was removed; the compounds were added to the plates at 10 μM concentrations (in 0.1% DMSO) in triplicates, followed by incubation for 48 h. DMSO (0.1%) was used as control. After incubation, medium was removed followed by the addition of PI (25 μl, 50 μg/mL in water/medium) to each well of the plates. At -80 °C, the plates were allowed to freeze for 24 h, followed by thawing at 25 ^{o}C. A fluorometer (Polar-Star BMG Tech) was used to record the readings at excitation and emission wavelengths of 530 and 620 nm for each well. The percentage cytotoxicity of compounds was calculated using the following formula:

$$\% \text{ Cytotoxicity} = \frac{A_C - A_{TC}}{A_C} \times 100$$

Where A*TC*= Absorbance of treated cells and AC= Absorbance of control. Vorinostat was used as positive control in the assay.

4.2.3. Enzymatic (HDAC) Evaluation

The enzymatic assays to evaluate the HDAC inhibitory effects of all final compounds against human recombinant HDAC-1, HDAC-2, HDAC-3, and HDAC-8 isoforms were carried out using

Fluorogenic HDAC Assay Kits purchased from BPS Bioscience (San Diego, CA, USA), following the manufacturer's instructions. The exact same assay conditions, buffer, and incubation protocols were applied uniformly across all four tested isoforms. All final compounds were initially screened at a single concentration of 10 μM. For the most potent derivatives, IC_{50} values were determined, using vorinostat (SAHA) as the positive reference drug control. All test compounds were dissolved in 100% DMSO to prepare stock solutions. A serial dilution of the compounds was performed in 100% DMSO, with the highest concentration at 1 mM. Each intermediate dilution was subsequently diluted 10-fold in the provided proprietary HDAC Assay Buffer (BPS Bioscience; composed of 25 mM Tris/HCl, pH 8.0, 137 mM NaCl, 2.7 mM KCl, 1 mM MgCl2 , and 0.1 mg/mL BSA) to form a 10% DMSO intermediate. A 5 μL aliquot of this solution was added to a 50 μL final reaction volume, ensuring a constant final DMSO concentration of 1% (v/v) across all assay wells to eliminate solvent-induced artifacts.

The reactions were initiated by mixing the respective human recombinant enzymes with the appropriate fluorogenic acetylated substrates (Fluorogenic HDAC Substrate 3 for HDAC-1, -2, -3; Fluorogenic HDAC Substrate Class 2A for HDAC-8). The enzyme-substrate mixture was incubated at 37 °C for 30 minutes. The deacetylation reaction was subsequently terminated by adding an equal volume (50 μL) of 2× HDAC Developer solution (BPS Bioscience), followed by an additional incubation at room temperature for 15 minutes.

Fluorescence intensity was measured at an excitation wavelength of 360 nm and an emission wavelength of 460 nm using a Tecan Infinite M1000 microplate reader. To ensure statistical rigor and

reproducibility, the assay was performed as three independent biological replicates (n = 3), with each experiment executed in technical triplicate.

The percentage of enzyme activity was calculated using the formula:

% activity = (F- Fb) / (Ft - Fb)

Where □ is the fluorescence intensity in the presence of the test compound, □□ is the total enzyme activity control (absence of inhibitor, representing 100% activity), and □□ is the blank background control (absence of HDAC enzyme, representing 0% activity). The percentage of inhibition was determined as:

% Inhibition=100−% Activity

Statistical Analysis and IC_{50} Determination

IC_{50} values were calculated from dose-response curves generated by fitting the experimental data to a non-linear regression curve. All data were analyzed using GraphPad Prism 5 software (GraphPad Software, San Diego, CA, USA) and presented as mean ± standard error of the mean (SEM). Statistical significance was evaluated using a one-way analysis of variance (ANOVA) test followed by Tukey's post-hoc multiple comparison test, where differences were considered statistically significant at P ≤ 0.05.

4.3. Docking studies

The crystal structures of HDAC-2 (PDB code: 4LXZ) was downloaded from the Protein Data Bank. Structures of compound **7a** was drawn and optimized using Avogadro molecular editors. The proteins were prepared using Autodock tools where the co-crystallized ligands and water molecules were removed then kollman charges and polar hydrogens were added. Autodock vina was used for molecular docking and the best docking poses were visualized using Discovery Studio Visualizer.

center_x = 24.167

center_y = -15.361

center_z = 1.611

size_x = 15

size_y = 15

size_z = 15

exhaustiveness = 8

Number of poses: 9

Protocol Validation (Re-docking): To formally validate our computational workflow, we performed a self-docking (re-docking) validation strategy using the native co-crystallized ligand (vorinostat, widely known as SAHA) within the human HDAC-2 crystal structure (PDB ID: 4LXZ). The top-ranked predicted docking pose successfully reproduced the experimental binding mode, yielding a Root Mean Square Deviation (RMSD) value of 1.49 Å. Because this value is well below the standard 2.0 Å success threshold, it demonstrates the high spatial accuracy and reliability of our docking protocol.

4.4. Statistical analysis

Computerized Prism 5 program was used to statistically analyzed data using one-way ANOVA test followed by Tukey's as post ANOVA for multiple comparison at P ≤.05. Data were presented as mean ± SEM.

Raw Data of HDAC-1

Compound 7a

IC_{50} = 113.246 nM

log10(Concentration nM)	% Remaining Activity
3.000	10.74
2.699	21.2
2.398	30.48
2.097	38.26
1.796	46.98
1.495	59.24
1.194	70.12
0.893	78.15

Raw Data

Concentration (nM)	RLU1	RLU2	RLU Average
1000	20406.304	20415.918	20411.111
500	19180.792	19192.928	19186.86
250	17223.061	17227.154	17225.108
125	16051.475	16060.174	16055.824
62.5	16087.629	16091.601	16089.615
31.25	12885.329	12889.634	12887.482
15.625	10344.167	10353.478	10348.823
7.813	8498.225	8501.464	8499.844

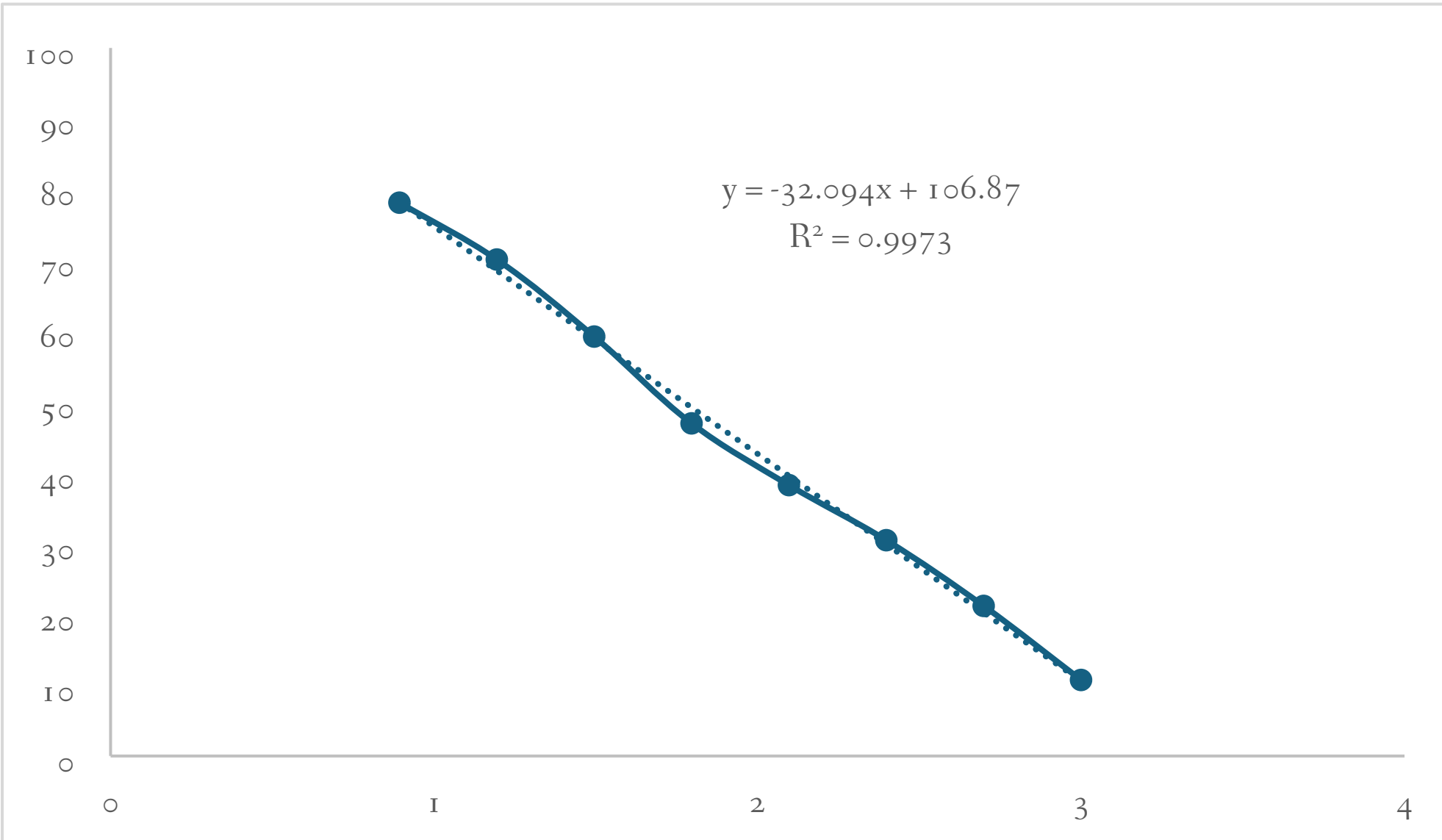

Compound 7c

IC₅₀ = 253.532 nM

log10(Concentration nM)	% Remaining Activity
3.000	15.64
2.699	25.25
2.398	30.86
2.097	41.55
1.796	47.5
1.495	55.9
1.194	65.81
0.893	73.21

Raw Data

Concentration (nM)	RLU1	RLU2	RLU Average
1000	25280.754	25292.818	25286.786
500	23768.61	23777.31	23772.96

Concentration (nM)	RLU1	RLU2	RLU Average
250	21633.557	21639.194	21636.375
125	19917.353	19925.171	19921.262
62.5	18668.752	18678.816	18673.784
31.25	17726.404	17732.166	17729.285
15.625	14983.993	14989.945	14986.969
7.813	13101.581	13106.334	13103.958

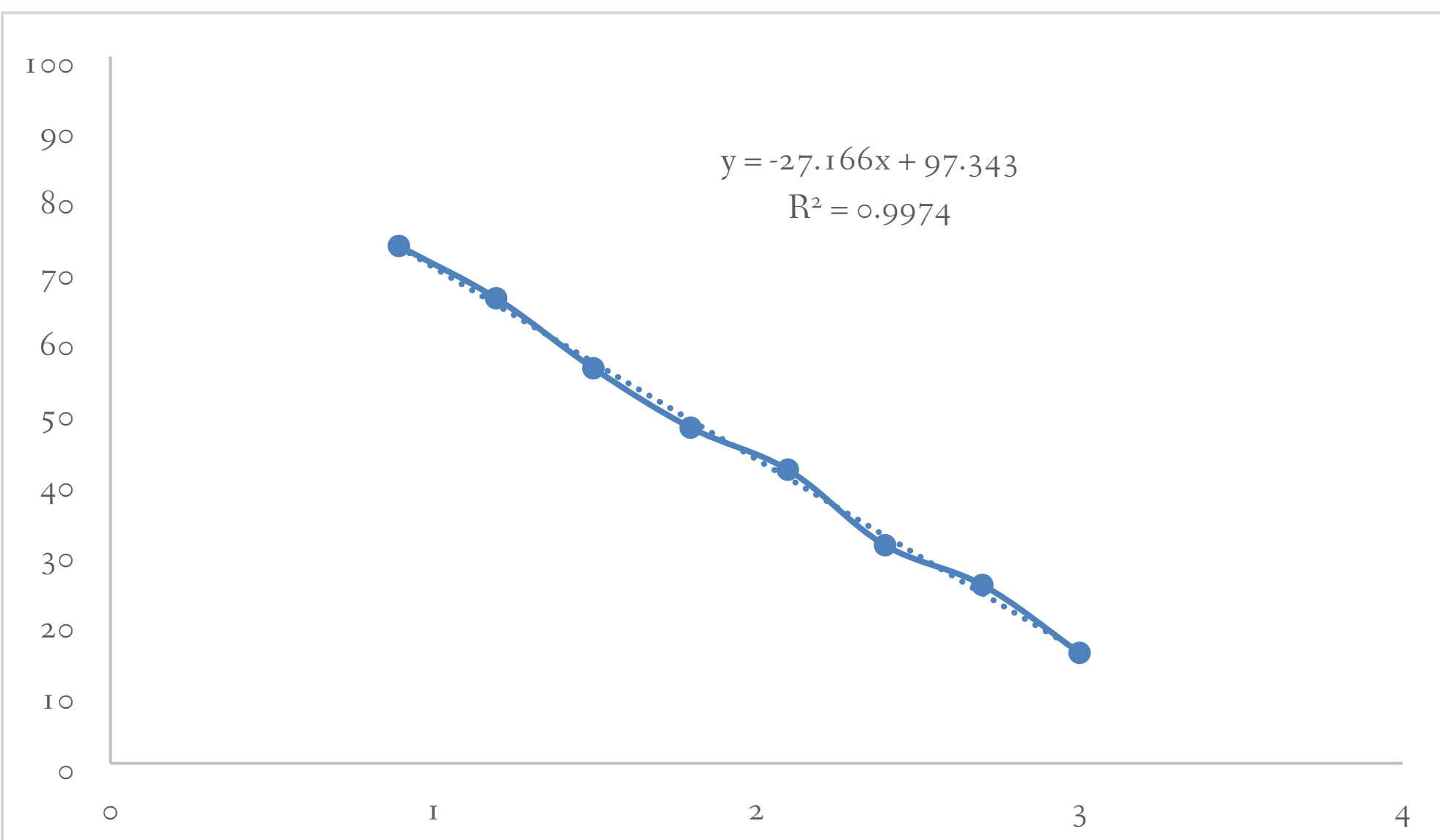

Compound 7d

IC₅₀ = 198.083 nM

log10(Concentration nM)	% Remaining Activity
3.000	13.88
2.699	24.66
2.398	43.56

log10(Concentration nM)	% Remaining Activity
2.097	60.92
1.796	77.62
1.495	87.2
1.194	92.91
0.893	97.83

Raw Data

Concentration (nM)	RLU1	RLU2	RLU Average
1000	22449.408	22461.294	22455.351
500	21389.873	21403.235	21396.554
250	19494.269	19506.369	19500.319
125	19484.682	19496.758	19490.72
62.5	17139.053	17148.549	17143.801
31.25	13208.111	13216.615	13212.363
15.625	12800.954	12809.675	12805.315
7.813	12071.476	12083.181	12077.329

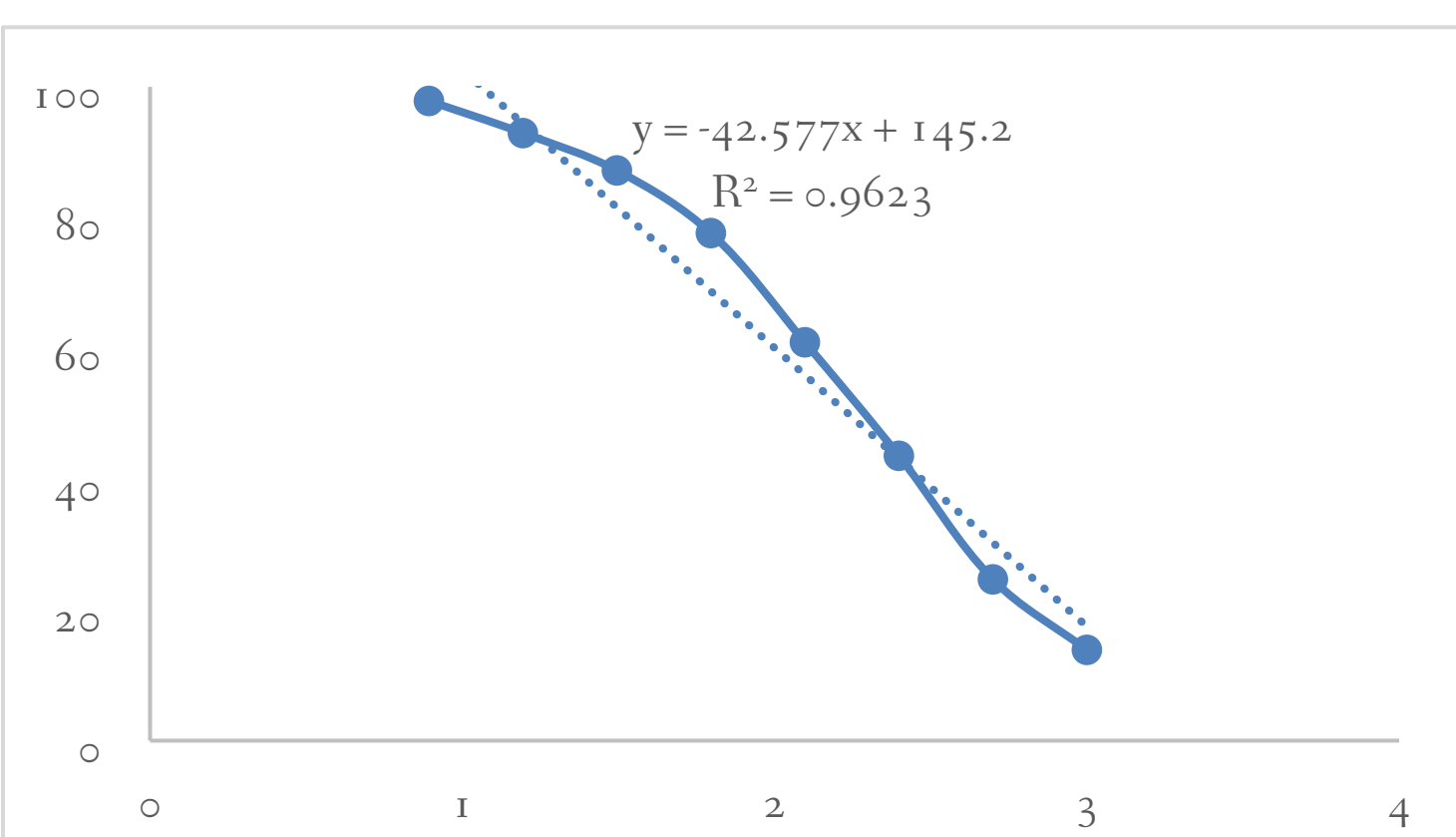

SAHA (HDAC-1)

IC₅₀ = 22.051 nM

Log Concentration	% Remaining Activity
2.6990	28.467
2.3979	31.582
2.0969	36.914
1.7959	44.736
1.4949	52.817
1.1938	57.924
0.8928	59.846
0.5917	60.714
0.2907	65.257

Raw Data

Concentration (nM)	RUL1	RUL2	RUL Average
500	2486.715	2494.382	2490.549
250	3018.264	3011.537	3014.901
125	3725.846	3733.492	3729.669
62.500	4684.137	4676.845	4680.491
31.250	5836.528	5844.116	5840.322
15.625	6824.715	6817.463	6821.089
7.8125	7569.283	7576.924	7573.104
3.9060	8096.417	8089.185	8092.801
1.9530	8514.736	8522.364	8518.550

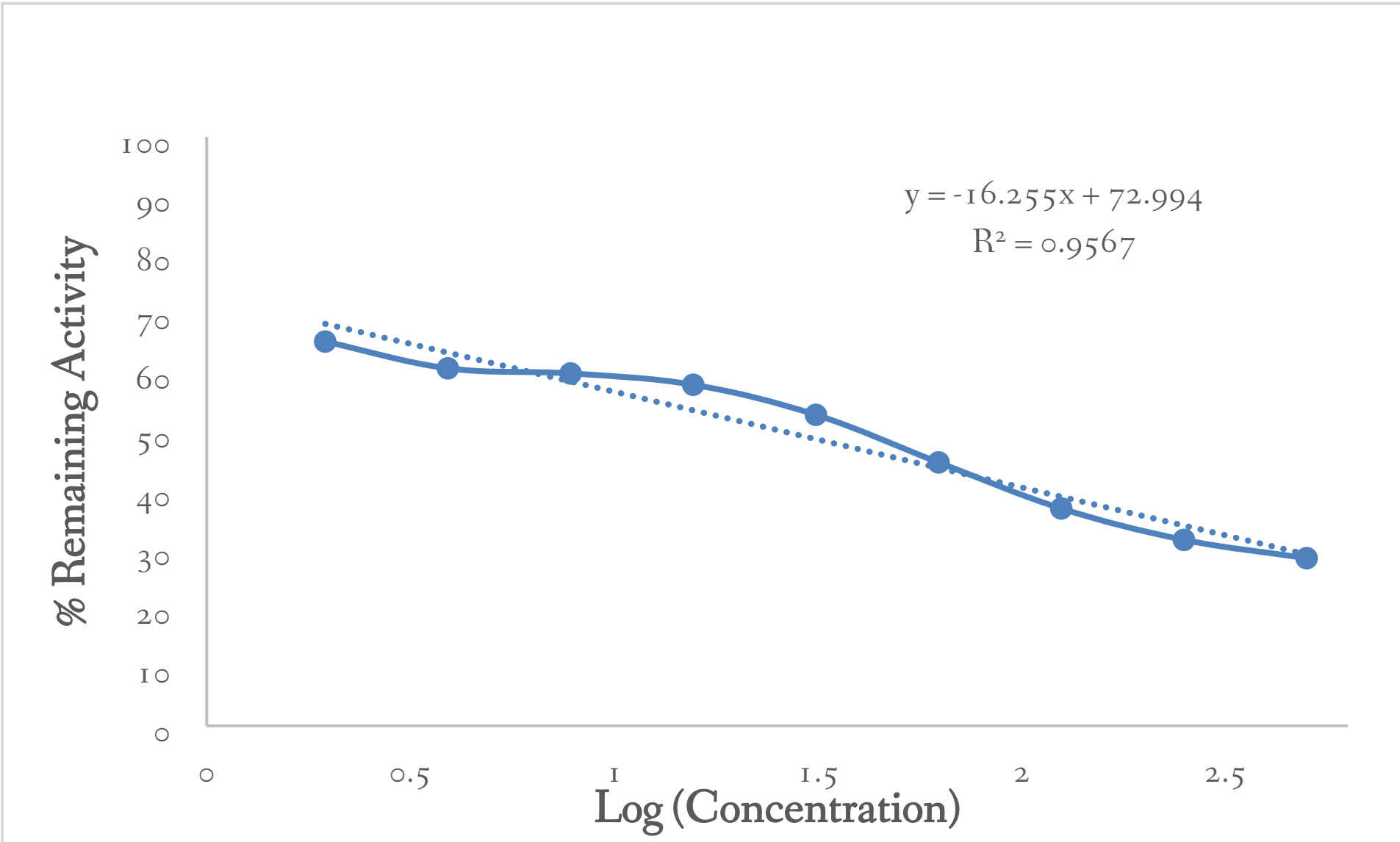

Raw Data for HDAC-2

Compound 7a

IC₅₀ = 37.159 nM

Log Concentration	% Remaining Activity
2.6990	21.285
2.3979	25.864
2.0969	33.517
1.7959	44.283
1.4949	56.908
1.1938	65.192
0.8928	69.784
0.5917	71.926
0.2907	73.075

Raw Data

Concentration (nM)	RUL1	RUL2	RUL Average
500	1836.517	1844.926	1840.722
250	2415.783	2408.264	2412.024
125	3188.642	3196.118	3192.380
62.500	4277.351	4269.887	4273.619

Concentration (nM)	RUL1	RUL2	RUL Average
31.250	5584.926	5592.473	5588.700
15.625	6765.418	6758.236	6761.827
7.8125	7818.537	7826.945	7822.741
3.9060	8524.681	8517.294	8520.988
1.9530	9066.247	9074.832	9070.540

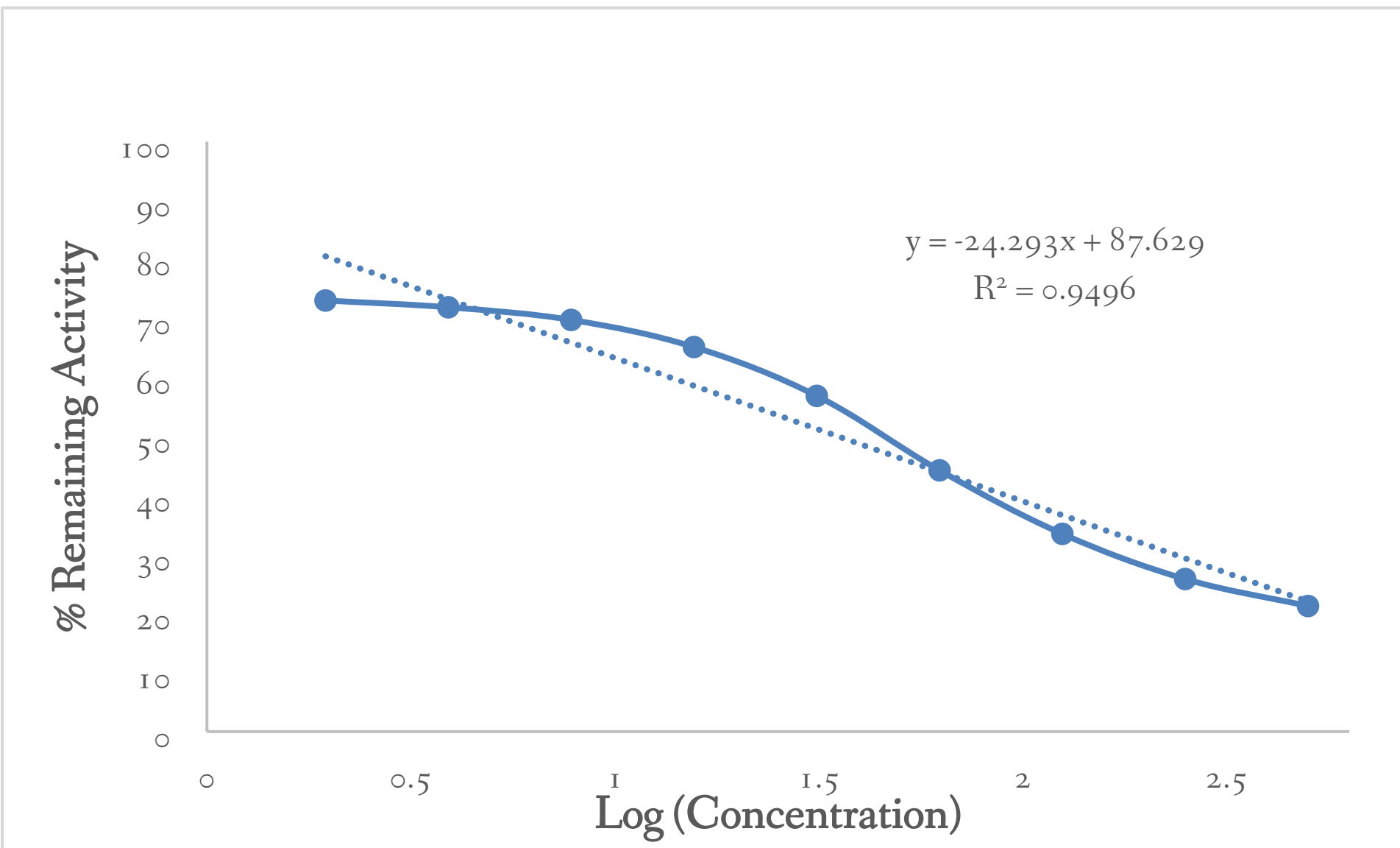

Compound 7c

IC₅₀ = 45.471 nM

log10(Concentration nM)	% Remaining Activity
3.000	4.38
2.699	7.12
2.398	13.58
2.097	22.83
1.796	42.75
1.495	60.85
1.194	77.98
0.893	87.89

Raw Data

Concentration (nM)	RLU1	RLU2	RLU Average
1000	24054.751	24057.656	24056.203
500	22580.85	22585.403	22583.127
250	21615.581	21619.454	21617.517
125	18864.656	18869.863	18867.26
62.5	16756.209	16769.798	16763.004
31.25	17091.597	17099.492	17095.544
15.625	18612.07	18621.715	18616.893
7.813	12642.786	12652.763	12647.774

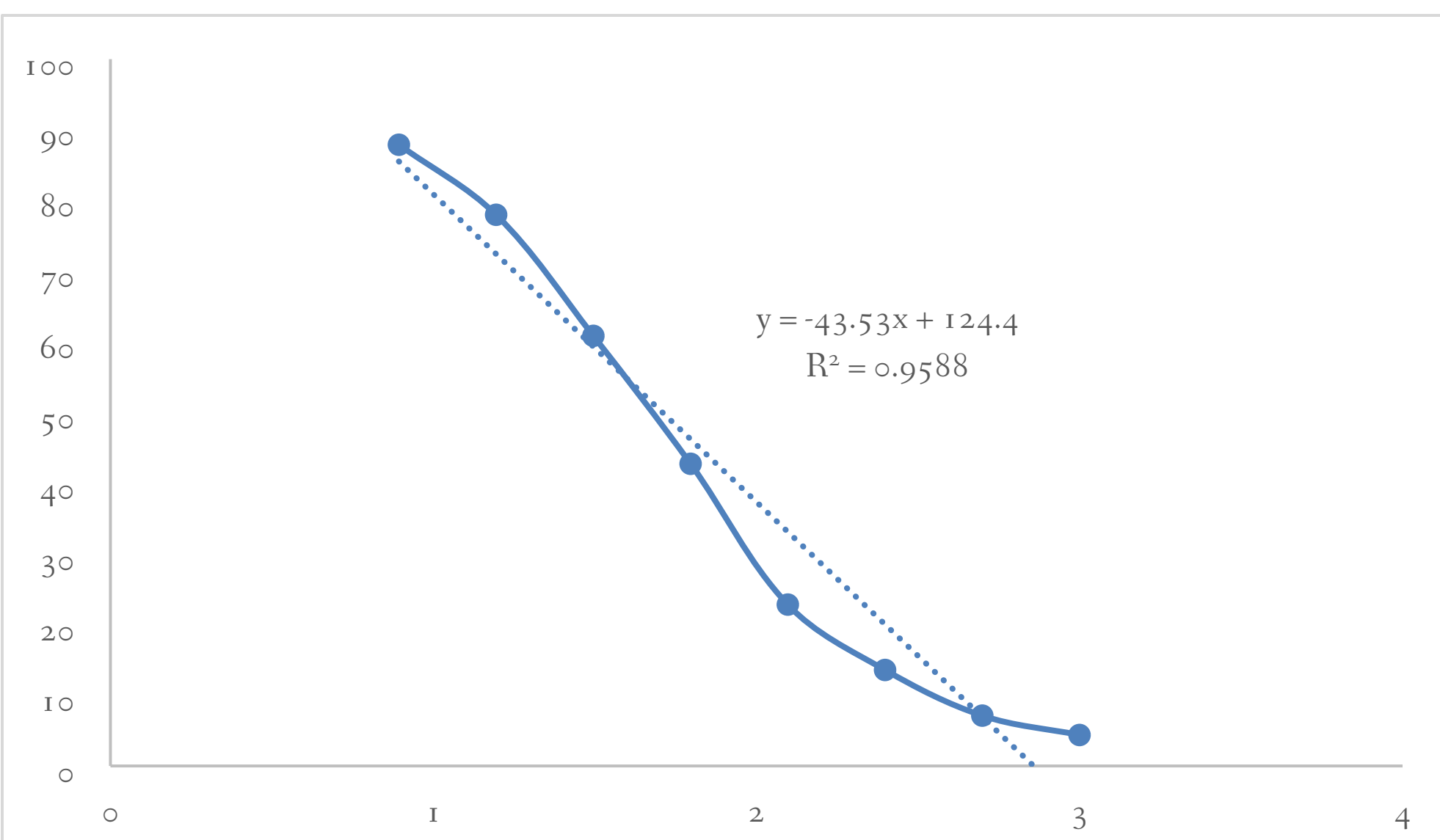

Compound 7d

IC₅₀ = 39.288 nM

Log Concentration	% Remaining Activity
2.6990	17.427
2.3979	22.186
2.0969	29.814
1.7959	40.926
1.4949	53.417
1.1938	64.538
0.8928	70.821
0.5917	74.032
0.2907	75.869

Raw Data

Concentration (nM)	RUL1	RUL2	RUL Average
500	1568.342	1575.914	1572.128
250	2194.675	2187.241	2190.958
125	3018.526	3026.148	3022.337
62.500	4167.832	4160.519	4164.176
31.250	5516.284	5524.913	5520.599
15.625	6735.491	6728.246	6731.869
7.8125	7748.935	7756.482	7752.709
3.9060	8504.627	8497.318	8500.973
1.9530	9056.274	9064.815	9060.545

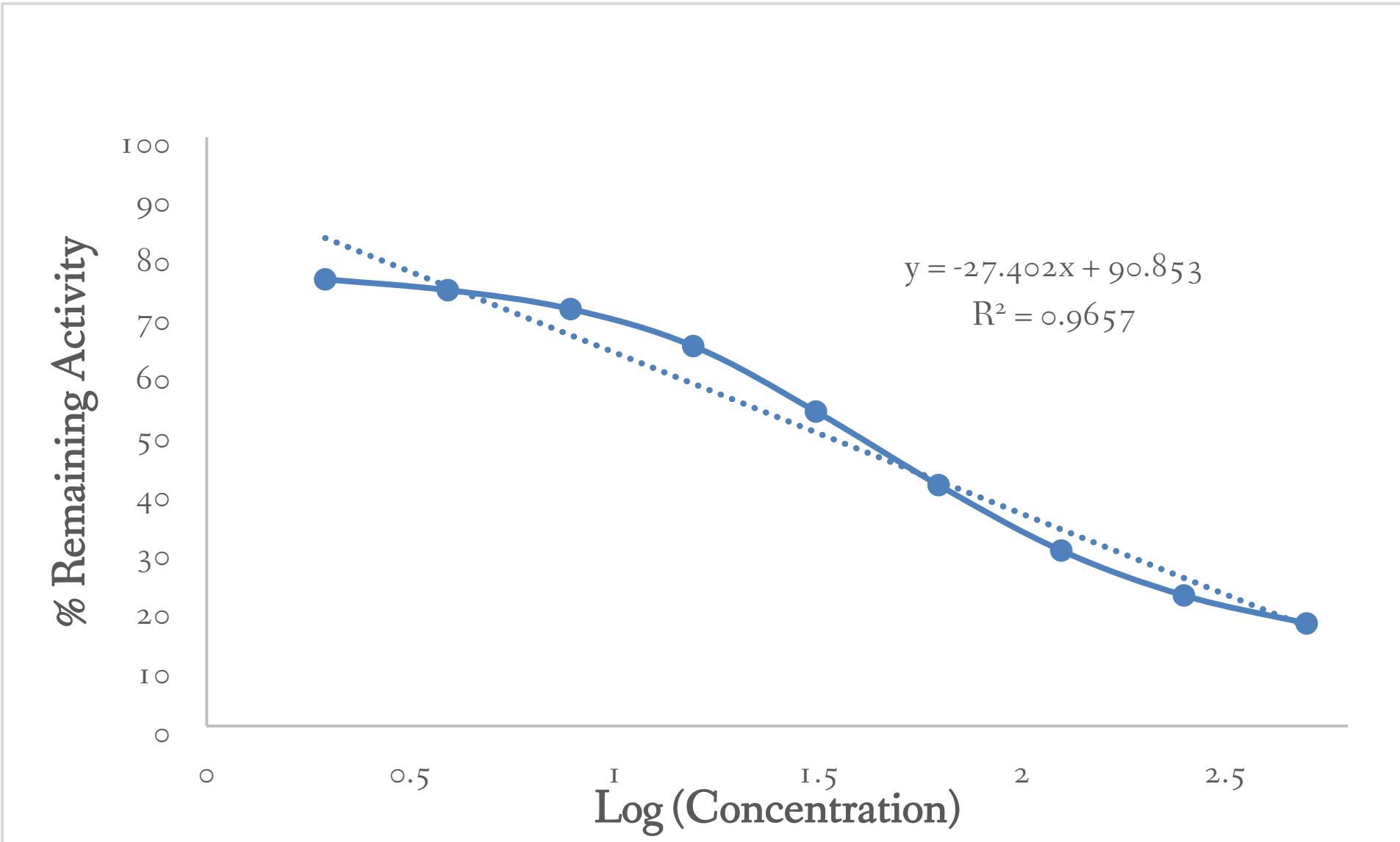

Compound 7e

IC₅₀ = 64.007 nM

Log Concentration	% Remaining Activity
2.6990	7.563
2.3979	12.886
2.0969	21.417
1.7959	35.842
1.4949	51.093

Log Concentration	% Remaining Activity
1.1938	66.784
0.8928	76.235
0.5917	81.408
0.2907	84.336

Raw Data

Concentration (nM)	RUL1	RUL2	RUL Average
500	864.518	871.946	868.232
250	1295.733	1288.451	1292.092
125	2017.846	2025.391	2021.619
62.500	3076.924	3069.518	3073.221
31.250	4475.386	4483.927	4479.657
15.625	5896.714	5889.365	5893.040
7.8125	7117.928	7125.436	7121.682
3.9060	8014.583	8007.192	8010.888
1.9530	8486.317	8494.864	8490.591

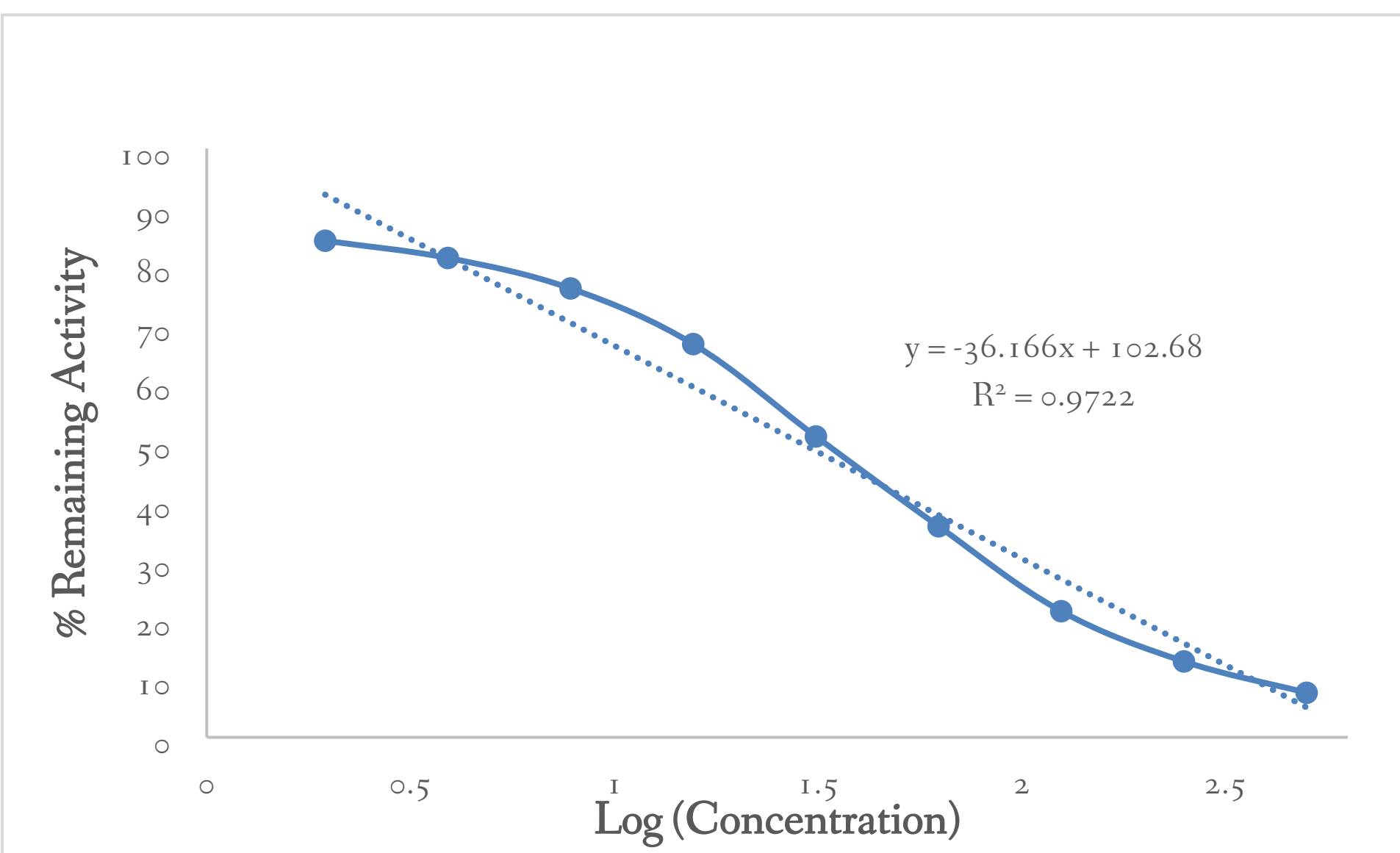

Compound 7f

IC₅₀ = 53.259 nM

Log Concentration (nM)	% Remaining Activity
1.699	53.12
1.398	57.84
1.097	62.61
0.796	76.73
0.495	80.68
0.194	94.31
-0.107	97.89

Raw Data

Concentration (nM)	RLU1	RLU2	RLU Average
50.000	26391.482	26441.773	26416.628
25.000	27984.631	27921.294	27952.963
12.500	29641.904	29712.558	29677.231
6.250	31294.337	31352.119	31323.228
3.125	32841.775	32791.462	32816.619
1.563	34112.663	34171.228	34141.946
0.781	35491.904	35421.337	35456.621

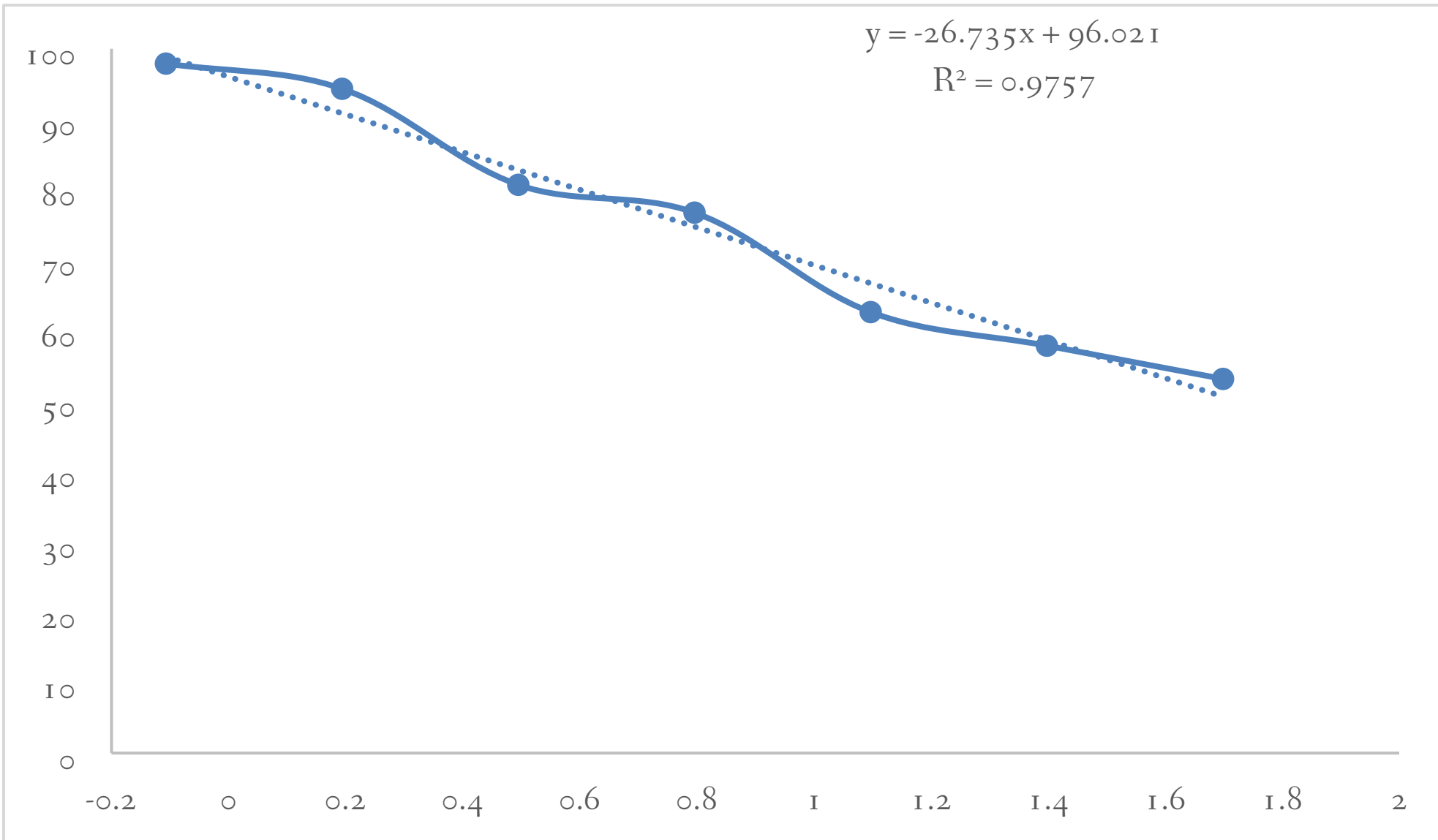

Compound 7l

IC₅₀ = 69.551 nM

Log Concentration (nM)	Remaining Activity (%)
2.3010	10.174
2	19.433
1.6990	32.474
1.3979	46.824
1.0969	60.198
0.7959	71.045
0.4949	79.495
0.1939	85.389
-0.1079	88.356
-0.4078	91.557

Raw Data

Concentration (nM)	RUL1	RUL2	RUL Average
200	4597.821	4618.927	4608.374
100	8786.145	8810.679	8798.412
50	14692.358	14716.724	14704.541
25	21188.413	21212.769	21200.591
12.500	27242.784	27268.336	27255.560

Concentration (nM)	RUL1	RUL2	RUL Average
6.250	32156.225	32180.839	32168.532
3.125	35983.761	36006.459	35995.110
1.563	38648.872	38674.398	38661.635
0.781	39994.531	40018.987	40006.759
0.391	40542.968	40566.927	40554.948

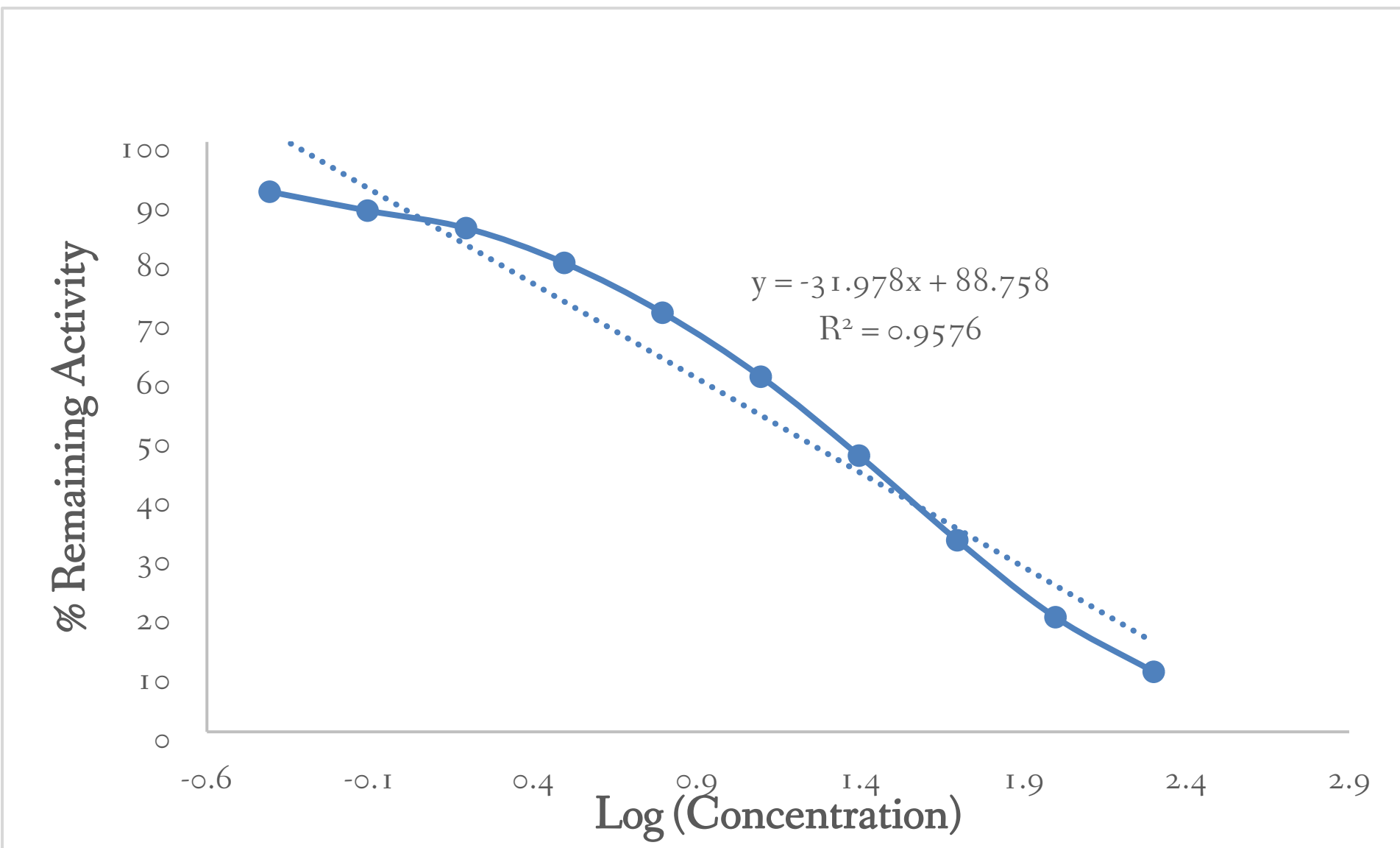

SAHA (HDAC-2)

IC_{50} = 30.706 nM

Log Concentration (nM)	Remaining Activity (%)
2.301	18.795
2	20.650
1.699	24.880
1.398	44.920
1.097	53.380
0.796	58.610
0.495	61.420
0.194	62.980
-0.107	63.740
-0.408	64.095

Raw Data

Concentration (nM)	RLU1	RLU2	RLU Average
200	14689.842	14714.618	14702.230
100	16142.973	16167.887	16155.430
50	19451.286	19481.414	19466.350
25	35123.817	35160.883	35142.350
12.500	41743.994	41781.166	41762.580
6.250	45831.729	45877.171	45854.450
3.125	48029.536	48074.944	48052.240
1.563	49247.531	49297.869	49272.700
0.781	49839.224	49895.336	49867.280
0.391	50116.587	50173.433	50145.010

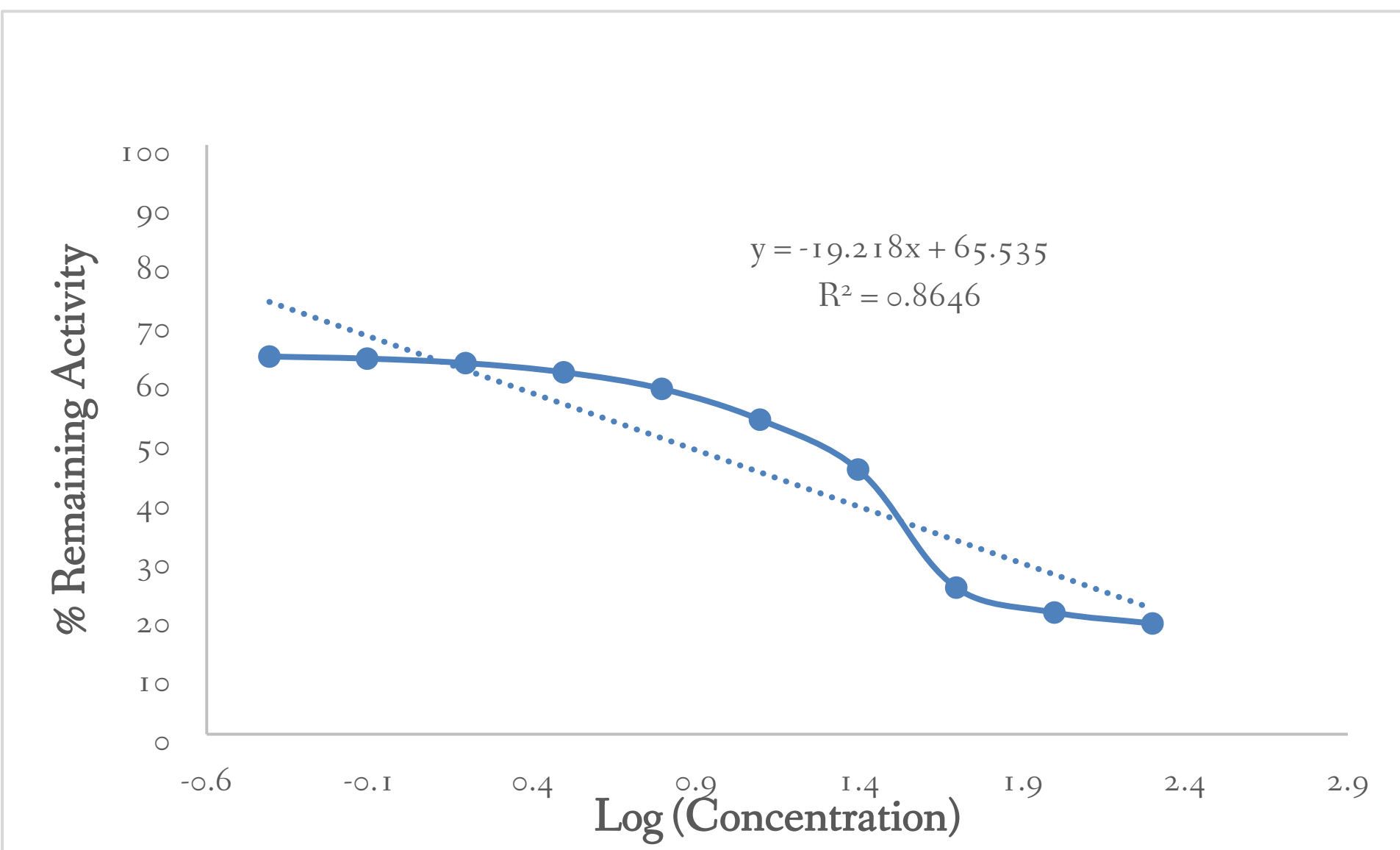

Raw data for Western Blotting Assay

Lab Report

*-

S	Compound			western blotting	B-actin
	code	Cells	conc	OD	
				HDAC2	
				60 kda	
I	control	SEM	---	7.77	√
II	7a	---	---	3.18	□

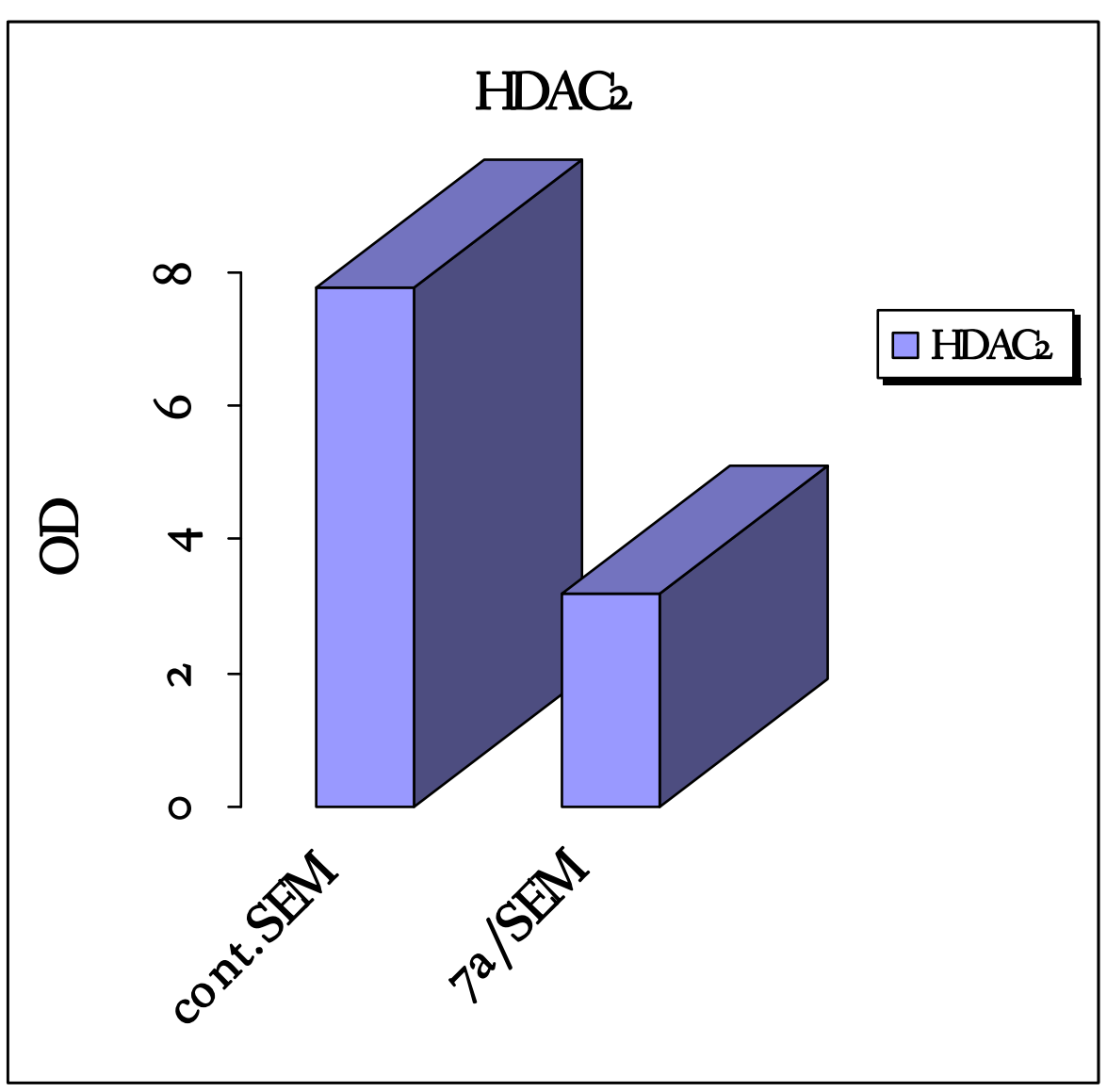

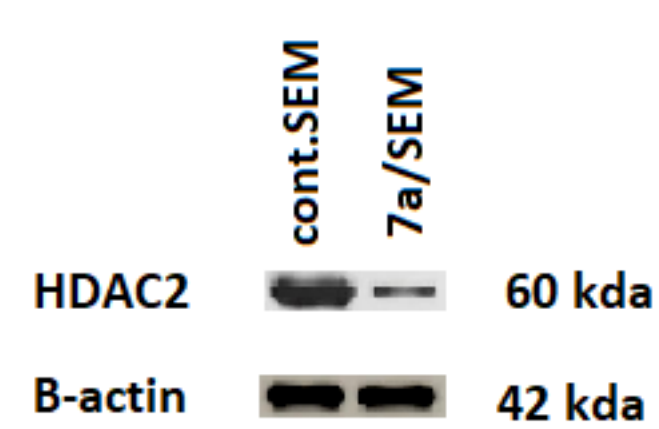

Detailed Results:

HDAC2	60 kda			
sample band	area	%	Rel.Density	adj. Density
Standared	1337	13.37	1	1
cont.SEM	6493	64.93	4.856394914	7.770231862
7a/SEM	2664	26.64	1.992520568	3.188032909

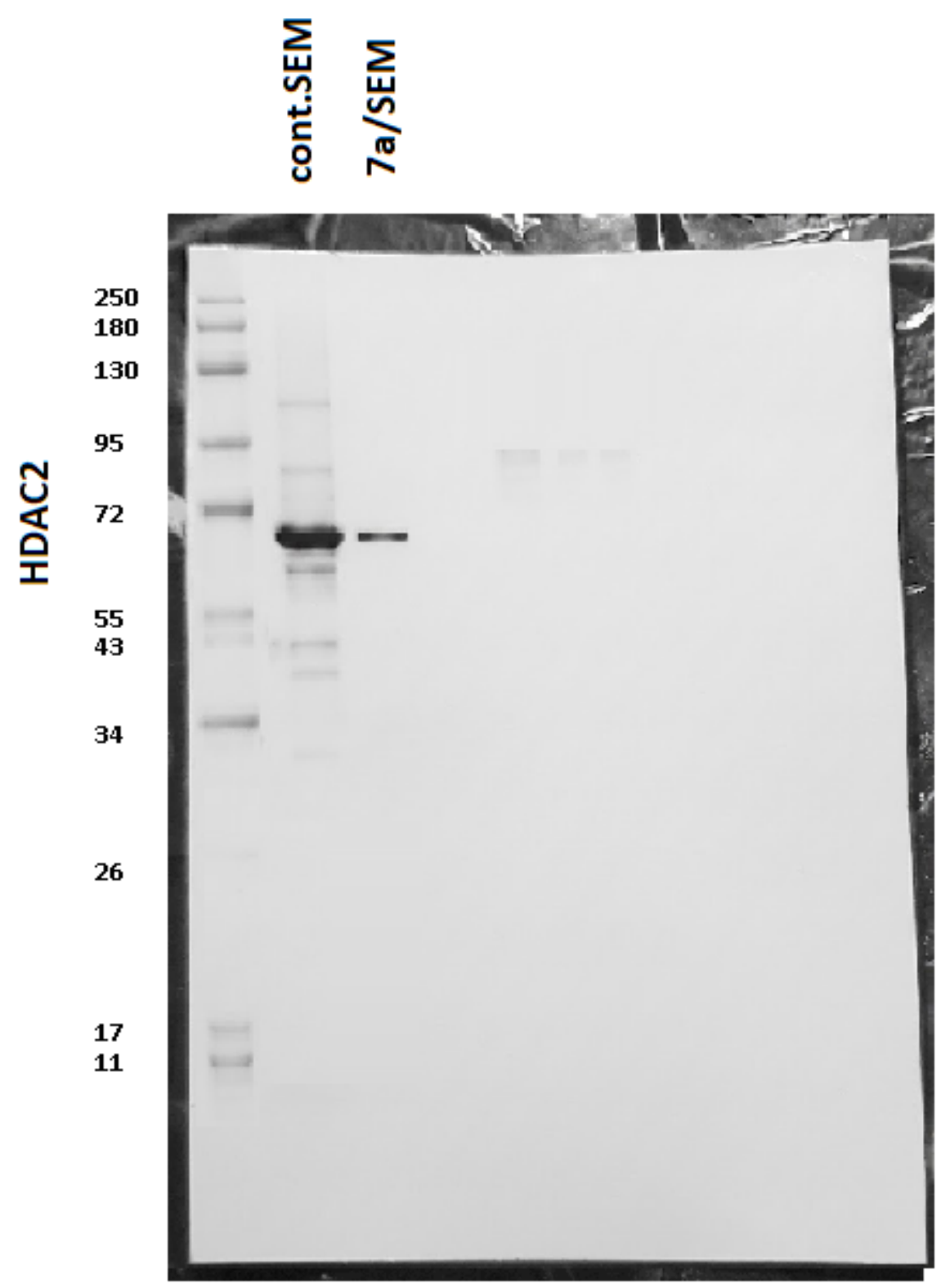

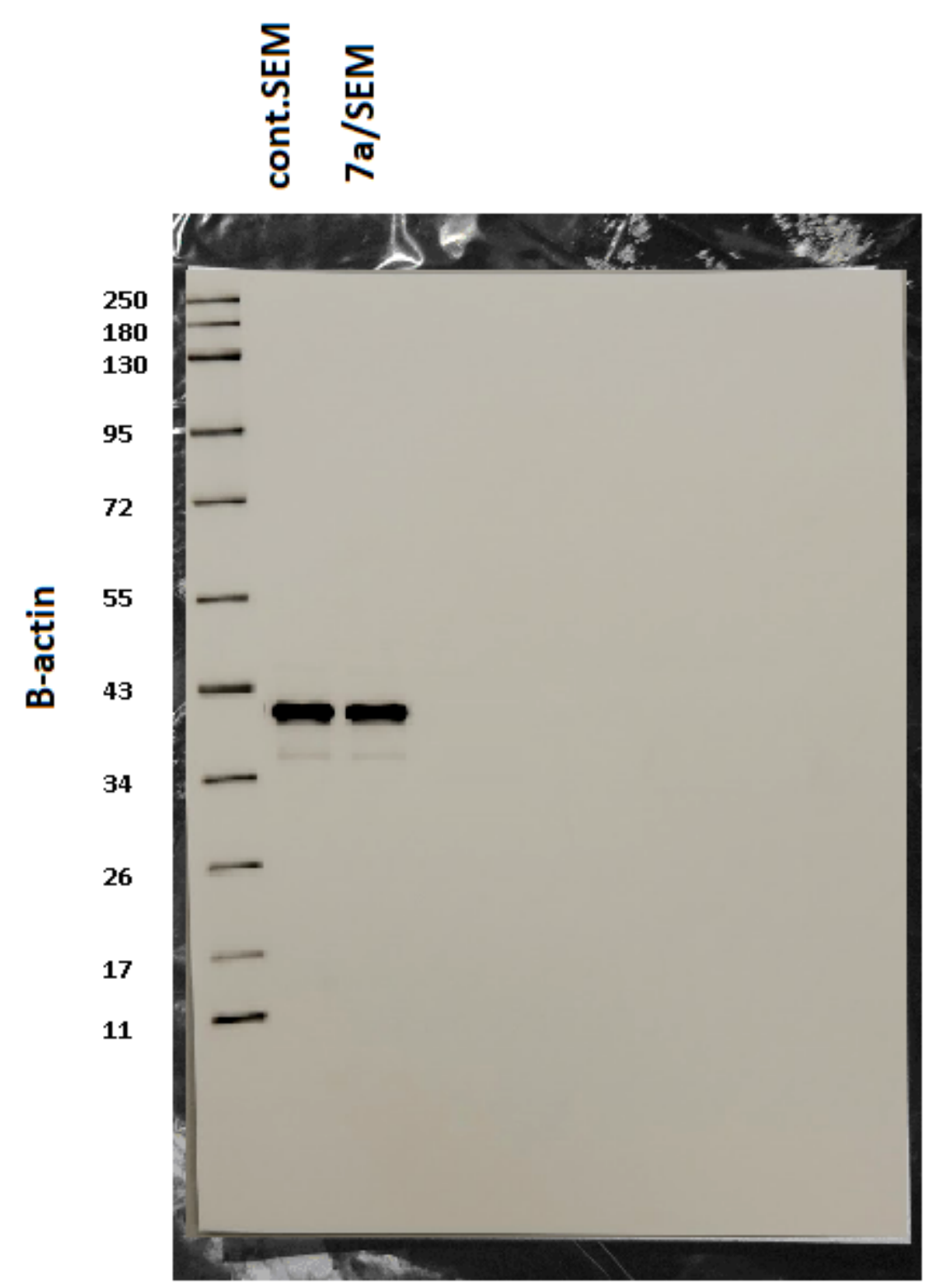

Detailed Western Blotting Protocol for HDAC2 and Beta Actin

1. Sample Preparation (SEM Cell Lysates)

- Following treatment of SEM cells with the compound (1 mg/ml) , after 24h incubation ,the cells being harvested and lysated using a standard RIPA buffer supplemented with protease and phosphatase inhibitors cocktail (Abcam-Ab201119).

2. Antibodies (The Key Reagents)

Target Protein	Recommended Antibody	Host / Type	Validated on SEM	Recommended Dilution	Expected Band Size
HDAC2	Invitrogen HDAC2 Monoclonal Antibody (9H4), Catalog # MA5-38405	Rabbit / Monoclonal	Yes	1:1,000	~60 kDa
Beta Actin (β-actin)	Invitrogen beta Actin Recombinant Mouse Monoclonal Antibody (A2-F6-R), Catalog # MA5-50640	Mouse / Monoclonal	Yes	1:10,000	42 kDa

3. Electrophoresis and Transfer

- **Gel Electrophoresis**: 25 µg protein samples Loaded per lane onto 4% Bis-Tris polyacrylamide gel..
- **Protein Transfer**: proteins Transferd from the gel to a nitrocellulose membrane.
  - **semi-dry transfer** system Invitrogen Power Blotter is used for 10 min.

4. Immunodetection

- **Blocking**: Incubate the membrane with a blocking buffer (5% non-fat dry milk in TBST) for 1 hour at room temperature.
- **Primary Antibody Incubation**: the membrane Incubate with diluted primary antibody (anti-HDAC2 at 1:1,000) overnight at 4°C with gentle shaking .
- **Secondary Antibody Incubation**: After washing, the membrane incubated with an **HRP-conjugated secondary antibody**. goat anti-rabbit secondary antibody (Invitrogen). dilution is 1:10,000 is used (Catalog # 31210) .
- Detection Kit: protein bands are Visualized using chemiSOLO chemiluminescent detection system.
- detection: **stabilized Streptavidin-HRP conjugate, chemiluminescent substrate (luminol/enhancer + peroxide).**
- **Imaging**: Capture the signal using a chemiluminescence imager or X-ray film.

Raw data for Cell Cycle Analysis

Lab Report

ser	Sample		DNA content				
	code	IC50 ug	%G0-G1	%S	%G2/M	Comment	
1	7a/SEM	---	37.86	15.92	46.22	Cell cycle arrest@G2/M	
2	Cont. SEM	---	59.66	33.13	7.21	---	

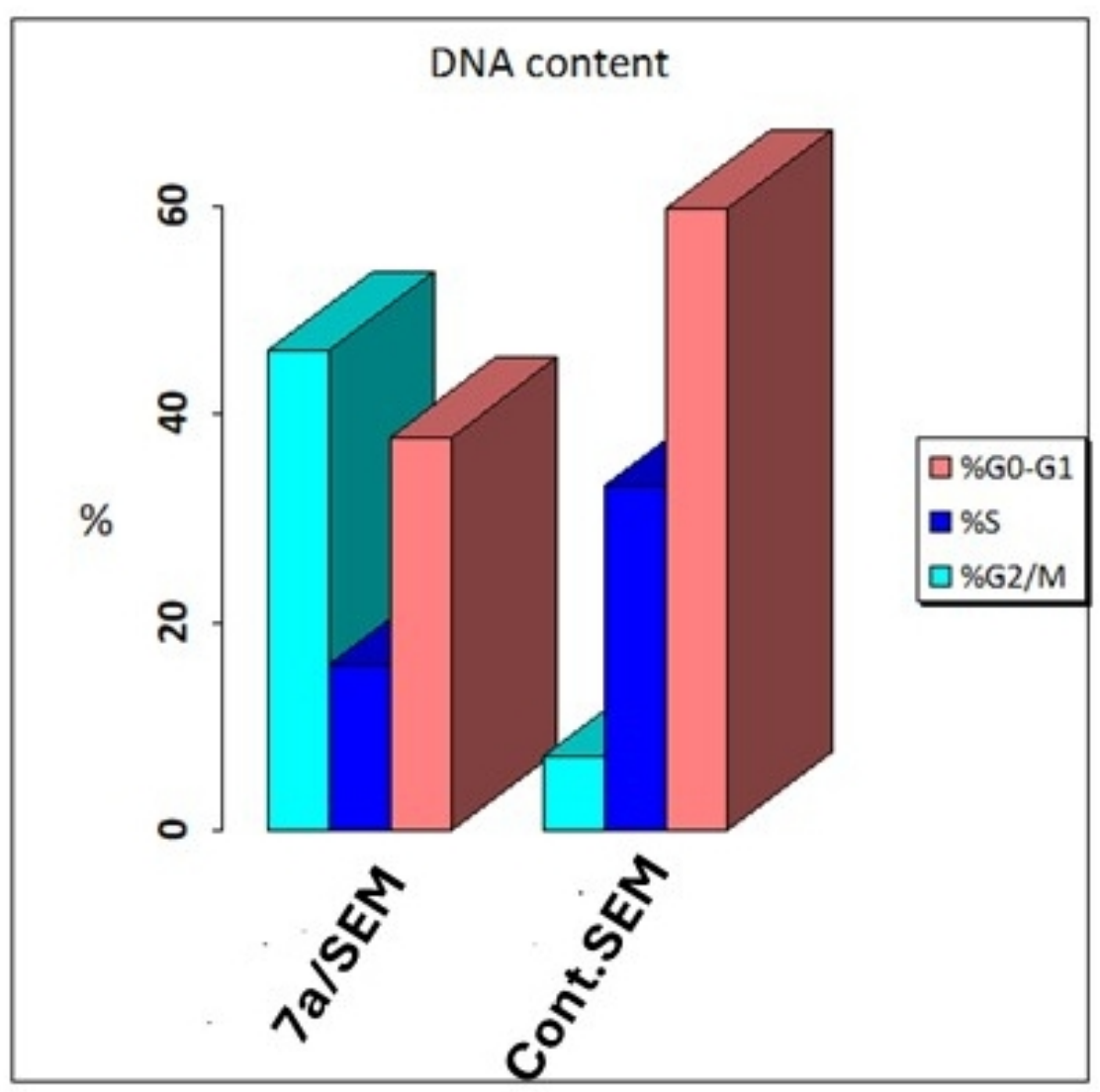

Apoptosis/necrosis :

s	code	conc	Apoptosis			Necrosis
			Total	Early	Late	
1	7a/ SEM	---	31.12	8.28	22.84	4.29
2	Cont. SEM	---	0.81	0.59	0.22	2.68

Detailed results :

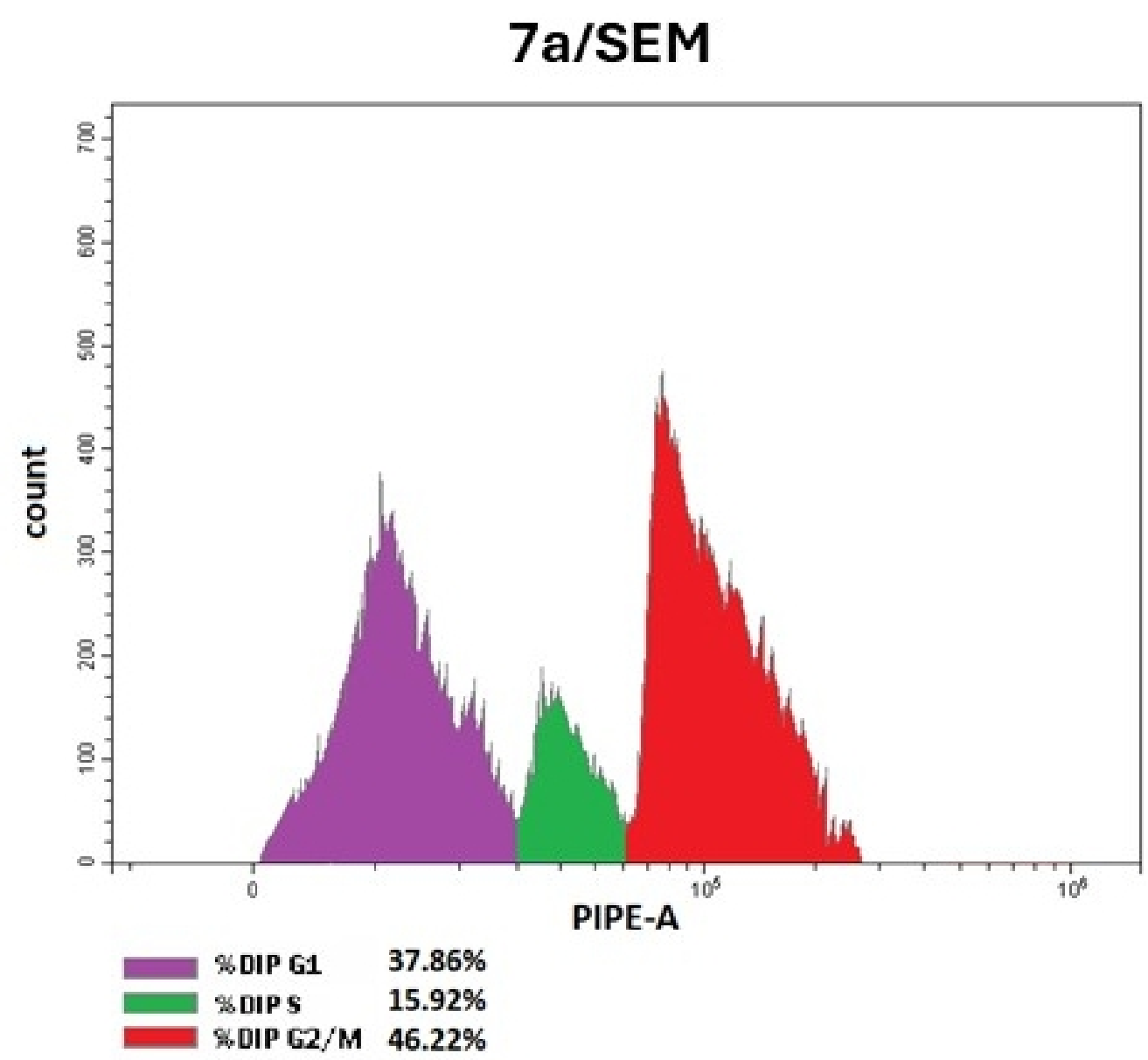

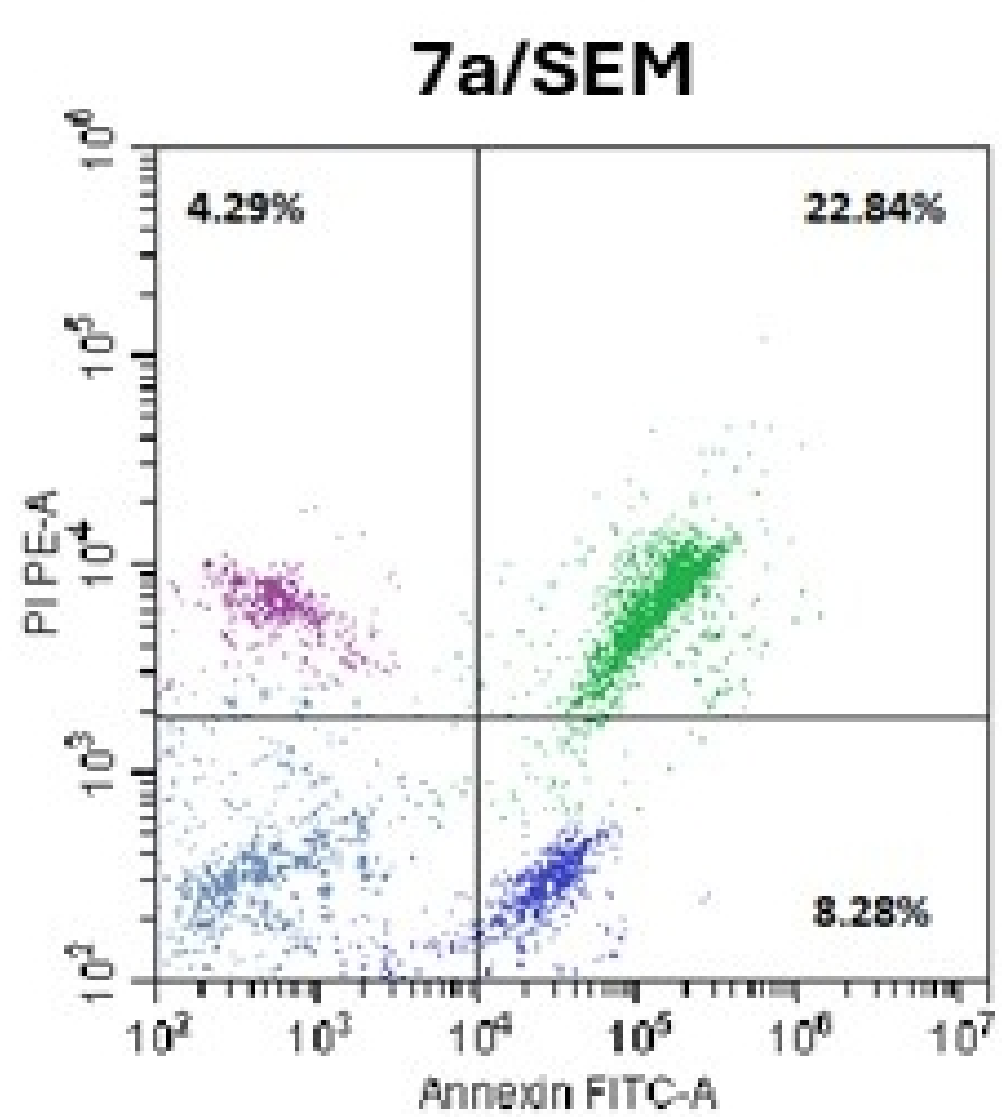

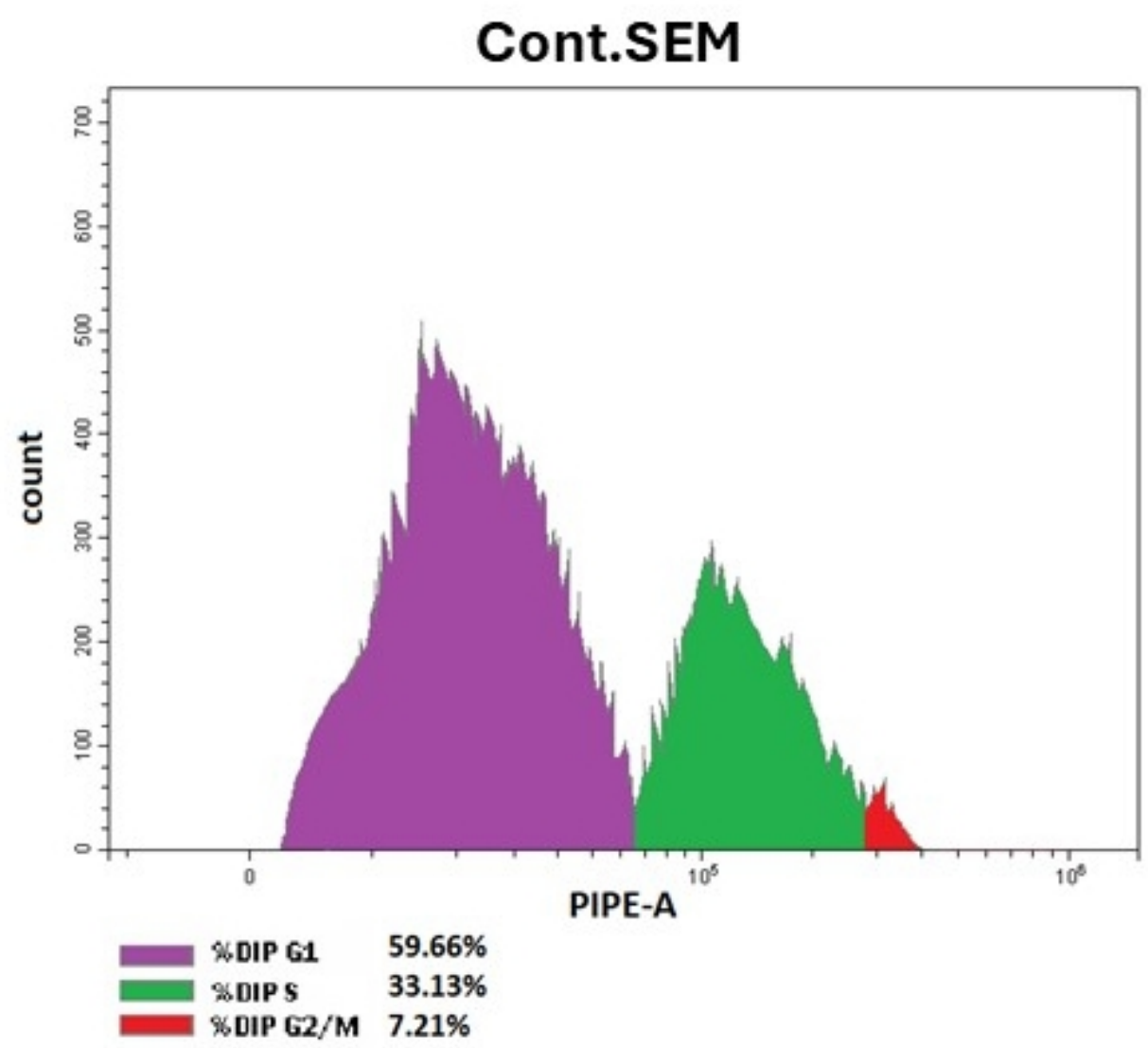
Cont.SEM
count
PIPE-A
%DIP G1 59.66%
%DIP S 33.13%
%DIP G2/M 7.21%

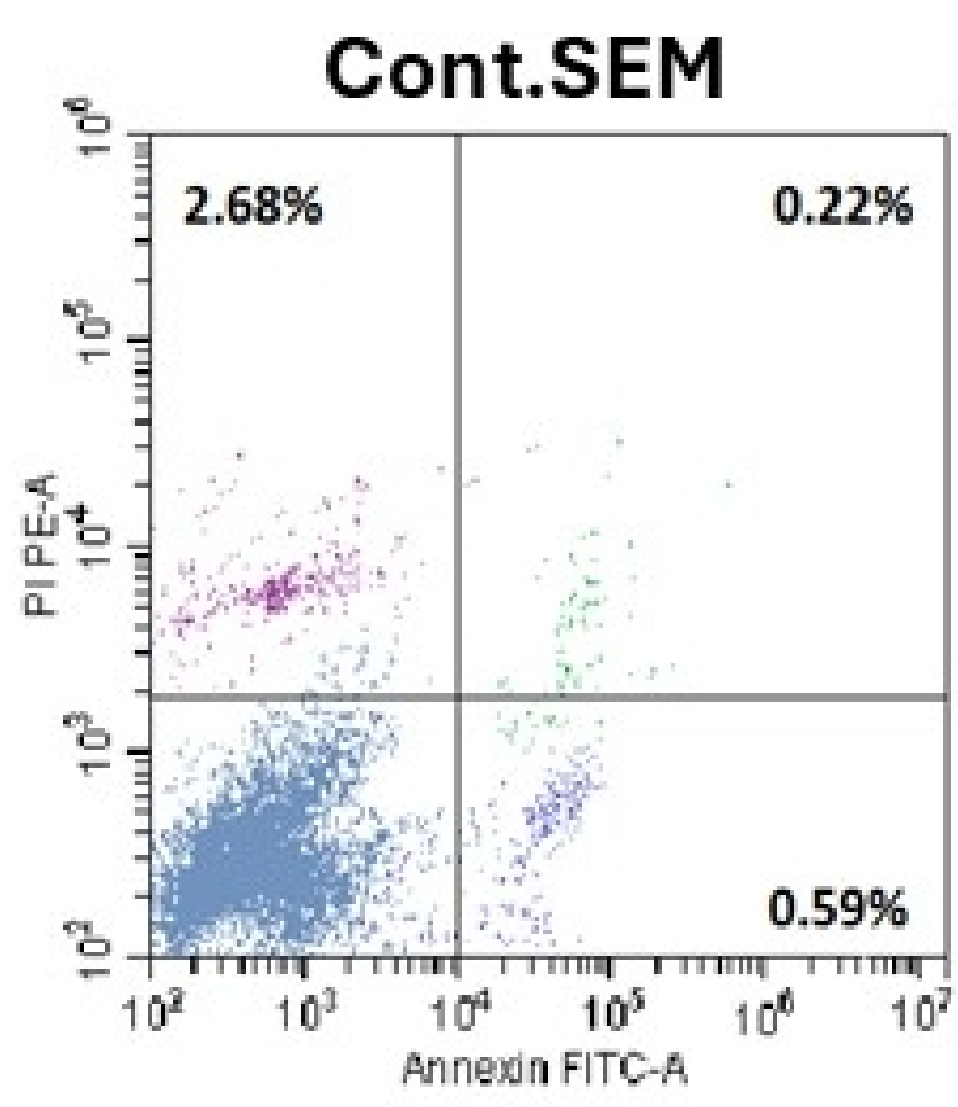
Cont.SEM
2.68%
0.22%
0.59%
PI PE-A
Annexin FITC-A

Raw Data of MTT assay

Average of Relative viability of cells (%)

Conc. (μM)	MDA-MB-231	SEM	A-549
Vorinostat (SAHA)			
100	6.3	7.1	6.2
50	11.2	13.9	10.9
25	14.1	18.7	14.3
12.5	28.3	31.4	26.9
6.25	45.8	47.9	41.5
3.125	57.6	60.5	58.4
1.56	71.2	73.8	69.1
Compound 7a			
100	7.4	6.3	8.3
50	12.1	11.2	12.9
25	18.8	14.1	21.5
12.5	27.9	28.3	30.6
6.25	52.7	45.8	54.8
3.125	63.5	57.6	62.2

1.56	72.4	71.2	75.4
Compound 7c			
100	9.2	6.2	8.1
50	16.6	10.9	15.2
25	25.7	14.3	23.0
12.5	37.2	26.9	34.8
6.25	61.3	41.5	51.6
3.125	72.9	58.4	68.9
1.56	95.4	69.1	87.5
Compound 7d			
100	6.5	4.7	7.8
50	13.1	10.2	14.5
25	20.8	16.5	21.2
12.5	33.6	23.4	34.0
6.25	48.7	41.6	50.3
3.125	63.3	58.1	64.9
1.56	86.2	67.3	87.4

Swiss ADME Predictions

Lipophilicity

The lipophilicity profile of compound **7a** further corroborates its potential as a therapeutic candidate, demonstrating an optimal equilibrium between hydrophilicity and lipophilicity. Lipophilicity was assessed by various computational approaches, yielding a consensus Log $P_{o/w}$ of 1.61. The principal atomistic and fragment-based prediction models exhibited substantial concordance. XLOGP3, WLOGP, and MLOGP yielded values of 2.02, 2.38, and 1.97, respectively, which were closely aligned. The SILICOS-IT hybrid model produced a significantly lower outlier value of 0.06; yet the overall cluster of core predictors indicates that compound **7a** resides within the ideal lipophilic range ($1 < \text{Log P} < 3$), typically aimed for in the initial phase of drug optimization.

The compound possesses moderate lipophilicity that is very favorable for its pharmacokinetic and ADME behavior. An intermediate Log P in this range is ideal for passive transcellular absorption as it

provides a balance of hydrophobicity that allows the molecule to partition into the lipid bilayer of intestinal epithelial cells but hydrophilicity that allows the molecule to desolvate and dissolve in the aqueous extracellular environment. Importantly, this moderate lipophilicity alleviates the structural concerns expressed earlier with the compound's fully planar framework (Fraction Csp^3 = 0.00) and suggests that the compound will not suffer from the extreme insolubility generally associated with intensely lipophilic, flat aromatic structures.

A consensus Log P below 3 is advantageous from both metabolic and safety standpoints to mitigate off-target risks. Excessively lipophilic compounds frequently undergo metabolic clearance through fast cytochrome P450 oxidation and exhibit a higher propensity for non-specific binding to plasma proteins and off-targets, including the hERG ion channel. Compound **7a** maintains a moderate lipophilicity, so mitigating the risk of hydrophobic promiscuity while preserving the structural characteristics necessary for effective membrane transport and durable target binding.

Water Solubility

The water solubility of lead compound **7a** was assessed using three apparent predictive models (ESOL, Ali, and SILICOS-IT), offering a thorough analysis of its dissolution characteristics in aqueous environments. Thermodynamic solubility estimates revealed Log S values ranging from -3.75 to -4.57, categorizing **7a** as "soluble" to "moderately soluble". The topological ESOL model predicted the highest solubility with a Log S of -3.75 (7.58×10^{-2} mg/mL; 1.77×10^{-4} mol/L), while the fragment-based Ali and SILICOS-IT methods provided more conservative estimates of -4.26 (2.37×10^{-2} mg/mL; 5.54×10^{-5} mol/L) and -4.57 (1.15×10^{-2} mg/mL; 2.70×10^{-5} mol/L), respectively.

Compound **7a** is sufficiently water soluble to support early biological screening and intravenous or oral formulation testing. These empirical patterns confirm the balancing effect of the compound's moderate lipophilicity over its unfavorable geometric limitations. Compound **7a**'s totally planar framework (Fraction Csp^3 = 0.00) prevents water from dissolving due to high crystal lattice energy and strong intermolecular stacking. The consensus Log P is 1.61, indicating that the molecule is in a controlled and moderate regime, and therefore not excessively hydrophobic, which would otherwise render a flat aromatic core entirely insoluble.

The modest solubility in water guarantees that free drug concentrations in biological fluids remain sufficiently elevated to facilitate effective engagement with target receptors, minimizing the immediate risk of precipitation. From the perspective of drug development, a solubility profile between (10^{-4}) and (10^{-5}) M is deemed highly acceptable for early-stage lead compounds. The modest solubility indicates that future formulation improvement may be necessary to enhance systemic exposure, potentially through the use of cosolvents, salt forms, or cyclodextrin complexation; nonetheless, the baseline data reveals no obvious biopharmaceutical concerns. Consequently, compound **7a** serves as a strong framework for additional therapeutic investigation owing to its favorable Lipinski compliance, regulated peripheral distribution, well-balanced lipophilicity, and satisfactory water solubility.

Pharmacokinetic profile

The pharmacokinetic features of compound **7a** align with its potential as an orally accessible medicinal drug, exhibiting a minimal likelihood of active efflux or central nervous system adverse effects. Computational screening indicates elevated gastrointestinal (GI) absorption, aligning with the structural

criteria established by Lipinski's and Veber's principles, which encompass its advantageous molecular weight, restricted hydrogen bonding potential, and ideal topological polar surface area (TPSA). **7a** is notably not a substrate for P-glycoprotein (P-gp), the primary ATP-binding cassette transporter that facilitates active drug efflux in the intestinal epithelium and blood-brain barrier. Compound **7a** evades P-glycoprotein-mediated efflux from target tissue cells, enhancing its bioavailability and leading to more consistent systemic concentrations following oral administration.

Compound **7a** is classified as non-permeant to the blood-brain barrier (BBB) based on its tissue distribution and target localization. The result aligns with the structural study of its elevated TPSA (124.25 Å2), which limits passive diffusion across the tight junctions of the brain capillary endothelium. The lack of penetration into the central nervous system is advantageous for a medication aimed at peripheral targets. It effectively confines the pharmacological activity of **7a** to systemic circulation and peripheral tissues, hence decreasing the likelihood of central side effects. Compound **7a** exhibits a skin permeation coefficient (Log Kp) value of -7.47 cm/s. This comparatively low number indicates a sluggish rate of skin absorption, affirming that the molecule is inadequately designed for transdermal delivery, while being highly optimized for oral or systemic administration routes.

The inherent physicochemical characteristics offer a first safeguard against metabolic risks. A comprehensive metabolic profile for the inhibition of cytochrome P450 (CYP) is currently unavailable for the principal isoforms (CYP1A2, CYP2C19, CYP2C9, CYP2D6, and CYP3A4). The moderate lipophilicity profile (consensus Log P = 1.61) considerably diminishes the likelihood of **7a** acting as a non-specific, high-affinity inhibitor or substrate for various metabolic enzymes, a susceptibility more

prevalent among more hydrophobic compounds. Elevated intestinal absorption, absence of active efflux, and stringent peripheral confinement all affirm compound **7a** as a highly attractive candidate for subsequent *in vivo* pharmacokinetic assessment.

Drug Likeness

The comprehensive assessment of compound 7a against known drug-likeness criteria indicates its significant potential for pharmaceutical development. It meets the stringent benchmark standards of medicinal chemistry. This molecule adheres to all of Lipinski's Rule of Five criteria, including molecular weight, hydrogen bond donors, and hydrogen bond acceptors. This exemplary adherence is also evidenced by additional rigorous drug-likeness criteria, including the Ghose, Veber, Egan, and Muegge factors. These models utilize distinct structural and electronic parameters for drug viability predictions, and the unanimous endorsement of compound 7a across all five filters underscores the resilience of its molecular architecture. This consistent validation indicates that the chemical structure is meticulously adjusted to circumvent the common structural alarms that lead to early-stage attrition in drug research.

Compound **7a** has a bioavailability score of 0.55, indicating its overall drug-likeness. This standard score predicts that 55% of the substances will have an oral bioavailability fraction better than 10% in rats, which is well linked with good human pharmacokinetics. The score indicates a harmonious convergence of the compound's structural features, including a balanced molecular weight (427.15 g/mol), moderate lipophilicity (consensus Log P = 1.61), and controlled topological polar surface area (124.25 Å2). Compound **7a** is a structurally disciplined lead candidate that passes these prediction filters without a

single violation and possesses the drug-like properties required for *in vivo* ADME characterization and preclinical effectiveness studies.

Medicinal Chemistry

The medicinal chemistry compatibility and structural alert evaluation of compound 7a demonstrates a distinct profile, achieving a balance between structural innovation and chemical reactivity considerations. The Pan-Assay Interference Compounds (PAINS) filter yielded a score of zero, signifying that the molecule lacks substructures that could lead to false positive results in biological experiments, attributable to non-specific binding, micelle formation, or chemical aggregation. Nonetheless, screening against the more rigorous Brenk database revealed four specific structural alerts: a hydroxamic acid moiety, an iodine atom, a nitro group, and an oxygen-nitrogen single bond. These specific functional groups are frequently highlighted due to their susceptibility to metabolic alteration, propensity for chemical reactivity, or possible mutagenicity. The lack of PAINS alerts signifies that compound **7a** will yield dependable, non-artifactual data in in vitro screening, whereas the existence of Brenk alerts denotes metabolic hot spots that require meticulous monitoring in further optimization stages.
